# Nickel-catalyzed hydroxymethylation with α-silicon *N*-methoxyphthalimides *via* radical Brook rearrangement

**DOI:** 10.1039/d6sc00986g

**Published:** 2026-03-30

**Authors:** Xiao-Bo Liu, Muhammad Bilal, Jiaying Zuo, Ya-Xin Yu, Yu-Juan Wu, Boming Shen, Peng-Hui Shen, Hua-Jian Xu, Yu-Feng Liang

**Affiliations:** a School of Chemistry and Chemical Engineering, Shandong University Jinan 250100 China yfliang@sdu.edu.cn; b Henan-Macquarie University Joint Centre for Biomedical Innovation, School of Life Sciences, Henan University Kaifeng 475004 China; c Department of Chemistry, Southern University of Science and Technology Shenzhen 518055 China shenbm@sustech.edu.cn; d School of Food and Biological Engineering, Hefei University of Technology Hefei 230009 China hjxu@hfut.edu.cn

## Abstract

The hydroxymethyl group is an important functional motif frequently found in the core structures of natural products and drugs. However, efficient and general methods for its direct introduction remain underexplored, especially within reductive cross-electrophile coupling frameworks. Herein, we report a nickel-catalyzed reductive hydroxymethylation of aryl halides and triflates enabled by a radical Brook rearrangement strategy. Key to this method is a newly designed, bench-stable α-trialkylsilyl *N*-methoxyphthalimide reagent, which acts as a masked hydroxymethyl radical precursor upon reductive N–O bond cleavage and subsequent 1,2-radical Brook rearrangement. The reaction proceeds under mild conditions, exhibits broad functional-group tolerance, and is applicable to a wide range of aryl bromides, iodides, triflates, heteroaryl substrates, and complex bioactive derivatives. Mechanistic studies support a radical pathway involving zinc-mediated single-electron transfer, alkoxyl radical formation, Brook rearrangement, and nickel-catalyzed cross-electrophile coupling. The synthetic utility of this protocol is further demonstrated through gram-scale synthesis and downstream diversification, highlighting its potential for late-stage hydroxymethylation and applications in medicinal chemistry.

## Abstract 2

Nickel-catalyzed reductive hydroxymethylation of aryl electrophiles with α-silicon *N*-methoxyphthalimides *via* a radical Brook rearrangement enables late-stage, functional-group-tolerant installation of hydroxymethyl groups.

## Graphical abstract

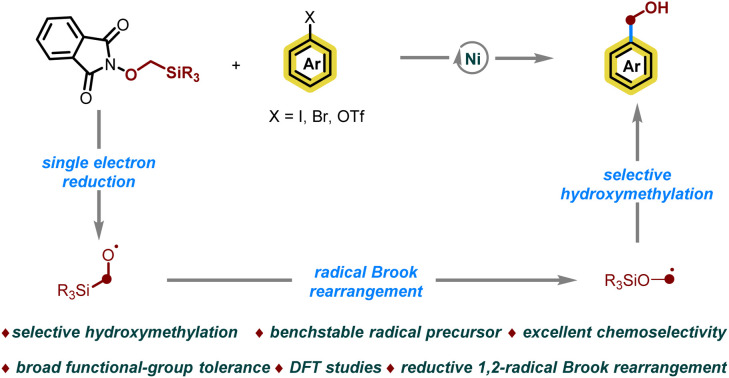

## Introduction

**Scheme 1:**
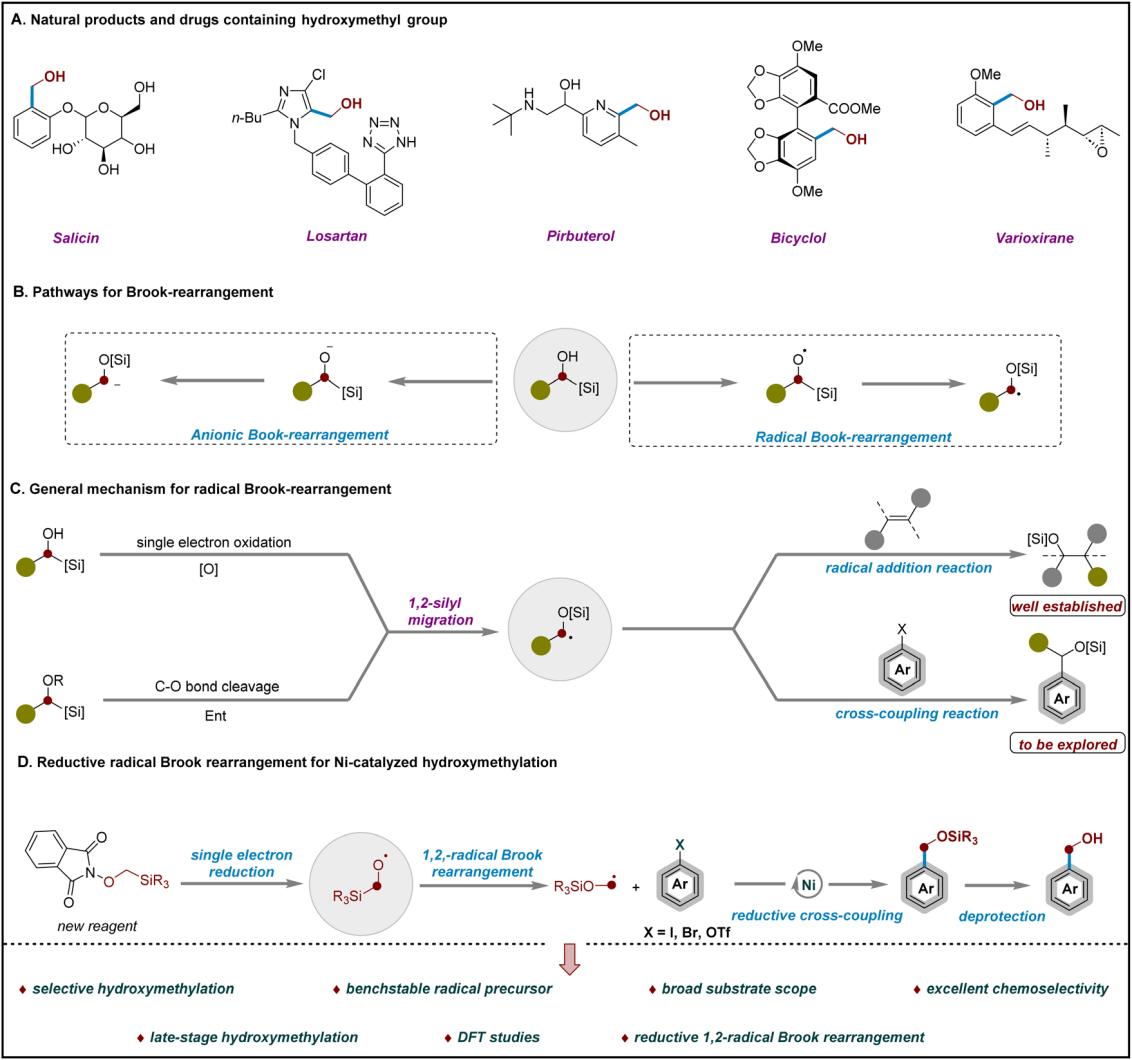
Hydroxymethylation: background and current perspectives.

The direct and efficient interconversion of important functional groups has long been a central theme in organic synthesis. Among them, the hydroxymethyl (–CH_2_OH) group represents an essential structural motif that frequently appears in the core scaffolds of natural products and pharmaceutical compounds (Scheme 1A). This functional group not only significantly influences the physicochemical properties of parent molecules but also serves as a versatile handle for downstream synthetic elaboration.^1–8^ Despite its apparent simplicity, the selective introduction of hydroxymethyl groups remains challenging due to the high reactivity and redox sensitivity of the –CH_2_OH unit, along with issues related to over-functionalization and poor chemoselectivity.^9–12^ Although various methods have been developed to access hydroxymethyl groups from diverse functional precursors including carboxylic acids, amides, ketones, and aldehydes,^13–20^ mild and general strategies for their direct installation remain highly desirable. In principle, a nickel-photoredox strategy could generate an α-alkoxy radical from methanol *via* catalytic hydrogen atom transfer (HAT). However, benzylic C–H bonds are relatively weak, rendering the resulting benzylic alcohol products more prone to HAT and potentially leading to selectivity issues. In contrast, the radical Brook rearrangement strategy enables the controlled generation of the hydroxymethyl radical and thus ensures enhanced chemoselectivity.^21–23^

The Brook rearrangement^24–26^ represents a fundamental transformation in organic chemistry, characterized by the base-mediated intramolecular [1,2]-anionic migration of a silyl group from carbon to oxygen, a process widely applied in synthetic methodology.^27–33^ In contrast, the radical Brook rearrangement remains considerably underexplored, primarily due to the difficulty in generating and controlling alkoxyl radicals under mild conditions (Scheme 1B).^34–42^ A notable advance was reported by Smith and co-workers in 2017, who achieved a formal radical Brook rearrangement of α-silyl alcohols *via* photocatalytic oxidation.^43^ More recently, Shen and co-workers established robust photocatalytic systems that efficiently generate alkoxyl radicals, demonstrating the synthetic utility of radical Brook rearrangements in constructing cyclobutanols, olefins, and functionalized cyclopentanols.^44–51^ Subsequent contributions from Zhang,^52^ Shu,^53^ Wang,^54^ and Glorius^55^ further expanded the scope and applicability of this transformation (Scheme 1C). Despite these advances, the application of radical Brook rearrangements to hydroxymethylation of aryl electrophiles remains a significant challenge. This stems from the inherent instability of hydroxymethyl radicals and the difficulty of synchronizing silyl migration with controlled C–O bond formation, which limits efficient and selective installation of the –CH_2_OH group on aromatic rings.

Cross-electrophile coupling has emerged as a powerful strategy for bond formation under reductive conditions, utilizing the broad availability and enhanced stability of electrophiles relative to nucleophiles. This approach streamlines synthesis, lowers cost, and serves as a versatile platform for constructing C–C, C–N, and C–O bonds, facilitating the modular assembly of complex molecular architectures.^56–63^ In this context, the relatively weak N–O bond can undergo single-electron transfer (SET) and homolytic cleavage, generating radicals that enable redox-active transformations. Alkoxyphthalimides have proven to be effective precursors for alkoxyl radicals, undergoing reductive N–O bond cleavage under mild photoredox or electrochemical conditions.^64–79^ Notably, MacMillan and co-workers recently reported the use of *N*-siloxyphthalimide derivatives as the first reductively activated halogen-atom transfer (XAT) reagents for the photoredox-mediated cross-coupling of tertiary alcohols with alkyl bromides.^80^

Motivated by these elegant advances,^43–80^ we envisioned that α-silicon *N*-methoxyphthalimides could serve as precursors to enable hydroxymethylation of aryl electrophiles *via* a sequence comprising single-electron reduction, radical Brook rearrangement, reductive cross-coupling, and final silyl ether deprotection. Guided by this hypothesis, we designed and synthesized a new, operationally simple, bench-stable reagent. This reduction-initiated strategy provides a practical and versatile platform for the site-selective installation of hydroxymethyl groups on aryl electrophiles *via* radical Brook rearrangement (Scheme 1D). Key advantages of this approach include: (1) first reductive hydroxymethylation of aryl halides and triflates enabled by a radical Brook rearrangement; (2) development of a bench-stable α-TMS *N*-methoxyphthalimide reagent as a masked hydroxymethyl radical precursor; (3) broad substrate scope, encompassing complex molecules, along with utility in late-stage functionalization and diversification; (4) excellent functional-group tolerance, accommodating halides, Bpin, free NH/OH groups, and other sensitive motifs; (5) mechanistic studies supporting a pathway involving zinc-assisted SET, alkoxyl radical formation, and 1,2-radical Brook rearrangement.

## Results and discussion

We initiated our investigation into cross-electrophilic hydroxymethylation *via* a 1,2-radical Brook rearrangement using 2-((trimethylsilyl)methoxy)isoindoline-1,3-dione (2a), easily prepared from *N*-hydroxyphthalimide and iodomethyl trimethylsilane under basic conditions (see SI for more details), as the alkoxyl radical precursor and 4-bromo-1,1′-biphenyl (1a) as the model electrophile (Table 1). After extensive optimization, the hydroxymethylated product 3 was obtained in 78% yield under the standard conditions employing NiBr_2_·DME (10 mol%) with dtbpy (12 mol%) as ligand, Zn as reductant, KI and TMSCl (1.0 equiv. each) as additives, and DMA as solvent at 35 °C for 12 h followed by TBAF-mediated desilylation at 0 °C (entry 1). Replacement of NiBr_2_·DME with Ni(COD)_2_ led to a reduced yield (39%) (entry 2), while other cross-electrophile coupling catalysts such as CoCl_2_ and CrCl_2_ proved ineffective (entry 3). Ligand evaluation revealed that bipyridine ligands exhibited superior performance relative to phenanthrolines and tridentate nitrogen ligands, whereas phosphine ligands completely suppressed reactivity (entry 4). Both TMSCl and KI were crucial for efficient conversion; alternative additives such as TESCl, LiBr, or MgCl_2_ resulted in diminished yields, and omission of KI reduced the yield to 46% (entries 5–7). KI may promote reactivity *via* halogen exchange to form a more reactive alkyl iodide, while LiBr is proposed to modulate nickel's Lewis acidity or stabilize coordinated intermediates.^56^ Temperature variation revealed that either lowering the temperature to ambient conditions or increasing it to 60 °C significantly suppressed product formation (entries 8–9). Zinc was identified as the optimal reductant, as substitution with Mn or Fe led to decreased efficiency or no reaction, respectively (entries 10–11). Solvent screening further established DMA as optimal, with DMSO giving lower yields and DCE completely inhibiting the reaction (entries 12–13). Control experiments confirmed that the nickel catalyst, reductant, and TMSCl are all indispensable for product formation (entries 14–15). Finally, switching the radical precursor to 2b or 2c resulted in comparable reactivity to 2a, indicating the generality of the alkoxyl radical precursor class (entries 16–17). Notably, the –SiMe_3_ group form 2a readily undergoes deprotection to give the final hydroxymethylation product during the workup, whereas the –SiMe_2_Ph group from 2b remains stable under these conditions, enabling isolation of the corresponding silyl ether intermediate.

**Table 1:** Optimization of the reaction conditions^a^

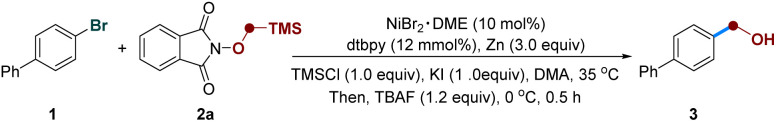		
Entry	Variation from the standard conditions	Yield of 3^b^
1	None	78%
2	Ni(COD)_2_ instead of NiBr_2_·DME	39%
3	CrCl_2_ or CoCl_2_ instead of NiBr_2_·DME	N.D.
4	L2–L10 as ligand	Trace-68%
5	TESCl instead of TMSCl	51%
6	LiBr or MgCl_2_ instead of KI	63%, 41%
7	Without KI	46%
8	*T* = 25 °C	60%
9	*T* = 60 °C	55%
10	Fe instead of Zn	N.D.
11	Mn instead of Zn	51%
12	DCE instead of DMA	Trace
13	DMSO instead of DMA	46%
14	Without TMSCl	Trace
15	Without [Ni] or Zn	N.D.
16	2b instead of 2a	71%
17	2c instead of 2a	66%
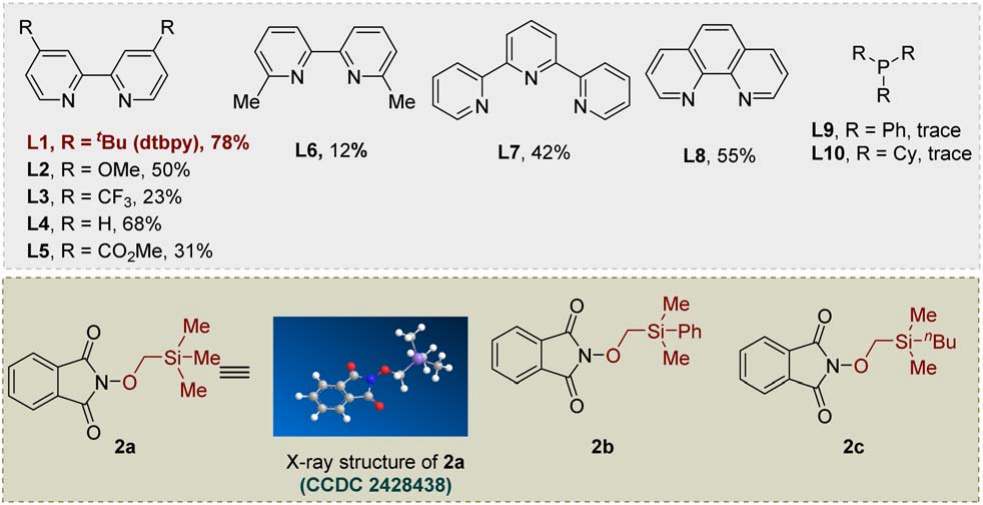		

a Reaction conditions: 1a (0.1 mmol), 2a (0.2 mmol, 2.0 equiv.), cat. (10 mol%), ligand (12 mol%), Zn (3.0 equiv.), TMSCl (1.0 equiv.) and KI (1.0 equiv.) in DMA (0.5 mL) at 35 °C for 8 h under N_2_; then TBAF (1.2 equiv.), 0 °C, 0.5 h.

b GC yields.

**Scheme 2:**
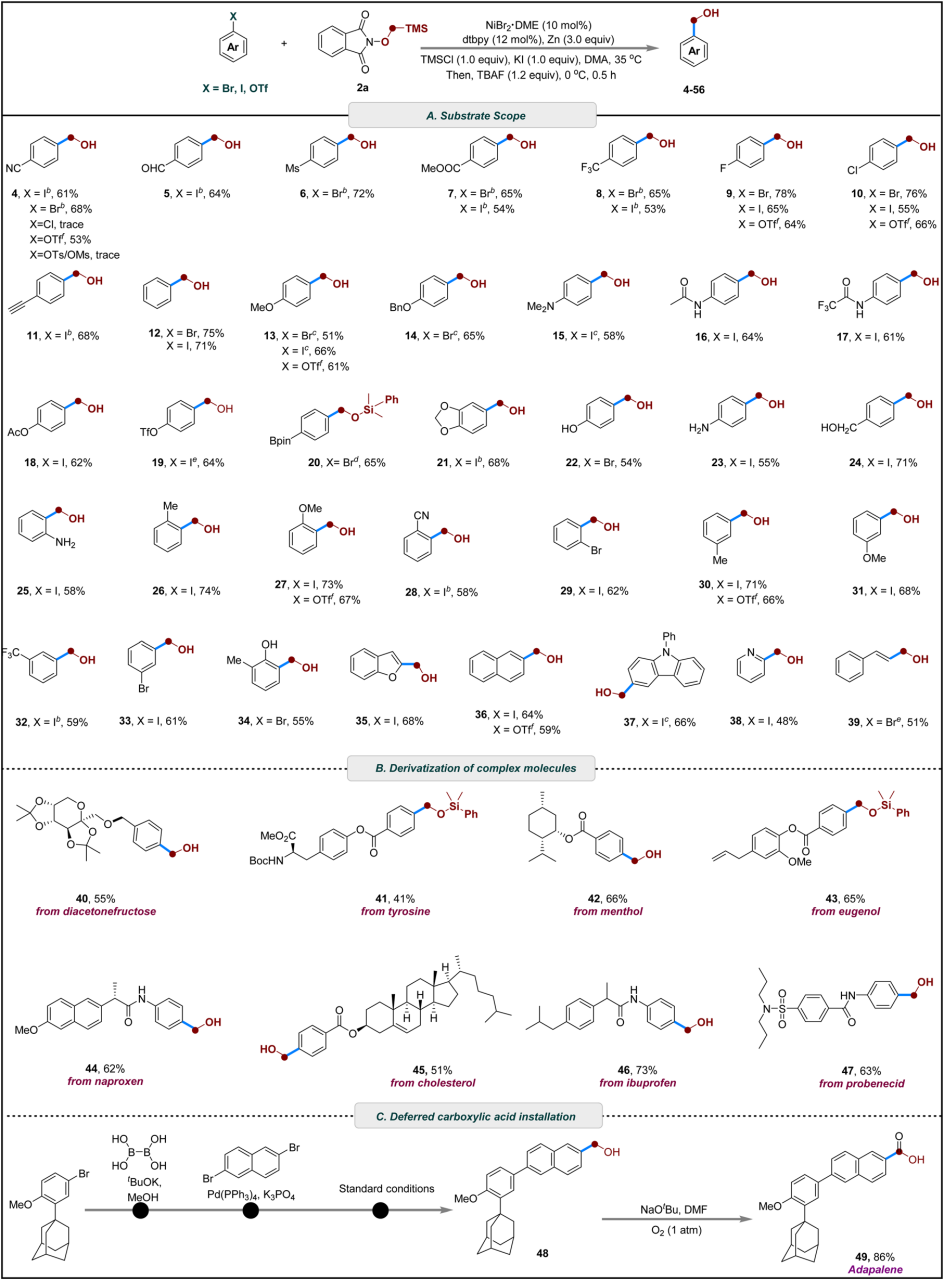
Scope of substrates. ^*a*^ Reaction conditions: Ar-X (0.2 mmol), 2a (0.4 mmol, 2.0 equiv.), NiBr_2_(DME) (10 mol%), dtbpy (12 mol%), TMSCl (1.0 equiv.), Zn (3.0 equiv.) and KI (1.0 equiv.) in DMA (1.0 mL) at 35 °C for 8 h under N_2_; then TBAF (1.2 equiv.), 0 °C, 0.5 h; isolated yields. ^*b*^ At 25 °C. ^*c*^ At 40 °C. ^*d*^2b instead of 2a. ^*e*^ Reaction time = 3 h, ^*f*^ NiBr_2_·bpy (10 mol%), NaI (1.0 equiv.), 40 °C.

With the optimized conditions in hand, we next evaluated the generality and functional-group tolerance of the radical Brook rearrangement-mediated cross-electrophilic hydroxymethylation (Scheme 2). A range of electron-deficient aryl bromides, bearing substituents such as cyano (4), fluoro (9), mesyl (6), chloro (10), trifluoromethyl (8), and ester (7) groups, underwent smooth conversion to the corresponding benzyl alcohols in 65–78% yields. Notably, substrates containing a formyl group, despite its weak C–H bond and typical incompatibility with radical hydrogen-abstraction processes, were well tolerated and afforded the desired product without observable side reactions (5). Alkynyl functionality was also compatible: a TMS-protected alkyne was smoothly hydroxymethylated, and subsequent fluoride-mediated desilylation delivered the free alkynyl benzyl alcohol in 68% yield (11). Electron-rich aryl halides participated efficiently as well, including those with methoxy (13), dimethylamino (15), phenyl (12), benzyloxy (14), acetamido (16), trifluoroacetamido (17), acetoxy (18), triflate (19), and methylenedioxy (21) substituents. Importantly, the protocol exhibited excellent tolerance toward synthetically sensitive boronate ester group (20). Interesting, when both iodine and bromine sites are present on the aromatic ring, hydroxymethylation occurs with high selectivity at the iodine position (29, 33). Remarkably, under shortened reaction times, 4-iodophenyl triflate selectively delivered the hydroxymethylated product 19 in 64% yield within 3 h, illustrating pronounced chemoselectivity between iodide and triflate in this cross-electrophile coupling system. Substrates bearing protic functional groups were also accommodated well. Free amines (23, 25) and phenolic or aliphatic hydroxyl groups (22, 24, 34) did not interfere with the transformation. Substitution patterns had minimal impact on efficiency: *ortho*- (25–29), *meta*- (30–33), and *para*-substituted (4–20) aryl halides all provided the desired products in fair to good yields. Furthermore, heteroaryl iodides derived from benzofuran (35), carbazole (37), and pyridine (38) served as competent coupling partners, highlighting the broad applicability of the method. The strategy could also be extended to alkenyl electrophiles, affording cinnamyl alcohol in moderate yield (39).

Having established a robust protocol for aryl halides, we next investigated the use of aryl trifluoromethanesulfonates as coupling partners. With minor optimization, both electron-rich and electron-deficient aryl triflates underwent efficient hydroxymethylation, with the substitution position showing little effect on the reaction outcome (4, 9, 10, 13, 27, 30, 36). These results further underscore the versatility of this cross-electrophilic strategy for installing hydroxymethyl groups from diverse electrophilic precursors (Scheme 2A).

The catalytic system also exhibited robustness and synthetic utility in the late-stage functionalization of biologically relevant scaffolds. Ether derivatives of fructose underwent reductive cross-electrophilic coupling to furnish the desired hydroxymethylated product in moderate yield (40). Additionally, ester derivatives derived from tyrosine (41), menthol (42), eugenol (43), and cholesterol (45) proved to be effective substrates. Moreover, amide derivatives of naproxen (44), ibuprofen (46), and probenecid (47) were also compatible with the reaction conditions, further demonstrating the broad applicability of the method (Scheme 2B).

To further demonstrate the practical utility of this method, a gram-scale reaction was conducted, providing 2-(hydroxymethyl)phenol in 65% yield, which underscores the robustness and scalability of the transformation. Subsequent downstream derivatizations of 2-(hydroxymethyl)phenol were performed to illustrate its synthetic flexibility (see SI for details). Selective benzylic oxidation to afford the corresponding salicylic acid^81^ and salicylaldehyde^82^ in 88% and 92% yield, respectively. Further functionalization of 2-(hydroxymethyl)phenol also furnished cyanophenol.^83^ In addition, intramolecular cyclization in the presence of formic acid to afford 2-coumaranone. Moreover, 1,2-benzisoxazole was synthesized through a two-step, one-pot sequence involving initial oxidation of followed by cyclization.^84,85^ Notably, this strategy offers a practical alternative route to adapalene (49),^86–89^ in which the hydroxymethyl group (48) is introduced at a late-stage and subsequently oxidized to the corresponding carboxylic acid (Scheme 2C). Masking the carboxyl functionality in this manner avoids the inherent incompatibility of carboxylic acids with reductive cross-coupling conditions, including catalyst poisoning, acid–base side reactions, and the need for repeated protection–deprotection steps during multistep synthesis.^90^

**Scheme 3:**
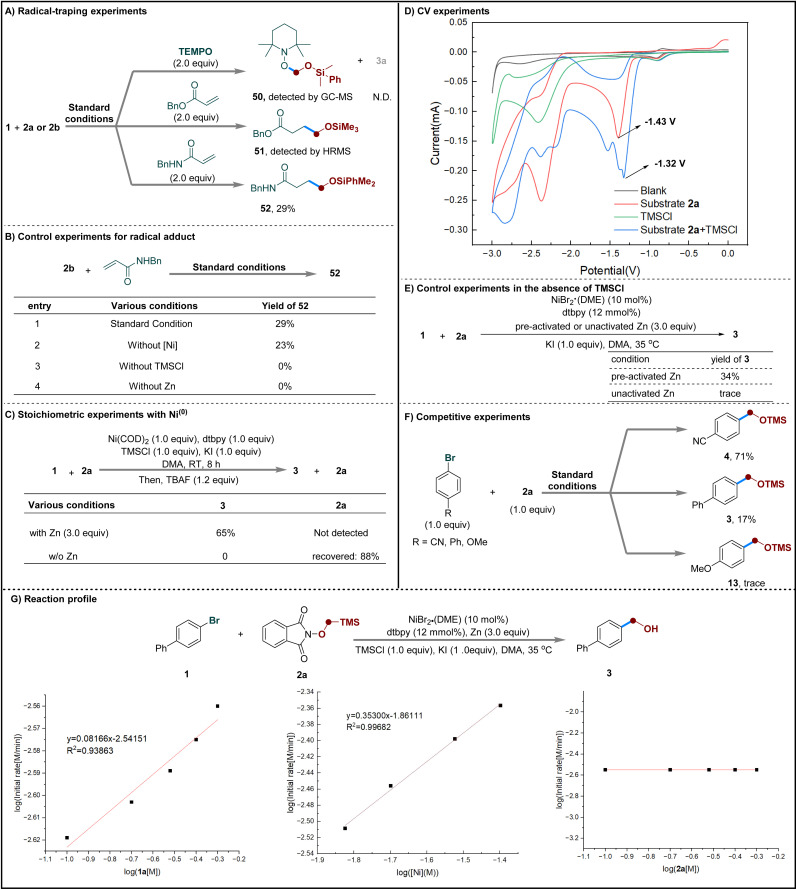
Mechanistic studies.

To gain further mechanistic insight, a series of experiments were performed (Scheme 3). First, the addition of radical scavengers (TEMPO or a Michael acceptor) completely suppressed product formation. The corresponding radical adducts (50–52) were detected by HR-MS, supporting the involvement of radical intermediates in the transformation (Scheme 3A).^66^ Control experiments showed that both zinc powder and TMSCl are essential for radical generation from precursor 2b, whereas the nickel catalyst is not required (Scheme 3B). Stoichiometric experiments with Ni^(0)^ suggested that zinc acts not only as the terminal reductant but may also participate directly in key elementary steps of the catalytic cycle (Scheme 3C).^69,91^ Cyclic voltammetry (CV) studies further supported this conclusion, showing that TMSCl lowers the reduction potential of 2a and thus facilitates its single-electron reduction (Scheme 3D).^92^ Further control experiments were performed using pre-activated or unactivated Zn in the absence of TMSCl (Scheme 3E). While the reaction with pre-activated Zn afforded the desired product in 34% yield, only trace amounts were observed with unactivated Zn under identical conditions. These findings suggest that TMSCl plays a dual role in the reaction: (1) activating zinc by removing surface oxides and (2) weakly coordinating to the substrate to facilitate single-electron reduction. Collectively, these observations support the involvement of TMSCl in promoting the Zn-mediated formation of the alkoxyl radical. Competition experiments using electronically differentiated aryl bromides indicate that electron-withdrawing groups appear to promote faster reaction rates compared to electron-donating groups (Scheme 3F).^93^ Kinetic studies showed that the reaction follows first-order kinetics with respect to both the nickel catalyst and the aryl bromide, while exhibiting zero-order dependence on the radical precursor. This suggests that radical generation is a fast step in the overall process (Scheme 3G).

**Fig. 1:**
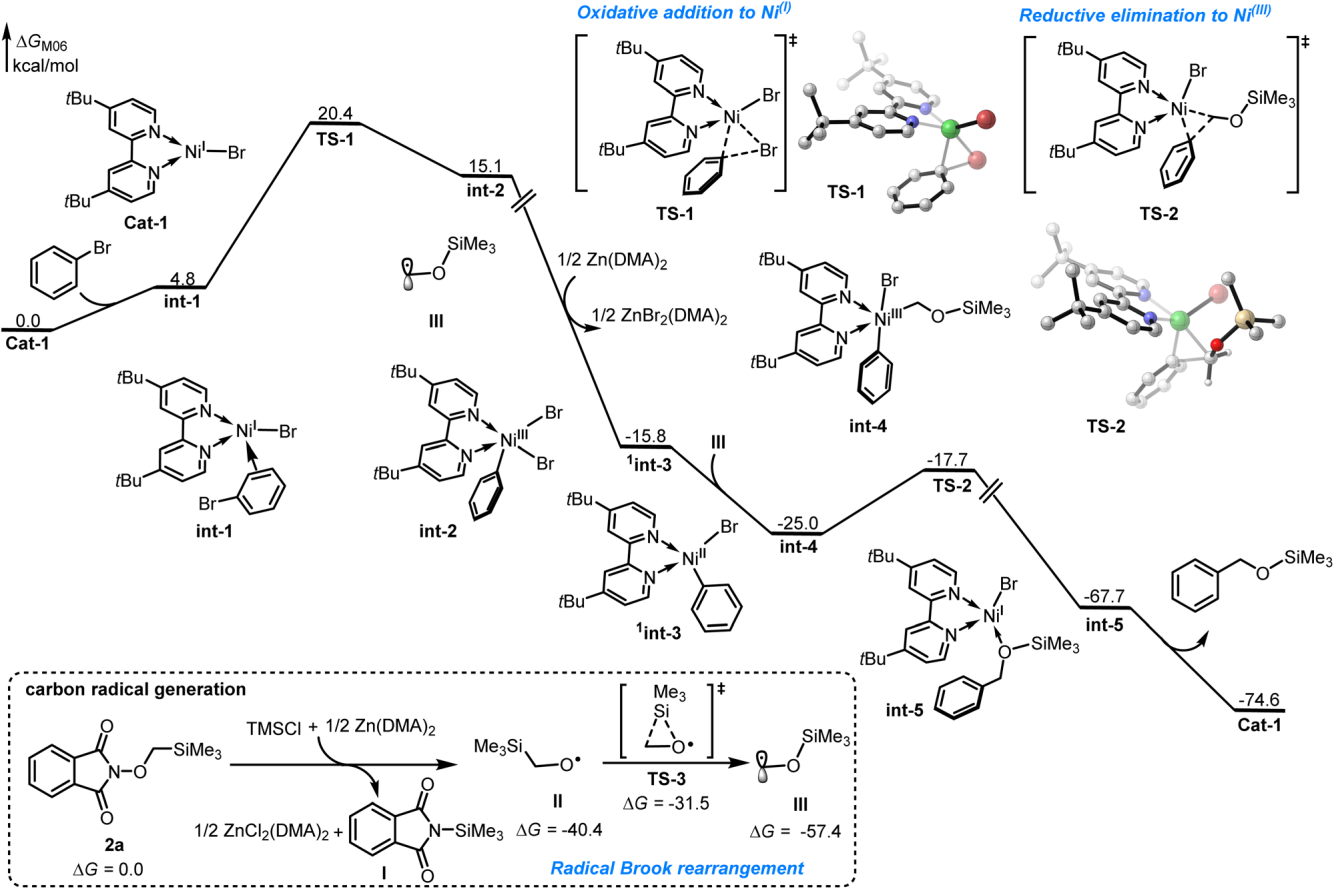
Free-energy profile for the nickel-catalyzed hydroxymethylation reaction. The energy values are given in kcal/mol and represent the relative free energies calculated at M06/6-311+G(d,p)-SDD-SMD(DMA)//B3LYP-D3(BJ)/6-31G(d)-SDD level of theory.

Density functional theory (DFT) calculations were performed to elucidate the reaction mechanism in the nickel-catalyzed hydroxymethylation reaction, using bromobenzene and α-TMS *N*-methoxyphthalimides 2a as the model substrates (see SI for details). As shown in Fig. 1, the combination of Zn and TMSCl could induce homolytic N–O bond cleavage of the reactant 2a, leading to the formation of oxygen radical II with the release of 40.4 kcal mol^−1^ free energy, followed by the radical brook rearrangement *via* transition state TS-3 with energy barrier of 8.9 kcal mol^−1^ to generate a stabilized carbon radical III. The catalytic cycle is initiated by *in situ* generation of the Ni^(I)^ species Cat-1 through the reduction of NiBr_2_ by zinc powder. The coordination of bromobenzene to species Cat-1 results in the formation of intermediate int-1 with free energy increase 4.8 kcal mol^−1^. Subsequent oxidative addition of bromobenzene to the Ni^(I)^ center occurs *via* a three-membered-ring transition state TS-1 with an energy barrier of 15.6 kcal mol^−1^ to reversibly generate Ni^(III)^ intermediate int-2, which is endergonic by 10.3 kcal mol^−1^ free energy. Subsequently, the reduction of intermediate int-2 by zinc powder would form Ni^(II)^ intermediate ^1^int-3 with the release of 30.9 kcal mol^−1^ free energy. Next, the radical addition of carbon radical III to the Ni^(II)^ center generates Ni^(III)^ intermediate int-4, which is exothermic by 9.2 kcal mol^−1^. The subsequent reductive elimination takes place through a three-membered-ring transition state TS-2 with an activation free energy of 7.3 kcal mol^−1^ to irreversibly generate intermediate int-5, followed by the dissociation of the coupling product to regenerate the active Ni^(I)^ species Cat-1.

**Scheme 4:**
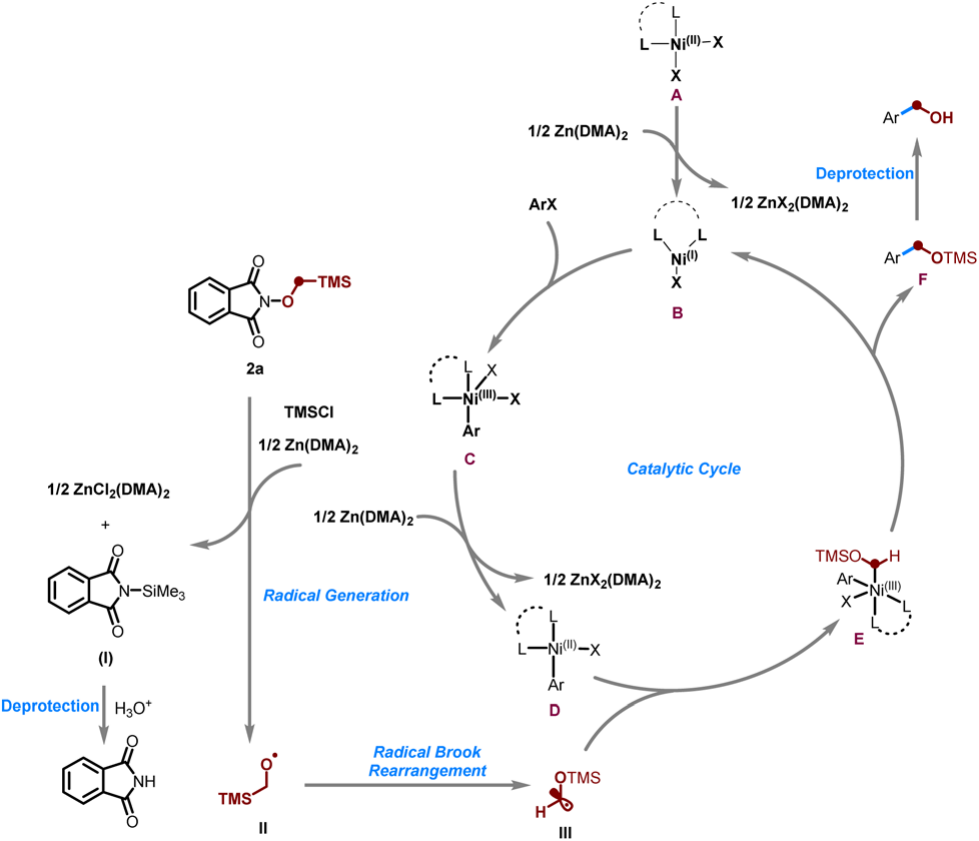
Proposed catalytic cycle.

Based on the above experimental observations and literature precedents,^48,68,94–98^ a plausible reaction mechanism is proposed (Scheme 4). Initially, α-TMS *N*-methoxyphthalimide 2a undergoes single-electron transfer reduction by zinc powder, facilitated by TMSCl, to generate *N*-(trimethylsilyl)phthalimide I and the corresponding α-TMS methoxyl radical II. Driven by the greater bond strength of Si–O relative to C–Si, radical II undergoes a 1,2-radical Brook rearrangement to afford the α-OTMS methyl radical III. In the catalytic cycle, reduction of the Ni^(II)^ precatalyst A by zinc yields a Ni^(I)^ species B, which undergoes oxidative addition with the aryl halide to give the aryl-Ni^(III)^ intermediate C. Subsequent reduction by zinc affords the Ni^(II)^ species D, which captures radical III to form the high-valent Ni^(III)^ intermediate E. Reductive elimination from E then delivers the coupling product F, which upon deprotection yields the final hydroxymethylated product and concurrently regenerates the Ni^(I)^ species for the next catalytic cycle.

## Conclusions

In summary, we have developed a robust nickel-catalyzed reductive hydroxymethylation of aryl electrophiles *via* a reduction-triggered radical Brook rearrangement. Using an easily accessible, bench-stable α-TMS *N*-methoxyphthalimide reagent, this approach enables the controlled generation of hydroxymethyl radicals through reductive N–O bond cleavage, thereby addressing long-standing challenges in radical hydroxymethylation. The method exhibits broad substrate scope, excellent tolerance toward sensitive functional groups, and compatibility with aryl halides, triflates, heterocycles, and complex molecular frameworks. Mechanistic studies support a cooperative zinc–nickel catalytic system involving alkoxyl radical formation, Brook rearrangement, and high-valent nickel intermediates. The successful late-stage functionalization of biologically relevant molecules and gram-scale synthesis further highlight the synthetic utility of this approach. We anticipate that this reductive Brook rearrangement platform will open new avenues for C1 and hydroxyalkyl radical chemistry in cross-electrophile coupling and late-stage molecular diversification.

## Author contributions

Y.-F. L. conceptualized of the project aims. X.-B. L., M. B., Y.-X. Y., Y.-J. W. and P.-H. S. carried out the design, chemical synthesis and characterization of products. J. Z. and B. S. performed the DFT studies. B. S., H.-J. X. and Y.-F. L. supervised the research. All authors participated in the data analysis and manuscript preparation.

## Conflicts of interest

There is no conflict of interest.

## Supplementary Material

SC-017-D6SC00986G-s001

SC-017-D6SC00986G-s002

## Data Availability

CCDC 2428438 contains the supplementary crystallographic data for this paper.^99^ Supplementary information (SI): full experimental procedures, reactions optimizations, ^1^H NMR, ^13^C NMR and ^19^F NMR spectra. See DOI: https://doi.org/10.1039/d6sc00986g.

## References

[cit1] Cheng Y., Xie N., Jin P., Wang T. (2015). Cell Biochem. Funct..

[cit2] Fu S., Wu H., Zhang H., Lian C. G., Lu Q. (2017). Oncotarget.

[cit3] White M. C., Zhao J. (2018). J. Am. Chem. Soc..

[cit4] Roughley S. D., Jordan A. M. (2011). J. Med. Chem..

[cit5] Brown D. G., Boström J. (2016). J. Med. Chem..

[cit6] Nafiu S. A., Ajeebi A. M., Alghamdi H. S., Aziz M. A., Shaikh M. N. (2023). Asian J. Org. Chem..

[cit7] Li J., Huang C.-Y., Li C.-J. (2022). Angew. Chem., Int. Ed..

[cit8] Blakemore D. C., Castro L., Churcher I., Rees D. C., Thomas A. W., Wilson D. M., Wood A. (2018). Nat. Chem..

[cit9] Tanwar L., Börgel J., Ritter T. (2019). J. Am. Chem. Soc..

[cit10] Liu W., Groves J. T. (2015). Acc. Chem. Res..

[cit11] Punniyamurthy T., Velusamy S., Iqbal J. (2005). Chem. Rev..

[cit12] Yan M., Lo J. C., Edwards J. T., Baran P. S. (2016). J. Am. Chem. Soc..

[cit13] Han C., Peng Y., Li G., Kong Q., Huo X., Zhang W. (2025). Nat. Commun..

[cit14] Caiger L., Sinton C., Constantin T., Douglas J. J., Sheikh N. S., Juliá F., Leonori D. (2021). Chem. Sci..

[cit15] Kawamoto T., Fukuyama T., Ryu I. (2012). J. Am. Chem. Soc..

[cit16] Li W. and Wu X.-F., The Chemical Transformations of C1 Compounds, John Wiley & Sons, Weinheim Germany, 2022, ch. 5, vol. 1, pp. 157–247

[cit17] Ong D. Y., Yen Z., Yoshii A., Revillo Imbernon J., Takita R., Chiba S. (2019). Angew. Chem., Int. Ed..

[cit18] Quevedo-Flores B., Bosque I., Gonzalez-Gomez J. C. (2024). Org. Lett..

[cit19] Amberchan G., Snelling R. A., Moya E., Landi M., Lutz K., Gatihi R., Singaram B. (2021). J. Org. Chem..

[cit20] Rastogi A., Kumar M., Kumar Gangwar M., Koley D. (2024). Adv. Synth. Catal..

[cit21] Atkins A. P., Dean A. C., Lennox A. J. J. (2024). Beilstein J. Org. Chem..

[cit22] Zhang J., Li Y., Zhang F., Hu C., Chen Y. (2016). Angew. Chem., Int. Ed..

[cit23] Zhu Y., Wang H., Shu P., Zhang K., Wang Q. (2024). Chin. J. Org. Chem..

[cit24] Gilman H., Wu T. C. (1953). J. Am. Chem. Soc..

[cit25] Brook A. G. (1958). J. Am. Chem. Soc..

[cit26] Shen X. , Zhang Y. and Zhou G., Radical Brook Rearrangement, John Wiley & Sons, Weinheim, 2025

[cit27] Brook A. G. (1974). Acc. Chem. Res..

[cit28] Page P. C. B., Klair S. S., Rosenthal S. (1990). Chem. Soc. Rev..

[cit29] Fleming I., Barbero A., Walter D. (1997). Chem. Rev..

[cit30] Smith A. B., Adams C. M. (2004). Acc. Chem. Res..

[cit31] Zhang H.-J., Priebbenow D. L., Bolm C. (2013). Chem. Soc. Rev..

[cit32] Eppe G., Didier D., Marek I. (2015). Chem. Rev..

[cit33] Deng Y., Smith III A. B. (2020). Acc. Chem. Res..

[cit34] Paredes M. D., Alonso R. (2000). J. Org. Chem..

[cit35] Zhang Y., Chen J.-J., Huang H.-M. (2022). Angew. Chem., Int. Ed..

[cit36] Zhang Y., Zhou G., Liu S., Shen X. (2025). Chem. Soc. Rev..

[cit37] Le C., Chen T. Q., Liang T., Zhang P., MacMillan D. W. (2018). Science.

[cit38] Lovett G. H., Chen S., Xue X.-S., Houk K. N., MacMillan D. W. C. (2019). J. Am. Chem. Soc..

[cit39] Chen X., Gong X., Li Z., Zhou G., Zhu Z., Zhang W., Liu S., Shen X. (2020). Nat. Commun..

[cit40] Li Z., Chen X., Peng C., Xu Y., Wang H., Liu S., Shen X. (2024). ChemCatChem.

[cit41] Lee N., Tan C.-H., Leow D. (2019). Asian J. Org. Chem..

[cit42] Agbaria M., Egbaria N., Nairoukh Z. (2024). Synthesis.

[cit43] Deng Y., Liu Q., Smith III A. B. (2017). J. Am. Chem. Soc..

[cit44] Zhang Y., Zhang Y., Shen X. (2021). Chem Catal..

[cit45] Zhang Y., Zhang Y., Guo Y., Liu S., Shen X. (2022). Chem Catal..

[cit46] Li Z., Zhang Y., Zhang Y., He X., Shen X. (2023). Angew. Chem., Int. Ed..

[cit47] Zhang Y., Zhang Y., Ye C., Qi X., Wu L.-Z., Shen X. (2022). Nat. Commun..

[cit48] Niu Y., Jin C., He X., Deng S., Zhou G., Liu S., Shen X. (2025). Angew. Chem., Int. Ed..

[cit49] Deng S., Peng C., Niu Y., Xu Y., Zhang Y., Chen X., Wang H., Liu S., Shen X. (2024). Acta Chim. Sin..

[cit50] Yang Z., Niu Y., He X., Chen S., Liu S., Li Z., Chen X., Zhang Y., Lan Y., Shen X. (2021). Nat. Commun..

[cit51] He X., Zhao Y., Zhang Z., Shen X. (2022). Org. Lett..

[cit52] Qin T., Xu C., Zhang G., Zhang Q. (2023). Org. Chem. Front..

[cit53] Ouyang X., Shi B., Zhao Y., Zhu Z., Li Z., Yang Y., Shu C. (2024). Chem. Sci..

[cit54] Zhou P., Ding L., Liu Y., Song H., Wang Q. (2024). Org. Lett..

[cit55] Laskar R., Dutta S., Spies J. C., Mukherjee P., Rentería-Gómez Á., Thielemann R. E., Daniliuc C. G., Gutierrez O., Glorius F. (2024). J. Am. Chem. Soc..

[cit56] Ehehalt L. E., Beleh O. M., Priest I. C., Mouat J. M., Olszewski A. K., Ahern B. N., Cruz A. R., Chi B. K., Castro A. J., Kang K. (2024). Chem. Rev..

[cit57] Weix D. J. (2015). Acc. Chem. Res..

[cit58] Poremba K. E., Dibrell S. E., Reisman S. E. (2020). ACS Catal..

[cit59] Knappke C. E. I., Grupe S., Gärtner D., Corpet M., Gosmini C., Wangelin A. J. V. (2014). Chem.–Eur. J..

[cit60] Liu J., Ye Y., Sessler J. L., Gong H. (2020). Acc. Chem. Res..

[cit61] Pang X., Su P.-F., Shu X.-Z. (2022). Acc. Chem. Res..

[cit62] Pan Q., Ping Y., Kong W. (2023). Acc. Chem. Res..

[cit63] Li P., Wang Y., Zhao H., Qiu Y. (2025). Acc. Chem. Res..

[cit64] Salgueiro D. C., Chi B. K., Guzei I. A., García-Reynaga P., Weix D. J. (2022). Angew. Chem., Int. Ed..

[cit65] Gao Y., Zhang B., He J., Baran P. S. (2023). J. Am. Chem. Soc..

[cit66] Lombardi L., Cerveri A., Giovanelli R., Reis M. C., López C. S., Bertuzzi G., Bandini M. (2022). Angew. Chem., Int. Ed..

[cit67] Lee D. S., Soni V. K., Cho E. J. (2022). Acc. Chem. Res..

[cit68] Liu D., Yang K., Fang D., Li S.-J., Lan Y., Chen Y. (2023). Angew. Chem., Int. Ed..

[cit69] Wang Y., Bao P., Dong X., Lan Y., Chen Y. (2025). J. Am. Chem. Soc..

[cit70] Zhang J., Li Y., Zhang F., Hu C., Chen Y. (2016). Angew. Chem., Int. Ed..

[cit71] Li Y.-B., Hu D.-D., Ren W.-R., Liu H., Wang Y.-L., Li K., Ke W.-C., Jin R.-X., Wang X.-S. (2025). Angew. Chem., Int. Ed..

[cit72] Chen F., Xu X., Chu L., Qing F. (2022). Org. Lett..

[cit73] Cong F., Lv X.-Y., Day C. S., Martin R. (2020). J. Am. Chem. Soc..

[cit74] Huihui K. M. M., Caputo J. A., Melchor Z., Olivares A. M., Spiewak A. M., Johnson K. A., DiBenedetto T. A., Kim S., Ackerman L. K. G., Weix D. J. (2016). J. Am. Chem. Soc..

[cit75] Liu X.-B., Liu R.-M., Bao X.-D., Xu H.-J., Zhang Q., Liang Y.-F. (2024). Chin. Chem. Lett..

[cit76] Qiao J.-F., Wang T.-Z., Shen P.-H., Guan Y.-Q., Yu Y.-X., Liang Y.-F. (2025). Green Chem..

[cit77] Wang A.-L., Yao Y.-F., Zhang X.-G., Xu P.-F. (2026). Org. Lett..

[cit78] Bilal M., Fatima K., Wu Y.-J., Guan Y.-Q., Liang Y.-F. (2026). JACS Au.

[cit79] Bilal M., Wu Y.-J., Shen P.-H., Guan Y.-Q., Wang T.-Z., Xu H.-J., Liang Y.-F. (2025). ChemCatChem.

[cit80] Gould C. A., Pace A. L., MacMillan D. W. C. (2023). J. Am. Chem. Soc..

[cit81] Das R., Chakraborty D. (2011). Appl. Organomet. Chem..

[cit82] Fernandes R. A., Kumar P. (2003). Tetrahedron Lett..

[cit83] McAllister G. D., Wilfred C. D., Taylor R. J. (2002). Synlett.

[cit84] Li H.-P., Ai H.-J., Qi X., Peng J.-B., Wu X.-F. (2017). Org. Biomol. Chem..

[cit85] Paschke A.-S. K., Schiele S., Pinard C., Sandrini F., Morandi B. (2025). Chem. Sci..

[cit86] Wang X., Guo X., Wang X., Li C., Wang S., Li H., Gao Y., Li Y., Wang J., Xu H. (2023). RSC Adv..

[cit87] Lim T., Ryoo J. Y., Han M. S. (2020). J. Org. Chem..

[cit88] Barder T. E., Walker S. D., Martinelli J. R., Buchwald S. L. (2005). J. Am. Chem. Soc..

[cit89] Miyaura N., Yanagi T., Suzuki A. (1981). Synth. Commun..

[cit90] Liu Z., Xiang J. (2006). Org. Process Res. Dev..

[cit91] Turro R. F., Wahlman J. L., Tong Z. J., Chen X., Yang M., Chen E. P., Hong X., Hadt R. G., Houk K., Yang Y.-F. (2023). J. Am. Chem. Soc..

[cit92] Aihara Y., Chatani N. (2013). Chem. Sci..

[cit93] Wang M., Zhang C., Ci C., Jiang H., Dixneuf P. H., Zhang M. (2023). J. Am. Chem. Soc..

[cit94] Wang T.-Z., Guan Y.-Q., Zhang T.-Y., Liang Y.-F. (2024). Adv. Sci..

[cit95] Kim S., Goldfogel M. J., Ahern B. N., Salgueiro D. C., Guzei I. A., Weix D. J. (2025). J. Am. Chem. Soc..

[cit96] Wu T., Castro A. J., Ganguli K., Rotella M. E., Ye N., Gallou F., Wu B., Weix D. J. (2025). J. Am. Chem. Soc..

[cit97] Guan Y.-Q., Wang T.-Z., Bilal M., Tan X.-R., Ackermann L., Liang Y.-F. (2025). Nat. Commun..

[cit98] Lin Q., Fu Y., Liu P., Diao T. (2021). J. Am. Chem. Soc..

[cit99] CCDC 2428438: Experimental Crystal Structure Determination, 2026, 10.5517/ccdc.csd.cc2mhzqv

