# Supplementary material for: Nickel-catalyzed hydroxymethylation with α-silicon N-methoxyphthalimides via radical Brook rearrangement
Source: Chem Sci. 2026 Mar 30;17(19):9653–62. doi: 10.1039/d6sc00986g (PMC13034179; doi:10.1039/d6sc00986g)
Supplement: SC-017-D6SC00986G-s001 [file SC-017-D6SC00986G-s001.pdf]

Supplementary Information (SI) for Chemical Science.
This journal is © The Royal Society of Chemistry 2026

Supplementary Information

Nickel-catalyzed hydroxymethylation with α-silicon N-methoxyphthalimides via radical Brook rearrangement

Xiao-Bo Liu,†[a] Muhammad Bilal,†[a] Jiaying Zuo,[b] Ya-Xin Yu,[a] Yu-Juan Wu,[a] Boming Shen,*[c] Peng-Hui Shen,[d] Hua-Jian Xu*[d] and Yu-Feng Liang*[a]

[a]*School of Chemistry and Chemical Engineering, Shandong University, Jinan 250100, China. E-mail: yfliang@sdu.edu.cn*

[b]*Henan-Macquarie University Joint Centre for Biomedical Innovation, School of Life Sciences, Henan University, Kaifeng 475004, China*

[c]*Department of Chemistry, Southern University of Science and Technology, Shenzhen 518055, China. E-mail: shenbm@sustech.edu.cn*

[d]*School of Food and Biological Engineering, Hefei University of Technology, Hefei, 230009, China. E-mail: hjxu@hfut.edu.cn*

† These authors contributed equally to this work.

Table of Contents

1. General remarks S2

2. General procedures S2

3. Optimization of conditions S5

4. Characterization data S10

5. Gram scale reaction and downstream modificatins S21

6. Crystallographic data S24

7. DFT studies S25

8. Mechanistic studies S57

9. References. S61

10. NMR spectra S63

1. General remarks

^{1}H NMR, ^{13}C NMR data were obtained on AVANCE III Bruker 400 and 500 MHz nuclear resonance spectrometers unless otherwise noted. Chemical shifts (in ppm) were referenced to tetramethylsilane (TMS) (δ = 0.00 ppm) in $CDCl_3$. The data of ^{1}H NMR was reported as follows: chemical shift, multiplicity (s = singlet, d = doublet, t = triplet, m = multiplet and br = broad), coupling constant (J values) in Hz and integration. ^{13}C NMR spectra were obtained by the same NMR spectrometers and were calibrated with $CDCl_3$ (δ = 77.16 ppm). Flash chromatography was performed using 300-400 mesh silica gel with the indicated eluent according to standard techniques. Analytical thin-layer chromatography (TLC) was performed on pre-coated, glass-backed silica gel plates. Analysis of crude reaction mixture was done on an Agilent 7890 GC System with an Agilent 5975 Mass Selective Detector. Visualization of the developed chromatogram was performed by UV absorbance (254 nm) unless otherwise noted. High-resolution mass spectral (HRMS) data were recorded on Bruker APEX IV Fourier transform ion cyclotron resonance mass spectrometer using electrospray ionization (ESI) mode.

2. General procedure

2.1. Preparation of Starting Materials

Synthesis of *N*-alkoxyphthalimide Substrates[1]

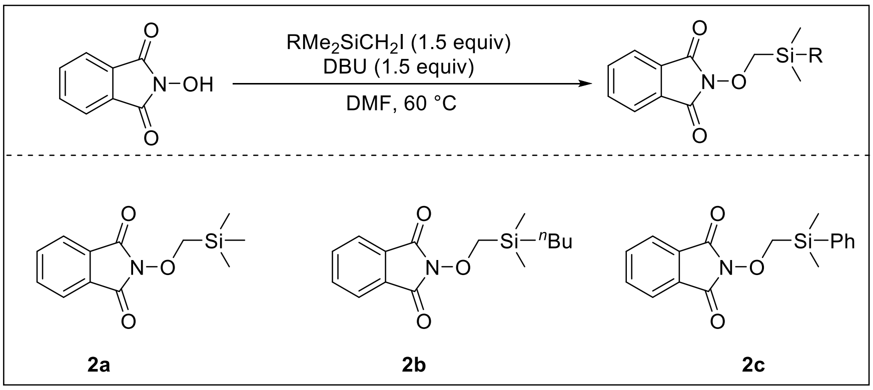

Following the procedure from the literature.[1] *N*-Hydroxyphthalimide (1.63 g, 10 mmol, 1.0 equiv) and RMe_2CH_2I (15 mmol, 1.5 equiv) were added in DMF (50 mL), then DBU (15 mmol, 1.5 equiv) was added with stirring. The suspension was stirred at 60 °C overnight. The resulting solution was taken up in EA (50 mL), washed with water (30 mL) and brine (30 mL) for three times. It was then dried over Na_2SO_4, concentrated in vacuo, purified by column chromatography (EA/PE = 1/5) to afford the *N*-alkoxyphthalimide **2a-2c**.

2-((Trimethylsilyl)methoxy)isoindoline-1,3-dione (2a); **^{1}H NMR** (500 MHz, Chloroform-*d*) δ 7.80 (dd, *J* = 5.5, 3.1 Hz, 2H), 7.72 (dd, *J* = 5.5, 3.1 Hz, 2H), 4.06 (s, 2H), 0.19 (s, 9H); **^{13}C NMR** (126 MHz, Chloroform-*d*) δ 163.8, 134.8, 129.7, 122.3, 74.5, 2.7; **HRMS(ESI)**: Found: m/z 250.0895. Calcd for $C_{12}H_{16}NO_3Si$: $(M+H)^+$ 250.0894.

2-((Butyldimethylsilyl)methoxy)isoindoline-1,3-dione (**2b**); **^{1}H NMR** (500 MHz, Chloroform-*d*) δ 7.80 (dd, *J* = 5.4, 3.2 Hz, 2H), 7.72 (dd, *J* = 5.5, 3.1 Hz, 2H), 7.66 – 7.62 (m, 2H), 7.39 – 7.36 (m, 3H), 4.29 (s, 2H), 0.49 (s, 6H); **^{13}C NMR** (126 MHz, Chloroform-*d*) δ 163.8, 136.3, 134.9,134.4, 130.2, 129.6, 128.6, 123.9, 74.0, 3.9; **HRMS(ESI)**: Found: m/z 312.1050. Calcd for $C_{17}H_{18}NO_3Si$: $(M+H)^+$ 312.1050.

2-((Butyldimethylsilyl)methoxy)isoindoline-1,3-dione (2c); **^{1}H NMR** (500 MHz, Chloroform-*d*) δ 7.63 (dd, *J* = 5.5, 3.1 Hz, 2H), 7.59 – 7.52 (m, 2H), 3.91 (s, 2H), 1.27 – 1.12 (m, 4H), 0.73 (t, *J* = 7.0 Hz, 3H), 0.59 – 0.48 (m, 2H); **^{13}C NMR** (126 MHz, Chloroform-*d*) δ 163.0, 134.1, 128.9, 123.1, 73.5, 26.3, 25.5, 13.6, 13.1, -5.0; **HRMS(ESI)**: Found: m/z 292.1363. Calcd for $C_{15}H_{22}NO_3Si$: $(M+H)^+$ 292.1363.

2.2. Synthesis of aryl halides

Following the procedure from the literature.[2-5]

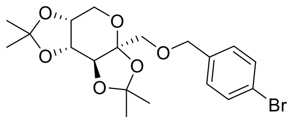

from diacetonefructose

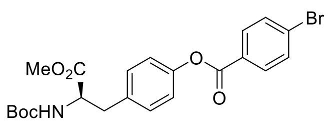

from tyrosine

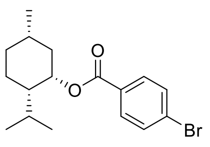

from menthol

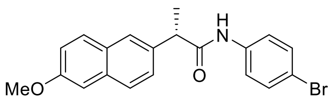

from naproxen

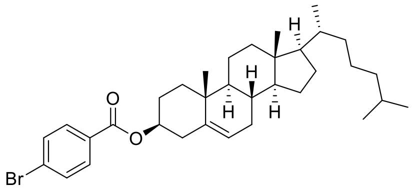

from cholesterol

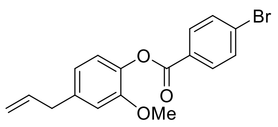

from eugenol

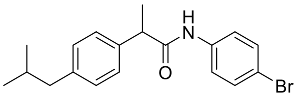

from ibuprofen

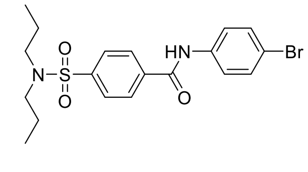

from probenecid

2.3. General procedure for hyrdoxymethylation of aryl halides

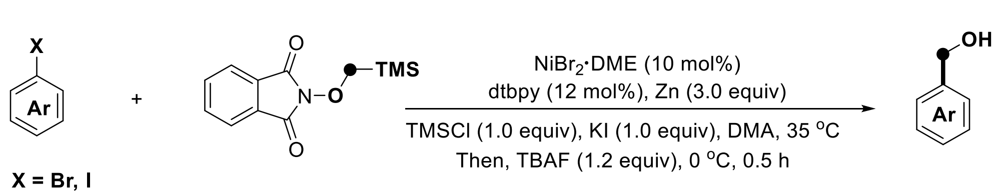

In a nitrogen-filled glovebox (or under a nitrogen atmosphere), an aryl halide (Ar–X, 0.20 mmol), **2a** (0.40 mmol, 2.0 equiv), $NiBr_2$·DME (10 mol%), dtbpy (12 mol%), zinc powder (3.0 equiv), potassium iodide (1.0 equiv), and TMSCl (1.0 equiv) were combined in N,N-dimethylacetamide (DMA, 1.0 mL). The resulting reaction mixture was stirred at 35 °C for 8 h under a nitrogen

atmosphere. After completion, the reaction was cooled to 0 °C and tetrabutylammonium fluoride (TBAF, 1.2 equiv) was added. The mixture was then diluted with ethyl acetate (5 mL), washed with water (5 mL), dried over anhydrous Na_2SO_4, filtered, and concentrated under reduced pressure. The crude product was purified by column chromatography on silica gel.

2.4. General procedure for hyrdoxymethylation of aryl triflates

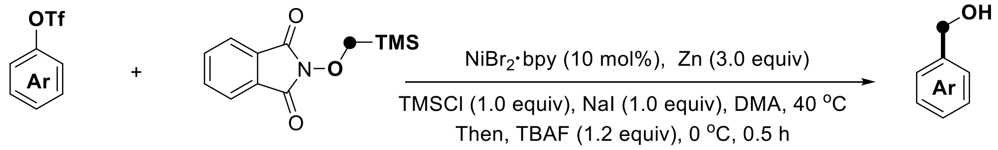

In a nitrogen-filled glovebox (or under a nitrogen atmosphere), an aryl triflates (Ar–OTf, 0.20 mmol), **2a** (0.40 mmol, 2.0 equiv), $NiBr_2$·bpy (10 mol%), zinc powder (3.0 equiv), NaI (1.0 equiv), and TMSCl (1.0 equiv) were combined in N,N-dimethylacetamide (DMA, 1.0 mL). The resulting reaction mixture was stirred at 40 °C for 8 h under a nitrogen atmosphere. After completion, the reaction was cooled to 0 °C and tetrabutylammonium fluoride (TBAF, 1.2 equiv) was added. The mixture was then diluted with ethyl acetate (5 mL), washed with water (5 mL), dried over anhydrous Na_2SO_4, filtered, and concentrated under reduced pressure. The crude product was purified by column chromatography on silica gel.

3. Optimization of the reaction conditions

Table S1. Optimization of the Catalysts

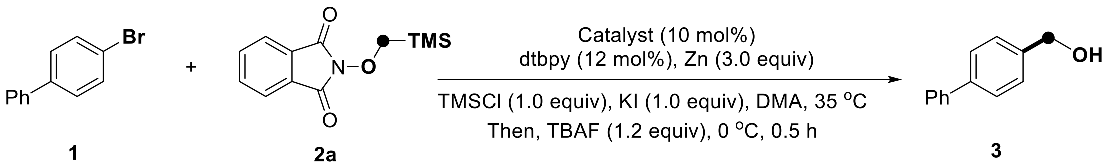

No.	Catalyst	Yield (3) %
1	$NiCl_2$	52
2	$NiBr_2$.DME	78
3	NiI_2	38
4	$NiBr_2$	60
5	$NiCl_2$.dppf	trace
6	$NiCl_2$. 1,10-Phen	52
7	$NiCl_2.6H_2O$	23
8	$Ni(acac)_2$	trace
9	NiF_2	N.D.
10	$NiCl_2$.dppp	trace
11	$CoCl_2$	N.D.
12	$FeCl_3$	N.D.

[a] Reaction conditions: **1** (0.1 mmol), **2a** (0.2 mmol, 2.0 equiv), cat. (10 mol %), dtbpy (12 mol%), Zn (3.0 equiv), TMSCl (1.0 equiv) and KI (1.0 equiv) in DMA (0.5 mL) at 35 °C for 8 h under N_2; then TBAF (1.2 equiv), 0 °C, 0.5 h.

Table S2. Optimization of the Ligands

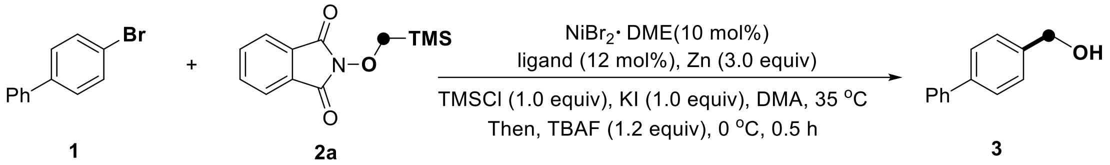

No.	Ligand	Yield (3) %
1	L1	68
2	L2	23
3	L3	50
4	L4	78
5	L5	31
6	L6	5
7	L7	42
8	L8	55
9	L9	48
10	L10	trace
11	L11	N.D.
12	L12	N.D.
13	L13	Trace
14	L14	Trace

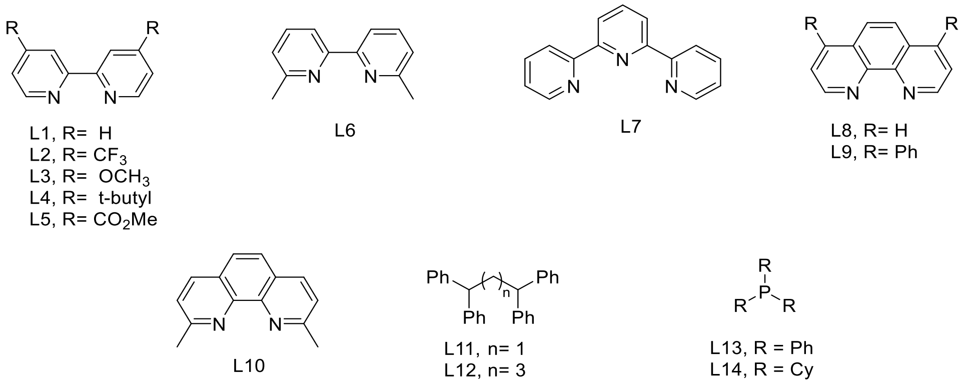

[a] Reaction conditions: **1** (0.1 mmol), **2a** (0.2 mmol, 2.0 equiv), $NiBr_2$.DME (10 mol %), ligand (12 mol%), Zn (3.0 equiv), TMSCl (1.0 equiv) and KI (1.0 equiv) in DMA (0.5 mL) at 35 °C for 8 h under N_2; then TBAF (1.2 equiv), 0 °C, 0.5 h.

Table S3. Optimization of Solvents

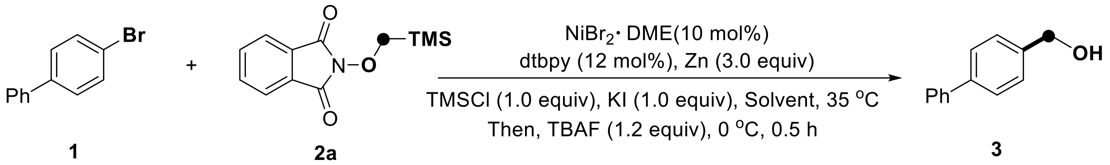

No.	Catalyst	Yield (3) %
1	DMSO	46
2	DMA	78
3	DMF	55
4	toulene	N.D.
5	1,4-dioxane	N.D.
6	NMP	trace
7	THF	46
8	DCE	trace
9	Acetone	71

[a] Reaction conditions: **1a** (0.1 mmol), **2a** (0.2 mmol, 2.0 equiv), $NiBr_2$.DME (10 mol %), dtbpy (12 mol%), Zn (3.0 equiv), TMSCl (1.0 equiv) and KI (1.0 equiv) in Solvent (0.5 mL) at 35 °C for 8 h under N_2; then TBAF (1.2 equiv), 0 °C, 0.5 h.

Table S4. Optimization of additives

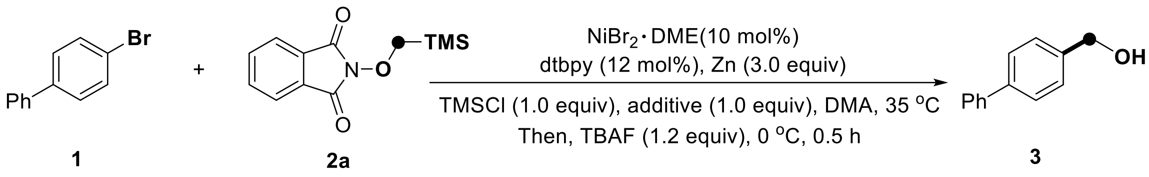

No.	Various conditions	Yield (3) %
1	NaI	38
2	LiCl	46
3	KI	78
4	TBAI	44
5	$MgBr_2$	27
6	$MgCl_2$	41
7	LiBr	63
8	KBr	60
9	$ZnCl_2$ instead of TMSCl	trace
10	TESCl instead of TMSCl	51
11	TIPSCl instead of TMSCl	55
12	$Fe(OTf)_2$ instead of TMSCl	trace
13	TMSCl (0.5 equiv)	30
14	TMSCl (2.0 equiv)	71

[a] Reaction conditions: **1** (0.1 mmol), **2a** (0.2 mmol, 2.0 equiv), $NiBr_2$.DME (10 mol %), dtbpy (12 mol%), Zn (3.0 equiv), TMSCl (1.0 equiv) and additive (1.0 equiv) in Solvent (0.5 mL) at 35 °C for 8 h under N_2; then TBAF (1.2 equiv), 0 °C, 0.5 h.

Table S5. Optimization of temperatures

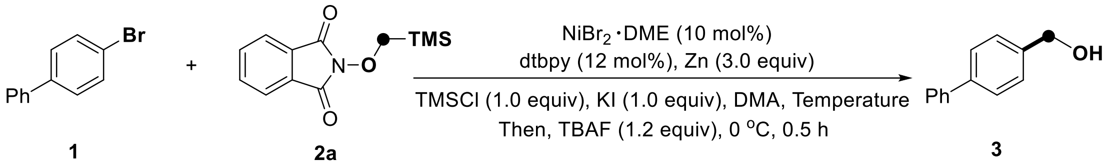

No.	Various conditions	Yield (3) %
1	RT	60
3	30 °C	72
2	35 °C	78
4	50 °C	61
5	60 °C	55
6	60 °C, with Cl instead of Br	20
7	80 °C, with Cl instead of Br	7
8	100 °C, with Cl instead of Br	trace

[a] Reaction conditions: **1** (0.1 mmol), **2a** (0.2 mmol, 2.0 equiv), $NiBr_2$.DME (10 mol %), dtbpy (12 mol%), Zn (3.0 equiv), TMSCl (1.0 equiv) and KI (1.0 equiv) in DMA (0.5 mL) at Temperature °C for 8 h under N_2; then TBAF (1.2 equiv), 0 °C, 0.5 h.

Table S6. Optimization of reductants

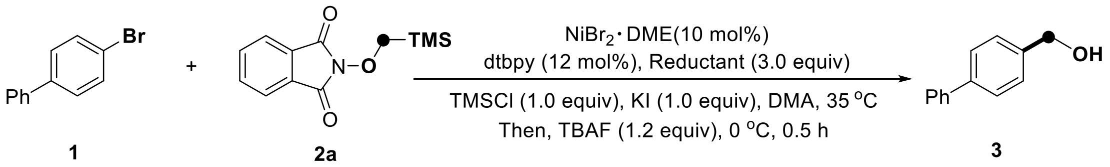

No.	Various conditions	Yield (3) %
1	Zn	78
2	Fe	N.D.
3	Mn	51

[a] Reaction conditions: **1** (0.1 mmol), **2a** (0.2 mmol, 2.0 equiv), $NiBr_2$.DME (10 mol %), dtbpy (12 mol%), Reductant (3.0 equiv), TMSCl (1.0 equiv) and KI (1.0 equiv) in DMA (0.5 mL) at 35 °C for 8 h under N_2; then TBAF (1.2 equiv), 0 °C, 0.5 h.

4. Characterization data

[1,1'-Biphenyl]-4-ylmethanol (3)[5]

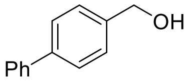

Following the **General Procedure 2.3., 3** was obtained in 78% yield (28.7 mg, 0.16 mmol) after flash column chromatography on silica gel (petroleum ether : ethyl acetate = 5 : 1); colorless liquid; **^{1}H NMR** (500 MHz, Chloroform-*d*) 7.63 (dd, *J* = 8.0, 4.3 Hz, 4H), 7.53 – 7.37 (m, 5H), 4.72 (s, 2H), 2.60 (s, 1H); **^{13}C NMR** (125 MHz, Chloroform-*d*) δ 140.9, 140.6, 140.0, 128.9, 127.5, 127.4, 127.3, 127.2, 64.9.

4-(Hydroxymethyl)benzonitrile (4)[6]

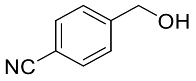

Following the **General Procedure 2.3.**, **4** was obtained in 68% (18.0 mg; X = Br) and 61% (X = I, 16.2 mg) yields while following **General Procedure 2.4.** 53 % (14.0 mg) yield; after flash column chromatography on silica gel (petroleum ether : ethyl acetate = 2 : 1); colorless liquid; **^{1}H NMR** (500 MHz, Chloroform-*d*) δ 7.63 (d, *J* = 8.4 Hz, 2H), 7.47 (d, *J* = 8.0 Hz, 2H), 4.76 (s, 2H), 2.61 (s, 1H); **^{13}C NMR** (125 MHz, Chloroform-*d*) δ 146.46, 132.30, 127.03, 118.92, 110.92, 64.08.

4-(Hydroxymethyl)benzaldehyde (5)[7]

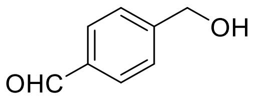

Following the **General Procedure 2.3.**, **5** was obtained in 64% (17.4 mg) yield after flash column chromatography on silica gel (petroleum ether : ethyl acetate = 2 : 1); white solid; **^{1}H NMR** (500 MHz, Chloroform-*d*) δ 9.93 (s, 6H), 7.79 (d, *J* = 8.7 Hz, 2H), 7.46 (d, *J* = 8.7 Hz, 2H), 4.73 (s, 2H), 2.14 (s, 1H); **^{13}C NMR** (125 MHz, Chloroform-*d*) δ 192.7, 148.4, 136.2, 130.6, 127.5, 65.1.

(4-(Methylsulfonyl)phenyl)methanol (6)[6]

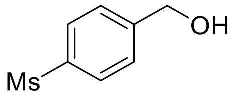

Following the **General Procedure 2.3.**, **6** was obtained in 72% yield (26.8 mg) after flash column chromatography on silica gel (petroleum ether : ethyl acetate = 1 : 1); yellow liquid; **^{1}H NMR** (500 MHz, Chloroform-*d*) δ 7.80 (d, *J* = 8.6 Hz, 2H), 7.49 (d, *J* = 8.5 Hz, 2H), 4.75 (s, 2H), 3.01 (s, 3H), 2.84 (s, 1H); **^{13}C NMR** (125 MHz, Chloroform-*d*) δ 147.2, 139.0, 127.4, 127.2, 63.9, 44.5.

Methyl 4-(hydroxymethyl)benzoate (7)[6]

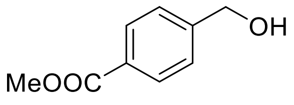

Following the **General Procedure 2.3.**, **7** was obtained in 65% (X = Br, 21.6 mg) and 54% (X = I, 17.9 mg) yields after flash column chromatography on silica gel (petroleum ether : ethyl acetate = 2 : 1);

white solid; ^{1}**H NMR** (500 MHz, Chloroform-*d*) δ 7.93 (d, *J* = 8.3 Hz, 2H), 7.35 (d, *J* = 8.3 Hz, 2H), 4.67 (s, 2H), 3.86 (s, 3H), 3.05 (s, 1H); ^{13}**C NMR** (125 MHz, Chloroform-*d*) δ 167.2, 146.3, 129.8, 129.0, 126.4, 64.4, 52.3.

(4-(Trifluoromethyl)phenyl)methanol (8)[7]

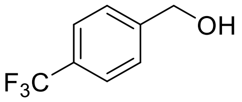

Following the **General Procedure 2.3.**, **8** was obtained in 65% (X = Br, 22.9 mg) and 53% (X = I, 18.6 mg) yields after flash column chromatography on silica gel (petroleum ether: ethyl acetate = 5: 1); colorless solid; ^{1}**H NMR** (500 MHz, Chloroform-*d*) δ 7.57 (d, *J* = 8.4 Hz, 2H), 7.40 (d, *J* = 8.3 Hz, 2H), 4.67 (s, 2H), 3.03 (s, 1H); ^{13}**C NMR** (125 MHz, Chloroform-*d*) δ 144.7, 129.73 (q, *J* = 32.6 Hz), 126.8, 125.4 (q, *J* = 3.8 Hz), 124.2 (q, *J* = 272.9 Hz), 63.7; ^{19}**F NMR** (471 MHz, Chloroform-d) δ -62.53.

(4-fluorophenyl)methanol (9)[6]

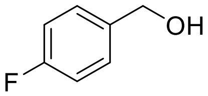

Following the **General Procedure 2.3.**, **9** was obtained in 78% (X = Br, 19.7 mg) and 65% (X = I, 18.6 mg) yields while following **General Procedure 2.4.** 64% (18.4 mg) yield; after flash column chromatography on silica gel (petroleum ether: ethyl acetate = 5 : 1); colorless liquid; ^{1}**H NMR** (500 MHz, Chloroform-*d*) δ 7.21 (d, *J* = 9.0 Hz, 2H), 6.94 (d, *J* = 9.0 Hz, 2H), 4.53 (s, 2H), 2.19 (s, 1H); ^{13}**C NMR** (125 MHz, Chloroform-*d*) δ 162.3 (d, *J* = 245.3 Hz), 136.6, 128.8 (d, *J* = 8.2 Hz), 115.4 (d, *J* = 21.4 Hz), 64.5; ^{19}**F NMR** (471 MHz, Chloroform-*d*) δ -114.94.

(4-Chlorophenyl)methanol (10)[6]

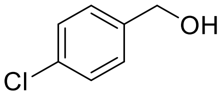

Following the **General Procedure 2.3.**, **10** was obtained in 76% (X = Br, 21.6 mg) and 55% (X = I, 15.6 mg) yields while following **General Procedure 2.4.** 66% (18.7 mg) yield; after flash column chromatography on silica gel (petroleum ether : ethyl acetate = 5 : 1); colorless liquid; ^{1}**H NMR** (500 MHz, Chloroform-d) δ 7.18 (d, *J* = 8.6 Hz, 2H), 7.10 (d, *J* = 8.5 Hz, 2H), 4.44 (s, 2H), 2.98 (s, 1H); ^{13}**C NMR** (125 MHz, Chloroform-d) δ 139.7, 133.7, 129.1, 128.7, 64.7.

(4-Ethynylphenyl)methanol (11)[8]

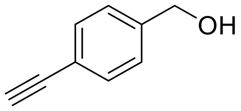

Following the **General Procedure 2.3.**, **11** was obtained in 68% yield (18.0 mg) after flash column chromatography on silica gel (petroleum ether : ethyl acetate = 5 : 1); white solid; ^{1}**H NMR** (500 MHz, Chloroform-*d*) δ 7.40 (d, *J* = 8.2 Hz, 2H), 7.22 (d, *J* = 8.3 Hz, 2H), 4.59 (s, 2H), 3.00 (s, 1H), 1.93 (s, 1H); ^{13}**C NMR** (125 MHz, Chloroform-*d*) δ 141.6, 132.3, 126.8, 121.3, 83.5, 64.8.

Phenylmethanol (12)[9]

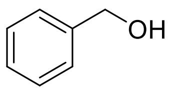

Following the **General Procedure 2.3.**, **12** was obtained in 75% (X = Br, 16.2 mg) and 71% (X = I, 15.3 mg) yields after flash column chromatography on silica gel (petroleum ether : ethyl acetate = 5 : 1); colorless liquid; **^{1}H NMR** (500 MHz, Chloroform-*d*) δ 7.39 (d, *J* = 7.6 Hz, 2H), 7.35 (d, *J* = 7.6 Hz, 2H), 7.29 (d, *J* = 7.6 Hz, 1H), , 4.60 (s, 2H), 4.39 (s, 2H), 2.61 (s, 1H); **^{13}C NMR** (125 MHz, Chloroform-*d*) δ 140.4, 134.9, 129.4, 129.0, 65.0.

(4-Methoxyphenyl)methanol (13)[7]

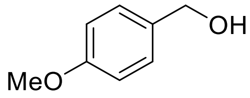

Following the **General Procedure 2.3.**, **13** was obtained in 51% (X = Br, 14.0 mg) and 66% (X = I, 18.2 mg) yields while following **General Procedure 2.4.** 61% (16.8 mg) yield; after flash column chromatography on silica gel (petroleum ether : ethyl acetate = 4 :1); white solid; **^{1}H NMR** (500 MHz, Chloroform-*d*) δ 7.21 (d, *J* = 8.8 Hz, 2H), 6.83 (d, *J* = 8.8 Hz, 2H), 4.51 (s, 2H), 3.75 (s, 3H), 2.95 (s, 1H); **^{13}C NMR** (125 MHz, Chloroform-*d*) δ 159.1, 133.8, 128.6, 113.5, 64.6, 55.7.

(4-(Benzyloxy)phenyl)methanol (14)[10]

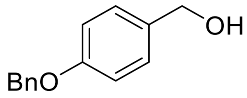

Following the **General Procedure 2.3.**, **14** was obtained in 65% yield (27.8 mg) after flash column chromatography on silica gel (petroleum ether : ethyl acetate = 4 : 1); white solid; **^{1}H NMR** (500 MHz, Chloroform-*d*) δ 7.29 (d, *J* = 7.6 Hz, 2H), 7.25 (d, *J* = 7.6 Hz, 2H), 7.19 (d, *J* = 7.6 Hz, 1H), 7.11 (d, *J* = 7.6 Hz, 2H), 6.82 (d, *J* = 7.6 Hz, 2H), 4.90 (s, 2H), 4.39 (s, 2H), 2.51 (s, 1H); **^{13}C NMR** (125 MHz, Chloroform-*d*) δ 158.7, 137.4, 133.9, 129.1, 129.0, 129.0, 128.4, 127.9, 115.3, 70.5, 65.2.

(4-(Dimethylamino)phenyl)methanol (15)[9]

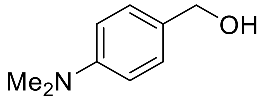

Following the **General Procedure 2.3.**, **15** was obtained in 58% yield (17.5 mg) after flash column chromatography on silica gel (petroleum ether : ethyl acetate = 3 : 1); white solid; **^{1}H NMR** (500 MHz, Chloroform-*d*) δ 7.09 (d, *J* = 8.7 Hz, 2H), 6.60 (d, *J* = 8.7 Hz, 2H), 4.38 (s, 2H), 2.80 (s, 6H), 2.54 (s, 1H); **^{13}C NMR** (125 MHz, Chloroform-*d*) δ 150.7, 129.6, 128.1, 113.2, 65.4, 41.4.

***N*-(4-(hydroxymethyl)phenyl)acetamide (16)**[11]

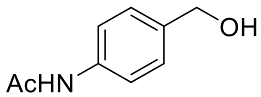

Following the **General Procedure 2.3.**, **16** was obtained in 64% yield (21.1 mg) after flash column chromatography on silica gel (petroleum ether : ethyl acetate = 1 : 2); yellow liquid; **^{1}H NMR** (500 MHz, DMSO-*d*$_6$) δ 9.85 (s, 1H), 7.49 (d, *J* = 6.8 Hz, 2H), 7.19 (d, *J* = 6.8 Hz, 2H), 5.05 (s, 1H), 4.39 (s, 2H), 1.98 (s, 3H); **^{13}C NMR** (125 MHz, DMSO-*d*$_6$) δ 173.4, 143.6, 142.3, 131.8, 124.3, 68.3, 29.2.

2,2,2-Trifluoro-*N*-(4-(hydroxymethyl)phenyl)acetamide (17)[10]

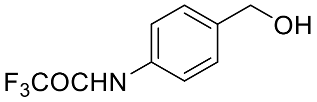

Following the **General Procedure 2.3.**, **17** was obtained in 61% yield (26.7 mg) after flash column chromatography on silica gel (petroleum ether : ethyl acetate = 1 : 2); yellow liquid; **^{1}H NMR** (500 MHz, DMSO-*d*$_6$) δ 11.09 (s, 1H), 7.48 (d, *J* = 8.6 Hz, 2H), 7.21 (d, *J* = 8.5 Hz, 2H), 5.09 (s, 1H), 4.34 (s, 2H); **^{13}C NMR** (125 MHz, DMSO-*d*$_6$) δ 154.85 (q, *J* = 36.3 Hz), 140.5, 135.2, 127.4, 121.8, 116.3 (q, *J* = 256.7 Hz), 62.9; **^{19}F NMR** (471 MHz, DMSO-*d*$_6$) δ -73.86.

4-(Hydroxymethyl)phenyl acetate (18)[12]

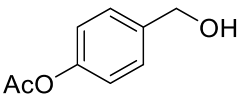

Following the **General Procedure 2.3.**, **18** was obtained in 62% yield (20.6 mg) after flash column chromatography on silica gel (petroleum ether : ethyl acetate = 3 : 1); colorless liquid; **^{1}H NMR** (500 MHz, Chloroform-*d*) δ 7.39 – 7.32 (m, 2H), 7.10 – 7.04 (m, 2H), 4.63 (s, 2H), 2.35 (s, 1H), 2.31 (s, 3H); **^{13}C NMR** (125 MHz, Chloroform-*d*) δ 169.74, 149.97, 138.63, 128.07, 121.62, 64.55, 21.13.

4-(hydroxymethyl)phenyl trifluoromethanesulfonate (19)

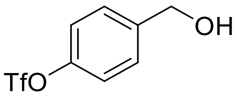

Following the **General Procedure 2.3.**, **19** was obtained in 64% yield (32.8 mg) after flash column chromatography on silica gel (petroleum ether : ethyl acetate = 5 : 1); yellow liquid; **^{1}H NMR** (500 MHz, Chloroform-*d*) δ 7.31 (d, *J* = 8.6 Hz, 2H), 7.15 (d, *J* = 8.5 Hz, 2H), 4.57 (s, 2H), 2.49 (s, 1H); **^{13}C NMR** (125 MHz, Chloroform-*d*) δ 148.8, 141.3, 128.5, 121.4, 118.8 (d, *J* = 272.9 Hz), 63.9; **^{19}F NMR** (471 MHz, Chloroform-*d*) δ -72.95; **HRMS(ESI)**: Found: m/z 257.0091. Calcd for $C_8H_8F_3O_4S$: $(M+H)^+$ 257.0090.

Dimethyl(phenyl)((4-(4,4,5,5-tetramethyl-1,3,2-dioxaborolan-2-yl)benzyl)oxy)silane (20)

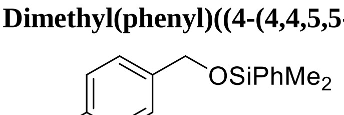

Following the **General Procedure 2.3.**, **20** was obtained in 65% yield (31.3 mg) after flash column chromatography on silica gel (petroleum ether : ethyl acetate = 10 : 1); colorless liquid; **^{1}H NMR** (500 MHz, Chloroform-*d*) δ 7.68 (d, *J* = 8.6 Hz, 2H), 7.51 (d, *J* = 8.6 Hz, 2H), 7.29 (d, *J* = 8.6 Hz, 2H), 7.22 (d, *J* = 8.5 Hz, 2H), 4.63 (s, 2H), 1.25 (s, 12H), 0.32 (s, 6H); **^{13}C NMR** (125 MHz, Chloroform-*d*) δ

145.7, 139.2, 136.5, 135.2, 131.4, 129.6, 127.4, 115.8, 85.5, 66.6, 25.2, 1.3; **HRMS(ESI)**: Found: m/z 369.2053. Calcd for $C_{21}H_{30}BO_3Si$: $(M+H)^+$ 369.2052.

Benzo[*d*][1,3]dioxol-5-ylmethanol (21)[10]

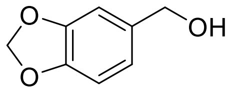

Following the **General Procedure 2.3.**, **21** was obtained in 68% yield (20.7 mg) after flash column chromatography on silica gel (petroleum ether : ethyl acetate = 2 : 1); colorless liquid; **^{1}H NMR** (500 MHz, Chloroform-*d*) δ 6.83 (s, 1H), 6.77 (d, *J* = 2.0 Hz, 2H), 5.93 (s, 2H), 4.53 (s, 2H), 2.15 (s, 1H); **^{13}C NMR** (125 MHz, Chloroform-*d*) δ 148.3, 147.6, 135.4, 121.0, 108.7, 108.4, 101.5, 65.7.

4-(Hydroxymethyl)phenol (22)[13]

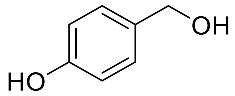

Following the **General Procedure 2.3.**, **22** was obtained in 54% yield (10.9 mg, 0.09 mmol) after flash column chromatography on silica gel (petroleum ether: ethyl acetate = 2 : 1); white solid; **^{1}H NMR** (500 MHz, Chloroform-*d*) δ 9.30 (s, 1H), 7.11 (d, *J* = 8.4 Hz, 2H), 6.72 (d, *J* = 8.3 Hz, 2H), 4.93 (s, 1H), 4.37 (s, 1H); **^{13}C NMR** (125 MHz, Chloroform-*d*) δ 159.7, 133.2, 128.5, 115.3, 63.3.

(4-Aminophenyl)methanol (23)[13]

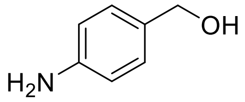

Following the **General Procedure 2.1**, **23** was obtained in 55% yield (13.3 mg) after flash column chromatography on silica gel (petroleum ether : ethyl acetate = 1 : 1); white solid; **^{1}H NMR** (500 MHz, Chloroform-*d*) δ 7.17 – 7.11 (m, 2H), 6.69 – 6.64 (m, 2H), 4.54 (s, 2H), 3.68 (s, 2H); **^{13}C NMR** (125 MHz, Chloroform-*d*) δ 146.1, 131.1, 128.8, 115.2, 65.3.

1,4-Phenylenedimethanol (24)[10]

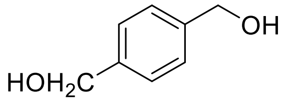

Following the **General Procedure 2.3.**, **24** was obtained in 71% yield (19.6 mg, 0.14 mmol) after flash column chromatography on silica gel (petroleum ether : ethyl acetate = 2 : 1); yellow liquid; **^{1}H NMR** (500 MHz, Chloroform-*d*) δ 7.49 (s, 4H), 4.70 (s, 4H); **^{13}C NMR** (125 MHz, Chloroform-*d*) δ 143.0, 127.3, 63.7.

(2-Aminophenyl)methanol (25)[11]

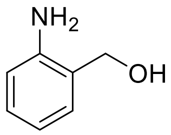

Following the **General Procedure 2.3.**, **25** was obtained in 58% yield (14.3 mg) after flash column chromatography on silica gel (petroleum ether : ethyl acetate = 1 : 1); yellow liquid; **^{1}H NMR** (500 MHz, Chloroform-*d*) δ 7.14 (t, *J* = 7.7 Hz, 1H), 7.06 (d, *J* = 7.4 Hz, 1H), 6.76 – 6.66 (m, 2H), 4.63 (s, 2H), 3.40 (s, 3H); **^{13}C NMR** (125 MHz, Chloroform-*d*) δ 146.0, 129.4, 129.2, 124.9, 118.2, 116.1, 64.7.

o-tolylmethanol (26)[10]

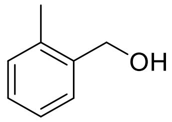

Following the **General Procedure 2.3.**, **26** was obtained in 74% yield (18.1 mg) after flash column chromatography on silica gel (petroleum ether: ethyl acetate = 5 : 1); colorless liquid; **^{1}H NMR** (500 MHz, Chloroform-*d*) δ 7.25 – 7.19 (m, 1H), 7.14 – 7.08 (m, 2H), 7.08 – 7.04 (m, 1H), 4.52 (s, 2H), 2.22 (s, 3H), 2.15 (s, 1H); **^{13}C NMR** (125 MHz, Chloroform-*d*) δ 139.2, 136.6, 130.8, 128.2, 128.0, 126.5, 63.8, 19.1.

(2-Methoxyphenyl)methanol (27)[9]

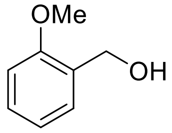

Following the **General Procedure 2.3.**, **27** was obtained in 73% (20.1 mg) while following **General Procedure 2.4.** 67% (18.5 mg) yield; after flash column chromatography on silica gel (petroleum ether : ethyl acetate = 5 :1); colorless oil; **^{1}H NMR** (500 MHz, Chloroform-*d*) δ 7.19 – 7.08 (m, 2H), 6.84 – 6.77 (m, 1H), 6.71 (d, *J* = 8.2 Hz, 1H), 4.52 (s, 2H), 3.67 (s, 3H), 2.89 (s, 1H); **^{13}C NMR** (125 MHz, Chloroform-*d*) δ 156.7, 129.3, 128.8, 128.5, 120.6, 110.2, 62.1, 54.5.

2-(hydroxymethyl)benzonitrile (28)[10]

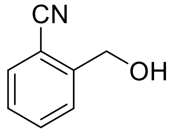

Following the **General Procedure 2.3.**, **28** was obtained in 58% yield (15.4 mg) after flash column chromatography on silica gel (petroleum ether : ethyl acetate = 3 :1); colorless liquid; **^{1}H NMR** (500 MHz, Chloroform-*d*) δ 7.90 – 7.82 (m, 1H), 7.66 (td, *J* = 7.4, 1.1 Hz, 1H), 7.50 (t, *J* = 7.1 Hz, 2H), 5.29 (s, 2H); **^{13}C NMR** (125 MHz, Chloroform-*d*) δ 171.12, 146.59, 134.05, 129.00, 125.62, 125.58, 122.22, 69.71.

(2-bromophenyl)methanol (29)[12]

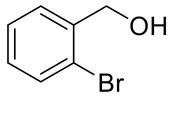

Following the **General Procedure 2.3.**, **29** was obtained in 52% yield (19.3 mg) after flash column chromatography on silica gel (petroleum ether: ethyl acetate = 5 : 1); colorless liquid; **^{1}H NMR** (500 MHz, Chloroform-*d*) δ 7.43 (d, *J* = 7.9 Hz, 1H), 7.35 (d, *J* = 7.7 Hz, 1H), 7.21 (t, *J* = 7.4 Hz, 1H), 7.05

(t, J = 7.8 Hz, 1H), 4.62 (s, 2H), 2.59 (s, 1H); **^{13}C NMR** (125 MHz, Chloroform-*d*) δ 139.7, 132.6, 129.1, 128.8, 127.7, 122.5, 64.9.

(3-Methoxyphenyl)methanol (30)[12]

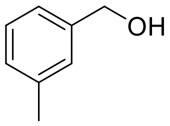

Following the **General Procedure 2.3.**, **30** was obtained in 71% yield (17.3 mg, 0.14 mmol) while following **General Procedure 2.4.** 66% (16.1 mg) yield after flash column chromatography on silica gel (petroleum ether : ethyl acetate = 5 : 1); colorless liquid; **^{1}H NMR** (500 MHz, Chloroform-*d*) δ 7.28 (t, J = 7.5 Hz, 1H), 7.23 – 7.10 (m, 3H), 4.66 (s, 2H), 2.40 (s, 3H).; **^{13}C NMR** (125 MHz, Chloroform-*d*) δ 140.9, 138.3, 128.5, 128.4, 127.8, 124.1, 65.3, 21.4.

(3-Methoxyphenyl)methanol (31)[12]

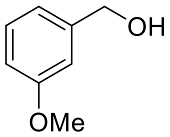

Following the **General Procedure 2.3.**, **31** was obtained in 68% yield (18.8 mg) after flash column chromatography on silica gel (petroleum ether: ethyl acetate = 5 : 1); colorless liquid; **^{1}H NMR** (500 MHz, Chloroform-*d*) δ 7.08 (t, J = 7.9 Hz, 1H), 6.73 (d, J = 6.9 Hz, 2H), 6.65 (d, J = 9.7 Hz, 1H), 4.38 (s, 2H), 3.58 (s, 3H), 3.48 (s, 1H); **^{13}C NMR** (125 MHz, Chloroform-*d*) δ 160.0, 143.0, 129.8, 119.5, 113.4, 112.6, 65.0, 55.5.

(3-(Trifluoromethyl)phenyl)methanol (32)[12]

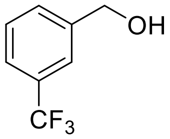

Following the **General Procedure 2.3.**, **32** was obtained in 59% yield (20.8 mg) after flash column chromatography on silica gel (petroleum ethe r: ethyl acetate = 5 : 1); yellow liquid; **^{1}H NMR** (500 MHz, Chloroform-*d*) δ 7.59 (s, 1H), 7.53 (d, J = 7.5 Hz, 1H), 7.49 – 7.41 (m, 2H), 4.66 (s, 2H), 2.97 (s, 1H); **^{13}C NMR** (125 MHz, Chloroform-*d*) δ 142.2, 131.35 (q, J = 32.6 Hz), 130.6, 129.5, 124.9, 124.0, 124.7 (q, J = 272.9 Hz), 64.8; **^{19}F NMR** (471 MHz, Chloroform-*d*) δ -62.68.

(3-Bromophenyl)methanol (33)[12]

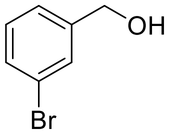

Following the **General Procedure 2.3.**, **33** was obtained in 61% yield (22.6 mg) after flash column chromatography on silica gel (petroleum ether : ethyl acetate = 5 : 1); colorless liquid; **^{1}H NMR** (500

MHz, Chloroform-*d*) δ 7.47 (s, 1H), 7.39 (dt, *J* = 7.4, 1.8 Hz, 1H), 7.24 – 7.18 (m, 2H), 4.58 (s, 2H), 2.71 (s, 1H); **^{13}C NMR** (125 MHz, Chloroform-*d*) δ 143.1, 130.6, 130.1, 129.9, 125.4, 122.6, 64.3.

2-(Hydroxymethyl)-6-methylphenol (34)[10]

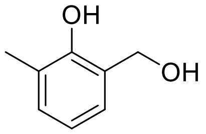

Following the **General Procedure 2.3.**, **34** was obtained in 55% yield (15.2 mg) after flash column chromatography on silica gel (petroleum ether : ethyl acetate = 5 : 1); colorless liquid; **^{1}H NMR** (500 MHz, Chloroform-*d*) δ 7.44 (s, 1H), 7.09 (d, *J* = 7.4 Hz, 1H), 6.87 (d, *J* = 7.4 Hz, 1H), 6.76 (t, *J* = 7.4 Hz, 1H), 4.85 (s, 2H), 2.26 (s, 3H); **^{13}C NMR** (125 MHz, Chloroform-*d*) δ 154.4, 130.9, 125.5, 125.4, 123.9, 119.6, 65.0, 15.6.

Benzofuran-2-ylmethanol (35)[10]

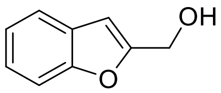

Following the **General Procedure 2.3.**, **35** was obtained in 68% yield (20.1 mg) after flash column chromatography on silica gel (petroleum ether: ethyl acetate = 4 : 1); yellow liquid; **^{1}H NMR** (500 MHz, Chloroform-*d*) δ 7.37 (d, *J* = 8.3 Hz, 1H), 7.30 (d, *J* = 7.0 Hz, 1H), 7.15 – 7.03 (m, 2H), 6.43 (d, *J* = 1.0 Hz, 1H), 4.55 (s, 2H), 3.11 (s, 1H); **^{13}C NMR** (125 MHz, Chloroform-*d*) δ 156.6, 155.1, 128.2, 124.4, 122.9, 121.2, 111.3, 104.1, 57.9.

Naphthalen-2-ylmethanol (36)[9]

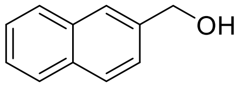

Following the **General Procedure 2.3.**, **36** was obtained in 64% yield (20.2 mg, 0.12 mmol) while following **General Procedure 2.4.** 59% (18.6 mg) yield; after flash column chromatography on silica gel (petroleum ether : ethyl acetate = 5 : 1); colorless liquid; **^{1}H NMR** (500 MHz, Chloroform-*d*) δ 7.89 – 7.79 (m, 3H), 7.76 (s, 1H), 7.55 – 7.48 (m, 2H), 7.45 (d, *J* = 8.4 Hz, 1H), 4.80 (s, 2H), 2.44 (s, 1H); **^{13}C NMR** (125 MHz, Chloroform-*d*) δ 138.4, 133.4, 133.0, 128.3, 128.0, 127.8, 126.2, 125.9, 125.5, 125.2, 65.4.

(9-Phenyl-9*H*-carbazol-3-yl)methanol (37)

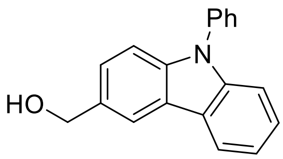

Following the **General Procedure 2.3.**, **37** was obtained in 66% yield (36.0 mg) after flash column chromatography on silica gel (petroleum ether : ethyl acetate = 3 : 1); yellow solid; **^{1}H NMR** (500 MHz, Chloroform-*d*) δ 8.00 (d, *J* = 7.8 Hz, 2H), 7.38 (d, *J* = 2.5 Hz, 4H), 7.24 (d, *J* = 3.8 Hz, 4H), 7.13 (dt, *J* = 8.0, 3.9 Hz, 2H), 4.60 (s, 2H), 2.27 (s, 1H); **^{13}C NMR** (125 MHz, Chloroform-*d*) δ 141.3, 140.6,

137.4, 128.9, 127.6, 123.9, 120.8, 120.5, 110.2, 65.2; **HRMS(ESI)**: Found: m/z 274.1226. Calcd for $C_{19}H_{16}NO$: $(M+H)^+$ 274.1225.

Pyridin-2-ylmethanol (38)[10]

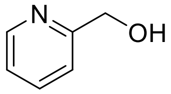

Following the **General Procedure 2.3.**, **38** was obtained in 48% yield (10.5 mg) after flash column chromatography on silica gel (petroleum ether : ethyl acetate = 3 : 1); yellow liquid; **^{1}H NMR** (500 MHz, Chloroform-*d*) δ 8.46 (d, *J* = 4.9 Hz, 1H), 7.60 (t, *J* = 7.7 Hz, 1H), 7.21 (d, *J* = 8.9 Hz, 1H), 7.12 (t, *J* = 6.3 Hz, 1H), 4.69 (s, 2H), 4.16 (s, 1H); **^{13}C NMR** (125 MHz, Chloroform-*d*) δ 159.4, 148.5, 136.8, 122.3, 120.7, 64.2.

(*E*)-3-phenylprop-2-en-1-ol (39)[9]

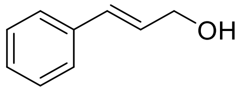

Following the **General Procedure 2.1**, **39** was obtained in 51% yield (13.7 mg) after flash column chromatography on silica gel (petroleum ether : ethyl acetate = 5 :1); colorless oil; **^{1}H NMR** (500 MHz, Chloroform-*d*) δ 7.29 (d, *J* = 7.0 Hz, 2H), t, *J* = 7.6 Hz, 2H), 7.16 (t, *J* = 7.2 Hz, 1H), 6.52 (d, *J* = 15.6 Hz, 1H), 6.27 (dt, *J* = 15.9, 5.7 Hz, 1H), 4.21 (s, 2H), 1.84 (s, 1H).; **^{13}C NMR** (125 MHz, Chloroform-*d*) δ 137.2, 131.6, 129.1, 129.1, 128.2, 127.0, 64.2.

(4-((((3a*S*,5a*R*,8a*R*,8b*S*)-2,2,7,7-tetramethyltetrahydro-3a*H*-bis([1,3]dioxolo)[4,5-b:4',5'-d]pyran-3a-yl)methoxy)methyl)phenyl)methanol (40)

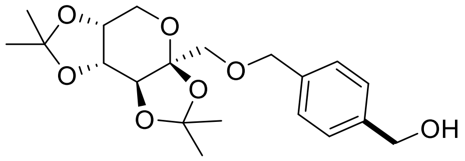

Following the **General Procedure 2.3.**, **40** was obtained in 55% yield (41.8 mg) after flash column chromatography on silica gel (petroleum ether : ethyl acetate = 1 : 1); yellow oil; **^{1}H NMR** (500 MHz, Chloroform-*d*) δ 7.33 (s, 4H), 4.68 (s, 2H), 4.62 – 4.57 (m, 2H), 4.43 (d, *J* = 2.6 Hz, 1H), 4.23 (d, *J* = 9.7 Hz, 1H), 3.91 (d, *J* = 13.0 Hz, 1H), 3.73 (d, *J* = 13.0 Hz, 1H), 3.61 (q, *J* = 10.6 Hz, 2H), 1.54 (s, 3H), 1.42 (d, *J* = 3.3 Hz, 6H), 1.33 (s, 3H); **^{13}C NMR** (125 MHz, Chloroform-*d*) δ 140.8, 138.2, 138.4, 127.6, 109.5, 109.2, 103.3, 74.0, 72.1, 71.6, 70.8, 70.7, 65.7, 61.6, 27.2, 26.4, 26.0, 24.6; **HRMS(ESI)**: Found: m/z 381.1910. Calcd for $C_{20}H_{29}O_7$: $(M+H)^+$381.1908.

(*S*)-4-(2-((tert-butoxycarbonyl)amino)-3-methoxy-3-oxopropyl)phenyl 4-(((dimethyl(phenyl)silyl)oxy)methyl)benzoate (41)

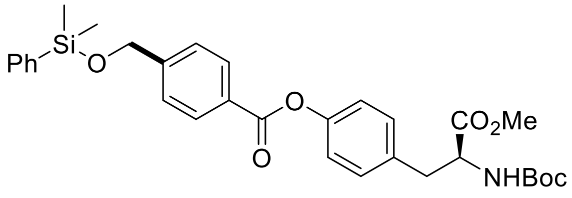

Following the **General Procedure 2.3.**, **41** was obtained in 41% yield (46.2 mg) after flash column chromatography on silica gel (petroleum ether : ethyl acetate = 5 :1); colorless oil; **^{1}H NMR** (500 MHz, Chloroform-*d*) δ 8.19 (d, J = 7.0 Hz, 1H), 8.14 (d, *J* = 8.4 Hz, 1H), 7.66 – 7.60 (m, 2H), 7.56 – 7.48 (m, 2H), 7.46 – 7.40 (m, 3H), 7.19 – 7.15 (m, 4H), 5.02 (d, *J* = 7.1 Hz, 1H), 4.78 (s, 1H), 4.61 (s, 1H), 3.73 (s, 3H), 3.11 (d, *J* = 11.1 Hz, 2H), 1.43 (s, 9H), 0.45 (s, 6H); **^{13}C NMR** (125 MHz, Chloroform-*d*) δ 172.8, 165.6, 155.7, 150.6, 147.5, 137.7, 134.1, 130.7, 130.4, 129.2, 128.6, 127.1, 126.8, 124.1, 122.3, 80.6, 65.0, 55.0, 52.9, 38.3, 28.9, -1.2; **HRMS(ESI)**: Found: m/z 564.2411. Calcd for $C_{31}H_{38}NO_7Si$: $(M+H)^+$ 564.2412.

(1*S*,2*S*,5*S*)-2-Isopropyl-5-methylcyclohexyl 4-(hydroxymethyl)benzoate (42)

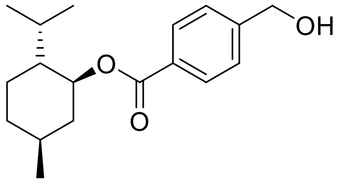

Following the **General Procedure 2.3.**, **42** was obtained in 66% yield (38.3 mg) after flash column chromatography on silica gel (petroleum ether : ethyl acetate = 3 : 1); colorless oil; **^{1}H NMR** (500 MHz, Chloroform-*d*) δ 7.87 (d, *J* = 8.3 Hz, 2H), 7.27 (d, *J* = 8.2 Hz, 2H), 4.79 (td, *J* = 10.9, 4.5 Hz, 1H), 4.57 (s, 2H), 3.67 (s, 1H), 1.98 (d, *J* = 12.7 Hz, 1H), 1.88 – 1.78 (m, 1H), 1.61 (d, J = 14.0 Hz, 2H), 1.49 – 1.37 (m, 2H), 0.98 (q, *J* = 10.7 Hz, 2H), 0.86 – 0.77 (m, 7H), 0.67 (s, 3H); **^{13}C NMR** (125 MHz, Chloroform-*d*) δ 165.7, 146.3, 129.7, 129.6, 126.4, 75.0, 64.2, 47.2, 40.9, 34.3, 31.4, 26.5, 23.7, 22.0, 20.7, 16.5; **HRMS(ESI)**: Found: m/z 291.1955. Calcd for $C_{18}H_{27}O_3$: $(M+H)^+$ 291.1955.

4-Allyl-2-methoxyphenyl 4-(((dimethyl(phenyl)silyl)oxy)methyl)benzoate (43)

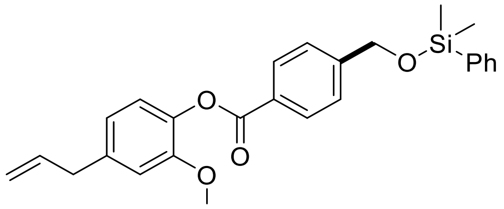

Following the **General Procedure 2.3.**, **43** was obtained in 65% yield (47.0 mg) after flash column chromatography on silica gel (petroleum ether: ethyl acetate = 10 : 1); colorless oil; **^{1}H NMR** (500 MHz, Chloroform-*d*) δ 8.18 (d, *J* = 8.1 Hz, 2H), 7.63 (d, *J* = 7.0 Hz, 2H), 7.43 (d, *J* = 20.7 Hz, 5H), 7.07 (d, *J* = 7.9 Hz, 1H), 6.86 – 6.80 (m, 2H), 6.08 – 5.92 (m, 1H), 5.17 – 5.10 (m, 2H), 4.79 (s, 2H), 3.81 (s, 3H), 3.42 (d, *J* = 6.6 Hz, 2H), 0.45 (s, 6H); **^{13}C NMR** (125 MHz, Chloroform-*d*) δ 166.6, 152.9, 148.4, 141.2, 140.0, 138.9, 135.3, 132.1, 131.6, 129.7, 127.9, 124.4, 122.5, 117.9, 114.6, 66.2, 58.2, 42.5, -1.2; **HRMS(ESI)**: Found: m/z 433.1829. Calcd for $C_{20}H_{24}O_8$: $(M+H)^+$ 433.1830.

***N*-(4-(hydroxymethyl)phenyl)-2-(6-methoxynaphthalen-2-yl)propanamide (44)**

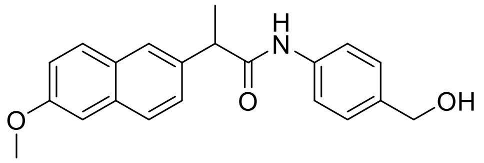

Following the **General Procedure 2.3.**, **44** was obtained in 62% yield (45.4 mg) after flash column chromatography on silica gel (petroleum ether : ethyl acetate = 1 : 1); colorless oil; **^{1}H NMR** (500 MHz, Chloroform-*d*) δ 10.10 (d, *J* = 2.6 Hz, 1H), 7.85 – 7.76 (m, 3H), 7.62 – 7.51 (m, 3H), 7.30 – 7.21 (m, 3H), 7.16 (dd, *J* = 8.9, 2.5 Hz, 1H), 5.15 -5.08 (m, 1H), 4.45 (d, *J* = 5.3 Hz, 2H), 3.98 (q, *J* = 7.1 Hz, 1H), 3.86 (s, 3H), 1.52 (dd, *J* = 7.1, 2.2 Hz, 3H); **^{13}C NMR** (125 MHz, Chloroform-*d*) δ 172.7, 157.5, 138.3, 137.8, 137.56, 133.7, 129.6, 128.9, 127.5, 127.3, 126.8, 125.9, 119.5, 119.2, 106.2, 63.1, 55.6, 46.4, 19.2; **HRMS(ESI)**: Found: m/z 336.1594. Calcd for $C_{21}H_{22}NO_3$: $(M+H)^+$ 336.1594.

(3*S*,8*S*,9*S*,10*R*,13*R*,14*S*,17*R*)-10,13-Dimethyl-17-((*R*)-6-methylheptan-2-yl)-2,3,4,7,8,9,10,11,12,13,14,15,16,17-tetradecahydro-1*H*-cyclopenta[a]phenanthren-3-yl 4-(hydroxymethyl)benzoate (45)

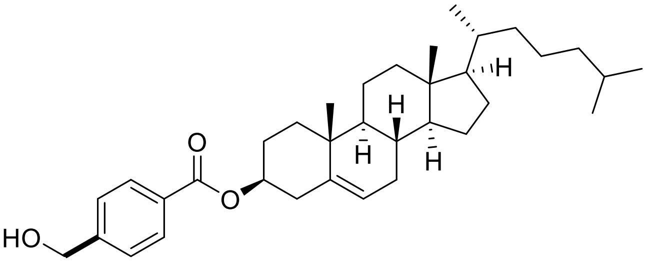

Following the **General Procedure 2.3.**, **45** was obtained in 51% yield (53.0 mg, 0.10 mmol) after flash column chromatography on silica gel (petroleum ether : ethyl acetate = 4 : 1); colorless oil; **^{1}H NMR** (500 MHz, Chloroform-*d*) δ 8.01 (d, *J* = 4.7 Hz, 2H), 7.40 (d, *J* = 7.9 Hz, 2H), 5.41 (s, 1H), 4.84 (s, 1H), 4.75 (s, 2H), 2.45 (d, *J* = 8.3 Hz, 2H), 1.77 – 1.02 (m, 33H), 0.87 – 0.83 (m, 6H), 0.68 (d, *J* = 3.2 Hz, 3H); **^{13}C NMR** (125 MHz, Chloroform-*d*) δ 165.9, 145.9, 139.6, 130.0, 129.8, 126.4, 122.8, 74.6, 64.7, 56.7, 56.2, 50.1, 42.3, 39.8, 39.5, 38.2, 37.0, 36.7, 36.2, 35.8, 33.9, 32.0, 28.3, 28.0, 27.9, 24.3, 23.9, 22.9, 22.6, 21.1, 19.4, 18.7, 11.9; **HRMS(ESI)**: Found: m/z 521.3990. Calcd for $C_{35}H_{53}O_3$: $(M+H)^+$ 521.3989.

***N*-(4-(hydroxymethyl)phenyl)-2-(4-isobutylphenyl)propanamide (46)**

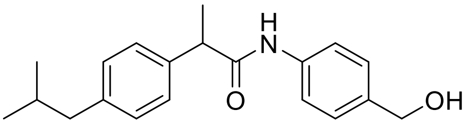

Following the **General Procedure 2.3.**, **46** was obtained in 73% yield (52.1 mg) after flash column chromatography on silica gel (petroleum ether : ethyl acetate = 1 : 1); colorless oil; **^{1}H NMR** (500 MHz, Chloroform-*d*) δ 7.33 (d, J = 8.4 Hz, 2H), 7.21 – 7.14 (m, 4H), 7.06 (d, *J* = 7.8 Hz, 2H), 4.51 (s, 2H), 3.70 – 3.60 (m, 1H), 2.39 (d, *J* = 7.2 Hz, 2H), 1.81 – 1.74 (m, 1H), 1.49 (d, J = 7.1 Hz, 3H), 0.83 (d, J = 6.6 Hz, 6H); **^{13}C NMR** (125 MHz, Chloroform-*d*) δ 173.5, 141.0, 138.6, 137.4, 136.4, 129.8, 127.7, 127.5,119.9, 64.8, 47.6, 45.0, 30.2, 22.4, 18.6; **HRMS(ESI)**: Found: m/z 312.1958. Calcd for $C_{20}H_{26}NO_2$: $(M+H)^+$ 312.1958.

4-(*N*,*N*-dipropylsulfamoyl)-*N*-(4-(hydroxymethyl)phenyl)benzamide (47)

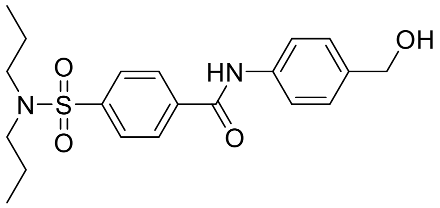

Following the **General Procedure 2.3.**, **47** was obtained in 63% yield (52.1 mg) after flash column chromatography on silica gel (petroleum ether: ethyl acetate = 1 : 2); White solid; ^{1}**H NMR** (500 MHz, DMSO-d_6) δ 10.46 (s, 1H), 8.17 – 8.11 (m, 2H), 7.99 – 7.92 (m, 2H), 7.77 – 7.71 (m, 2H), 7.36 – 7.29 (m, 2H), 5.16 (s, 1H), 4.49 (d, J = 5.2 Hz, 2H), 3.22 – 2.86 (m, 4H), 1.49 (h, J = 7.4 Hz, 4H), 0.83 (t, J = 7.4 Hz, 6H); ^{13}**C NMR** (125 MHz, DMSO-d_6) δ 165.1, 142.4, 139.0, 138.7, 137.9, 129.1, 127.3, 120.7, 63.1, 50.1, 21.7, 11.1; **HRMS(ESI)**: Found: m/z 391.1670. Calcd for $C_{20}H_{26}N_2O_4S$: $(M+H)^+$ 391.1673.

5. Gram scale reaction and downstream modificatins

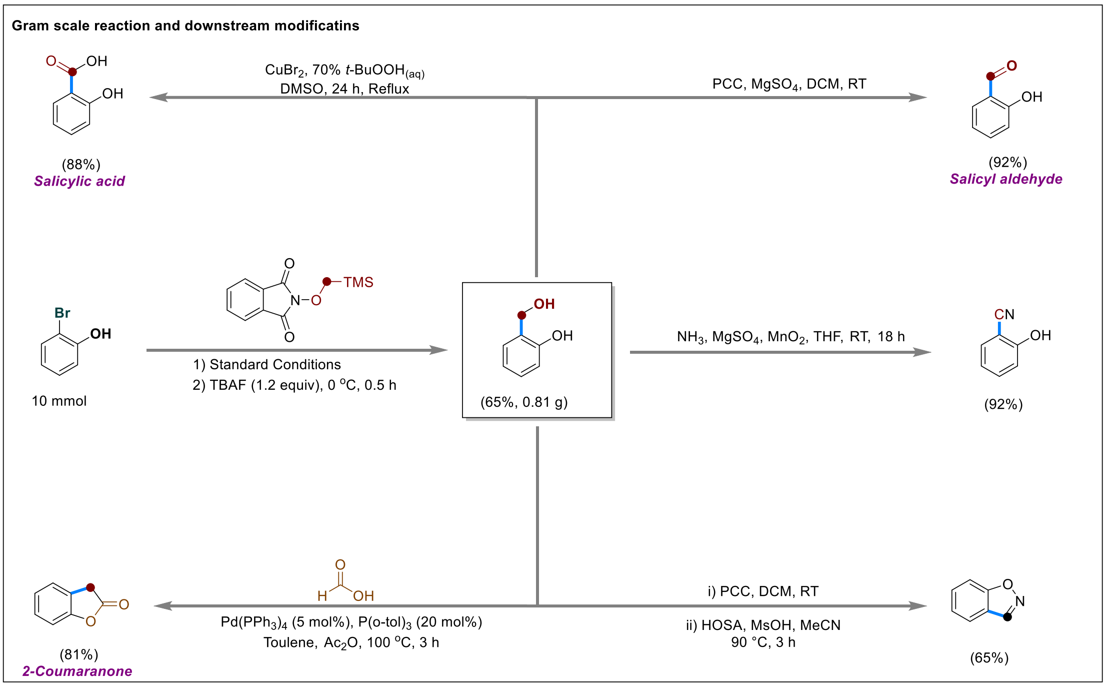

5.1. Gram scale synthesis

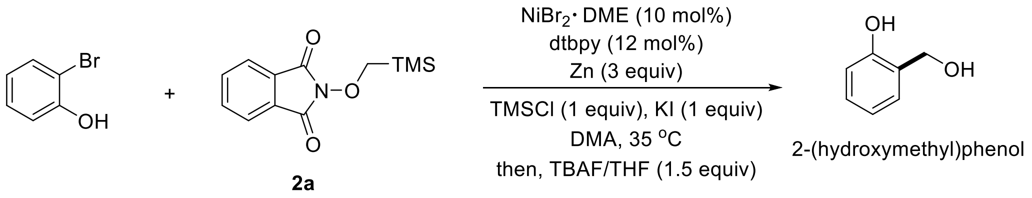

Gram-Scale Synthesis of 2-(hydroxymethyl)phenol. To a 100 mL of Schlenk tube were added 2-bromophenol (10.0 mmol, 1.0 equiv), **2a** (20.0 mmol), $NiBr_2{\cdot}DME$ (10 mol%), dtbpy (12 mol%), and zinc powder (30.0 mmol, 3.0 equiv.) under N_2 atmosphere. DMA (50 mL) was added and the tube stirred at 35 °C for 8 hours. After the indicated time, the Schlenk was opened to air and

TBAF/THF (1.5 equiv) was added and stirred for 15 min. Then, transfer to the separation funnel, add 10 mL of water, extract the water phase with ethyl acetate (30 mL × 3), combine the organic layer, wash the organic phase with saturated sodium chloride for three times, dry on anhydrous Na_2SO_4, and remove the solvent under pressure. The residue was purified with silica gel chromatography to afford product **3a** (0.81 g, 65%) after flash column chromatography on silica gel (petroleum ether : ethyl acetate = 1 : 1); colorless liquid; **^{1}H NMR** (500 MHz, Chloroform-*d*) δ 7.38 (s, 1H), 7.23 (t, *J* = 7.7 Hz, 1H), 7.06 (d, *J* = 7.4 Hz, 1H), 6.94 – 6.85 (m, 2H), 4.87 (s, 2H), 2.47 – 2.43 (m, 1H); **^{13}C NMR** (125 MHz, $CDCl_3$) δ 156.07, 129.54, 127.87, 124.66, 120.11, 116.55, 64.65.

5.2. Selective oxidation to carboxylic acid

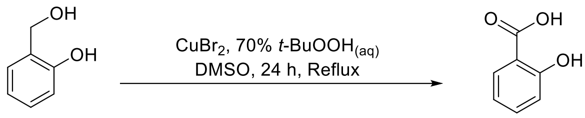

Under a nitrogen atmosphere, $CuBr_2$ (5 mol%) was added to a stirred solution of 2-(hydroxymethyl)phenol (1.0 equiv, 0.2 mmol) in MeCN (0.5 mL), followed by the addition of 70% *tert*-BuOOH in water (5 equiv). The reaction progress was monitored by TLC until complete consumption of the aldehyde. After completion, the volatiles were removed under reduced pressure, and the resulting residue was treated with saturated $NaHCO_3$ solution and extracted with ethyl acetate. The aqueous layer was then acidified with 2 M HCl and extracted with ethyl acetate. The combined organic extracts were concentrated under vacuum, and the crude product was purified by column chromatography using ethyl acetate/Petroleum ether as the eluent yielded salicylic acid (24.3 mg, 88%) as a white solid; **^{1}H NMR** (400 MHz, DMSO) δ 7.80 (dd, *J* = 7.8, 1.8 Hz, 1H), 7.51 (ddd, *J* = 8.6, 7.3, 1.8 Hz, 1H), 6.99 – 6.87 (m, 2H); **^{13}C NMR** (100 MHz, DMSO-d^6) δ 172.4, 161.6, 136.1, 130.7, 119.6, 117.6, 113.3.[14]

5.3. Selective oxidation to aldehyde

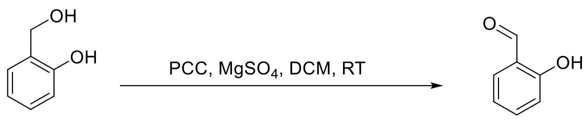

2-(hydroxymethyl)phenol (1.0 equiv, 0.2 mmol) was dissolved in dichloromethane, $MgSO_4$ (6.0 equiv, 1.2 mmol) after homogenization join PCC (3.0 equiv, 0.6 mmol) to the solution. The reaction mixture was stirred at room temperature overnight, effecting selective oxidation to the corresponding salicylaldehyde. The progress of the reaction was monitored by TLC. Upon completion, the mixture was diluted with petroleum ether and filtered. The filtrate was washed with water, and the product was purified by column chromatography to afford salicylaldehyde with yield (22.4 mg, 92%) as a yellow oil. **^{1}H NMR** (400 MHz, $CDCl_3$) δ 11.04 (s, 1H), 9.90 (s, 1H), 7.55 (ddd, *J* = 16.3, 7.7, 1.7 Hz, 2H), 7.07 – 6.97 (m, 2H); **^{13}C NMR** (100 MHz, $CDCl_3$) δ 196.6, 161.6, 137.0, 133.8, 120.7, 119.9, 117.6.[15]

5.4. Synthesis of cyanophenol

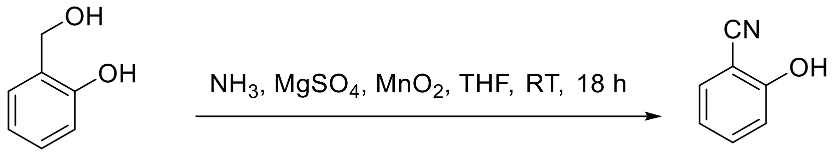

The reaction was performed by stirring the compound 48 (0.2 mmol, 1.0 equiv) with manganese dioxide (MnO_2, 3.0 equiv, 0.6 mmol), magnesium sulfate ($MgSO_4$, 3.0 equiv, 0.6 mmol), and ammonia (3.0 equiv, 0.6 mmol) in tetrahydrofuran (THF) at room temperature for 18 h. Isolated by column chromatography yielded cyanophenol (16.61 mg, 55%) as a white solid; ^{1}H NMR (400 MHz, DMSO) δ 11.04 (s, 1H), 7.59 (dd, J = 7.8, 1.7 Hz, 1H), 7.49 (ddd, J = 8.4, 7.4, 1.7 Hz, 1H), 7.02 (dd, J = 8.4, 1.0 Hz, 1H), 6.92 (td, J = 7.6, 1.0 Hz, 1H); ^{13}C NMR (100 MHz, DMSO) δ 160.6, 135.2, 133.7, 120.0, 117.5, 116.6, 99.3.[16]

5.5. Synthesis of 2-coumaranone

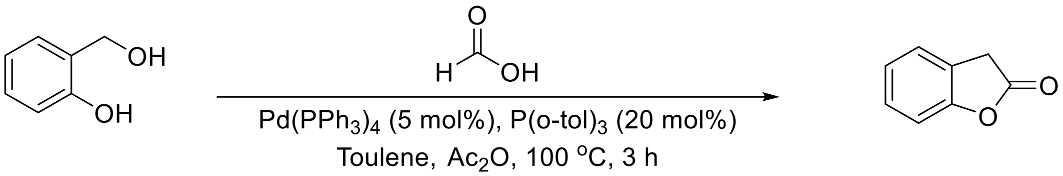

$Pd(PPh_3)_4$ (5 mol%) and P(*o*-tolyl)$_3$ (20 mol%) were charged into an oven-dried reaction tube under a nitrogen atmosphere. Toluene (0.5 mL) and compound 48 (1.0 equiv, 0.2 mmol) were then added, followed by a mixture of formic acid (3.0 equiv, 0.6 mmol) and acetic anhydride (3.0 equiv, 0.6 mmol). The reaction mixture was stirred at 30 °C for 1.5 h and subsequently heated to 100 °C for 2 h. After completion, the mixture was filtered and concentrated under reduced pressure. The crude product was purified by silica gel column chromatography the product afforded 2-coumaranone with yield (21.7 mg, 81%) as a white solid. **^{1}H NMR** (400 MHz, $CDCl_3$) δ 7.36 – 7.26 (m, 1H), 7.19 – 7.07 (m, 1H), 3.74 (s, 1H); **^{13}C NMR** (100 MHz, $CDCl_3$) δ 174.1, 154.7, 128.9, 124.7, 124.1, 123.1, 110.7, 33.0.[17]

5.6. Cylization

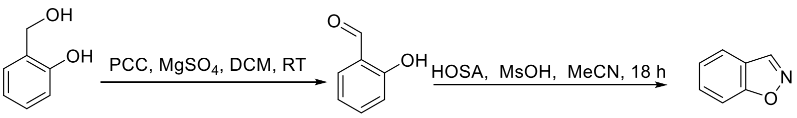

Salicylaldehyde (1.0 equiv) was dissolved in a solvent mixture solvent (MeCN/H_2O = 10/1) (0.5 mL) the join HOSA (1.5 equiv) at 0 °C, and the reaction mixture was stirred for 1 h. $NaHCO_3$ (2.5 equiv) was then added, and the mixture was stirred at room temperature for an additional 1 h; After completion, the mixture was diluted with ethyl acetate and wash with water on the Na_2SO_4 and concentrated under reduced pressure. The crude product was purified by silica gel column chromatography the product afforded benzo[d]isoxazole with yield (15.47 mg, 65%) as a yellow

oil; **^{1}H NMR** (400 MHz, $CDCl_3$) δ 8.74 (d, J = 1.1 Hz, 1H), 7.76 (dt, J = 7.9, 1.0 Hz, 1H), 7.64 (dt, J = 8.5, 1.1 Hz, 1H), 7.59 (ddd, J = 8.4, 6.9, 1.2 Hz, 1H), 7.35 (ddd, J = 7.9, 7.0, 1.0 Hz, 1H); **^{13}C NMR** (100 MHz, $CDCl_3$) δ 162.2, 146.2, 130.1, 123.8, 122.0, 121.3, 109.8.[15][18]

5.7. Synthesis of Adapalene

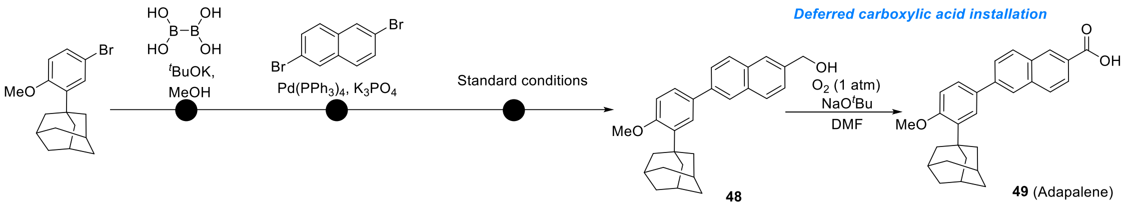

The commercially available 1-(5-bromo-2-methoxyphenyl)adamantane was first converted to the corresponding boronic acid, followed by Suzuki coupling and subsequent transformation according to our method (General Procedure 2.1). Final oxidation was carried out according to the reported literature procedure, affording adapalene as a white solid after purification by column chromatography (86% yield). **^{1}H NMR** (400 MHz, DMSO) δ 8.54 (d, J = 1.6 Hz, 1H), 8.17 – 8.12 (m, 1H), 8.09 (d, J = 8.6 Hz, 1H), 8.01 (d, J = 8.7 Hz, 1H), 7.92 (dd, J = 8.5, 1.7 Hz, 1H), 7.82 (dd, J = 8.6, 1.9 Hz, 1H), 7.58 (dd, J = 8.5, 2.3 Hz, 1H), 7.51 (d, J = 2.4 Hz, 1H), 7.05 (d, J = 8.6 Hz, 1H), 3.80 (s, 3H), 2.54 – 2.34 (m, 2H), 2.07 (d, J = 2.9 Hz, 6H), 2.03 – 1.97 (m, 2H), 1.69 (t, J = 2.9 Hz, 6H); **^{13}C NMR** (100 MHz, DMSO) δ 168.0, 159.1, 140.7, 139.1, 138.5, 135.9, 132.0, 130.7, 130.3, 128.8, 128.1, 126.4, 126.2, 126.0, 125.6, 124.5, 113.2, 55.8, 40.5, 37.09, 37.03, 28.9.[19-22]

6. Crystallographic data

A suitable crystal was selected on a Rigaku XtaLAB Synergy diffractometer. The structure was solved by direct methods using SHELXT and refined by full-matrix least-squares methods against F^2 by SHELXL using Olex2. The crystal structure has been deposited at The Cambridge Crystallographic Data Centre (CCDC: 2428438).

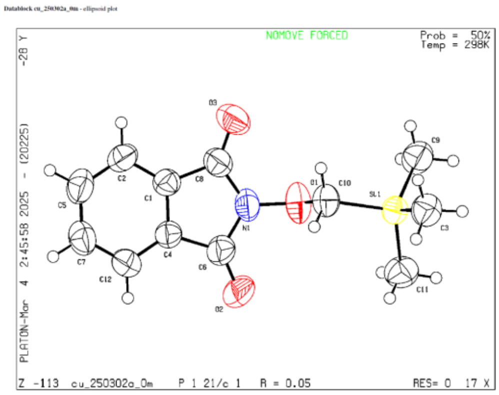

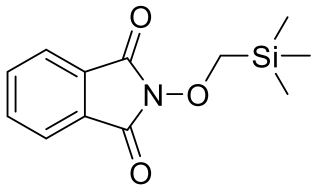

2a

Table S7. Crystallographic data

Compound	2a
Empirical formula	$C_{12}H_{15}NO_3Si$
Formula weight	249.34
CCDC No.	2428438
Temperature/K	298.0
Crystal system	monoclinic
Space group	$P2_1/c$
a/Å	6.0357(15)
b/Å	19.028(5)
c/Å	11.731(3)
α/°	90
β/°	92.745(12)
γ/°	90
Volume/Å ^{3}	1345.8(5)
Z	4
ρcalcg/cm ^{3}	1.231
μ/mm $^{-1}$	1.529
F(000)	528
Crystal size/mm ^{3}	0.032 × 0.025 × 0.014
Radiation	Cu Kα (λ = 1.54178 Å)
2Θ range for data collection/°	8.86 – 133.12
Index ranges	$-6 \le h \le 7$, $-22 \le k \le 16$, $-13 \le l \le 13$
Reflections collected	7778
Independent reflections	2344 [R_{int} = 0.043, R_{sigma} = 0.037]
Data/restr./para.	2344 / 0 / 157
Goodness-of-fit on F2	1.083
Final R indexes [I>=2σ (I)]	R_1 = 0.0499, wR_2 = 0.1347
Final R indexes [all data]	R_1 = 0.0563, wR_2 = 0.1396
Largest diff. peak/hole/e Å $^{-3}$	0.220 / −0.249

7. Computational investigations

7.1. Computational Methods.

All of the DFT calculations were carried out with the Gaussian 16 series of programs [23]. The B3LYP-D3 functional [24] with D3 version of Grimme's dispersion correctionst[25] with a Becke-Johnson (BJ) damping function [26] and the standard 6-31G(d) basis set (SDD basis set[27] for Ni and Zn atoms) was used for geometry optimizations. Harmonic vibrational frequency calculations were performed for all stationary points to determine whether they are local minima or transition structures and to derive thermochemical corrections for the enthalpies and free energies. The M06[28] functional with the 6-311+G(d,P) basis set (SDD basis set for Ni and Zn atoms) was used

to calculate the single-point energies and give more accurate energy information. The solvent effects were considered by single-point calculations of the gas-phase stationary points with the SMD solvation model[29] in n,n-dimethylacetamide solvent. The energies reported in this paper are the M06 calculated Gibbs free energies in n,n-dimethylacetamide solvent. The 3D diagrams of molecules were generated using CYLView [30].

7.2. Thermodynamic stability comparison between the singlet and triplet of int-3.

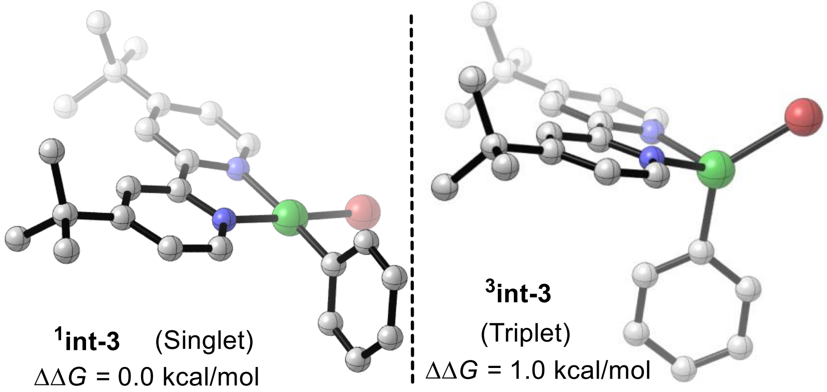

Fig. S1. Thermodynamic stability comparison for singlet and triplet states of Ni(II) species of **int-3**. The energy values are given in kcal /mol and represent the relative free energies calculated at B3LYP-D3(BJ)//6-311+G (d,p)-SDD-SMD(DMA)//B3LYP-D3(BJ)/6-31G(d)-SDD level of theory.

7.3. Thermodynamic stability comparison for different conformations of the intermediate int-4 and transition state int-4.

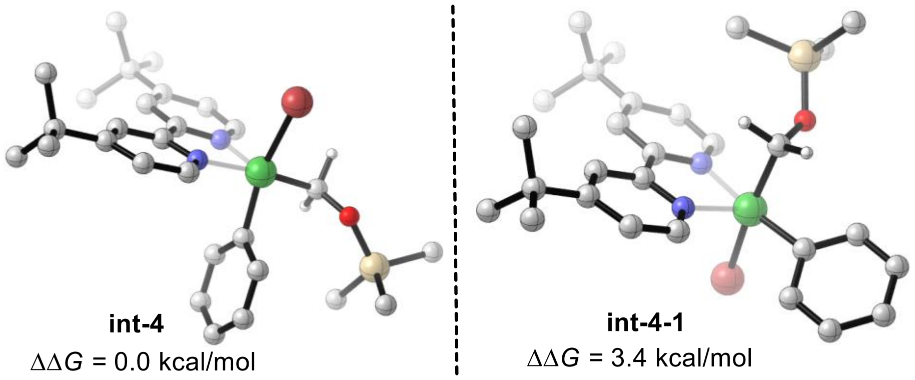

Fig. S2. Thermodynamic stability comparison for different conformations of the intermediate **int-4**. The energy values are given in kcal /mol and represent the relative free energies calculated at B3LYP-D3(BJ)//6-311+G (d,p)-SDD-SMD(DMA)//B3LYP-D3(BJ)/6-31G(d)-SDD level of theory.

7.4. Thermodynamic stability comparison for different conformations of the transition state TS-2.

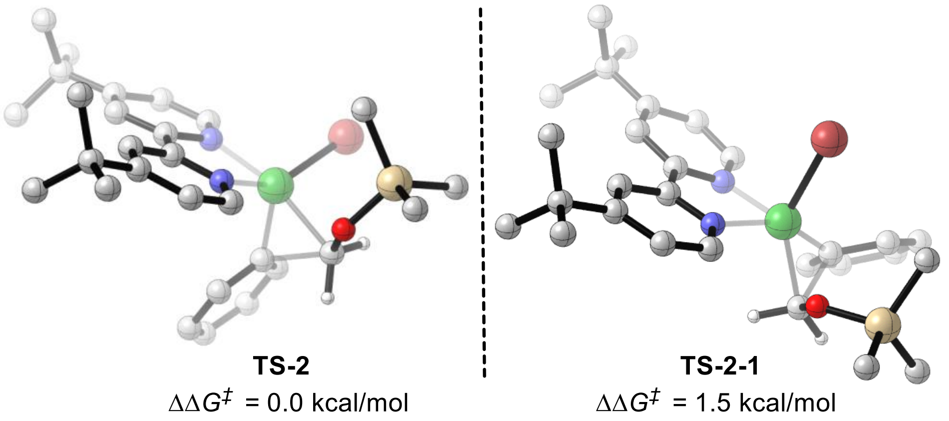

Fig. S3. Thermodynamic stability comparison for different conformations of the transition state **TS-2**. The energy values are given in kcal /mol and represent the relative free energies calculated at B3LYP-D3(BJ)//6-311+G (d,p)-SDD-SMD(DMA)//B3LYP-D3(BJ)/6-31G(d)-SDD level of theory.

7.5. Absolute energies and Cartesian coordinates for DFT-optimized compounds and transition states. Values are given in Hartree

Cat-1

B3LYP-D3(BJ)/6-31G(d)·SCF energy: -3552.460621 a.u.

B3LYP-D3(BJ)/6-31G(d)·Thermal correction to enthalpy: 0.412250

B3LYP-D3(BJ)/6-31G(d)·Thermal correction to Gibbs free energy: 0.331150

M06/6-311+G(d,p)·SCF energy in solution: -3554.593213 a.u.

Ni	2.34920600	0.00062000	-0.00044600
C	-0.39763600	-0.73869200	-0.00006800
C	-1.54557000	-1.52630400	-0.00020200
C	-1.45772800	-2.92474800	0.00009300
C	-0.16738400	-3.46886400	0.00051800
C	0.94103300	-2.62823900	0.00060500
N	0.84189600	-1.29213200	0.00030600
H	-2.51808300	-1.04897600	-0.00062200
H	-0.00665600	-4.53929700	0.00078400
H	1.95198500	-3.02333600	0.00093700
C	0.93955100	2.62883400	-0.00202700
C	-0.16933800	3.46883800	-0.00146300

C	-1.45937500	2.92399100	-0.00026300
C	-1.54642700	1.52549200	0.00023400
C	-0.39805000	0.73853500	-0.00047500
N	0.84117500	1.29268000	-0.00153000
H	1.95028300	3.02449600	-0.00289000
H	-0.00920100	4.53936100	-0.00193700
H	-2.51867400	1.04762700	0.00128400
C	-2.73293800	3.77342700	0.00055900
C	-2.73081800	-3.77487700	-0.00012600
C	-3.55862300	-3.44423600	1.26152000
H	-3.84869200	-2.38899300	1.29352800
H	-4.47581000	-4.04372100	1.27658500
H	-2.99017800	-3.66502800	2.17136700
C	-3.55773200	-3.44484000	-1.26252100
H	-2.98863700	-3.66604800	-2.17185900
H	-4.47490100	-4.04434500	-1.27796000
H	-3.84778400	-2.38961500	-1.29522400
C	-2.42012000	-5.28043300	0.00034700
H	-1.85225700	-5.57803900	-0.88791600
H	-1.85278900	-5.57759900	0.88909600
H	-3.35657700	-5.84771300	0.00020200
C	-3.55901600	3.44307600	1.26342100
H	-4.47651800	4.04206000	1.27924400
H	-3.84845000	2.38768900	1.29640000
H	-2.98959200	3.66471600	2.17244800
C	-3.56125300	3.44219600	-1.26061000
H	-3.85081800	2.38680500	-1.29230800
H	-4.47874200	4.04122700	-1.27525900
H	-2.99341600	3.66313400	-2.17080100
C	-2.42299800	5.27912300	-0.00024700
H	-1.85474000	5.57707700	0.88764400
H	-1.85633800	5.57645400	-0.88936900
H	-3.35972900	5.84594100	0.00039800
Br	4.62324500	0.00117800	0.00061500

Phenyl bromide

B3LYP-D3(BJ)/6-31G(d)·SCF energy: -2803.377214 a.u.

B3LYP-D3(BJ)/6-31G(d)·Thermal correction to enthalpy: 0.097540

B3LYP-D3(BJ)/6-31G(d)·Thermal correction to Gibbs free energy: 0.060048
M06/6-311+G(d,p)·SCF energy in solution: -2805.541146 a.u.

C	-2.87907500	0.00004900	-0.00005600
C	-2.17923300	1.20755000	0.00008500
C	-0.78383900	1.21565300	-0.00001800
C	-0.10383200	-0.00010900	-0.00040600
C	-0.78393400	-1.21573000	-0.00003900
C	-2.17942200	-1.20742200	0.00008500
H	-3.96486400	0.00019000	0.00011300
H	-2.71687900	2.15138700	0.00014200
H	-0.23088800	2.14822100	0.00011800
H	-0.23122100	-2.14843600	0.00000400
H	-2.71703100	-2.15128900	0.00032800
Br	1.80905400	-0.00000100	0.00003900

int-1

B3LYP-D3(BJ)/6-31G(d)·SCF energy: -6355.559522 a.u.
B3LYP-D3(BJ)/6-31G(d)·Thermal correction to enthalpy: 0.511993
B3LYP-D3(BJ)/6-31G(d)·Thermal correction to Gibbs free energy: 0.412136
M06/6-311+G(d,p)·SCF energy in solution: -6360.147573 a.u.

Ni	1.55002900	-0.12012700	-1.90347800
C	-0.87307300	1.03849100	-1.01010500
C	-1.75688100	1.98274400	-0.49629200
C	-1.42297800	3.34239100	-0.47502600
C	-0.17749000	3.68919700	-1.01411900
C	0.65758600	2.69862600	-1.52110600
N	0.33126800	1.39973300	-1.51748300
H	-2.70257700	1.65461700	-0.08305100
H	0.15962200	4.71754000	-1.04091600
H	1.63326900	2.93590000	-1.93372500
C	-0.20010600	-2.49189400	-1.46268500
C	-1.32822100	-3.14732000	-0.98363100
C	-2.40704900	-2.40815300	-0.48445800
C	-2.27469700	-1.01510800	-0.51457600
C	-1.11569000	-0.41782800	-1.00136400
N	-0.08244800	-1.15755300	-1.47359300

H	0.65580500	-3.04272600	-1.83934300
H	-1.34029400	-4.22902100	-0.98756700
H	-3.07696900	-0.39144800	-0.14033000
C	-0.19420800	0.64581700	2.35504500
C	1.01248900	0.94510700	1.72053700
C	1.84522700	-0.08553000	1.28140000
C	1.47925700	-1.41879800	1.48368700
C	0.27523500	-1.69693400	2.12378300
C	-0.57396200	-0.68147600	2.56244900
H	-0.84871700	1.44575600	2.68947100
H	2.77316700	0.13058300	0.76243600
H	2.11784300	-2.22089400	1.13279000
H	-1.50681900	-0.92461700	3.05783700
Br	-0.22287300	-3.52363600	2.42518900
Br	3.80304900	-0.37410100	-2.16517900
H	1.29420900	1.97920600	1.54768800
C	-2.38772600	4.35859900	0.14073500
C	-3.68116200	-3.04838100	0.07124600
C	-1.85018400	5.79578800	0.04933800
H	-2.57194300	6.48678900	0.49693000
H	-0.90374800	5.91068000	0.58888700
H	-1.69499200	6.10494400	-0.99009600
C	-3.74131900	4.29237200	-0.59964600
H	-4.19631900	3.29908200	-0.53046200
H	-4.44340100	5.01230800	-0.16428900
H	-3.61823800	4.53325000	-1.66100600
C	-2.59460600	4.00554400	1.63094900
H	-1.64482200	4.04376500	2.17601500
H	-3.28421700	4.71880500	2.09630100
H	-3.01536500	3.00239800	1.75587100
C	-4.86693300	-2.66057200	-0.83953900
H	-5.79440200	-3.10504000	-0.46079100
H	-5.00625200	-1.57496100	-0.88038000
H	-4.70848200	-3.01822800	-1.86277700
C	-3.93748200	-2.53056500	1.50386900
H	-4.85364800	-2.97971200	1.90352700
H	-3.10598200	-2.79611200	2.16332600
H	-4.06312800	-1.44316000	1.53094900

C	-3.57515100	-4.58088500	0.12595700
H	-3.44659900	-5.01650600	-0.87096000
H	-2.73798600	-4.89896400	0.75612800
H	-4.49399900	-4.99738200	0.55159300

TS-1

B3LYP-D3(BJ)/6-31G(d)·SCF energy: -6355.535371 a.u.

B3LYP-D3(BJ)/6-31G(d)·Thermal correction to enthalpy: 0.510045

B3LYP-D3(BJ)/6-31G(d)·Thermal correction to Gibbs free energy: 0.413191

M06/6-311+G(d,p)·SCF energy in solution: -6360.123768 a.u.

Imaginary frequency: -134.57 cm^{-1}

Ni	1.64135000	-0.53273900	-0.48203500
C	-0.96180000	0.76158200	-0.42283000
C	-1.90618600	1.76925100	-0.24981100
C	-1.52647300	3.11771100	-0.32077400
C	-0.17978500	3.37944200	-0.58975100
C	0.71410000	2.32244800	-0.75191500
N	0.33349300	1.04921600	-0.65965400
H	-2.93606400	1.51007100	-0.03661000
H	0.19313600	4.39246100	-0.66786100
H	1.76720200	2.47410500	-0.96565100
C	-0.38042900	-2.82242200	-0.26339300
C	-1.64690600	-3.39811900	-0.20191100
C	-2.78260300	-2.58193600	-0.21403100
C	-2.56001400	-1.20003700	-0.28927100
C	-1.26697800	-0.68669800	-0.34562600
N	-0.18663400	-1.50258100	-0.33402200
H	0.51983700	-3.42713000	-0.24044800
H	-1.72584600	-4.47611700	-0.14606900
H	-3.40227100	-0.51981500	-0.31633800
C	0.58739600	0.97318400	3.04403900
C	1.43575100	2.08589500	2.99770000
C	2.61662400	2.02302900	2.25133600
C	2.94243200	0.87908300	1.52398400
C	2.02611700	-0.18638600	1.49875600
C	0.89235200	-0.18138000	2.32916900
H	-0.31649300	1.00190600	3.64762300

H	3.29582600	2.87144800	2.23309200
H	3.84353100	0.82780500	0.92534000
H	0.24029000	-1.04637300	2.36199400
Br	2.86268400	-2.13293700	1.06581000
Br	3.29990000	0.14177600	-2.06451400
H	1.19278200	2.98109900	3.56241900
C	-4.21175900	-3.12648800	-0.15412700
C	-4.96977400	-2.68977700	-1.42740600
H	-5.99672800	-3.07088500	-1.40068100
H	-5.01980400	-1.60000700	-1.51894700
H	-4.48251300	-3.08133400	-2.32672500
C	-4.24249500	-4.66095100	-0.06779200
H	-3.73480000	-5.02752800	0.83109100
H	-5.28118300	-5.00421300	-0.02472800
H	-3.77604700	-5.12634800	-0.94285500
C	-4.91779000	-2.54969700	1.09299700
H	-4.39363200	-2.84097300	2.00968600
H	-4.96640500	-1.45631800	1.06501300
H	-5.94429300	-2.92839700	1.15086500
C	-2.56603100	4.21707400	-0.08777900
C	-3.70644200	4.06834300	-1.11839700
H	-4.20734800	3.09827200	-1.03632800
H	-4.46025000	4.84748200	-0.95892400
H	-3.32505400	4.16520300	-2.14057900
C	-3.13370700	4.06616400	1.34129100
H	-2.33703900	4.15255600	2.08807300
H	-3.87468700	4.84953900	1.53663200
H	-3.62429600	3.09774900	1.48379500
C	-1.96175900	5.62356900	-0.22765400
H	-1.16637600	5.79820500	0.50495900
H	-1.55163600	5.79105300	-1.22948900
H	-2.73969700	6.37499000	-0.05737500

int-2

B3LYP-D3(BJ)/6-31G(d)·SCF energy: -6355.547323 a.u.

B3LYP-D3(BJ)/6-31G(d)·Thermal correction to enthalpy: 0.511741

B3LYP-D3(BJ)/6-31G(d)·Thermal correction to Gibbs free energy: 0.413875

M06/6-311+G(d,p)·SCF energy in solution: -6360.132935 a.u.

Ni	1.35479700	-0.33729200	-0.42816100
C	-1.20409200	0.89227300	-0.20049700
C	-2.17845500	1.88488500	-0.14751000
C	-1.82759500	3.23996100	-0.20081600
C	-0.46306500	3.53105700	-0.30503200
C	0.46380800	2.49580000	-0.35183300
N	0.10309400	1.21415900	-0.30205700
H	-3.22023000	1.60110400	-0.06733000
H	-0.10417700	4.55066700	-0.35335400
H	1.52712800	2.68933900	-0.43512100
C	-0.53680800	-2.67164600	-0.21786200
C	-1.78736100	-3.28179500	-0.15361300
C	-2.94502800	-2.49861300	-0.09293900
C	-2.76859200	-1.10731400	-0.10124500
C	-1.49180300	-0.55798000	-0.16784800
N	-0.39924000	-1.34600600	-0.21984100
H	0.38951500	-3.23448400	-0.27011900
H	-1.83737900	-4.36278300	-0.15600100
H	-3.63180400	-0.45470500	-0.06274300
C	1.18385900	-0.05455700	3.79343400
C	2.19786000	0.86905500	4.05047800
C	2.99193400	1.33359800	3.00042300
C	2.76968900	0.87940400	1.69644300
C	1.73919200	-0.02715200	1.44910700
C	0.95123000	-0.50791200	2.49009800
H	0.56835300	-0.42968500	4.60763600
H	3.79710600	2.03834100	3.19257600
H	3.41675500	1.21754400	0.89025000
H	0.16456700	-1.23146900	2.30389700
Br	2.97928900	-2.01819200	-0.27365300
Br	1.28620400	-0.09725700	-2.82923600
H	2.37642100	1.21599300	5.06435800
C	-4.35572400	-3.08915200	-0.02508300
C	-2.91444800	4.31597000	-0.14687400
C	-5.04209400	-2.60471400	1.27130300
H	-5.12358900	-1.51345900	1.30716500
H	-6.05502700	-3.01742100	1.33486400

H	-4.48395000	-2.93069900	2.15566600
C	-4.33948900	-4.62626200	-0.02535400
H	-5.36638000	-5.00239300	0.02481100
H	-3.88410100	-5.02723600	-0.93723900
H	-3.79727400	-5.02733700	0.83792000
C	-5.16134200	-2.60566100	-1.25147100
H	-4.68654300	-2.92873200	-2.18390300
H	-6.17466500	-3.02126000	-1.22026700
H	-5.24839300	-1.51466900	-1.27878200
C	-3.69760400	4.17359200	1.17707100
H	-4.48164500	4.93696600	1.23144200
H	-4.17904800	3.19406700	1.26325500
H	-3.03514600	4.30087300	2.04002400
C	-2.32657900	5.73453300	-0.21816000
H	-1.77804900	5.90027400	-1.15171800
H	-3.13763700	6.46860000	-0.17627500
H	-1.65249300	5.93779400	0.62118300
C	-3.87357600	4.12004100	-1.34208000
H	-3.33709800	4.20541800	-2.29294200
H	-4.36104800	3.14007500	-1.31777600
H	-4.65819200	4.88448700	-1.32048000

$Zn(DMA)_2$

B3LYP-D3(BJ)/6-31G(d)·SCF energy: -802.871411 a.u.

B3LYP-D3(BJ)/6-31G(d)·Thermal correction to enthalpy: 0.284770

B3LYP-D3(BJ)/6-31G(d)·Thermal correction to Gibbs free energy: 0.209622

M06/6-311+G(d,p)·SCF energy in solution: -802.658803 a.u.

O	-0.30773600	-1.09920800	1.14414400
C	0.45168600	-0.23675400	-0.96356400
H	1.32129600	-0.85544200	-0.73604600
H	0.04591700	-0.53030400	-1.93800900
H	0.77474000	0.80873800	-1.03681600
O	3.06328000	-2.07740300	-0.04465900
C	1.44966000	-3.72915500	0.59819700
H	1.63434900	-4.74485000	0.96192600
H	0.54328900	-3.33447800	1.06715100
H	1.29329600	-3.75940700	-0.48086200

C	2.61666700	-2.79101200	0.85505100
C	-0.56648900	-0.40823500	0.14750900
N	3.14067000	-2.76141300	2.12321500
N	-1.76973700	0.22914800	0.02897300
C	2.66678000	-3.56902100	3.23226700
H	3.33035000	-4.42577900	3.42280300
H	2.63436400	-2.95263400	4.13821000
H	1.65869300	-3.93611100	3.04644000
C	4.33078900	-1.96753200	2.38480300
H	5.16738700	-2.61606600	2.67917900
H	4.58701600	-1.42795500	1.47470700
H	4.13929100	-1.25221300	3.19414200
C	-2.76096100	0.10014100	1.08684000
H	-2.40482100	-0.63198300	1.80882000
H	-2.91533800	1.06320400	1.59072700
H	-3.71897100	-0.22766600	0.66433400
C	-2.14128500	1.11080100	-1.06313800
H	-1.35409400	1.16387100	-1.81196200
H	-3.05905600	0.75166000	-1.54717200
H	-2.33145400	2.12539200	-0.68776000
Zn	1.23007100	-0.16043000	3.11085000

$ZnBr_2(DMA)_2$

B3LYP-D3(BJ)/6-31G(d)·SCF energy: -5945.853353 a.u.

B3LYP-D3(BJ)/6-31G(d)·Thermal correction to enthalpy: 0.291774

B3LYP-D3(BJ)/6-31G(d)·Thermal correction to Gibbs free energy: 0.212597

M06/6-311+G(d,p)·SCF energy in solution: -5950.845224 a.u.

O	-1.39492000	0.45408600	-1.51697600
C	-3.43247700	1.59978600	-1.02626800
H	-2.86389900	2.42937800	-1.44681400
H	-4.41348000	1.54599100	-1.50629400
H	-3.57335700	1.78158500	0.04589500
O	0.87913700	-1.46728200	-0.55842600
C	2.70088900	-1.41905800	-2.10060700
H	3.01389600	-0.39462800	-2.33495700
H	3.55427900	-2.08979000	-2.23163900
H	1.90310300	-1.70133800	-2.78788000

C	2.13065800	-1.43418800	-0.70387300
C	-2.61073700	0.34619400	-1.20000600
N	2.95468700	-1.39358000	0.35478300
N	-3.17280800	-0.85563800	-1.00214400
C	4.35811000	-1.00826100	0.24982800
H	4.45079900	0.07633800	0.38560000
H	4.92925000	-1.52112400	1.02858500
H	4.77088400	-1.28076900	-0.71981700
C	2.39164300	-1.31019700	1.70347300
H	3.05648000	-1.83877700	2.39259300
H	2.30490200	-0.26157100	2.00918100
H	1.40253800	-1.76341600	1.71865900
C	-2.34367500	-2.05968000	-1.06687800
H	-2.00005400	-2.32620800	-0.06156200
H	-2.94254700	-2.87472200	-1.48400800
H	-1.47456900	-1.87094800	-1.69241100
C	-4.45799100	-1.03001800	-0.33477700
H	-5.10111200	-0.16471100	-0.48374100
H	-4.95851300	-1.91159800	-0.74520700
H	-4.29526700	-1.17345400	0.74058000
Zn	-0.01948600	0.33513600	0.00392500
Br	-1.38113800	-0.06237600	1.90558400
Br	1.76558400	1.85752800	-0.24134000

[1]int-3

B3LYP-D3(BJ)/6-31G(d)·SCF energy: -3784.114640 a.u.

B3LYP-D3(BJ)/6-31G(d)·Thermal correction to enthalpy: 0.509109

B3LYP-D3(BJ)/6-31G(d)·Thermal correction to Gibbs free energy: 0.418256

M06/6-311+G(d,p)·SCF energy in solution: -3786.094864 a.u.

Ni	1.40175300	0.11712400	1.23954400
C	-1.18253900	1.18886700	0.58765700
C	-2.22554000	2.08131400	0.35661400
C	-2.03050300	3.46142500	0.50519300
C	-0.75096400	3.87168500	0.89068000
C	0.25080000	2.92827200	1.10662300
N	0.04644400	1.61544500	0.96043100
H	-3.19646000	1.70500800	0.05955700

H	-0.51168800	4.91826800	1.02879400
H	1.25771500	3.20180500	1.40670900
C	-0.19325500	-2.30334700	0.63000600
C	-1.33706900	-3.00422100	0.26125800
C	-2.52152100	-2.32112500	-0.02592700
C	-2.47019200	-0.92566600	0.08265900
C	-1.29817000	-0.27604600	0.45607100
N	-0.15670200	-0.96526600	0.73031700
H	0.73208500	-2.81592200	0.85556300
H	-1.27845400	-4.08355900	0.20505600
H	-3.35490400	-0.33769200	-0.12664300
C	3.25841600	-3.28467700	2.79057800
C	3.92981400	-3.82021900	1.68942100
C	3.90917200	-3.13193700	0.47452500
C	3.21284800	-1.92441500	0.36051400
C	2.51659000	-1.38459500	1.45294500
C	2.56314000	-2.07692200	2.67283200
H	3.27917500	-3.80491500	3.74597000
H	4.44031600	-3.53238700	-0.38650800
H	3.21567400	-1.39839200	-0.59176300
H	2.05349700	-1.67119500	3.54415000
Br	3.16498300	1.48150700	1.82277200
H	4.47179100	-4.75813200	1.77984700
C	-3.82086900	-3.01501000	-0.43884500
C	-3.18648600	4.43134900	0.24876100
C	-4.23899700	-2.50874400	-1.83717500
H	-5.17034600	-2.99491000	-2.14832400
H	-4.40775200	-1.42700200	-1.84565700
H	-3.46886700	-2.73569500	-2.58217800
C	-3.66476900	-4.54313500	-0.49724700
H	-3.38950900	-4.96055300	0.47739200
H	-4.61527200	-4.99710400	-0.79572500
H	-2.90827200	-4.84615000	-1.22923000
C	-4.92310300	-2.67136800	0.58754400
H	-5.86254100	-3.15985900	0.30574200
H	-4.64573500	-3.01513400	1.58981000
H	-5.10971700	-1.59382400	0.64043500
C	-4.34451700	4.10124000	1.21643200

H	-5.18194500	4.78711300	1.04627900
H	-4.71501200	3.08051500	1.07630100
H	-4.02459200	4.20358800	2.25894000
C	-3.66745500	4.26700100	-1.21000300
H	-4.49654300	4.95470800	-1.41084000
H	-2.85986200	4.48907900	-1.91577900
H	-4.02063600	3.25033600	-1.41110200
C	-2.77057400	5.89534900	0.46489900
H	-2.44060600	6.07587200	1.49366200
H	-1.96398200	6.19268000	-0.21395100
H	-3.62561600	6.55113800	0.27108500

^{3}int-3

B3LYP-D3(BJ)/6-31G(d)·SCF energy: -3784.111143 a.u.

B3LYP-D3(BJ)/6-31G(d)·Thermal correction to enthalpy: 0.508239

B3LYP-D3(BJ)/6-31G(d)·Thermal correction to Gibbs free energy: 0.411582

M06/6-311+G(d,p)·SCF energy in solution: -3786.086544 a.u.

Ni	1.69194000	-0.32461200	-0.71285900
C	-0.91938400	0.79877800	-0.19601200
C	-1.90500400	1.69733600	0.20631300
C	-1.58033100	3.02424900	0.51770600
C	-0.23319200	3.38568100	0.40124900
C	0.70391500	2.43940100	-0.00234900
N	0.37348700	1.18027200	-0.29483700
H	-2.93265100	1.36438500	0.28197400
H	0.10305000	4.39046100	0.62128400
H	1.75677800	2.68542200	-0.09696500
C	-0.21135700	-2.61995400	-1.19233400
C	-1.43888700	-3.27506200	-1.16911900
C	-2.59437200	-2.57377200	-0.80570400
C	-2.43361300	-1.21938200	-0.48499200
C	-1.17638900	-0.62106700	-0.53096100
N	-0.08009000	-1.32859000	-0.88362600
H	0.70400900	-3.13577500	-1.46442800
H	-1.47428600	-4.32395900	-1.43301400
H	-3.29500400	-0.63064500	-0.19520200
C	1.59686200	-1.28825900	3.47523400

C	2.93018300	-1.58916700	3.76251800
C	3.88279000	-1.51839600	2.74492800
C	3.50072800	-1.14896900	1.45057800
C	2.16519700	-0.84041700	1.13256700
C	1.22976400	-0.92067600	2.17795700
H	0.84613700	-1.34048500	4.26188800
H	4.92420800	-1.75085900	2.95817600
H	4.26190300	-1.10092200	0.67320800
H	0.18033200	-0.69169700	1.98616900
Br	3.00502900	-0.10888800	-2.64769300
H	3.22288100	-1.87588500	4.76974400
C	-3.98280600	-3.21569400	-0.74509500
C	-2.67575400	3.99665500	0.96257700
C	-4.92361700	-2.47433200	-1.72060400
H	-5.92319400	-2.92192500	-1.68907200
H	-5.02426500	-1.41457600	-1.46473500
H	-4.55185700	-2.53900100	-2.74882700
C	-3.94922200	-4.70284600	-1.13280700
H	-3.31331700	-5.28459900	-0.45669900
H	-4.96041400	-5.11843500	-1.07545000
H	-3.58936600	-4.84763400	-2.15729800
C	-4.52863400	-3.09514800	0.69511100
H	-5.52343700	-3.55013200	0.75758900
H	-3.87223600	-3.60717700	1.40678700
H	-4.61859600	-2.05079000	1.01117300
C	-3.33860400	3.45200500	2.24734700
H	-4.12646100	4.13652600	2.58087200
H	-3.79542300	2.47019400	2.08606000
H	-2.60531700	3.35383600	3.05492500
C	-3.73262900	4.11164100	-0.15821100
H	-4.52548000	4.80444700	0.14493700
H	-3.28344400	4.48897200	-1.08313600
H	-4.19947600	3.14639700	-0.37968200
C	-2.11914200	5.39919000	1.25615200
H	-1.37927000	5.38167900	2.06375400
H	-1.65462500	5.84566100	0.37019300
H	-2.93537000	6.05810700	1.56940900

III

B3LYP-D3(BJ)/6-31G(d)·SCF energy: -523.797795 a.u.

B3LYP-D3(BJ)/6-31G(d)·Thermal correction to enthalpy: 0.151608

B3LYP-D3(BJ)/6-31G(d)·Thermal correction to Gibbs free energy: 0.104487

M06/6-311+G(d,p)·SCF energy in solution: -523.663339 a.u.

C	2.89920500	-0.85874500	1.18254300
H	3.24362300	-1.85789200	0.91874300
H	2.37945200	-0.70159500	2.12204400
O	3.62912100	0.20876500	0.75661400
Si	4.41843200	0.27477600	-0.74048800
C	5.48351900	-1.26206800	-0.95857200
H	6.06554900	-1.19466500	-1.88583100
H	6.19020400	-1.37588200	-0.12869700
H	4.88237200	-2.17658200	-1.01641000
C	5.46887000	1.82419700	-0.65489500
H	6.00195000	1.99047700	-1.59860000
H	4.84906400	2.70623300	-0.45946300
H	6.21389300	1.75373700	0.14520500
C	3.13198600	0.38449200	-2.10599000
H	3.60279000	0.40481200	-3.09634900
H	2.45316600	-0.47527600	-2.07360100
H	2.52574100	1.29131100	-2.00189400

int-4

B3LYP-D3(BJ)/6-31G(d)·SCF energy: -4307.954723 a.u.

B3LYP-D3(BJ)/6-31G(d)·Thermal correction to enthalpy: 0.664186

B3LYP-D3(BJ)/6-31G(d)·Thermal correction to Gibbs free energy: 0.547147

M06/6-311+G(d,p)·SCF energy in solution: -4309.797289 a.u.

Ni	1.27791400	-0.02226000	-0.31079000
C	-1.43055900	1.04161700	-0.31033000
C	-2.48567900	1.95066900	-0.35919800
C	-2.24366200	3.31942300	-0.53258700
C	-0.90559000	3.70948100	-0.65005800
C	0.10086300	2.74996300	-0.59022000
N	-0.15187400	1.45304500	-0.42420800

H	-3.50394300	1.59500600	-0.26340000
H	-0.62899800	4.74655700	-0.78879300
H	1.14738800	3.02094700	-0.68066800
C	-0.53596100	-2.46081400	0.02317700
C	-1.74427500	-3.14604200	0.11558100
C	-2.94785700	-2.43461400	0.07705300
C	-2.85002500	-1.04376300	-0.05791800
C	-1.60734800	-0.41944000	-0.14586200
N	-0.46254100	-1.13531200	-0.09855200
H	0.41114400	-2.98954100	0.04263400
H	-1.72673400	-4.22393500	0.21152300
H	-3.75138000	-0.44562500	-0.10205700
C	1.18387200	0.53101000	3.90068200
C	2.15847800	1.51427900	4.08391600
C	2.87844500	1.98065500	2.98123500
C	2.62489300	1.45988100	1.70840900
C	1.64350000	0.47963300	1.51148400
C	0.92928600	0.02169300	2.62277600
H	0.62397100	0.15567100	4.75468100
H	3.64522200	2.74101300	3.11329900
H	3.22147500	1.80500100	0.86528100
H	0.16873100	-0.74720700	2.50182600
Br	1.50574300	-0.01847200	-2.71730800
H	2.35962200	1.90859800	5.07640100
C	2.62113600	-1.41589200	-0.00442300
H	2.48980900	-2.02350100	-0.90473400
H	2.35544600	-1.97173600	0.90240700
C	-4.32270500	-3.10159100	0.16757300
C	-3.41477100	4.30397800	-0.58734900
C	-4.33525600	3.92516300	-1.76859900
H	-4.74207500	2.91449700	-1.66044800
H	-5.17954200	4.62153000	-1.82425100
H	-3.79118200	3.96866600	-2.71806600
C	-4.20676600	4.21892900	0.73609100
H	-3.57086400	4.47625800	1.59000700
H	-5.05041400	4.91790000	0.71384900
H	-4.60918100	3.21508700	0.90663400
C	-2.94186700	5.75369700	-0.78165700

H	-2.30010200	6.08519200	0.04197000
H	-2.39208800	5.87864700	-1.72080800
H	-3.80971900	6.42042100	-0.81455500
C	-5.12081400	-2.78363300	-1.11631400
H	-6.10879700	-3.25515700	-1.06913500
H	-5.27003200	-1.70712900	-1.24775800
H	-4.60200600	-3.16198700	-2.00347300
C	-5.07301100	-2.54316700	1.39694000
H	-5.21834500	-1.46021600	1.32882300
H	-6.06169800	-3.00888200	1.47563100
H	-4.52199300	-2.75185800	2.32037800
C	-4.21571700	-4.62832800	0.31153200
H	-3.71202500	-5.08280400	-0.54850700
H	-3.67479900	-4.91383800	1.22046600
H	-5.21940700	-5.06110600	0.37391100
O	3.90761800	-0.89191300	0.03797500
Si	5.07590300	-1.18316200	1.21100400
C	6.13931500	-2.62581900	0.62261700
H	6.94384100	-2.84842900	1.33484200
H	5.53872900	-3.53571900	0.50270500
H	6.59844400	-2.40219600	-0.34716800
C	4.30899200	-1.61159800	2.87647300
H	5.10732700	-1.77872800	3.61109900
H	3.66647900	-0.80878000	3.24829400
H	3.71249500	-2.53033500	2.83217200
C	6.11232100	0.37924400	1.31780600
H	7.02010600	0.21088300	1.91002900
H	6.41813500	0.70852600	0.31822100
H	5.54672100	1.19114500	1.78533700

int-4-1

B3LYP-D3(BJ)/6-31G(d)·SCF energy: -4307.950639 a.u.

B3LYP-D3(BJ)/6-31G(d)·Thermal correction to enthalpy: 0.664566

B3LYP-D3(BJ)/6-31G(d)·Thermal correction to Gibbs free energy: 0.548436

M06/6-311+G(d,p)·SCF energy in solution: -4309.793086 a.u.

Ni	1.24815100	-0.46725200	1.59345600
C	-1.24159600	0.84220900	0.87485600

C	-2.19087300	1.85384200	0.73970500
C	-1.94056500	3.13971300	1.23619700
C	-0.69788200	3.34819000	1.84552700
C	0.20686500	2.29627300	1.94603600
N	-0.06450400	1.07474800	1.48696600
H	-3.13126400	1.64277300	0.24625600
H	-0.41988200	4.31412600	2.24652000
H	1.17899600	2.42122000	2.40835100
C	-0.45690100	-2.63299900	0.18683400
C	-1.58792400	-3.14917400	-0.44230400
C	-2.69028000	-2.32221700	-0.67949900
C	-2.58222000	-0.99429900	-0.24535700
C	-1.42115000	-0.54104600	0.37697500
N	-0.36671400	-1.36168400	0.57780000
H	0.41174900	-3.24988000	0.38965900
H	-1.58854800	-4.19054200	-0.73748700
H	-3.41023400	-0.31226000	-0.39324100
C	4.30554800	-2.91160400	3.22461600
C	4.60638400	-3.78032300	2.17554100
C	3.90483500	-3.66754500	0.97403600
C	2.91225900	-2.69466200	0.82596400
C	2.57719100	-1.83666500	1.88397000
C	3.29283100	-1.95749700	3.08250100
H	4.85422500	-2.97769000	4.16172000
H	4.14512400	-4.32171100	0.13862400
H	2.43490800	-2.57290600	-0.14181200
H	3.06799500	-1.29991800	3.91895200
Br	2.45233600	0.55682800	-0.24136300
H	5.38564400	-4.52969900	2.28827300
C	0.48398000	-0.90804400	3.34180500
H	0.83305600	-1.85972900	3.74348000
H	-0.60254500	-0.92684700	3.18875300
O	0.87856300	0.16216400	4.17811000
Si	-0.10448600	0.85421400	5.34898500
C	-1.87195200	1.05636100	4.71385900
H	-2.48232700	1.56067300	5.47374700
H	-1.91028600	1.65861700	3.80064200
H	-2.34722000	0.09189000	4.50234100

C	0.66054800	2.52995700	5.71681100
H	0.16910000	3.01226000	6.57032800
H	1.72481700	2.42623100	5.95605200
H	0.57522300	3.20307200	4.85646500
C	-0.11501400	-0.23606400	6.88446100
H	0.89633200	-0.34589000	7.29230100
H	-0.75363200	0.18200800	7.67239200
H	-0.48862500	-1.24015200	6.65033300
C	-3.97007700	-2.80167700	-1.36912200
C	-3.00622900	4.23085800	1.10906600
C	-2.53886100	5.56691900	1.70862400
H	-1.64663700	5.94941400	1.20123000
H	-3.33034500	6.31498900	1.59654500
H	-2.31738700	5.47664600	2.77775500
C	-3.33487200	4.44949100	-0.38393500
H	-3.71803100	3.53867700	-0.85521300
H	-4.09956700	5.22723000	-0.48906600
H	-2.44490800	4.76603400	-0.93808600
C	-4.27594200	3.77250600	1.86078700
H	-4.06020100	3.59435200	2.91996000
H	-5.05162200	4.54347300	1.79293200
H	-4.68551500	2.84777800	1.44145600
C	-4.19553000	-1.96288300	-2.64657000
H	-5.10882800	-2.29219300	-3.15464400
H	-4.30414500	-0.89720500	-2.42053700
H	-3.35678200	-2.07536000	-3.34173400
C	-3.89205100	-4.28500800	-1.76493800
H	-3.75582900	-4.93200200	-0.89144100
H	-4.82475100	-4.58246000	-2.25509100
H	-3.07328600	-4.47499300	-2.46733700
C	-5.16339900	-2.61210600	-0.40672500
H	-6.08881900	-2.94968900	-0.88654900
H	-5.02239000	-3.19307000	0.51117100
H	-5.29747800	-1.56283400	-0.12439500

TS-2

B3LYP-D3(BJ)/6-31G(d)·SCF energy: -4307.946082 a.u.

B3LYP-D3(BJ)/6-31G(d)·Thermal correction to enthalpy: 0.663276

B3LYP-D3(BJ)/6-31G(d)·Thermal correction to Gibbs free energy: 0.548160

M06/6-311+G(d,p)·SCF energy in solution: -4309.786695 a.u.

Imaginary frequency: -291.90 cm^{-1}

Ni	1.10253300	-1.01074500	0.24657100
C	-0.92667800	1.11488000	-0.05907100
C	-1.54938100	2.36110300	-0.11443700
C	-0.83092300	3.53730800	0.13224700
C	0.52581300	3.38363000	0.43319300
C	1.09002000	2.11092900	0.46353400
N	0.39170900	0.99539900	0.22073600
H	-2.60207900	2.41814000	-0.36133700
H	1.15907600	4.23646100	0.64217900
H	2.13696000	1.95341700	0.69356700
C	-1.41584700	-2.44319000	-0.57271000
C	-2.78858100	-2.60495000	-0.74423400
C	-3.63534600	-1.49352100	-0.68440800
C	-3.02532700	-0.25201900	-0.45972400
C	-1.64405500	-0.15883600	-0.30058400
N	-0.85548100	-1.25166300	-0.34954400
H	-0.71991100	-3.27418100	-0.62628600
H	-3.17544800	-3.60048100	-0.92042500
H	-3.63490700	0.64084300	-0.39649100
C	0.47474800	-3.21466300	3.60686000
C	-0.13667900	-2.31439300	4.48139900
C	-0.09416100	-0.94515500	4.19722900
C	0.54307300	-0.48233000	3.04724100
C	1.12343800	-1.38751400	2.13930300
C	1.11348700	-2.75801600	2.45117500
H	0.45535900	-4.28059700	3.82133000
H	-0.55386800	-0.23376600	4.87973100
H	0.58537100	0.58467000	2.84928800
H	1.57819900	-3.46039100	1.76537300
Br	1.96175900	-2.87849500	-1.13564200
H	-0.62916200	-2.67156400	5.38161900
C	2.93393800	-0.87462400	1.39168000
H	3.36313500	-1.83348100	1.09879000
H	3.17856700	-0.63263500	2.42347500

O	3.36987900	0.20241700	0.59945200
Si	4.29952600	0.22636400	-0.82135000
C	5.36558700	-1.31126400	-0.93689600
H	6.03467400	-1.24366700	-1.80385900
H	5.98814700	-1.43717200	-0.04297000
H	4.73725200	-2.19900800	-1.05887300
C	5.35678400	1.77141000	-0.59450200
H	5.99590000	1.94232900	-1.46965200
H	4.73152800	2.66320000	-0.46360600
H	6.00474200	1.68532600	0.28517000
C	3.18951800	0.45724000	-2.31900900
H	3.78935200	0.65674800	-3.21643400
H	2.59144700	-0.44234900	-2.49216300
H	2.50896100	1.30526100	-2.17489500
C	-5.15519000	-1.58211300	-0.84674600
C	-1.53241900	4.89592300	0.06359700
C	-5.58667900	-0.71406100	-2.04928000
H	-6.67367700	-0.76520200	-2.17883500
H	-5.31586800	0.33770300	-1.91057000
H	-5.11470800	-1.06419300	-2.97362700
C	-5.62705300	-3.02451600	-1.09159500
H	-5.36915600	-3.68296000	-0.25507300
H	-6.71632400	-3.03990200	-1.20235400
H	-5.19428600	-3.44316200	-2.00666700
C	-5.82949000	-1.05687400	0.43994700
H	-6.91965700	-1.11311300	0.34273400
H	-5.53133300	-1.65364800	1.30864500
H	-5.56492700	-0.01382600	0.64188900
C	-2.10923300	5.09330800	-1.35572900
H	-2.83639400	4.31611400	-1.61193000
H	-2.61691900	6.06210500	-1.42281300
H	-1.31344400	5.07088200	-2.10801300
C	-2.67979400	4.92331900	1.09743300
H	-2.29518900	4.77897400	2.11286900
H	-3.19513800	5.88965900	1.06056500
H	-3.42112900	4.14137100	0.90388800
C	-0.57239600	6.05747800	0.36732000
H	-0.14823900	5.98091900	1.37446000

H	0.25220700	6.10115000	-0.35249700
H	-1.11504100	7.00656300	0.30754200

TS-2-1

B3LYP-D3(BJ)/6-31G(d)·SCF energy: -4307.937312 a.u.

B3LYP-D3(BJ)/6-31G(d)·Thermal correction to enthalpy: 0.662951

B3LYP-D3(BJ)/6-31G(d)·Thermal correction to Gibbs free energy: 0.547664

M06/6-311+G(d,p)·SCF energy in solution: -4309.783804 a.u.

Imaginary frequency: -356.27 cm^{-1}

Ni	-1.05480900	-0.32150600	0.08282300
C	1.58194600	0.93441800	-0.25605400
C	2.59598000	1.88986600	-0.31031500
C	2.29348100	3.25735700	-0.29804600
C	0.93809200	3.59716900	-0.23708700
C	-0.02496200	2.59226200	-0.19265800
N	0.28136100	1.29291400	-0.20361000
H	3.62990100	1.57195600	-0.36053600
H	0.61478900	4.63028800	-0.22183600
H	-1.08496200	2.81585900	-0.14942400
C	0.88145000	-2.63026100	-0.14698100
C	2.12627400	-3.24502400	-0.05205900
C	3.28745000	-2.46605700	-0.04694600
C	3.10933700	-1.08017300	-0.13585400
C	1.83365400	-0.52563900	-0.22509600
N	0.72905200	-1.30653400	-0.24323700
H	-0.03593200	-3.20821700	-0.14508000
H	2.16770900	-4.32375300	0.02733600
H	3.97306800	-0.42783900	-0.11247000
C	-4.07360400	-3.18734100	0.22393200
C	-3.68294900	-4.12383300	-0.73941300
C	-2.65187600	-3.80098300	-1.62402700
C	-2.02254000	-2.55700400	-1.54731000
C	-2.38518500	-1.61786300	-0.56188800
C	-3.44381100	-1.94682000	0.30722100
H	-4.86623600	-3.43070500	0.92746100
H	-2.33988300	-4.51568800	-2.38235200
H	-1.23713800	-2.30996500	-2.25910900

H	-3.72798600	-1.25334900	1.08996100
Br	-1.04162800	-0.32825300	2.51337000
H	-4.17815200	-5.08870400	-0.80046400
C	-2.42527500	0.17946300	-1.41726100
H	-3.25137800	-0.21666500	-2.00287500
H	-1.60955000	0.44549200	-2.09789800
C	4.69656900	-3.05321700	0.06689200
C	3.42074000	4.29245300	-0.34623300
C	4.34018300	4.09176400	0.87877300
H	4.79034600	3.09393600	0.89058500
H	5.15343700	4.82627300	0.86265900
H	3.78103600	4.21855200	1.81185500
C	4.23525400	4.09355100	-1.64334200
H	3.60213300	4.22728500	-2.52730000
H	5.04976100	4.82518100	-1.69179700
H	4.67999600	3.09452800	-1.69593600
C	2.88406800	5.73280200	-0.32268800
H	2.23994000	5.94017500	-1.18431300
H	2.31589800	5.93862100	0.59090500
H	3.72195400	6.43687200	-0.35794800
C	5.52031700	-2.63537300	-1.17095300
H	5.60410500	-1.54706400	-1.25600200
H	6.53469100	-3.04447600	-1.10286200
H	5.06112100	-3.01097800	-2.09195300
C	5.36716300	-2.50382200	1.34554200
H	4.79262300	-2.77821700	2.23649600
H	6.37737000	-2.91666400	1.44662300
H	5.45174700	-1.41258300	1.32413600
C	4.67777700	-4.58829800	0.14595300
H	4.12986200	-4.94210500	1.02588300
H	4.22477100	-5.03505300	-0.74599100
H	5.70353100	-4.96406400	0.22099800
O	-2.81608100	1.28563900	-0.65421700
Si	-4.37509800	1.63201900	-0.06650000
C	-4.55497700	3.48094900	-0.36468200
H	-5.52898000	3.84362800	-0.01395600
H	-3.78178100	4.03781300	0.17861900
H	-4.46332500	3.72702700	-1.42863000

C	-4.49012700	1.24871700	1.76960100
H	-5.16200700	0.40517500	1.96527300
H	-3.50435700	0.98654300	2.17388100
H	-4.87073400	2.11362700	2.32622600
C	-5.62944800	0.65145600	-1.07101100
H	-5.48504200	-0.42735000	-0.94243700
H	-6.64468500	0.89175800	-0.73168200
H	-5.57498600	0.88113100	-2.14160600

int-5

B3LYP-D3(BJ)/6-31G(d)·SCF energy: -4308.028043 a.u.

B3LYP-D3(BJ)/6-31G(d)·Thermal correction to enthalpy: 0.666011

B3LYP-D3(BJ)/6-31G(d)·Thermal correction to Gibbs free energy: 0.550048

M06/6-311+G(d,p)·SCF energy in solution: -4309.868301 a.u.

Ni	1.30138200	-1.03819700	-0.61753200
C	-1.03396900	0.59990400	-0.58016800
C	-1.84070100	1.73173100	-0.48851700
C	-1.29082900	3.01706000	-0.54050600
C	0.09753600	3.09091000	-0.70580000
C	0.84694600	1.92424500	-0.79348300
N	0.31113500	0.69722800	-0.73150600
H	-2.90941000	1.61048300	-0.36123800
H	0.61062800	4.04305600	-0.75797200
H	1.92505000	1.95727000	-0.88745800
C	-0.96766100	-3.02226900	-0.50963800
C	-2.27209200	-3.40915200	-0.21452600
C	-3.26518700	-2.43829700	-0.03472300
C	-2.87047400	-1.10303600	-0.18488200
C	-1.54704000	-0.78217300	-0.47757700
N	-0.60566000	-1.74041300	-0.63216200
H	-0.15985900	-3.73776300	-0.63291800
H	-2.49180000	-4.46508700	-0.11906200
H	-3.59494300	-0.31009500	-0.04472300
C	-0.52145900	-1.11414100	2.70422000
C	-1.07331400	0.14984600	2.91440400
C	-0.27406900	1.28926800	2.78567300
C	1.06995800	1.16017900	2.43900200

C	1.62552200	-0.10215400	2.20337700
C	0.82171100	-1.23977900	2.34465700
H	-1.14199400	-2.00180000	2.78833400
H	-0.70036200	2.27506400	2.95002100
H	1.68922100	2.04565800	2.31946400
H	1.24661400	-2.21704300	2.13680900
Br	2.55846500	-3.09251100	-0.64352900
H	-2.12362900	0.24840000	3.17590300
C	3.04028900	-0.23125200	1.70061800
H	3.40406300	-1.25369600	1.84059700
H	3.70424000	0.46369100	2.23250400
O	3.06078600	0.07889100	0.29444200
Si	4.53509500	0.20412700	-0.55065600
C	4.07247300	0.06671600	-2.35993000
H	3.29842600	0.79211000	-2.63698500
H	4.94444200	0.24366800	-3.00204900
H	3.68478200	-0.93685900	-2.56247600
C	5.72725600	-1.13622700	-0.00541400
H	6.01622900	-1.03118600	1.04702000
H	5.25125300	-2.11286800	-0.14048400
H	6.64482800	-1.10548900	-0.60593300
C	5.23226900	1.91568800	-0.15710000
H	6.19117400	2.07643200	-0.66518300
H	4.54752800	2.70895300	-0.48130300
H	5.40634700	2.04487200	0.91808000
C	-2.19114800	4.24583600	-0.39347700
C	-4.71302600	-2.77081600	0.33571400
C	-4.95165700	-4.28609200	0.43524700
H	-5.99762800	-4.47696600	0.69750400
H	-4.74936900	-4.79100900	-0.51559800
H	-4.32662000	-4.74530400	1.20876300
C	-5.66177100	-2.19506600	-0.73800100
H	-6.70299700	-2.42224500	-0.48207200
H	-5.56851400	-1.10762100	-0.82358000
H	-5.44811100	-2.62923800	-1.72067400
C	-5.03404300	-2.13286400	1.70593400
H	-6.06709700	-2.35708100	1.99562900
H	-4.36800800	-2.52393500	2.48304800

H	-4.92057500	-1.04404200	1.68301700
C	-3.26594700	4.23079300	-1.50204600
H	-3.89289800	3.33494900	-1.44963800
H	-3.92133500	5.10375300	-1.40387000
H	-2.80335800	4.25963400	-2.49451400
C	-1.39881100	5.55878200	-0.49996300
H	-2.08011500	6.40939600	-0.39318400
H	-0.64121400	5.64027500	0.28720500
H	-0.89920100	5.65250400	-1.47042400
C	-2.87510600	4.19703600	0.99110100
H	-2.12932600	4.20504300	1.79373700
H	-3.52890000	5.06694700	1.12215700
H	-3.48577500	3.29621800	1.11003100

(benzyloxy)trimethylsilane

B3LYP-D3(BJ)/6-31G(d)·SCF energy: -755.539463 a.u.

B3LYP-D3(BJ)/6-31G(d)·Thermal correction to enthalpy: 0.251624

B3LYP-D3(BJ)/6-31G(d)·Thermal correction to Gibbs free energy: 0.191586

M06/6-311+G(d,p)·SCF energy in solution: -755.258619 a.u.

C	-0.50031600	-0.68189100	2.74094900
C	-1.06244900	0.49050800	2.22976500
C	-0.30618600	1.30587000	1.38888900
C	1.00499000	0.95648200	1.05820400
C	1.57021300	-0.21343700	1.56687500
C	0.80735400	-1.02982100	2.41006900
H	-1.08152600	-1.32489500	3.39639700
H	-0.73644100	2.21920900	0.98653900
H	1.60052000	1.58316800	0.40454400
H	1.24051100	-1.94487600	2.81006500
H	-2.08233800	0.76362300	2.48598300
C	2.98972700	-0.61665400	1.23165400
H	2.98496600	-1.62681000	0.79233100
H	3.57664100	-0.67934000	2.16205100
O	3.58288400	0.30472200	0.33789500
Si	5.16319200	0.16245900	-0.20707200
C	5.40995200	1.62367200	-1.35570700
H	5.26059800	2.57032700	-0.82466100

H	6.42271300	1.62990600	-1.77600400
H	4.69999300	1.59151100	-2.18958200
C	5.38161000	-1.46540100	-1.13043300
H	5.21452300	-2.33290600	-0.48116400
H	4.68123200	-1.53684300	-1.97054600
H	6.39788200	-1.55084200	-1.53422000
C	6.35658300	0.24095100	1.24896200
H	7.39638800	0.19818800	0.90230100
H	6.22966000	1.17382600	1.81022500
H	6.21105900	-0.59170900	1.94716800

2a

B3LYP-D3(BJ)/6-31G(d)·SCF energy: -1036.291951 a.u.

B3LYP-D3(BJ)/6-31G(d)·Thermal correction to enthalpy: 0.269317

B3LYP-D3(BJ)/6-31G(d)·Thermal correction to Gibbs free energy: 0.203613

M06/6-311+G(d,p)·SCF energy in solution: -1035.939213 a.u.

C	4.23214100	0.18973500	-1.27557900
C	4.27210300	-1.09422100	-0.72064500
C	3.22831700	-1.56552200	0.08763300
C	2.16254000	-0.71108400	0.31666800
C	2.12178600	0.57201200	-0.23916400
C	3.14672500	1.04537700	-1.04168800
H	5.05584700	0.52731600	-1.89740500
H	5.12577800	-1.73483500	-0.92053000
H	3.24592000	-2.55973200	0.52213700
H	3.10314800	2.04235900	-1.46795900
C	0.91627800	-0.93546300	1.10717700
C	0.85290800	1.23520100	0.18067500
O	0.56947900	-1.91110000	1.73701900
O	0.43355800	2.34391300	-0.07100900
N	0.18643800	0.25283000	0.93699900
O	-0.96443700	0.50757500	1.63631300
C	-2.12509600	0.57047000	0.74821000
H	-2.00833700	1.44132200	0.09201900
H	-2.93723500	0.79838400	1.44707000
Si	-2.55036300	-1.02682800	-0.22063200
C	-4.17472300	-0.62807900	-1.10350600

H	-4.52025000	-1.49082400	-1.68546900
H	-4.96871400	-0.37037900	-0.39281400
H	-4.06007600	0.21334200	-1.79714700
C	-1.22671700	-1.42141400	-1.51293200
H	-0.90451500	-0.52194800	-2.05050200
H	-0.34237000	-1.88688400	-1.06723900
H	-1.62407400	-2.12418200	-2.25539200
C	-2.75520200	-2.44910500	0.99253300
H	-2.96654500	-3.39016700	0.47104900
H	-1.83696500	-2.57765100	1.57461300
H	-3.57947600	-2.26058300	1.69047100

$ZnCl_2(DMA)_2$

B3LYP-D3(BJ).SCF energy: -1723.418905 a.u.

B3LYP-D3(BJ).Thermal correction to enthalpy: 0.292075

B3LYP-D3(BJ).Thermal correction to Gibbs free energy: 0.214922

B3LYP-D3(BJ).SCF energy in solution: -1723.197278 a.u.

O	1.51520000	-0.28826400	-1.30180000
C	3.62023000	-1.33238100	-0.87738700
H	3.18181700	-2.08452400	-1.53348000
H	4.62447200	-1.08011500	-1.22887300
H	3.69566800	-1.75273100	0.13250200
O	-1.02164300	1.21357000	-0.19956500
C	-2.75273700	1.41455800	-1.83045300
H	-2.96345200	0.46301800	-2.33264400
H	-3.65385500	2.03336600	-1.85263200
H	-1.94987600	1.91771000	-2.36971100
C	-2.25618100	1.12472000	-0.43505100
C	2.68236900	-0.15016000	-0.84573300
N	-3.12473300	0.76623500	0.52348200
N	3.09262200	1.01763200	-0.32867900
C	-4.48386900	0.32028400	0.23787500
H	-4.50221500	-0.77334800	0.15577700
H	-5.14353100	0.63125300	1.05313100
H	-4.85317400	0.74983900	-0.69137300
C	-2.62451500	0.40857900	1.85197600
H	-3.37782600	0.68776700	2.59413600

H	-2.43793400	-0.66964500	1.90823400
H	-1.69388700	0.93647400	2.05048500
C	2.14734400	2.12882900	-0.21623600
H	1.67742400	2.11934000	0.77291300
H	2.69272500	3.06632800	-0.35954700
H	1.37105300	2.02605600	-0.97074300
C	4.29774200	1.15166100	0.48126100
H	5.02355900	0.37743400	0.24069800
H	4.75268000	2.12835800	0.29155000
H	4.03687200	1.07654000	1.54402600
Zn	-0.00119600	-0.59313400	0.04333700
Cl	1.09713100	-0.52731900	1.99218100
Cl	-1.53915600	-2.03481900	-0.66475800

TMSCl

B3LYP-D3(BJ)/6-31G(d)·SCF energy: -869.544426 a.u.

B3LYP-D3(BJ)/6-31G(d)·Thermal correction to enthalpy: 0.123099

B3LYP-D3(BJ)/6-31G(d)·Thermal correction to Gibbs free energy: 0.080899

M06/6-311+G(d,p)·SCF energy in solution: -869.443385 a.u.

Si	0.33914200	0.00001800	-0.00000300
C	0.89686000	-0.99587500	-1.49008200
H	0.52826600	-2.02571500	-1.43765700
H	0.53045500	-0.55205600	-2.42172700
H	1.99253300	-1.02971100	-1.53832400
C	0.89692300	-0.79255400	1.60744600
H	0.52878000	-0.23206200	2.47317900
H	0.53013400	-1.82114500	1.68903300
H	1.99260100	-0.81785100	1.66057700
C	0.89694900	1.78838100	-0.11739600
H	0.53055100	2.37329300	0.73278900
H	1.99263300	1.84695700	-0.12250900
H	0.52845500	2.25796100	-1.03548000
Cl	-1.76745800	0.00002200	0.00002100

I

B3LYP-D3(BJ)/6-31G(d)·SCF energy: -921.856748 a.u.

B3LYP-D3(BJ)/6-31G(d)·Thermal correction to enthalpy: 0.235209

B3LYP-D3(BJ)/6-31G(d)·Thermal correction to Gibbs free energy: 0.175966

M06/6-311+G(d,p)·SCF energy in solution: -921.540011 a.u.

C	4.01424200	-0.75474000	0.00019900
C	4.05051900	0.64639000	0.00012700
C	2.87072600	1.40042300	-0.00007600
C	1.67120800	0.70439800	-0.00021900
C	1.63531400	-0.68813800	-0.00015000
C	2.79689300	-1.44624300	0.00006500
H	4.94718800	-1.31067000	0.00036200
H	5.01112700	1.15307100	0.00024500
H	2.88431600	2.48557800	-0.00011900
H	2.75454700	-2.53065300	0.00012000
C	0.26504700	1.20858200	-0.00033100
C	0.20535800	-1.10856100	-0.00026300
O	-0.09836600	2.36762600	-0.00016700
O	-0.23870000	-2.24228500	-0.00009500
N	-0.57160800	0.06911000	-0.00057600
Si	-2.38759500	-0.00294700	0.00002100
C	-2.88719100	-0.92763100	-1.55492900
H	-2.43383400	-1.92299600	-1.57167400
H	-3.97659900	-1.04394200	-1.60385700
H	-2.56761300	-0.38760700	-2.45352000
C	-2.88606000	-0.92603500	1.55628900
H	-3.97549900	-1.04137500	1.60673100
H	-2.43348000	-1.92174800	1.57338800
H	-2.56486000	-0.38538300	2.45392800
C	-3.05474000	1.74758400	-0.00042000
H	-4.15168600	1.70922200	-0.00053300
H	-2.72879000	2.30771200	0.88015100
H	-2.72861000	2.30736600	-0.88114900

II

B3LYP-D3(BJ)/6-31G(d)·SCF energy: -523.764011 a.u.

B3LYP-D3(BJ)/6-31G(d)·Thermal correction to enthalpy: 0.151549

B3LYP-D3(BJ)/6-31G(d)·Thermal correction to Gibbs free energy: 0.105018

M06/6-311+G(d,p)·SCF energy in solution: -523.636872 a.u.

Si	4.34177900	0.28515300	-0.83989700
C	5.41679700	-1.25506700	-0.88366800
H	6.05176700	-1.27104800	-1.77743800
H	6.07135300	-1.30759600	-0.00628200
H	4.79087500	-2.15294700	-0.88906300
C	5.37619500	1.85380700	-0.65506900
H	6.03132100	1.97997600	-1.52590600
H	4.74408600	2.74575500	-0.58180800
H	6.01163400	1.81334900	0.23661200
C	3.18624300	0.38456200	-2.31795200
H	3.74768900	0.42358000	-3.25898700
H	2.52963200	-0.49074900	-2.34402300
H	2.55308700	1.27763800	-2.26791200
O	2.52377800	-0.93722500	0.58554500
C	3.21794200	0.17619400	0.78371400
H	2.63696200	1.12179000	0.78909400
H	3.93994200	0.16026000	1.62645000

TS-3

B3LYP-D3(BJ)/6-31G(d)·SCF energy: -523.754886 a.u.

B3LYP-D3(BJ)/6-31G(d)·Thermal correction to enthalpy: 0.150521

B3LYP-D3(BJ)/6-31G(d)·Thermal correction to Gibbs free energy: 0.105154

M06/6-311+G(d,p)·SCF energy in solution:-523.622813 a.u.

Imaginary frequency: -300.40 cm^{-1}

Si	0.22750700	0.00007200	0.05561300
C	0.44870400	1.59070600	1.02833100
H	1.50468300	1.75339500	1.27540300
H	0.10980300	2.45088900	0.43931300
H	-0.13176400	1.57165800	1.95477000
C	1.54871700	-0.00183200	-1.32753100
H	2.54725100	-0.00119700	-0.86955900
H	1.46874300	-0.89005300	-1.96331300
H	1.46873900	0.88463600	-1.96575700
C	0.44858400	-1.58793500	1.03264800
H	1.50453700	-1.74996600	1.28027000
H	-0.13197600	-1.56638700	1.95897400
H	0.10975000	-2.44970600	0.44591600

O	-1.78924000	0.00050900	0.23896300
C	-1.46402900	-0.00152200	-1.06085000
H	-1.60641000	-0.92706400	-1.63950200
H	-1.60639300	0.92221000	-1.64238500

8. Mechanistic studies

8.1. Radical trap experiment with TEMPO

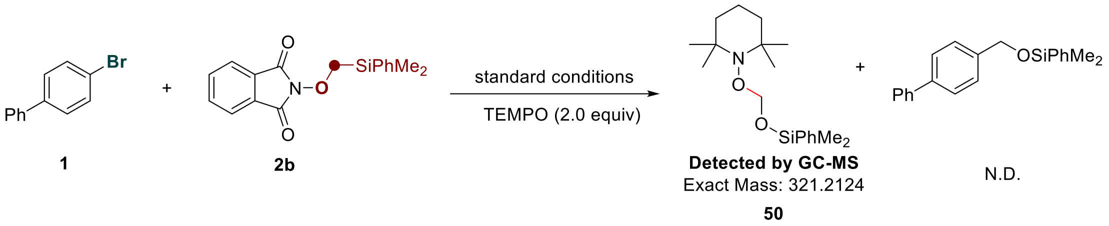

Follow the **General Procedure 2.3.**, add TEMPO (2.0 equiv) and the desired product was not detected. The results were determined by GC-MS. The TEMPO derived adduct **50** was detected by GC-MS.

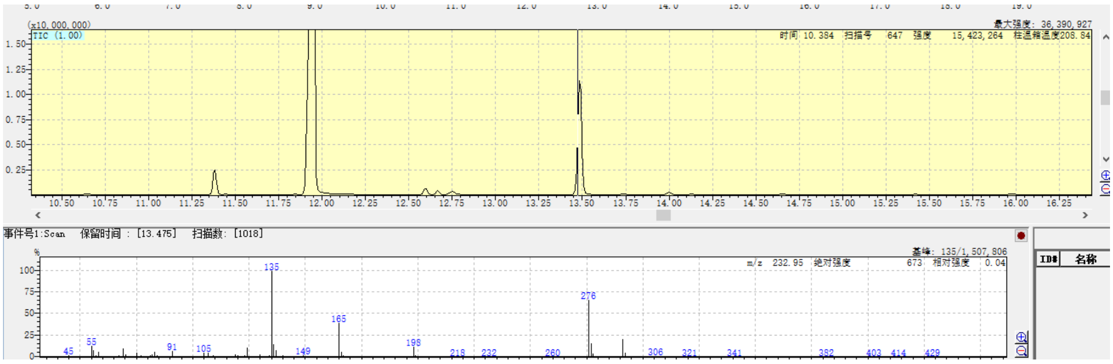

Figure S4. Mass spectrum of the observed adduct.

8.2. Trapping with benzyl acrylate

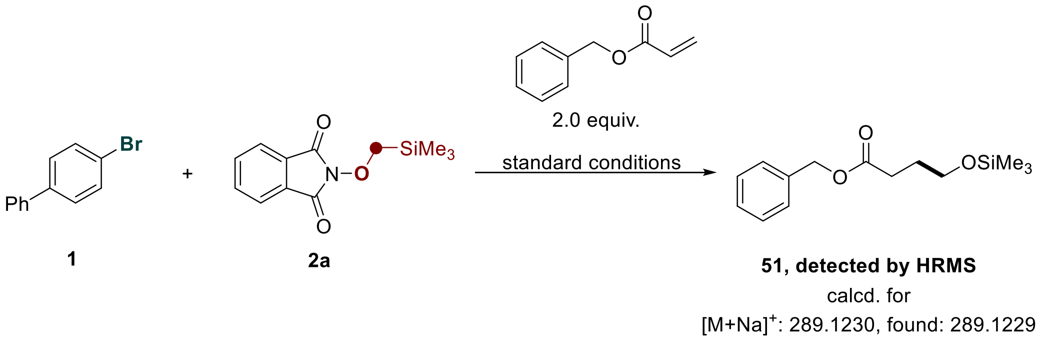

Radical trapping experiment with benzyl acrylate in the presence of Ni catalyst was set up according to **General Procedure 2.3.** with benzyl acrylate (2.0 equiv). Adduct compound **51** was

detected by HRMS.

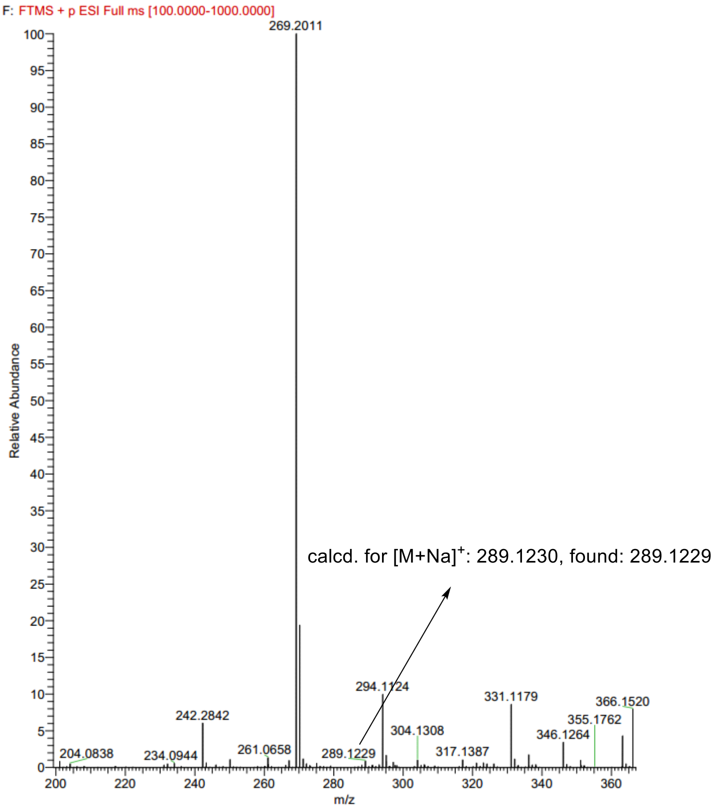

Fig. S5. HRMS of beznyl acrylate radical adduct (**51**).

8.3. Trapping with *N*-benzylacrylamide

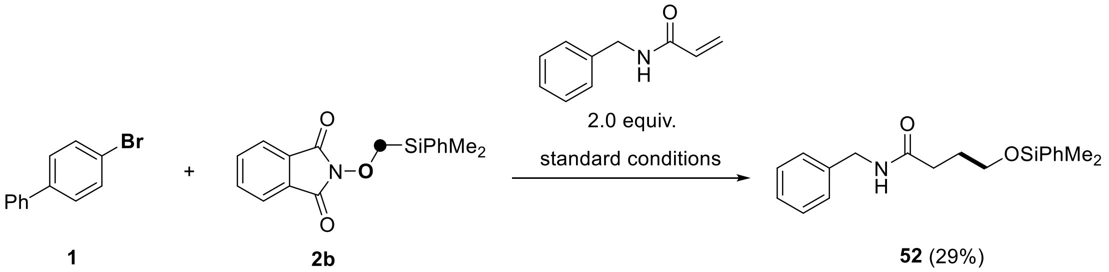

Radical trapping experiment with *N*-benzylacrylamide under the standard conditions was set up according to **General Procedure 2.3.** The adduct **52** was isolated in 29% yield. **^{1}H NMR** (500 MHz, $CDCl_3$) δ 7.47 – 7.44 (m, 2H), 7.33 – 7.24 (m, 5H), 7.22 – 7.16 (m, 3H), 4.30 (d, *J* = 5.7 Hz, 2H), 3.56 (t, *J* = 5.9 Hz, 2H), 2.21 (t, *J* = 7.3 Hz, 2H), 1.86 – 1.74 (m, 2H), 0.27 (s, 6H); **^{13}C NMR** (125 MHz, $CDCl_3$) δ 174.7, 140.4, 135.4, 135.0, 131.7, 130.7, 129.9, 129.8, 129.5, 64.2, 45.5, 35.3, 30.8, -0.8.

8.4. Stiochiometeric experiments with $Ni^{(0)}$

$$\mathbf{1} + \mathbf{2a} \xrightarrow[\text{Then, TBAF (1.2 equiv)}]{\text{Ni(COD)}_2\text{ (1.0 equiv), dtbpy (1.0 equiv); TMSCl (1.0 equiv), KI (1.0 equiv); DMA, RT, 8 h}} \mathbf{3} + \mathbf{2a}$$

Various conditions	3	2a
with Zn (3.0 equiv)	65%	Not detected
w/o Zn	0	recovered: 88%

8.5. Competitive experiments

Competetion experiment

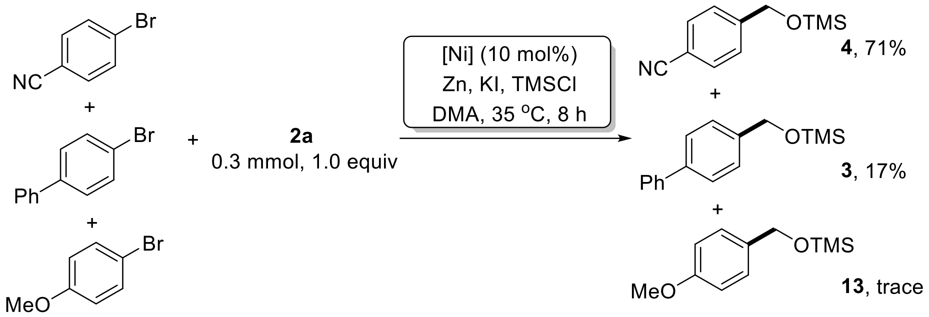

The reaction was set up with equal amount of aryl bromides (0.3 mmol, each 0.1 mmol) and **2a** (0.3 mmol) according to **General Procedure 2.3.** GC-MS analysis of the mixture after 8 h.

8.6. Control experiment with activated Zinc

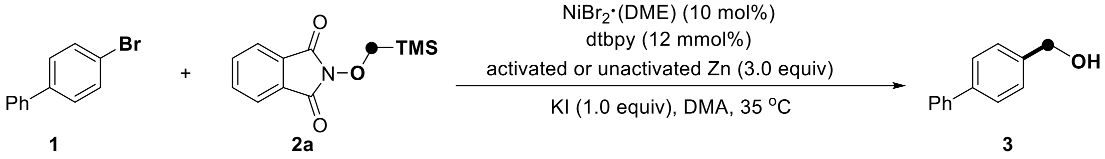

condition	yield of **3**
activated Zn	34%
unactivated Zn	trace

The reaction was set up with equal amount of aryl bromides (0.3 mmol, each 0.1 mmol) and **2a** (0.3 mmol) according to **General Procedure 2.3.** TMSCl may also play a role in activating zinc by removing the surface oxide layer and thereby enhancing the efficiency of electron transfer. To examine this possibility, we performed control experiments using pre-activated zinc. Under these conditions, the reaction still proceeded efficiently, although the presence of TMSCl resulted in slightly improved yields and reproducibility. These observations suggest that TMSCl likely plays a dual role in the reaction: (1) assisting in the activation of zinc by removing the surface oxide layer, and (2) weakly coordinating to the substrate to facilitate its reduction.

8.7. Cyclic voltammetry

Cyclic voltammetry was performed in a three-electrode cell (10 mL), glassy carbon (diameter 3 mm) as the working electrode, Pt wire as the auxiliary electrode, and SCE (saturated calomel electrode) as the reference electrode. All measurement were carried out at a concentration of 0.05

M in anhydrous MeCN with an electrolyte $TBAPF_6$, 0.1M), with a scan rate of 100 mV/s.

A. CV studies of **2a** upto -1.43 V

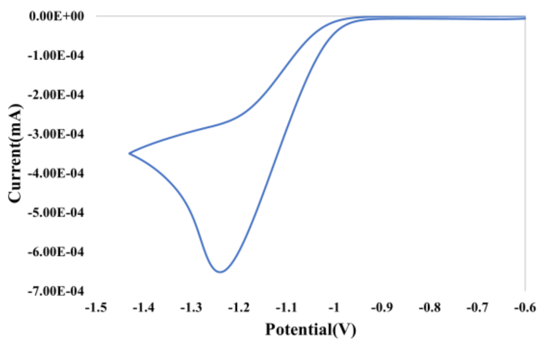

B. CV studies of **2a** upto -2.33 V

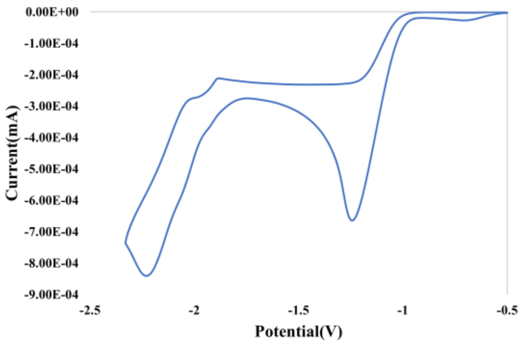

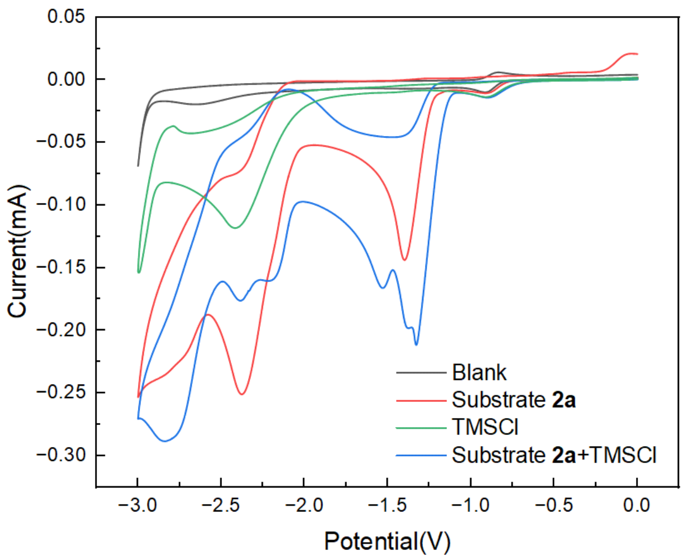

The obtained voltammograms indicate that once compound **2a** undergoes reduction at the cathode, the resulting reduced species cannot be oxidized back to the original compound within the investigated potential window. This behavior suggests that the reduction process is electrochemically irreversible, and no corresponding reversible oxidation process is observed.

8.8 Identification of phthalimide intermediate

Under standard conditions, phthalimide has been observed, indicating the possible formation of N-(trimethylsilyl)phthalimide, which subsequently undergoes desilylation or deprotection, in agreement with DFT studies.

Phthalimide: ^{1}**H NMR** (500 MHz, DMSO) δ 11.36 (s, 1H), 7.90 – 7.80 (m, 4H); ^{13}**C NMR** (125 MHz, DMSO) δ 169.72, 134.88, 133.01, 123.43.

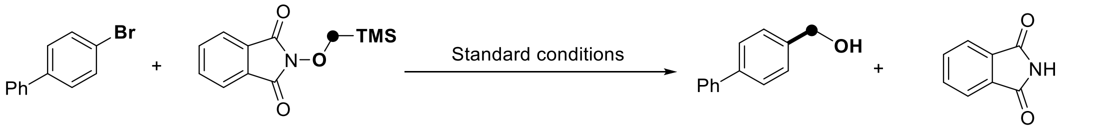

1 (0.2 mmol) **2a** (0.4 mmol) **3** (0.156 mmol, 78%) phthalimide (0.27 mmol)

9. References

[1] D. Fang, Y. Zhang and Y. Chen, *Org. Lett.*, 2022, **24**, 2050–2054.

[2] V. V. Voronin, M. S. Ledovskaya, K. S. Rodygin and V. P. Ananikov, *Org. Chem. Front.*, 2020, **7**, 1334–1342.

[3] W. Guo, J. Huang, H. Wu, T. Liu, Z. Luo, J. Jian and Z. Zeng, *Org. Chem. Front.*, 2018, **5**, 2950–2954.

[4] X.-J. Zhao, Z.-H. Li, T.-M. Ding, J.-M. Tian, Y.-Q. Tu, A.-F. Wang and Y.-Y. Xie, *Angew. Chem., Int. Ed.*, 2021, **60**, 7061–7065.

[5] M. Sayama, A. Inoue, S. Nakamura, S. Jung, M. Ikubo, Y. Otani, A.Uwamizu, T.Kishi, K. Makide, J. Aoki, T. Hirokawa and T. Ohwada, *J. Med. Chem.*, 2017, **60**, 6384–6399.

[6] M. K. Barman, K. Das and B. Maji, *J. Org. Chem.*, 2019, **84**, 1570–1579.

[7] S. R. Tamang, D. Bedi, S. Shafiei-Haghighi, C. R. Smith, C. Crawford and M. Findlater, *Org. Lett.*, 2018, **20**, 6695–6700.

[8] I. S. Turan, D. Yildiz, A. Turksoy, G. Gunaydin and E. U. Akkaya, *Angew. Chem., Int. Ed.*, 2016, **55**, 2875–2878.

[9] H.-T. Song, W. Ding, Q.-Q. Zhou, J. Liu, L.-Q. Lu and W.-J. Xiao, *J. Org. Chem.*, 2016, **81**, 7250–7255.

[10] D. Y. Ong, Z. Yen, A. Yoshii, J. Revillo Imbernon, R. Takita and S. Chiba, *Angew. Chem., Int. Ed.*, 2019, **58**, 4992–4997.

[11] D. Wei, A. Bruneau-Voisine, T. Chauvin, V. Dorcet, T. Roisnel, D. A. Valyaev, N. Lugan and J.-B. Sortais, *Adv. Synth. Catal.*, 2018, **360**, 676–681.

[12] J. Lee, T. Ryu, S. Park and P. H. Lee, *J. Org. Chem.*, 2012, **77**, 4821–4825.

[13] Z. Jia, F. Zhou, M. Liu, X. Li, A.S.C. Chan and C.-J. Li, *Angew. Chem., Int. Ed.*, 2013, **52**, 11871–11874.

[14] R. Das and D. Chakraborty, *Appl. Organometal. Chem.*, 2011, **25**, 437–442.

[15] R. A. Fernandes and P. Kumar, *Tetrahedron Lett.*, 2003, **44**, 1275–1278.

[16] G. D. McAllister, C. D. Wilfred and R. J. Taylor, *Synlett*, 2002, 1291–1292.

[17] H.-P. Li, H.-J. Ai, X. Qi, J.-B. Peng and X.-F. Wu, *Org. Biomol. Chem.*, 2017, **15**, 1343–1345.

[18] A-S. K. Paschke, S. Schiele, C. Pinard, F. Sandrini and B. Morandi, *Chem. Sci.*, 2025, **16**, 11464–11467.

[19] X. Wang, X. Guo, X. Wang, C. Li, S. Wang, H. Li, Y. a. Gao, Y. Li, J. Wang and H. Xu, *RSC Adv.*, 2023, **13**, 13819–13823.

[20] T. Lim, J. Y. Ryoo and M. S. Han, *J. Org. Chem.*, 2020, **85**, 10966–10972.

[21] T. E. Barder, S. D. Walker, J. R. Martinelli and S. L. Buchwald, *J. Am. Chem. Soc.*, 2005, **127**, 4685–4696.

[22] N. Miyaura, T. Yanagi and A. Suzuki, *Synth. Commun.*, 1981, **11**, 513–519.

[23] Gaussian 16, Revision A.03, M. J. Frisch, G. W. Trucks, H. B. Schlegel, G. E. Scuseria, M. A. Robb, J. R. Cheeseman, G. Scalmani, V. Barone, G. A. Petersson, H. Nakatsuji, X. Li, M. Caricato, A. V. Marenich, J. Bloino, B. G. Janesko, R. Gomperts, B. Mennucci, H. P. Hratchian, J. V. Ortiz, A. F. Izmaylov, J. L. Sonnenberg, D. Williams-Young, F. Ding, F. Lipparini, F. Egidi, J. Goings, B. Peng, A. Petrone, T. Henderson, D. Ranasinghe, V. G. Zakrzewski, J. Gao, N. Rega, G. Zheng, W. Liang, M. Hada, M. Ehara, K. Toyota, R. Fukuda, J. Hasegawa, M. Ishida, T. Nakajima, Y. Honda, O. Kitao, H. Nakai, T. Vreven, K. Throssell,

J. A. Montgomery, Jr., J. E. Peralta, F. Ogliaro, M. J. Bearpark, J. J. Heyd, E. N. Brothers, K. N. Kudin, V. N. Staroverov, T. A. Keith, R. Kobayashi, J. Normand, K. Raghavachari, A. P. Rendell, J. C. Burant, S. S. Iyengar, J. Tomasi, M. Cossi, J. M. Millam, M. Klene, C. Adamo, R. Cammi, J. W. Ochterski, R. L. Martin, K. Morokuma, O. Farkas, J. B. Foresman, and D. J. Fox, Gaussian, Inc., Wallingford CT, 2016.

[24] (a) C. Lee, W. Yang and R. G. Parr, *Phys. Rev. B: Condens. Matter Mater.* Phys. 1988, **37**, 785–789. (b) A. D. Becke, *J. Chem. Phys.*, 1993, **98**, 5648–5652.

[25] S. Grimme, J. Antony, S. Ehrlich and H. Krieg, *J. Chem. Phys.*, 2010, **132**, 154104.

[26] S. Grimme, S. Ehrlich and L. Goerigk, *J. Comp. Chem.*, 2011, **32**, 1456–1465.

[27] M. Dolg, U. Wedig, H. Stoll and H. Preuss, *J. Chem. Phys.*, 1987, **86**, 866–872.

[28] Y. Zhao and D. G. Truhlar, *Theor. Chem. Acc.*, 2008, **120**, 215–241.

[29] A. V. Marenich, C. J. Cramer and D. G. Truhlar, *J. Phys. Chem. B*, 2009, **113**, 6378–6396.

[30] C. Y. Legault, *CYLView, 1.0b*; Universitéde Sherbrooke: Québec, Montreal, Canada, 2009; (http://www.cylview.org)

10. NMR Spectra

2-((Trimethylsilyl)methoxy)isoindoline-1,3-dione (2a)

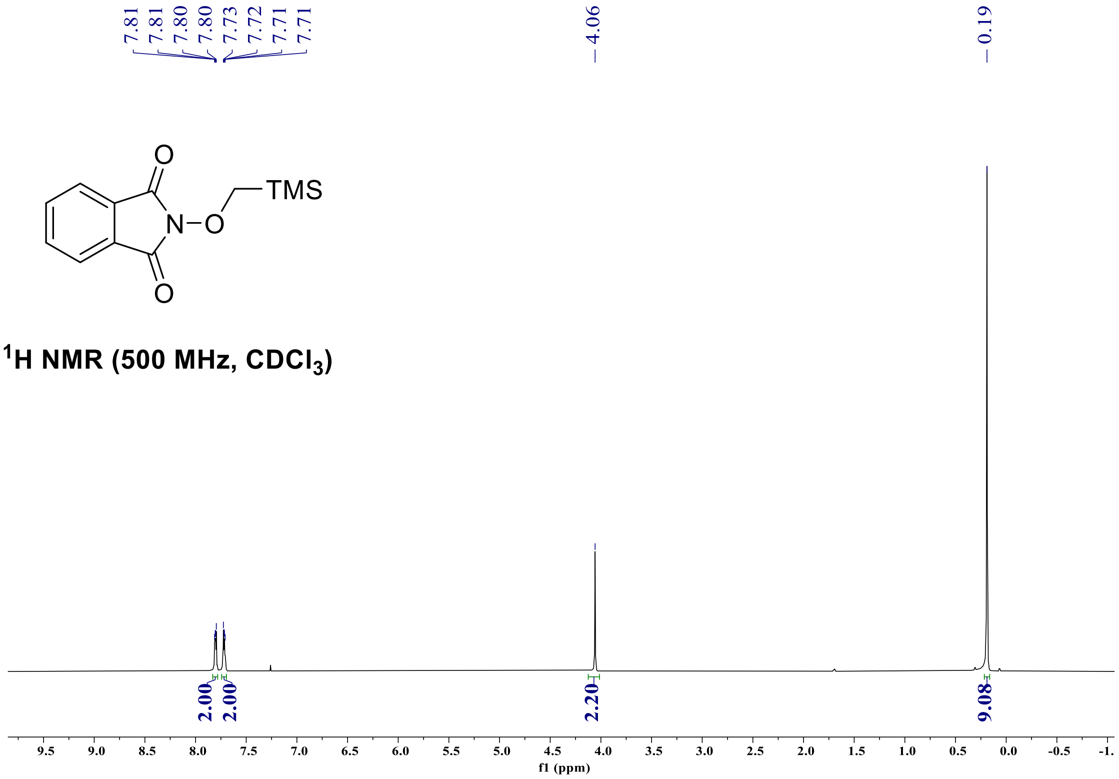

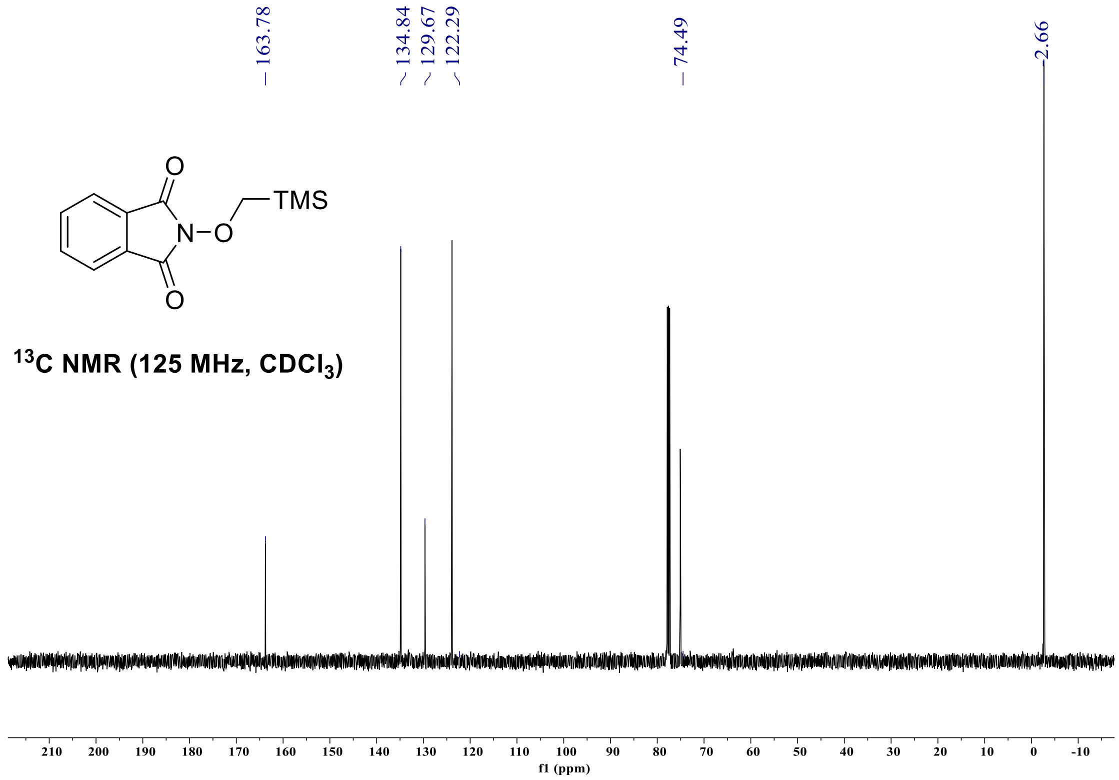

2-((Butyldimethylsilyl)methoxy)isoindoline-1,3-dione (2b)

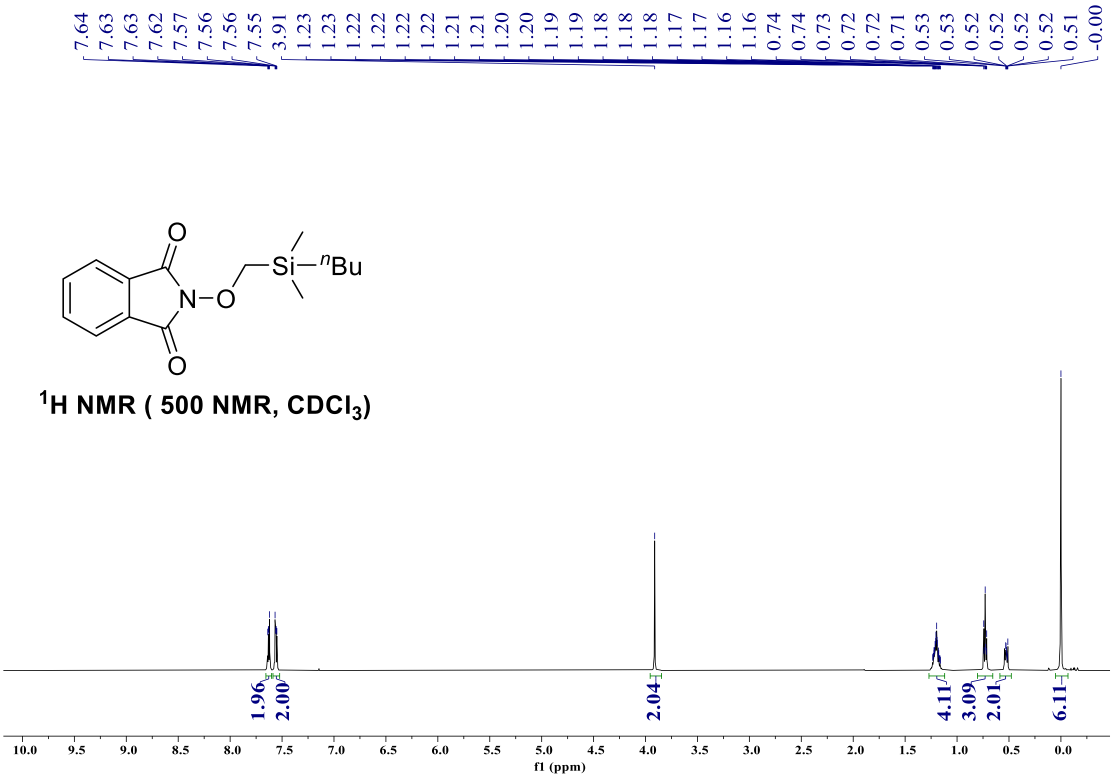

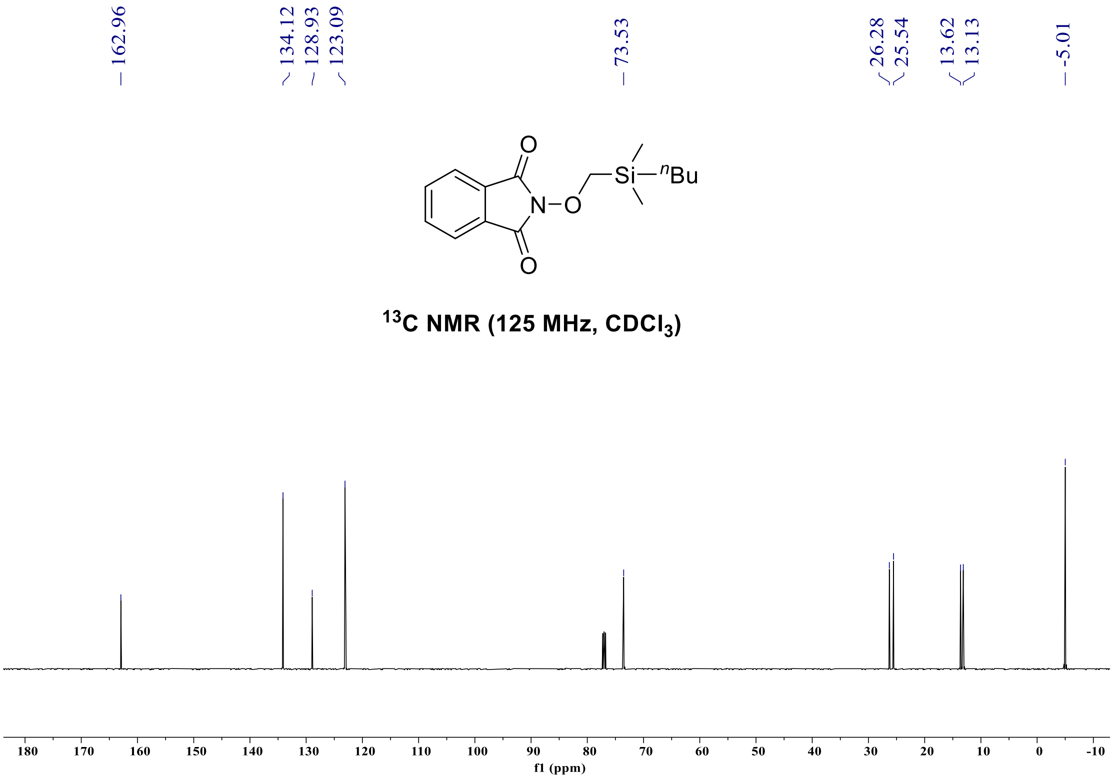

2-((Butyldimethylsilyl)methoxy)isoindoline-1,3-dione (2c)

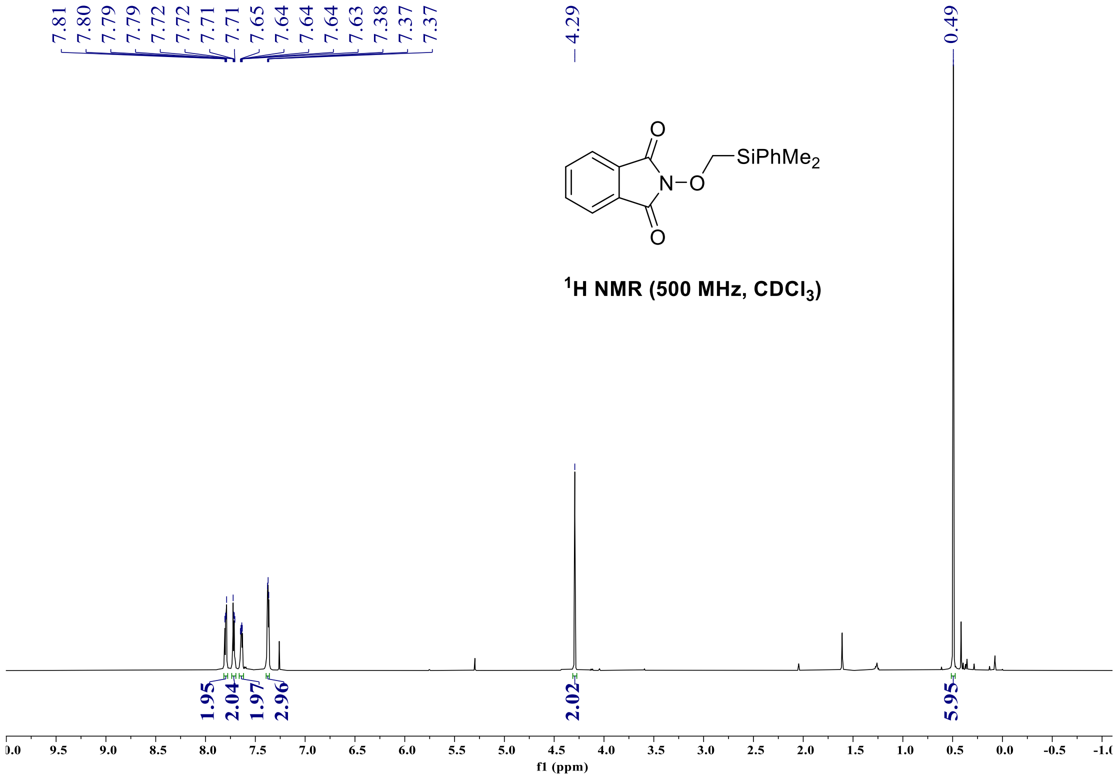

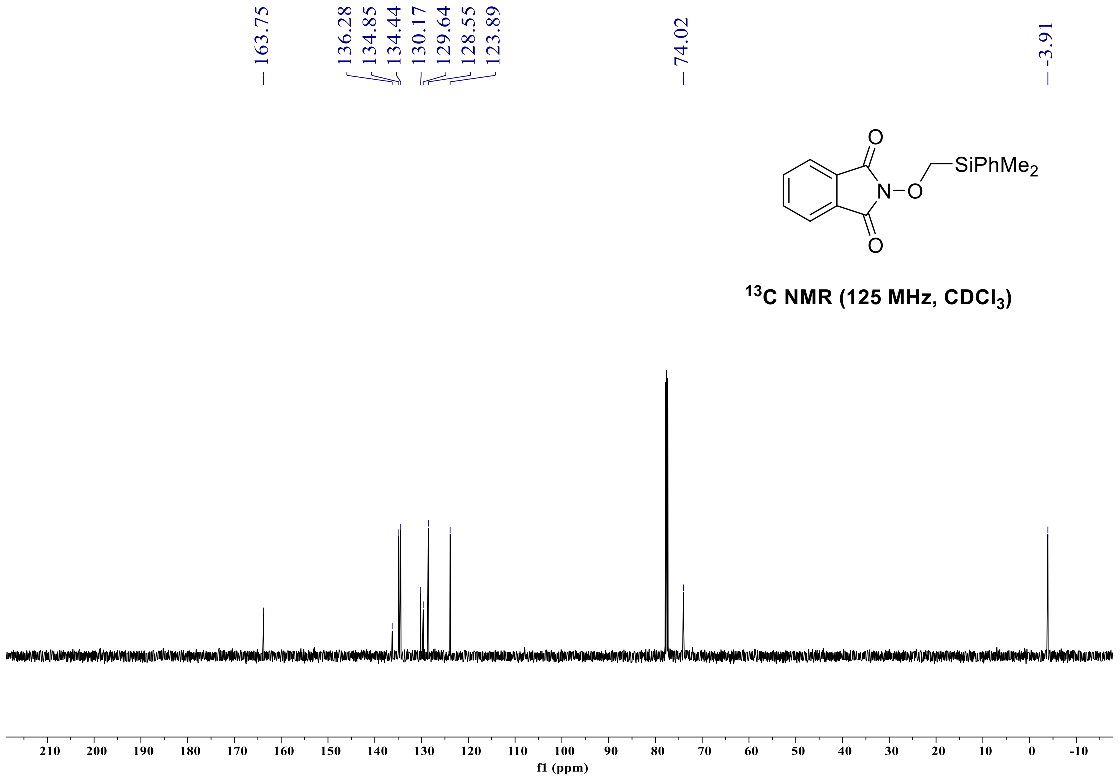

[1,1'-Biphenyl]-4-ylmethanol (3)

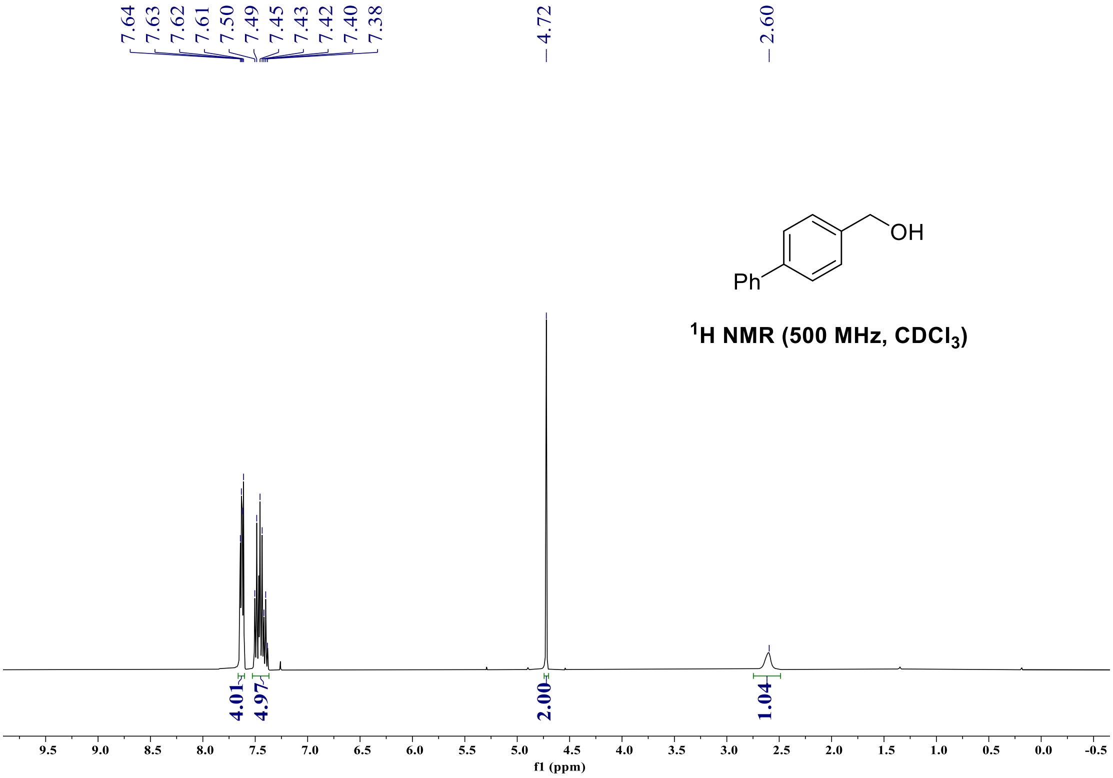

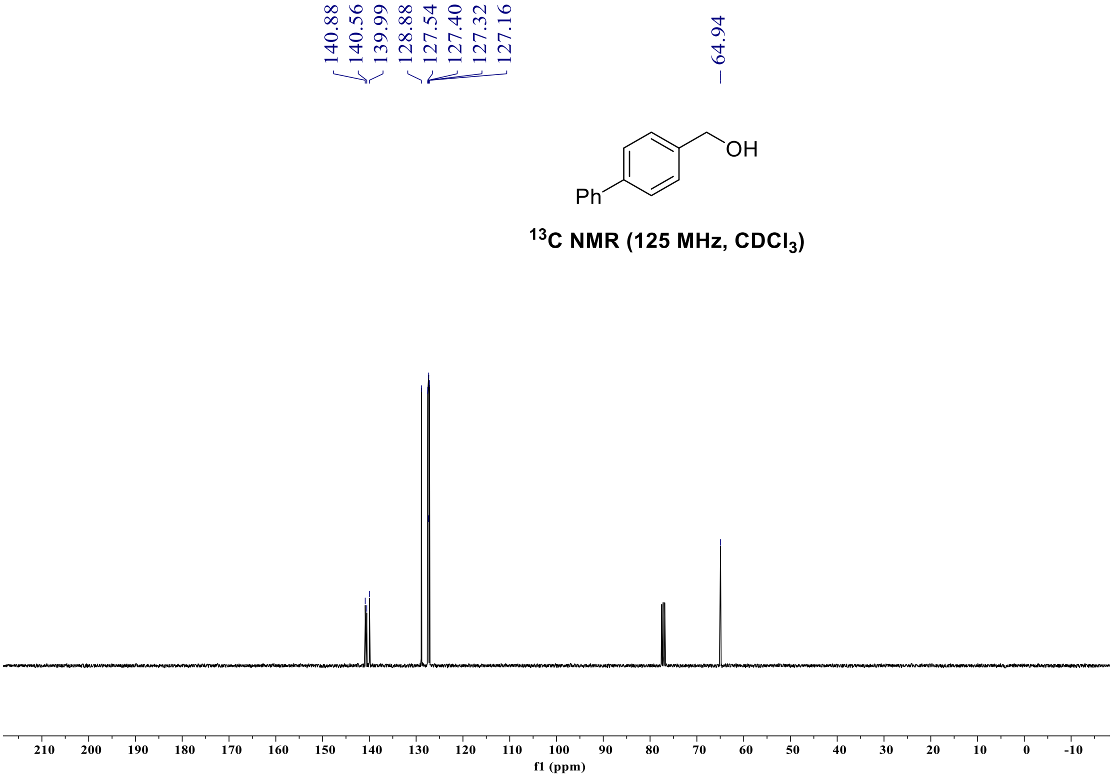

4-(Hydroxymethyl)benzonitrile (4)

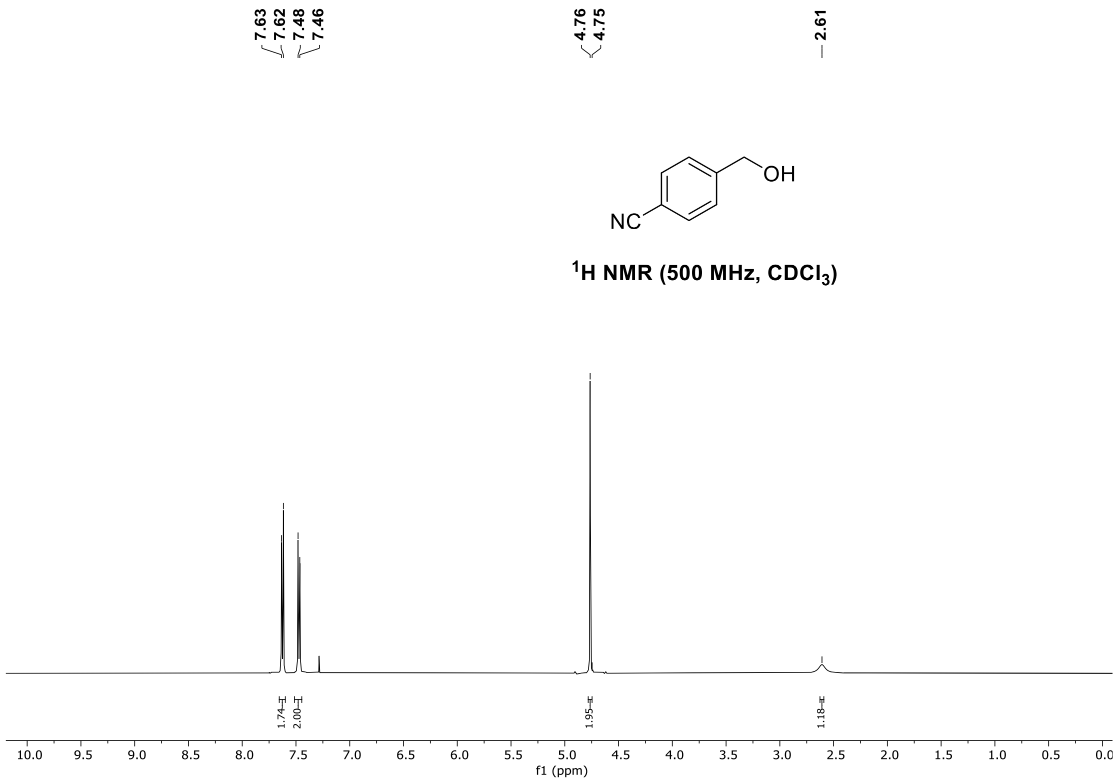

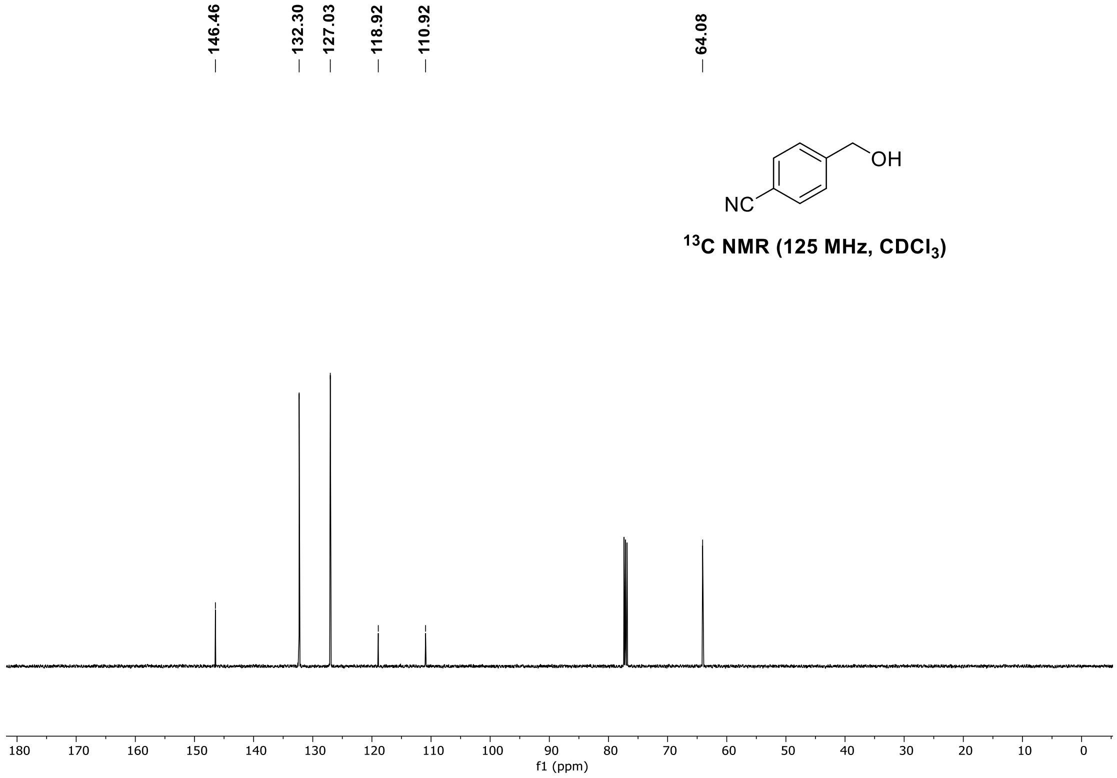

4-(Hydroxymethyl)benzaldehyde (5)

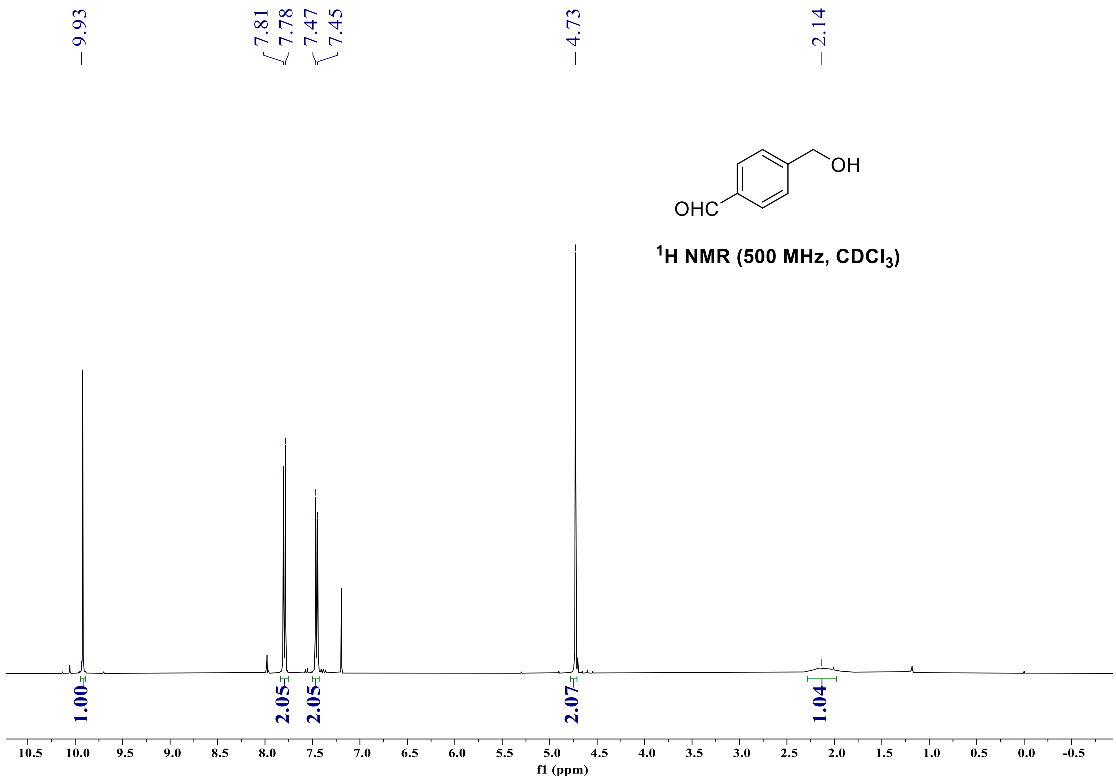

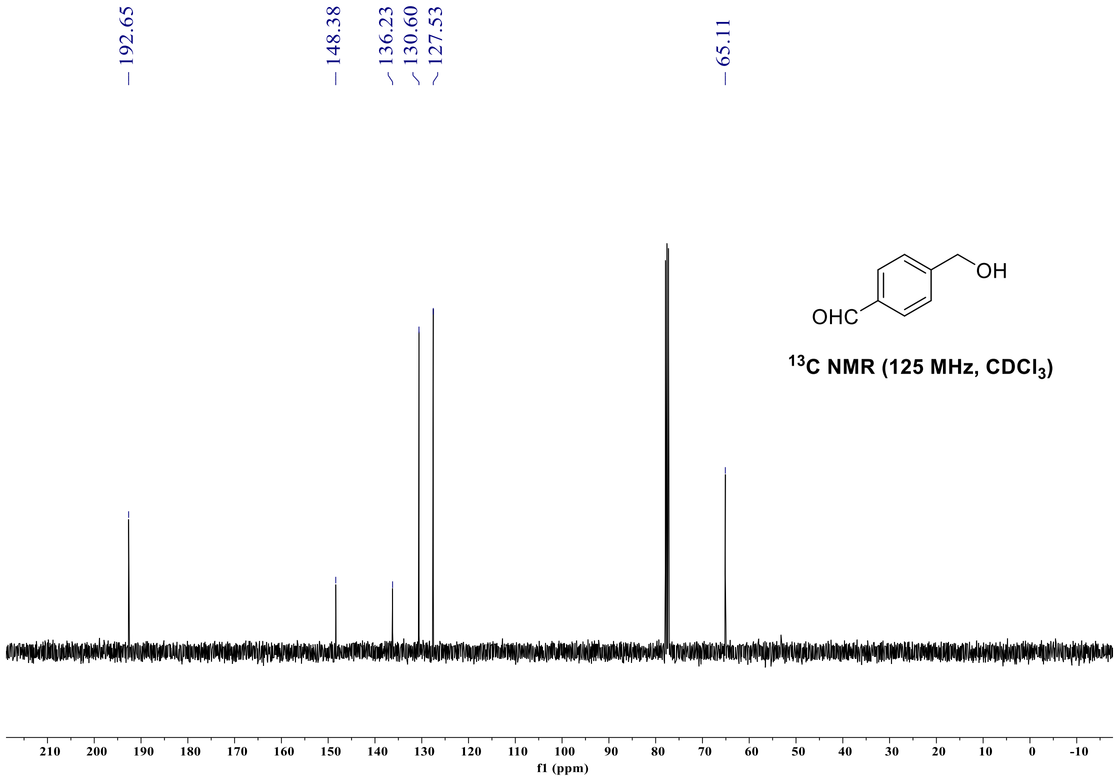

(4-(Methylsulfonyl)phenyl)methanol (6)

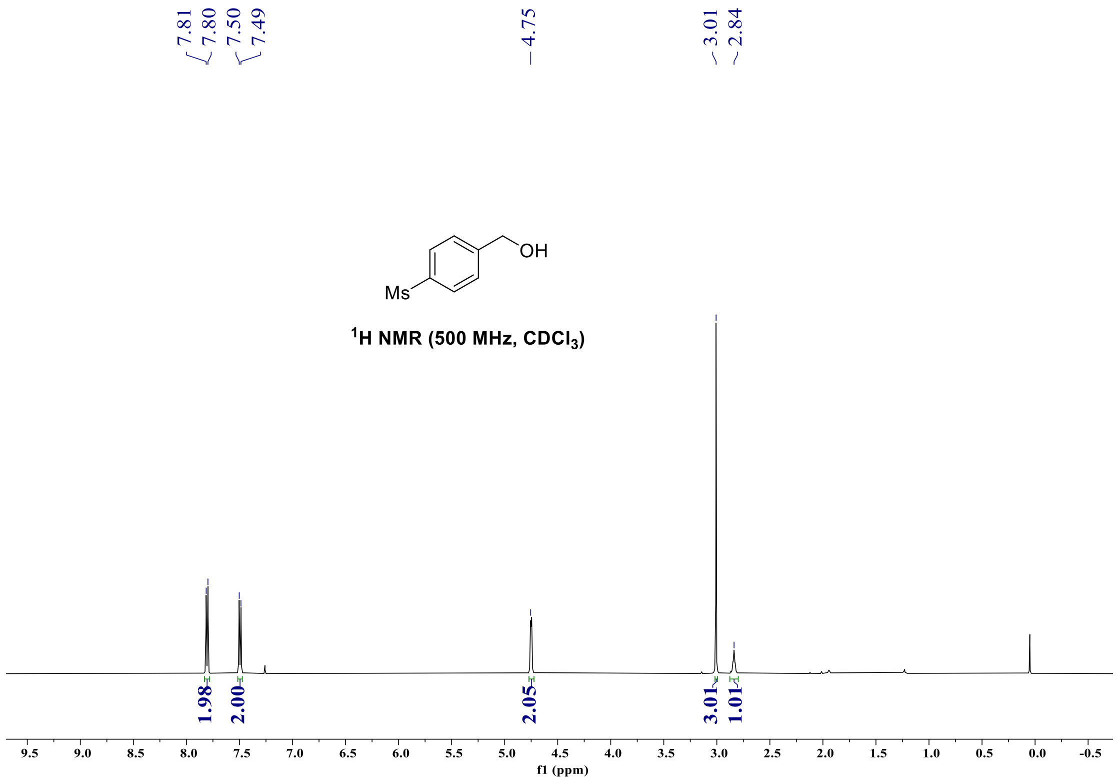

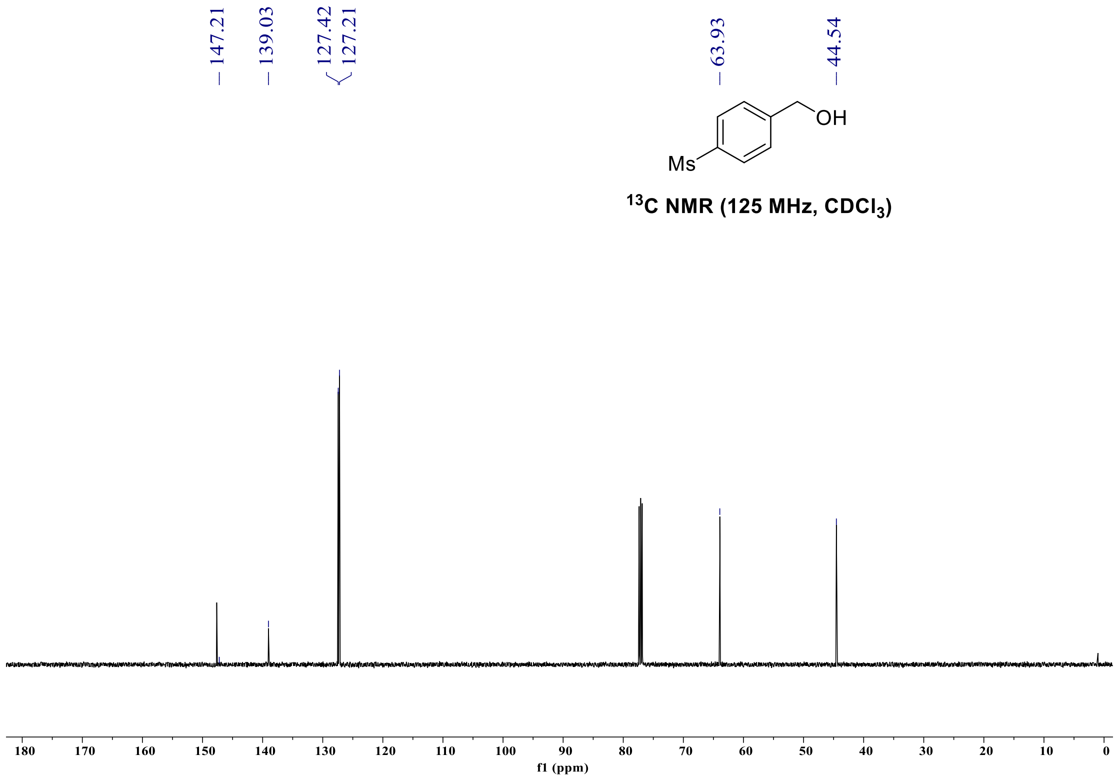

Methyl 4-(hydroxymethyl)benzoate (7)

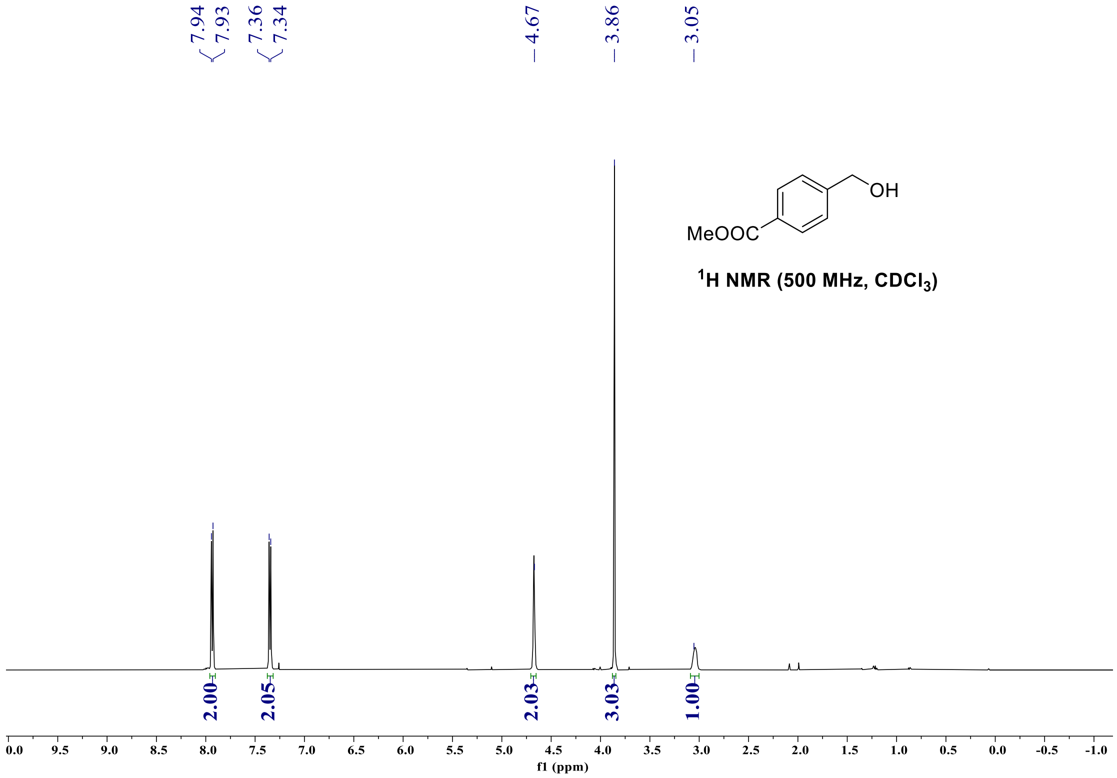

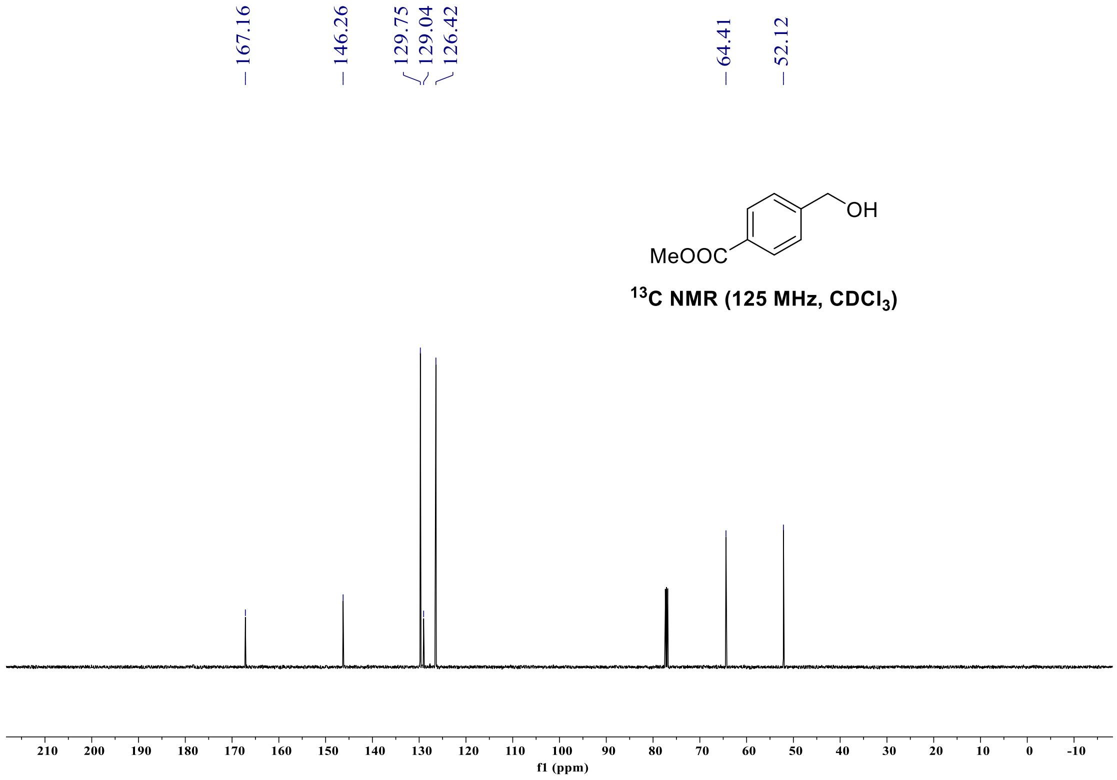

(4-(Trifluoromethyl)phenyl)methanol (8)

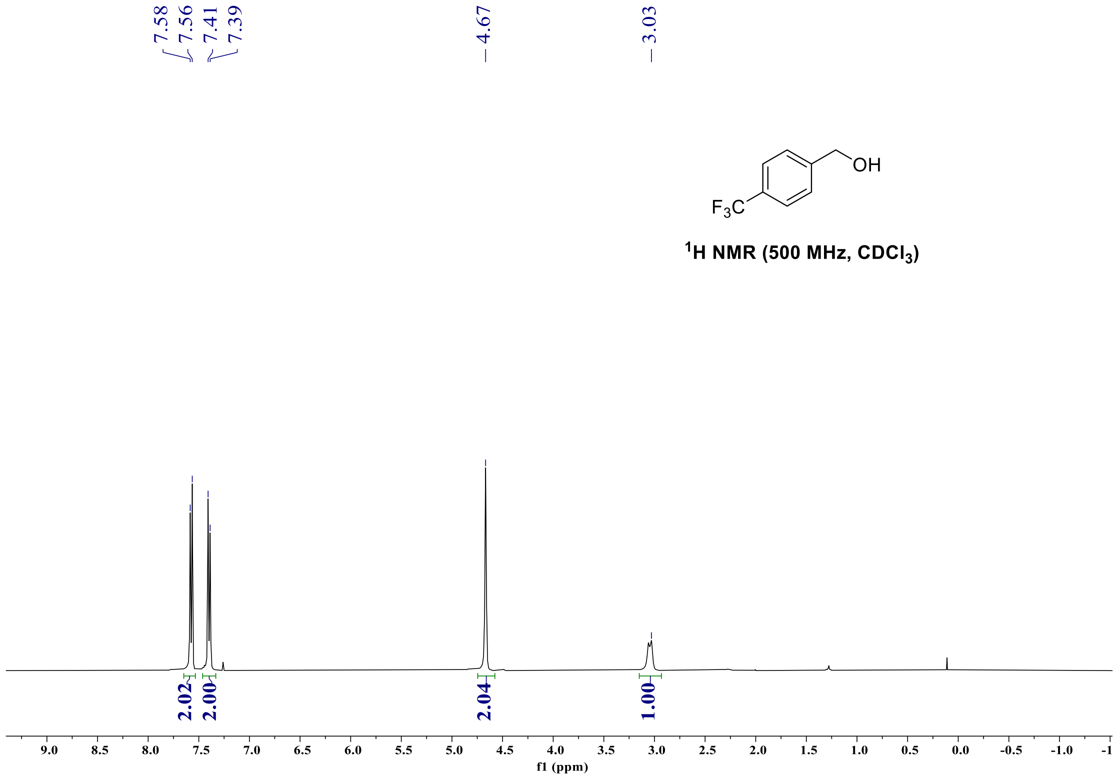

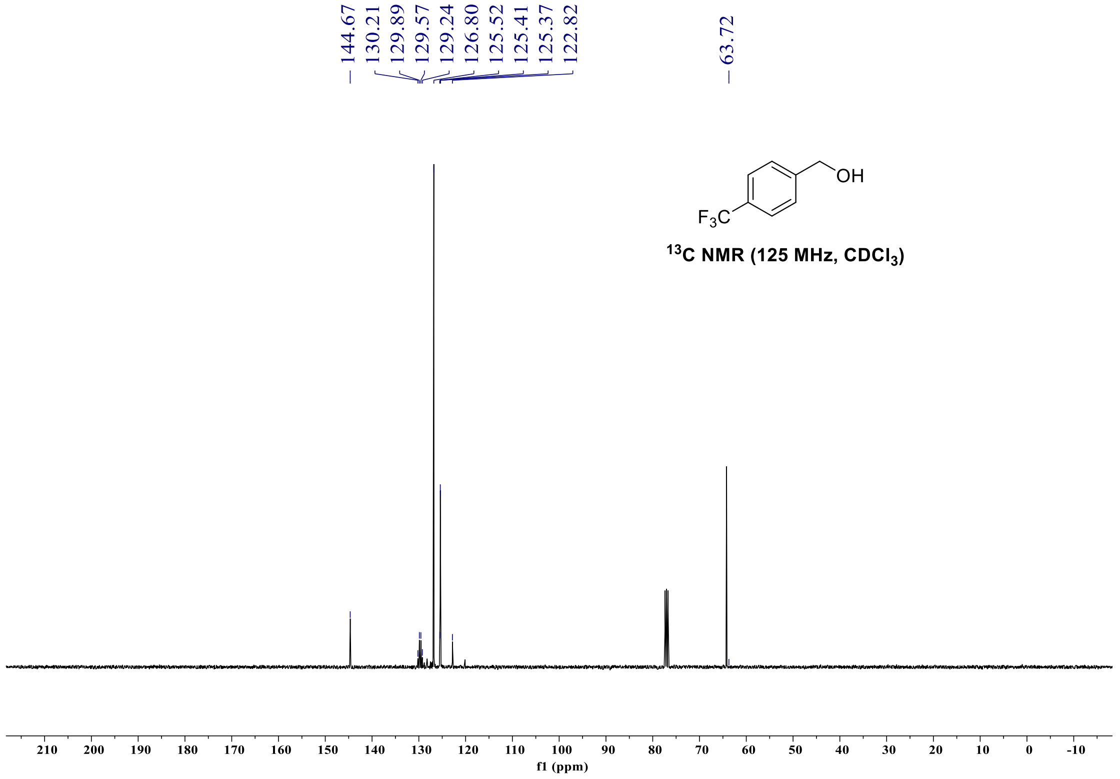

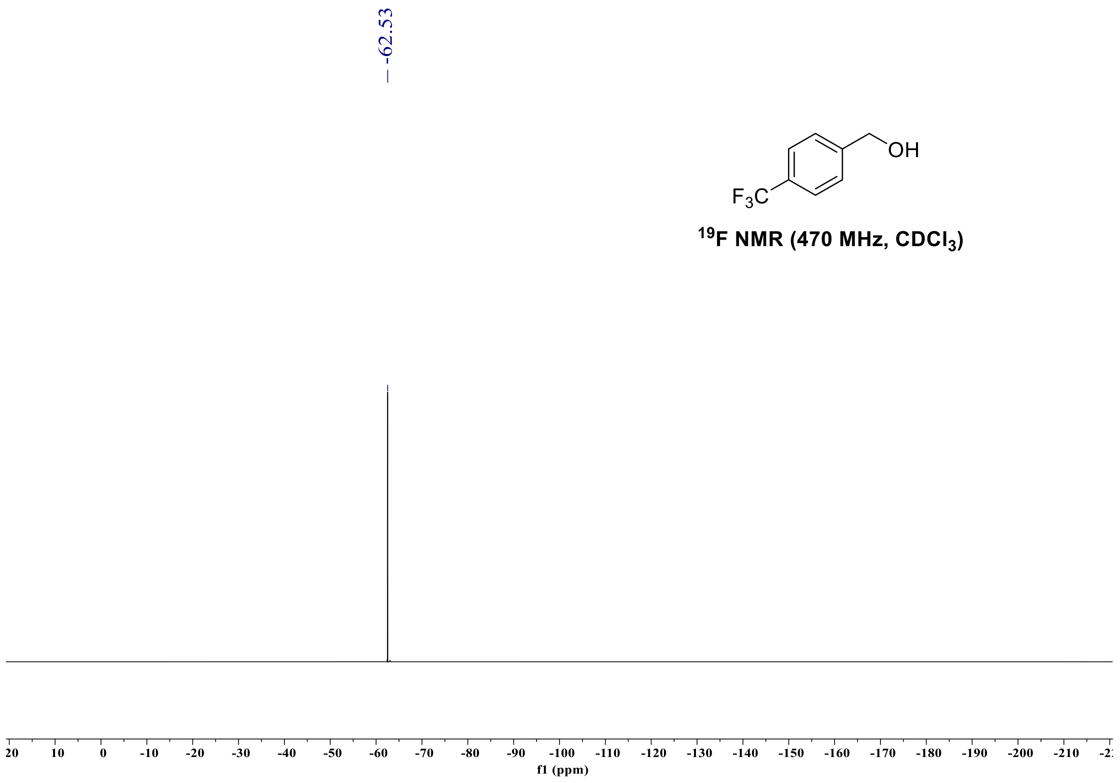

(4-fluorophenyl)methanol (9)

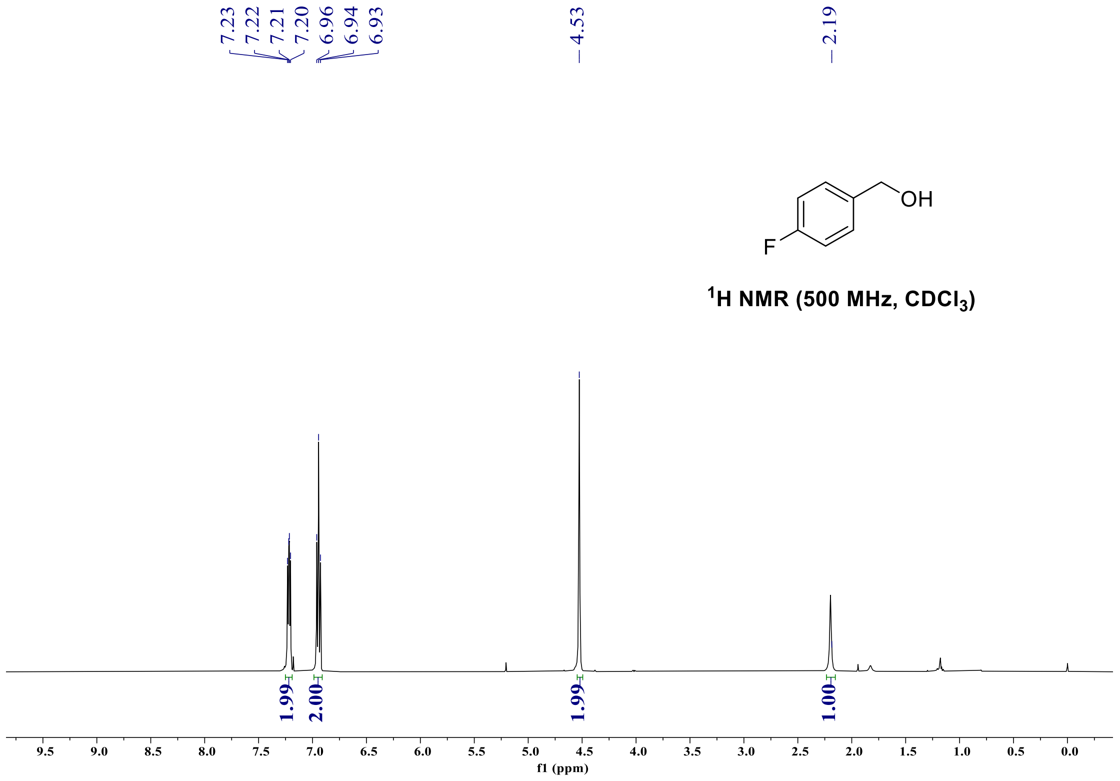

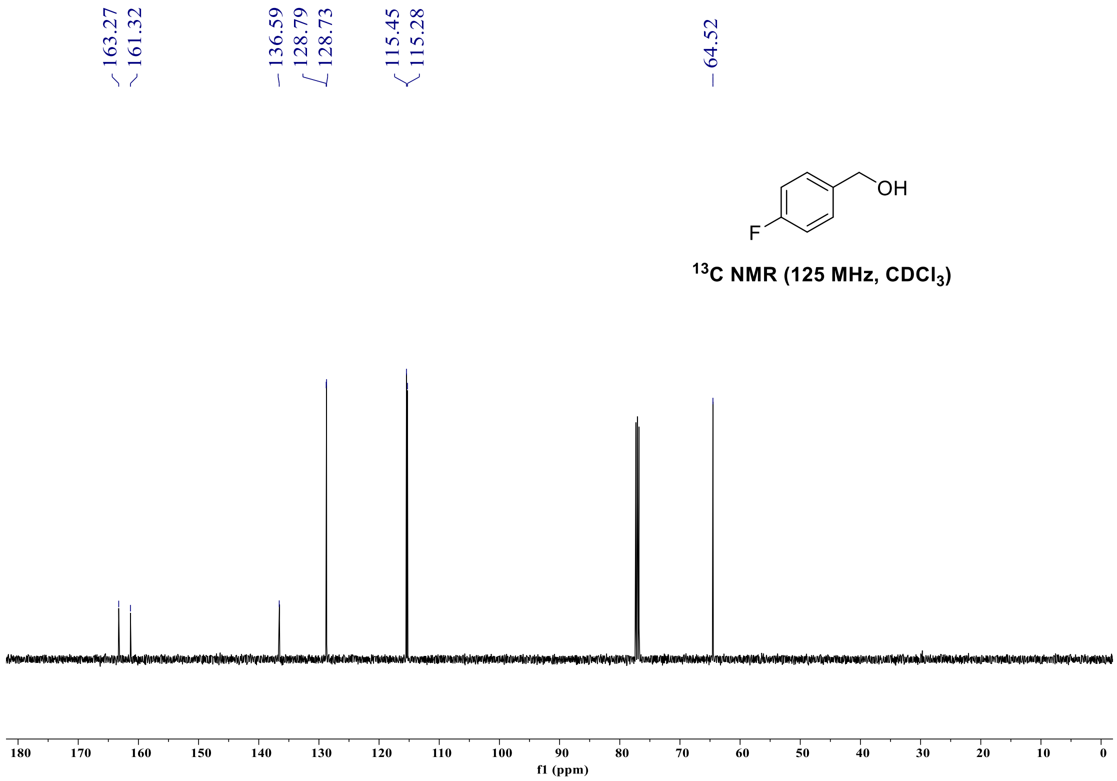
163.27
161.32
136.59
128.79
128.73
115.45
115.28
64.52
OH
F
^{13}C NMR (125 MHz, $CDCl_3$)
180 170 160 150 140 130 120 110 100 90 80 70 60 50 40 30 20 10 0
f1 (ppm)

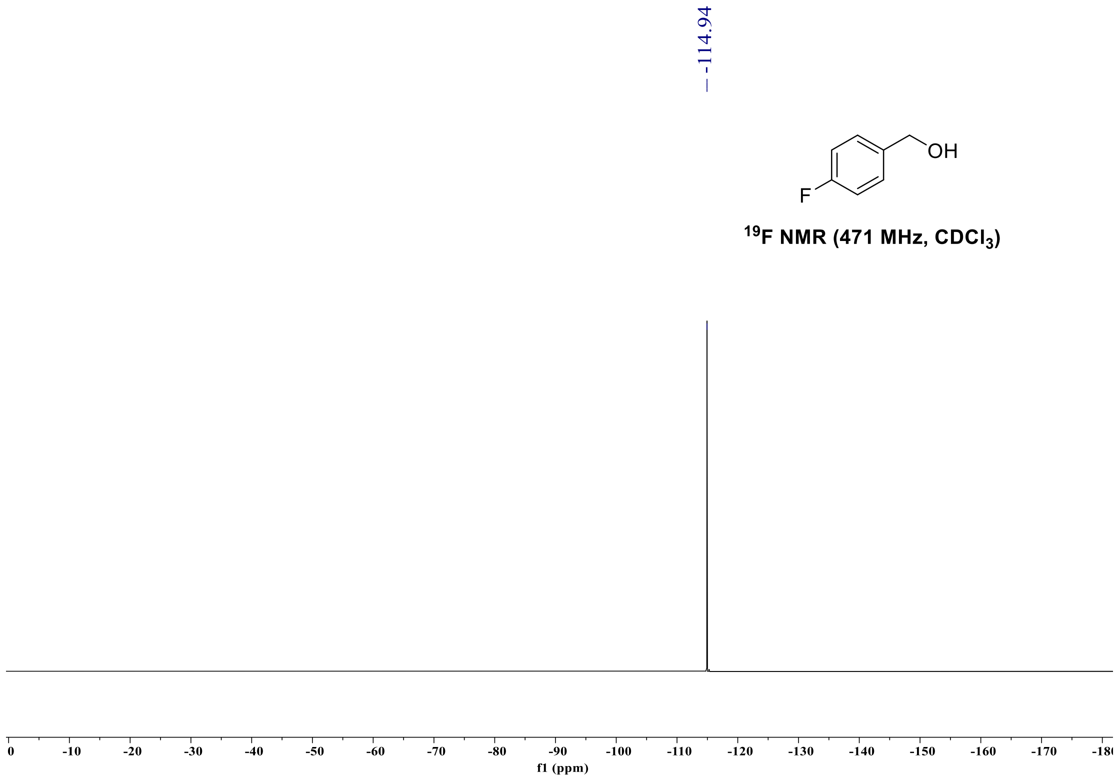
-114.94
OH
F
^{19}F NMR (471 MHz, $CDCl_3$)
0 -10 -20 -30 -40 -50 -60 -70 -80 -90 -100 -110 -120 -130 -140 -150 -160 -170
f1 (ppm)

(4-Chlorophenyl)methanol (10)

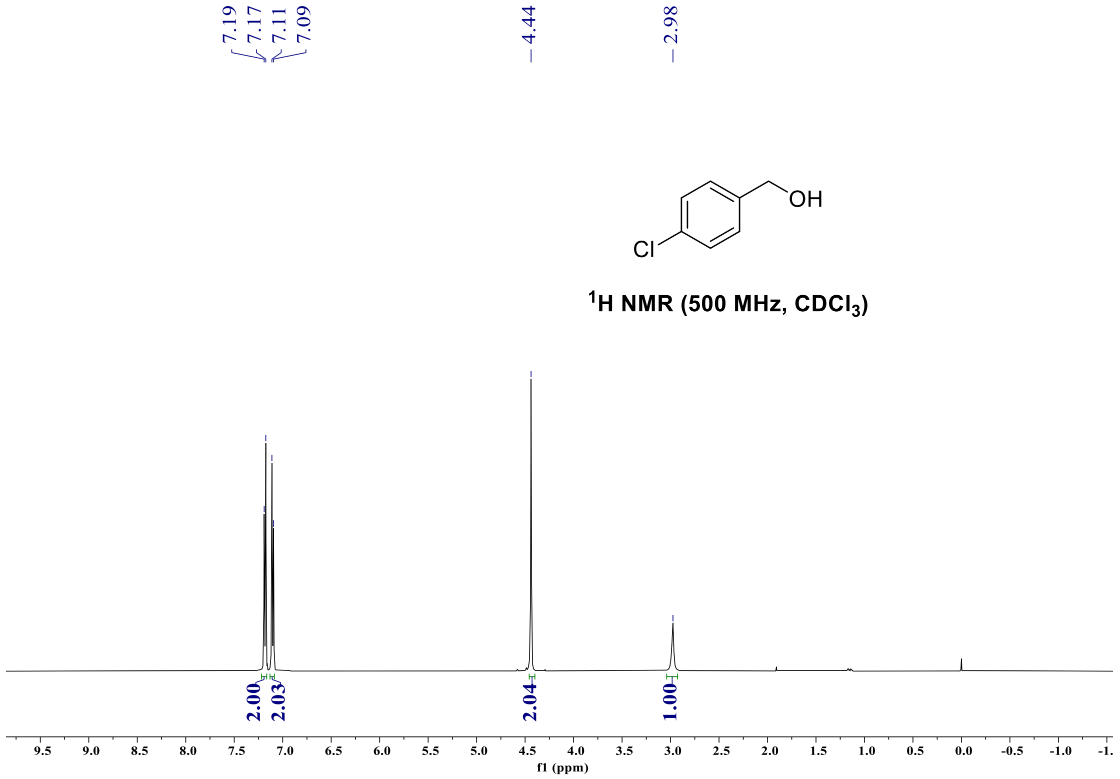

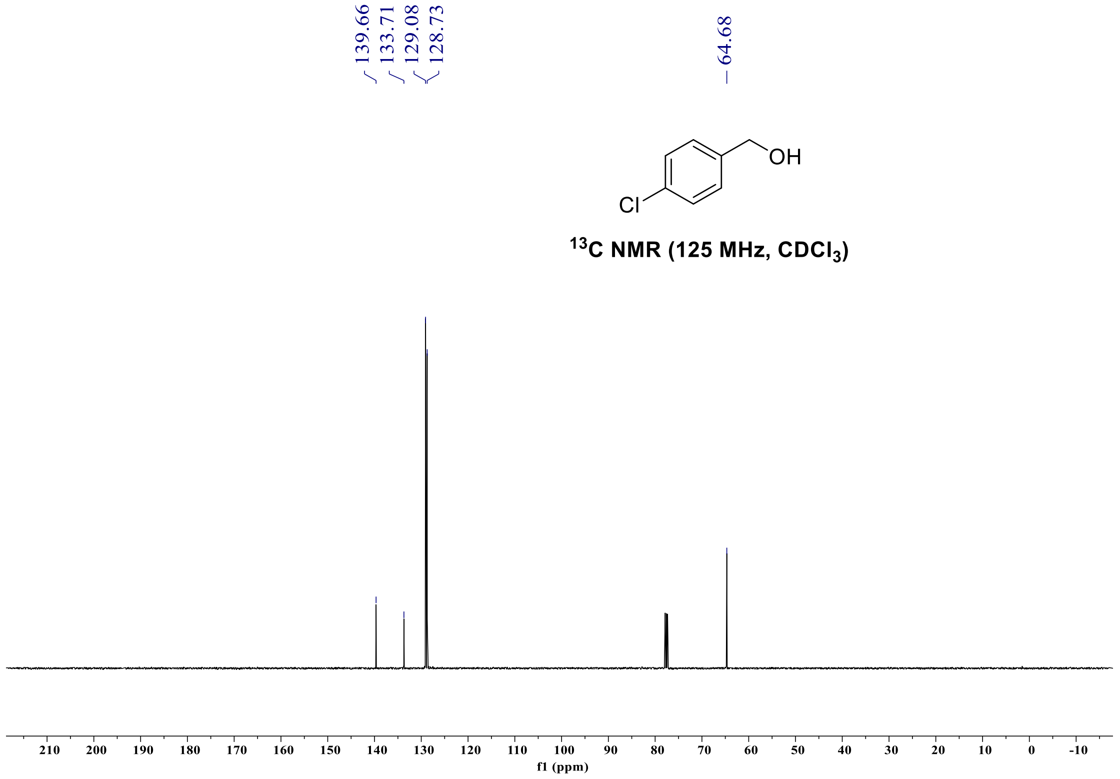

(4-Ethynylphenyl)methanol (11)

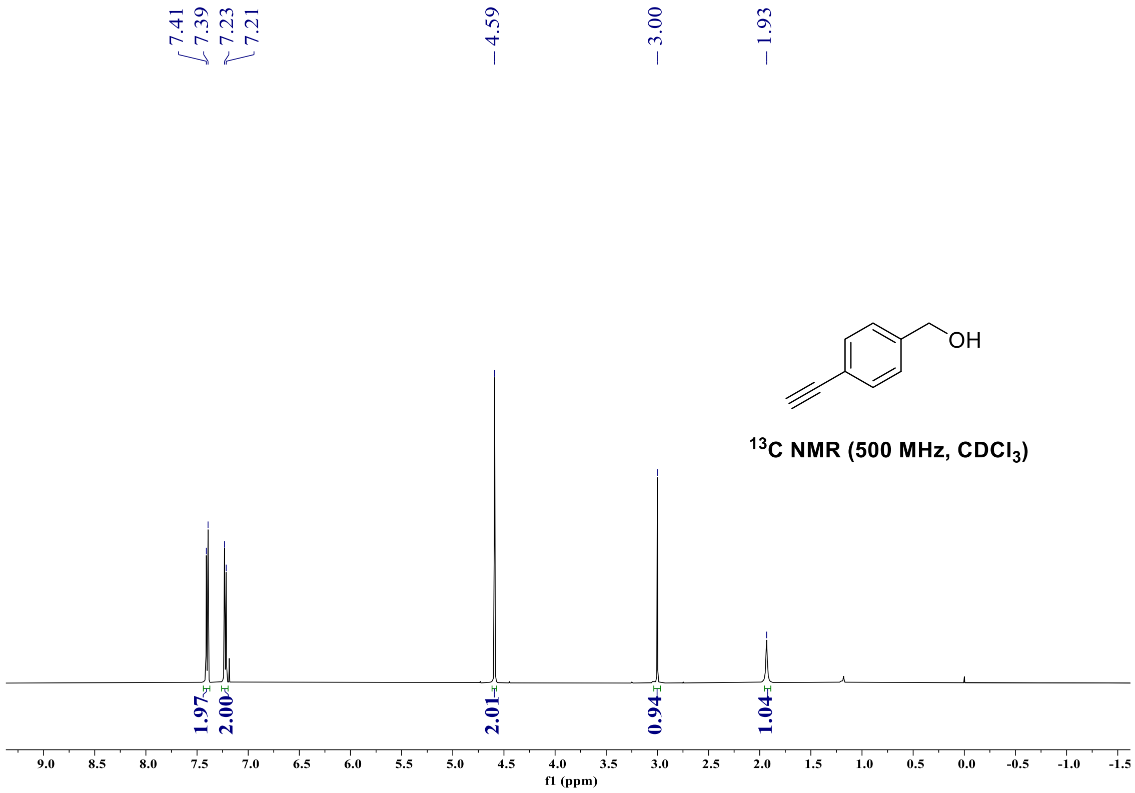

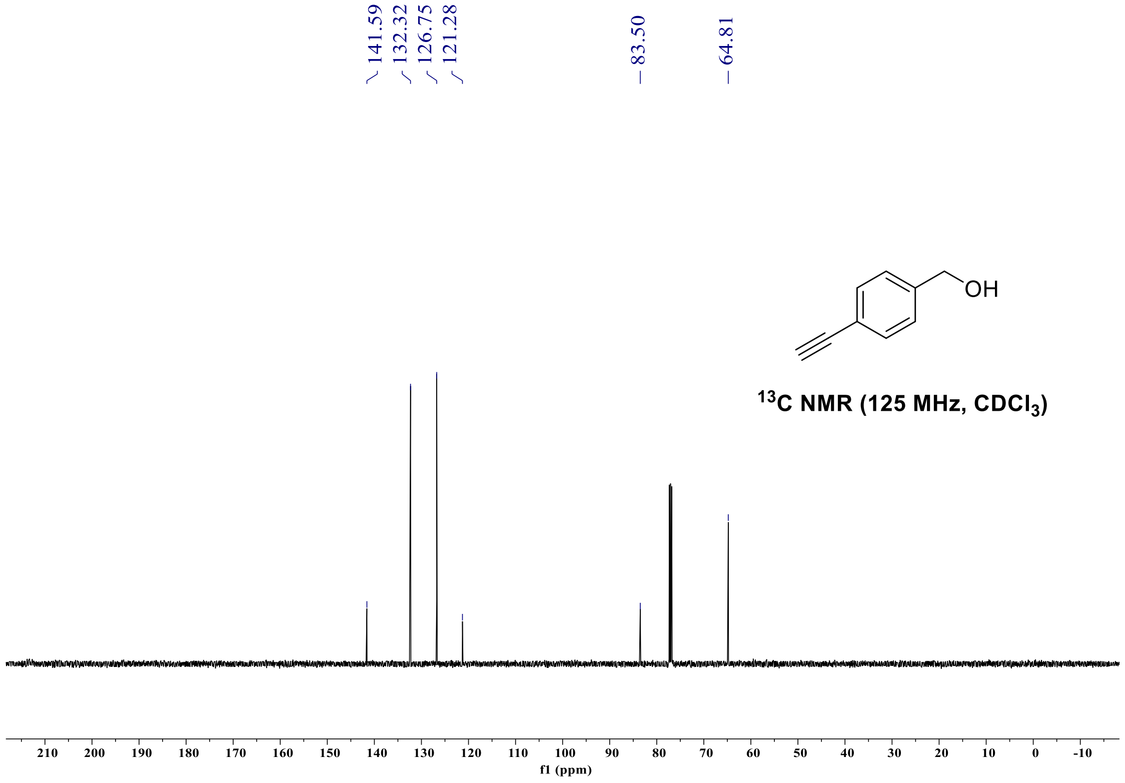

Phenylmethanol (12)

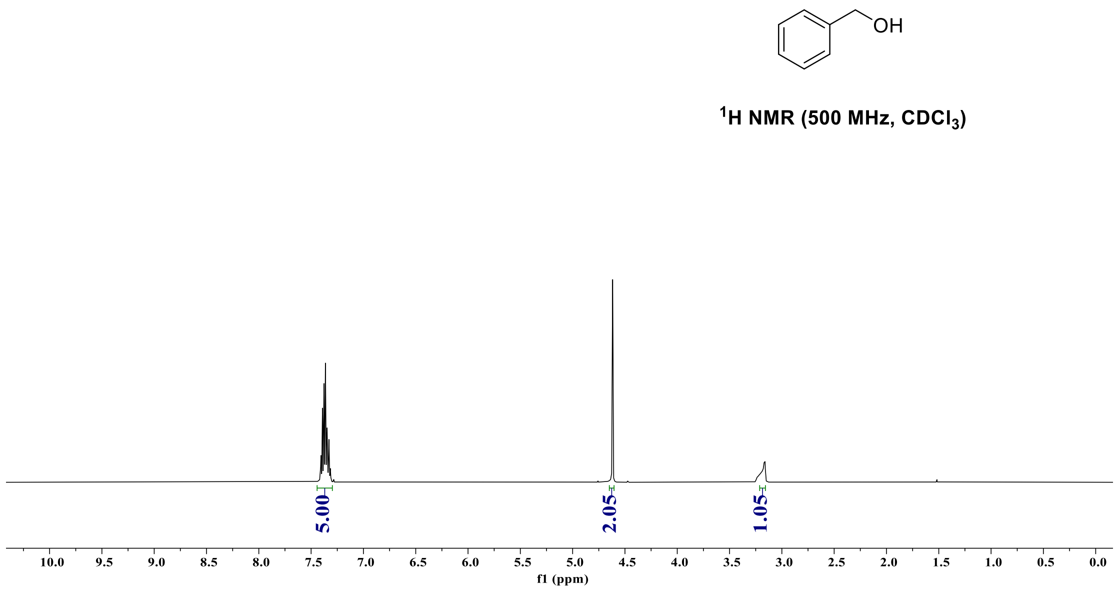

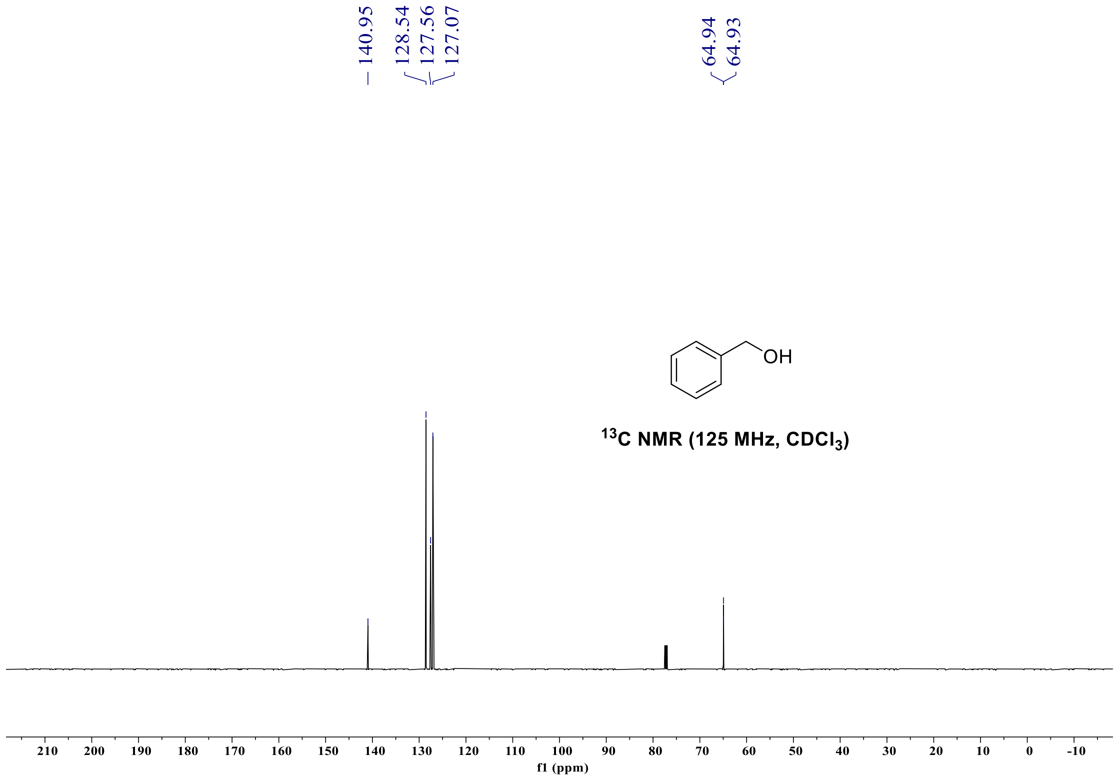

(4-Methoxyphenyl)methanol (13)

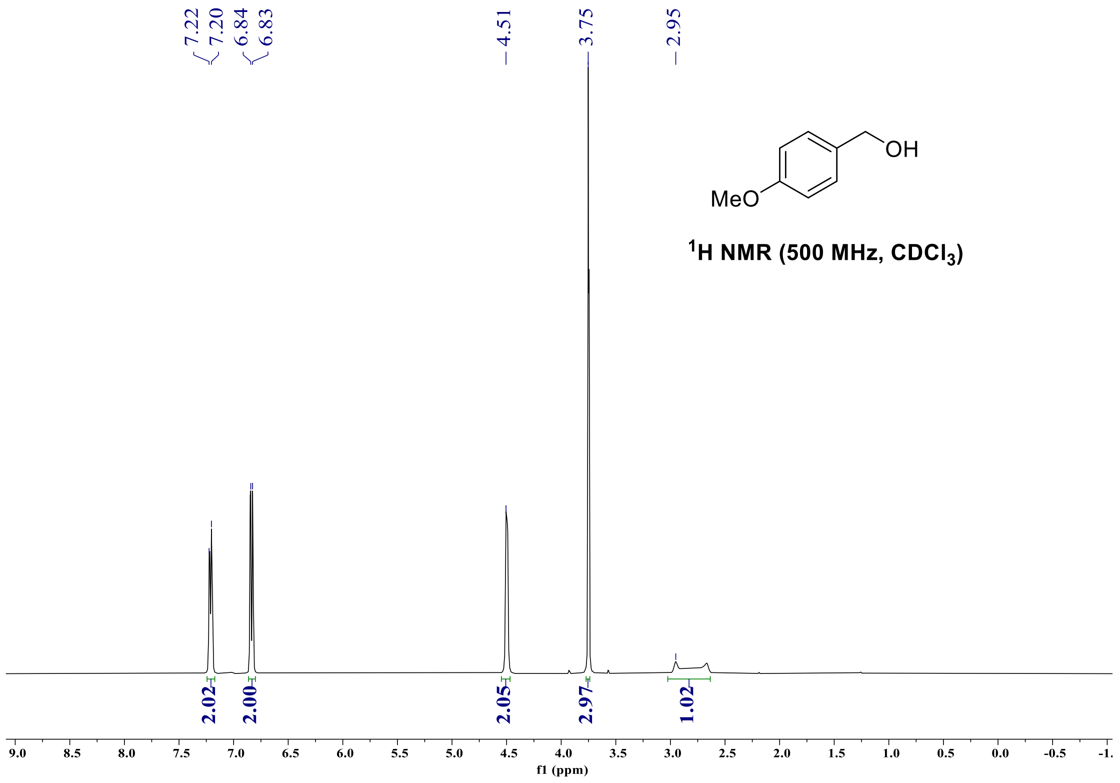

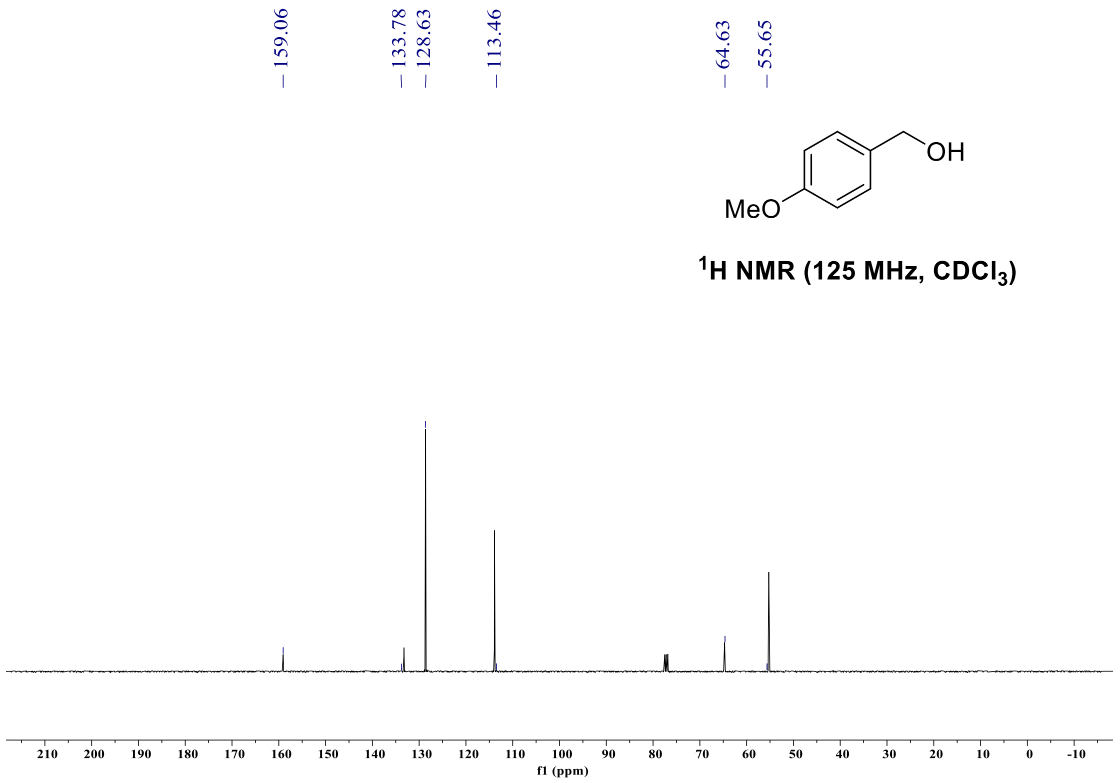

(4-(Benzyloxy)phenyl)methanol (14)

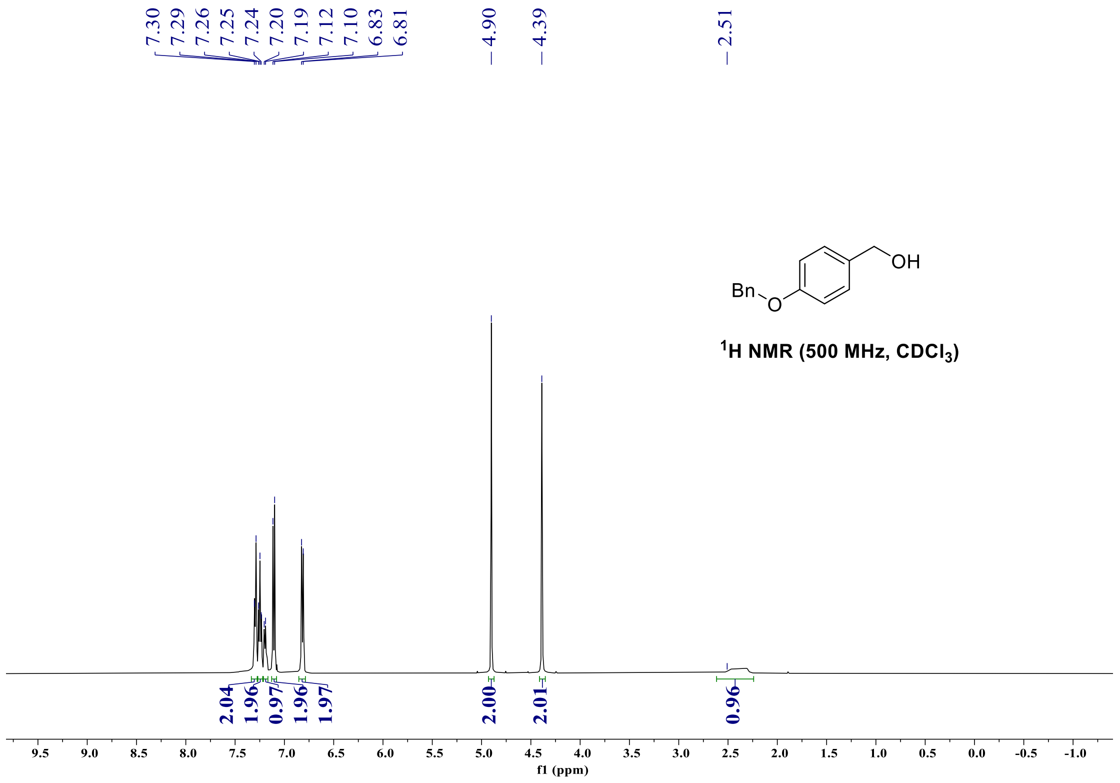

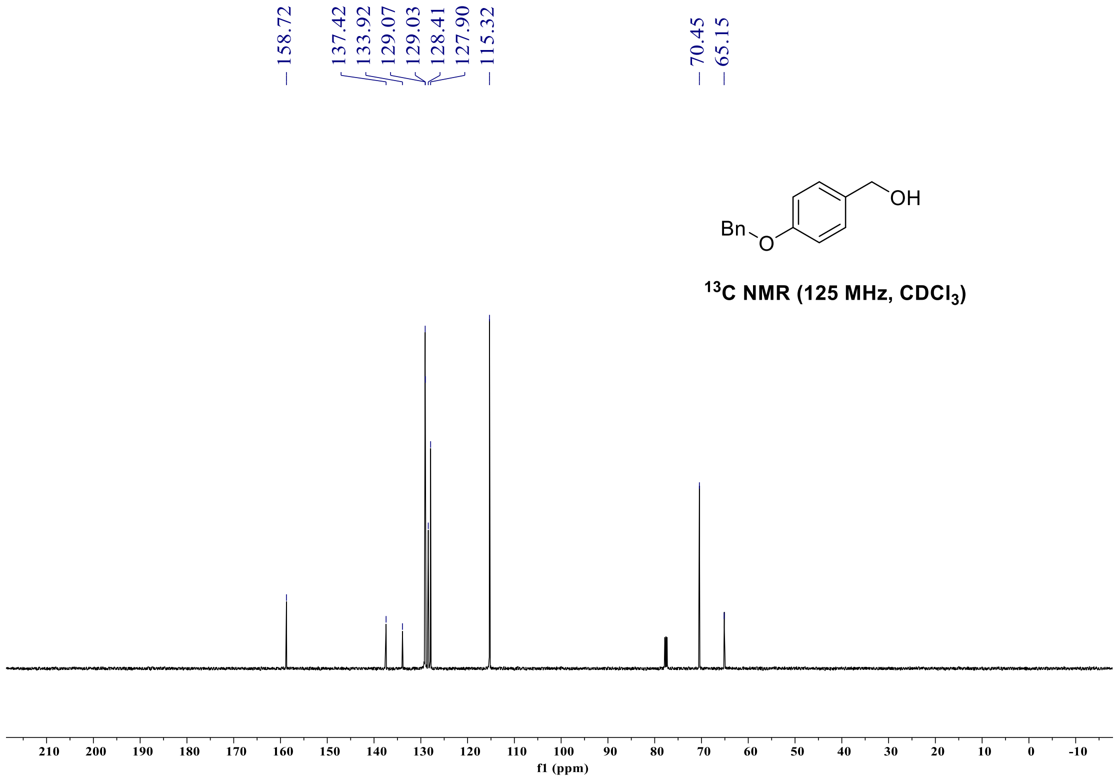

(4-(Dimethylamino)phenyl)methanol (15)

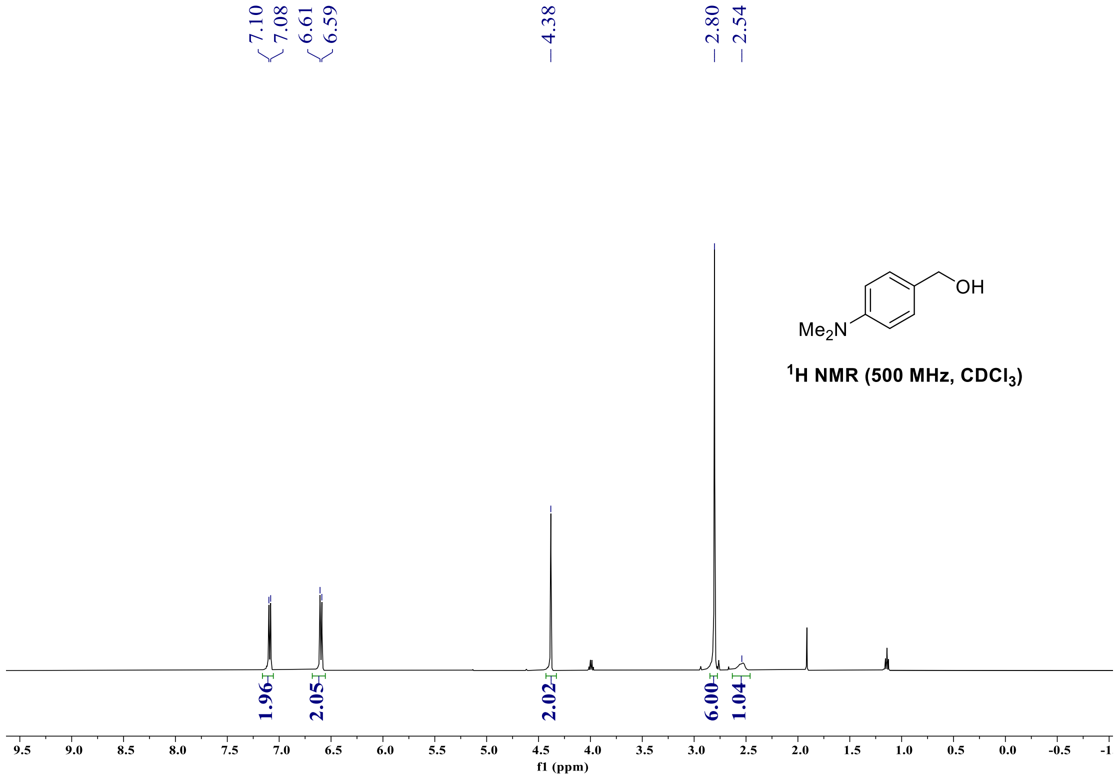

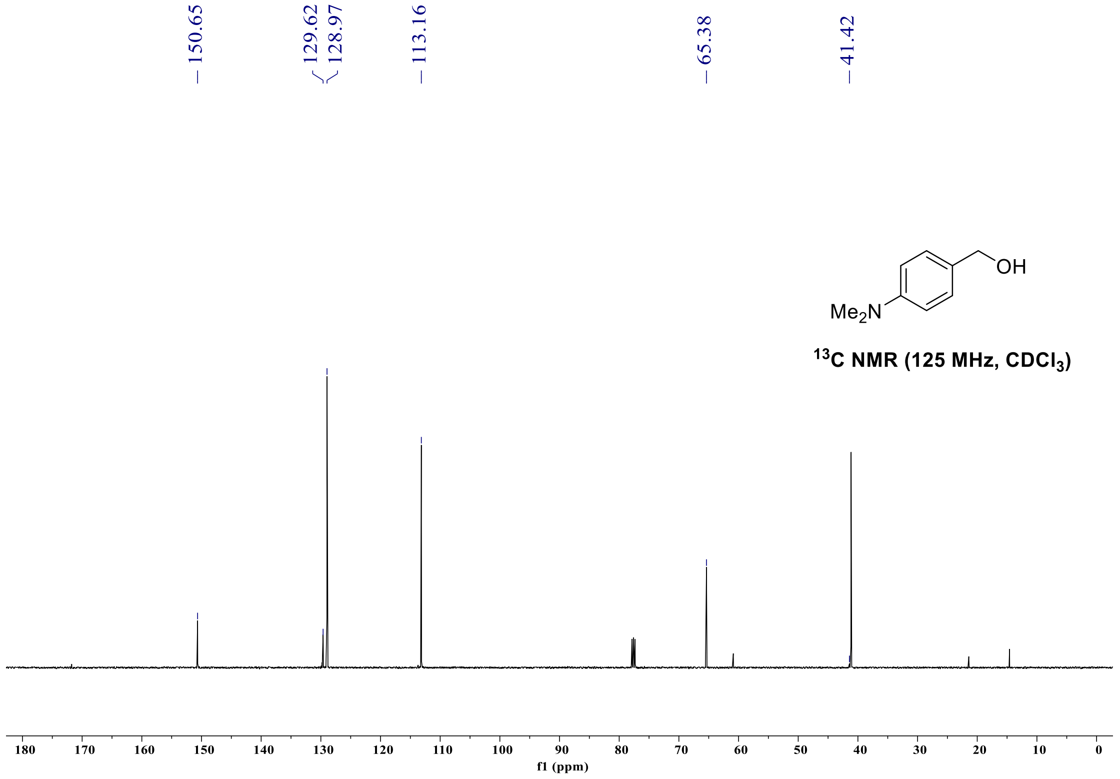

N-(4-(hydroxymethyl)phenyl)acetamide (16)

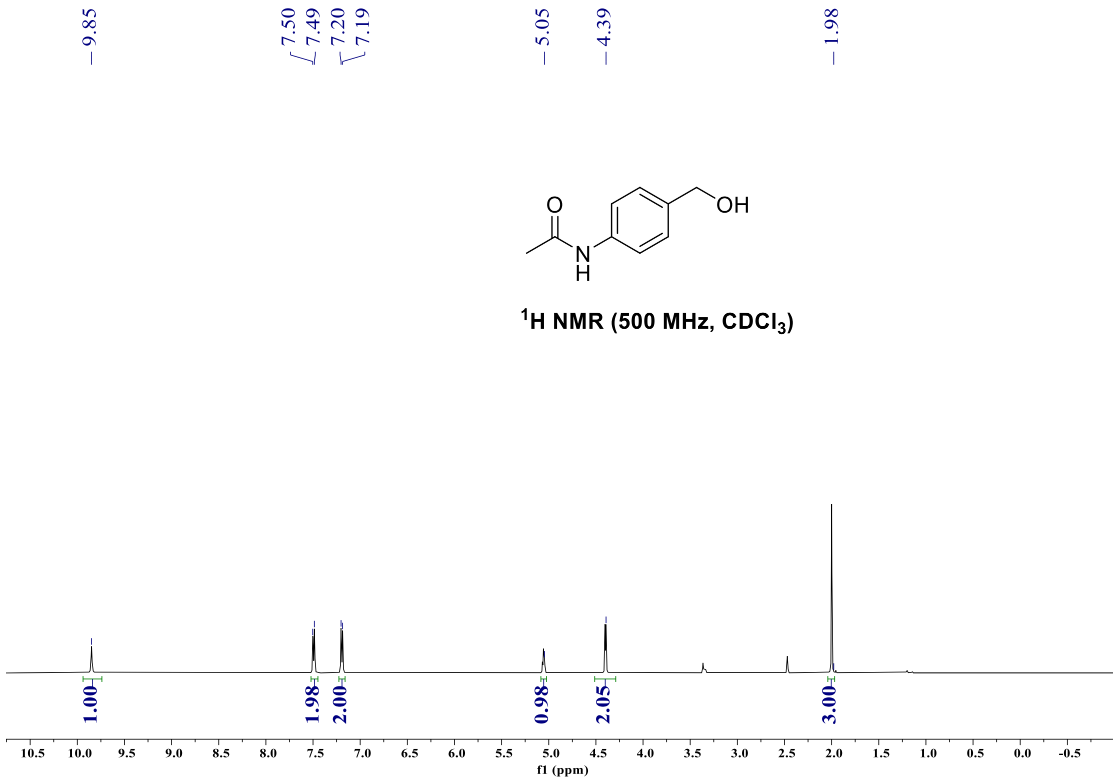

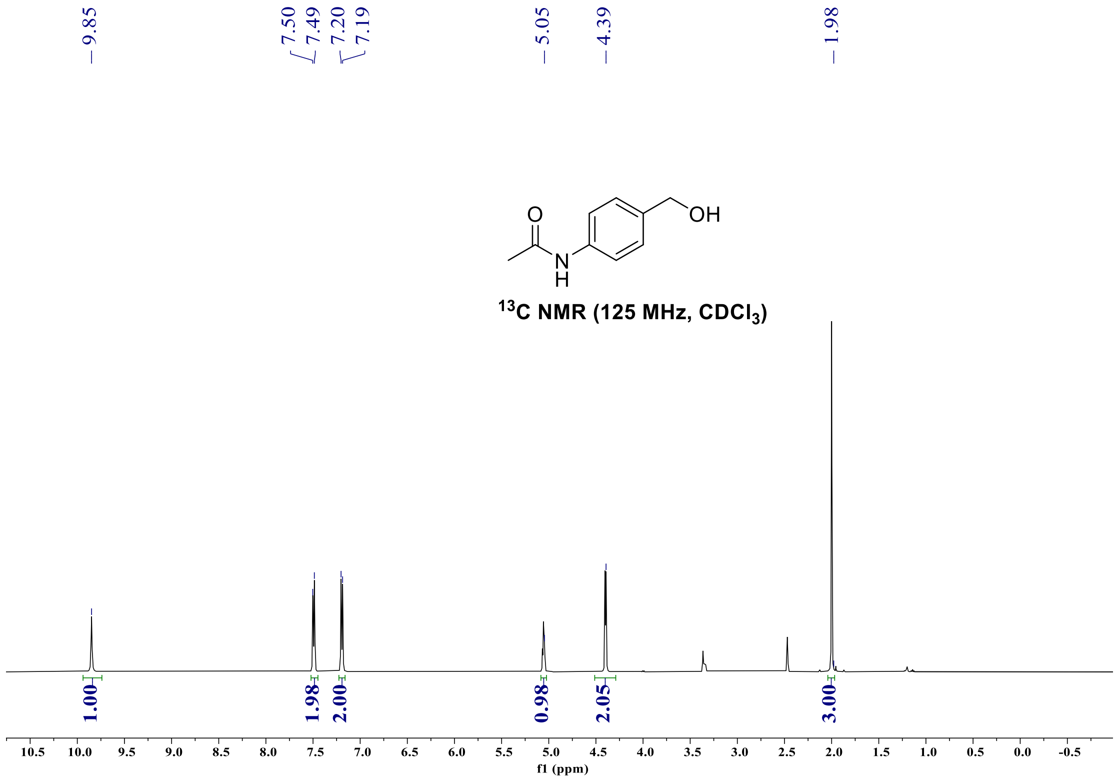

2,2,2-Trifluoro-*N*-(4-(hydroxymethyl)phenyl)acetamide (17)

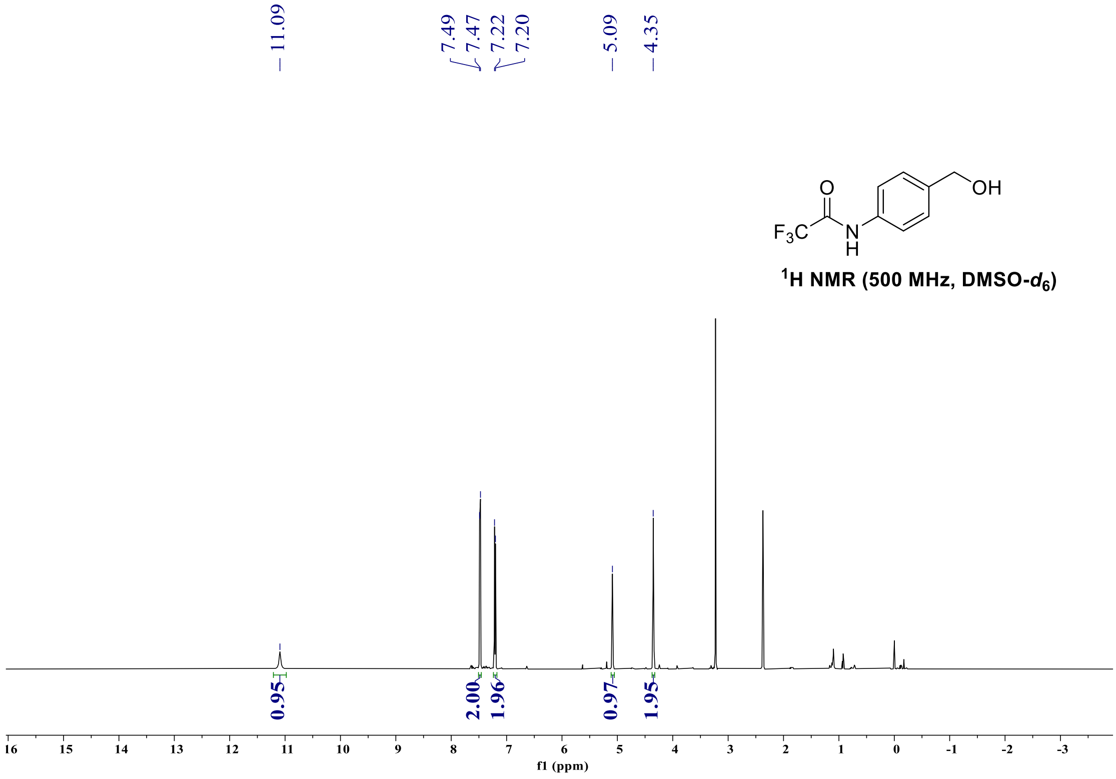

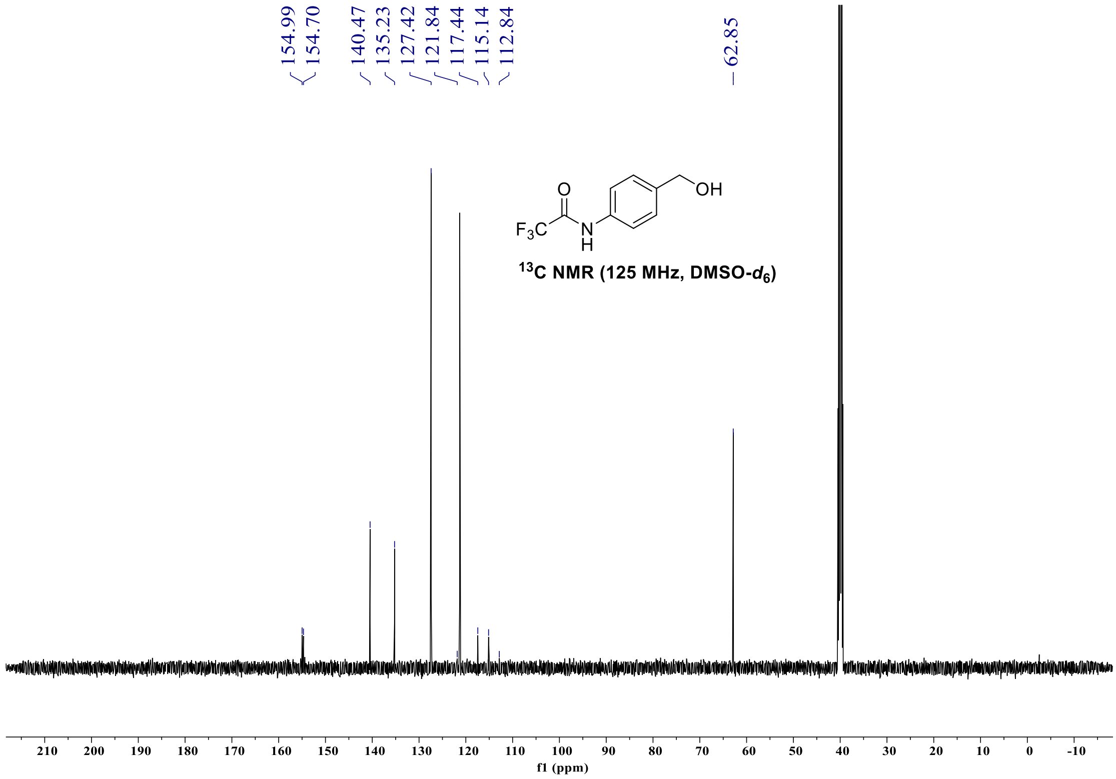

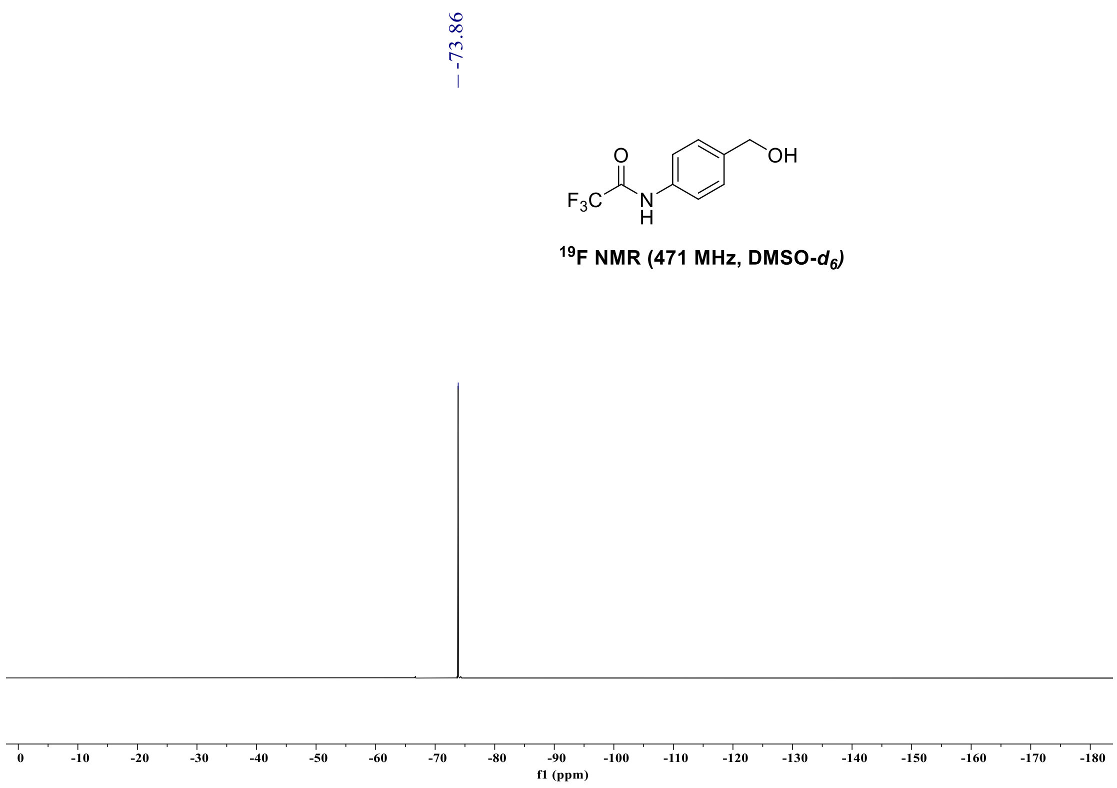

4-(Hydroxymethyl)phenyl acetate (18)

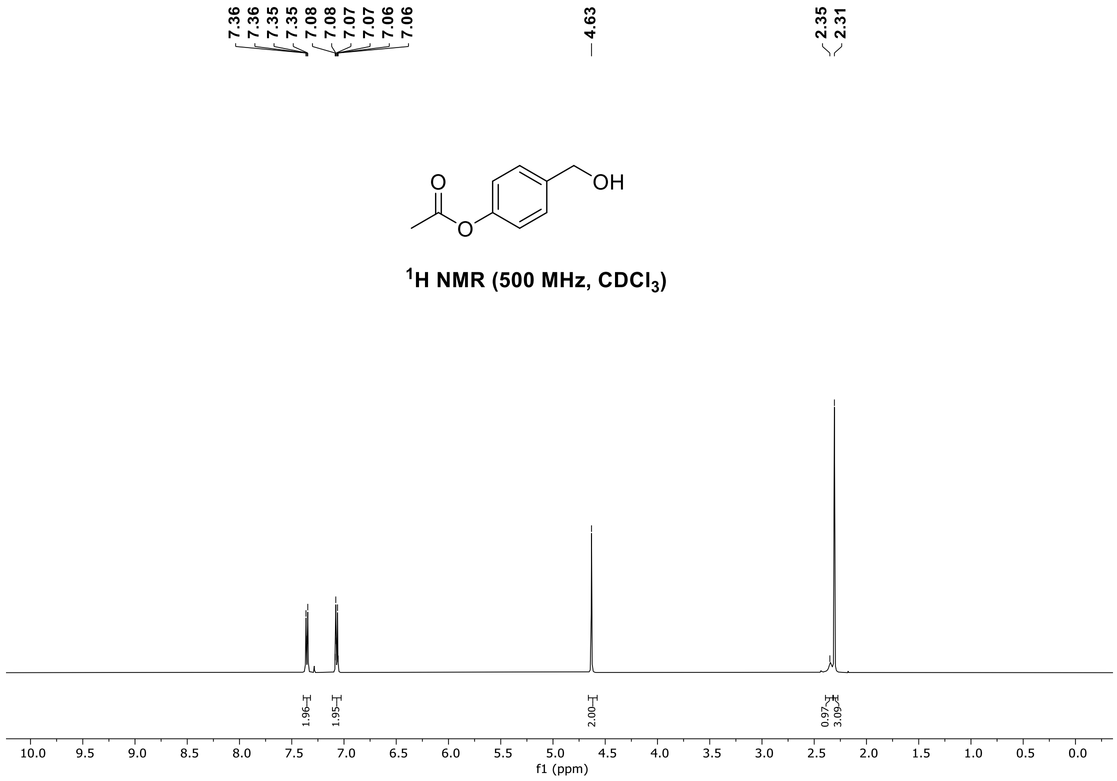

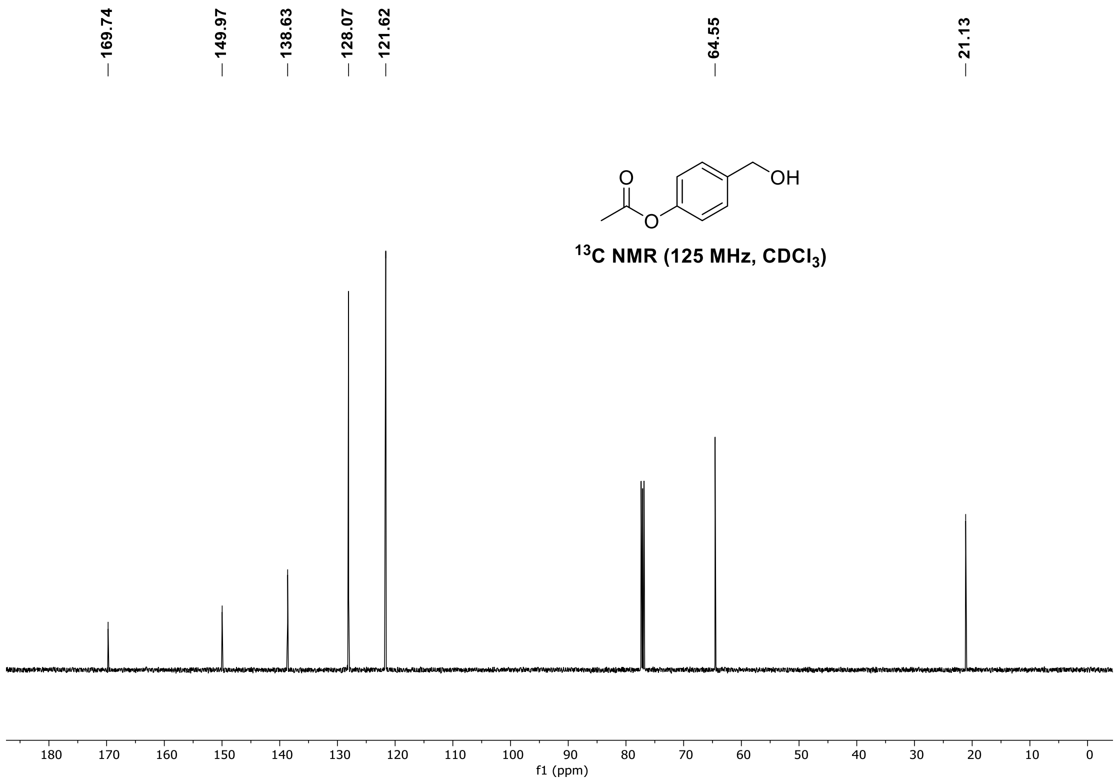

4-(hydroxymethyl)phenyl trifluoromethanesulfonate (19)

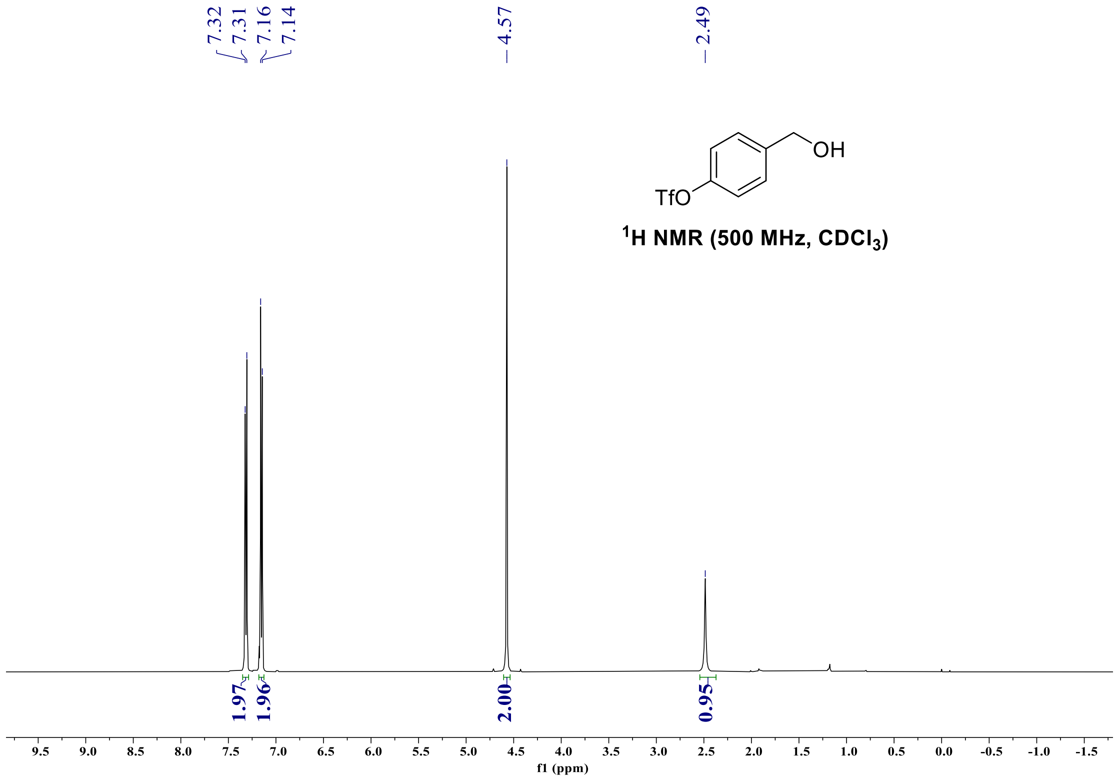

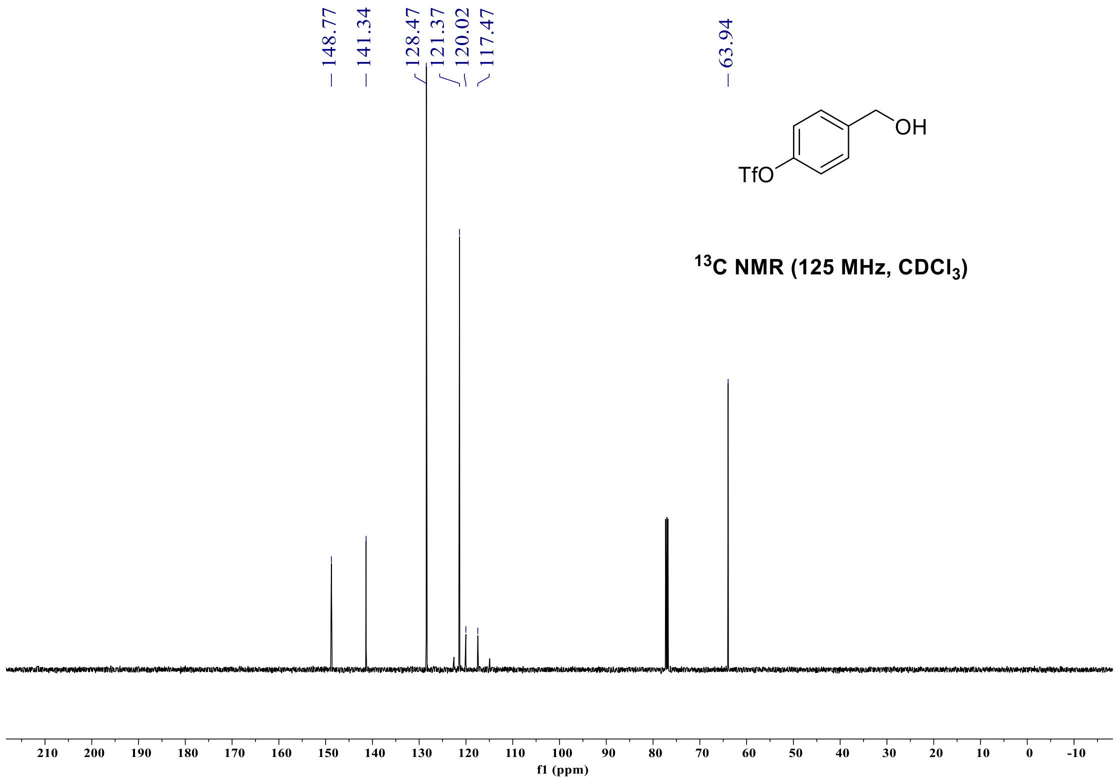
148.77
141.34
128.47
121.37
120.02
117.47
63.94
OH
TfO
13C NMR (125 MHz, CDCl3)
f1 (ppm)

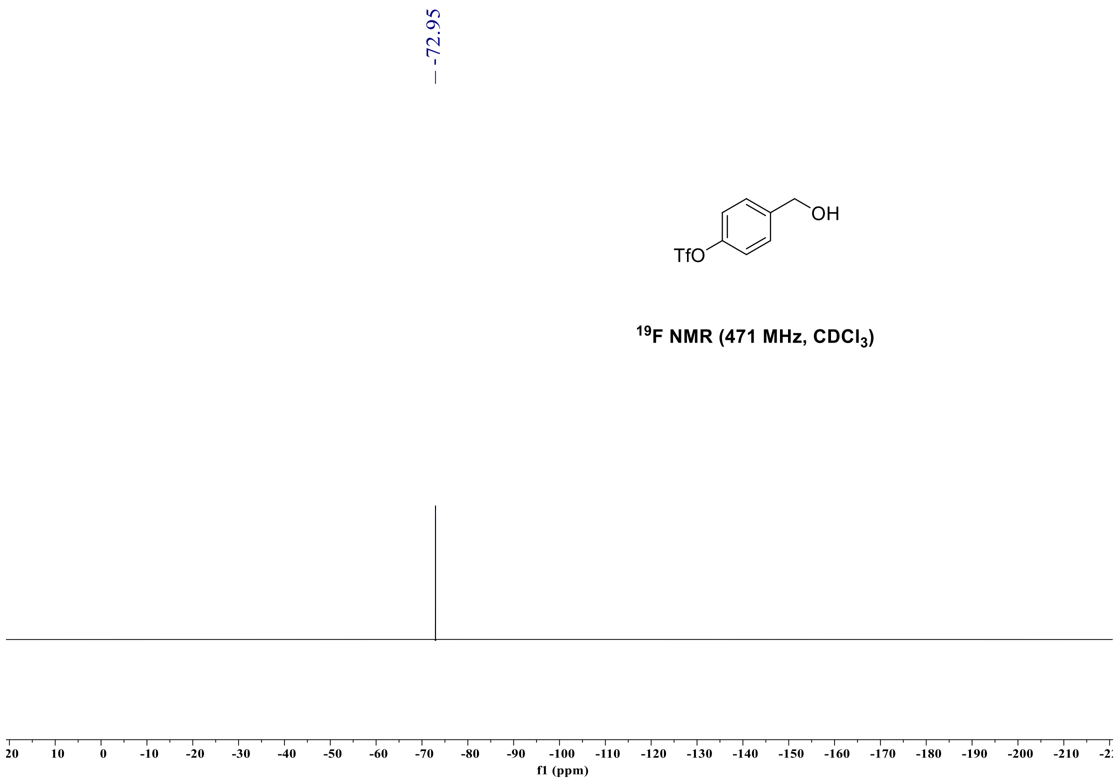
-72.95
OH
TfO
19F NMR (471 MHz, CDCl3)
f1 (ppm)

Dimethyl(phenyl)((4-(4,4,5,5-tetramethyl-1,3,2-dioxaborolan-2-yl)benzyl)oxy)silane (20)

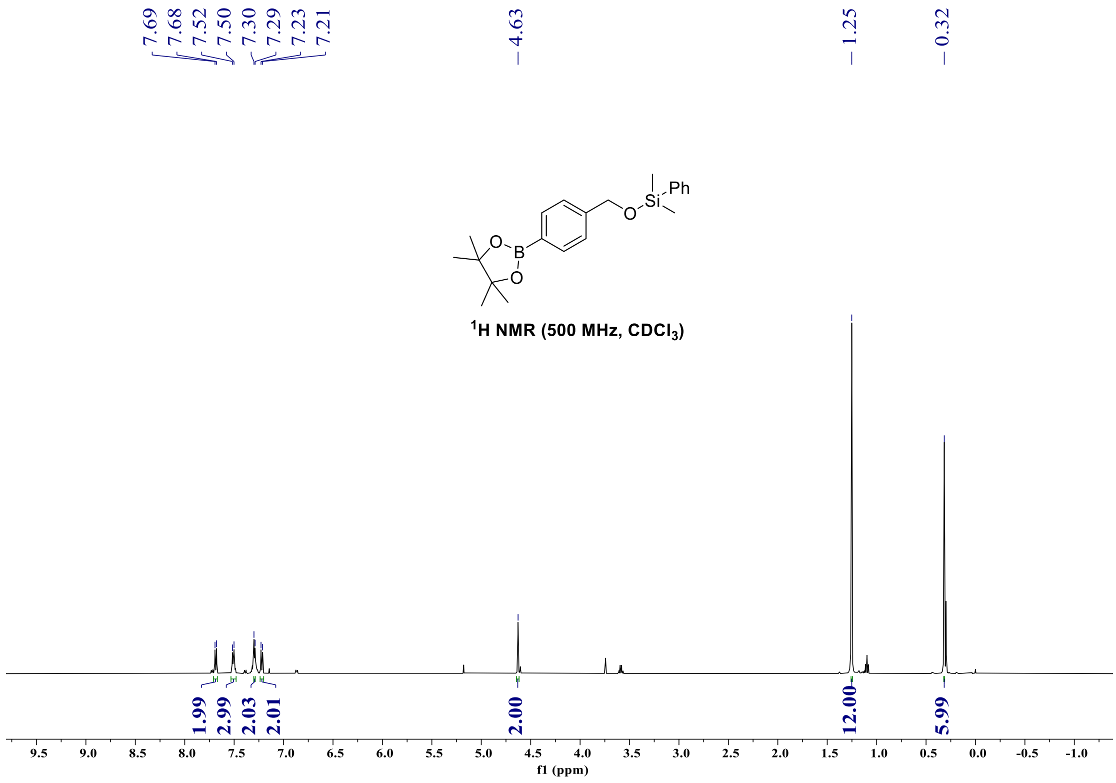

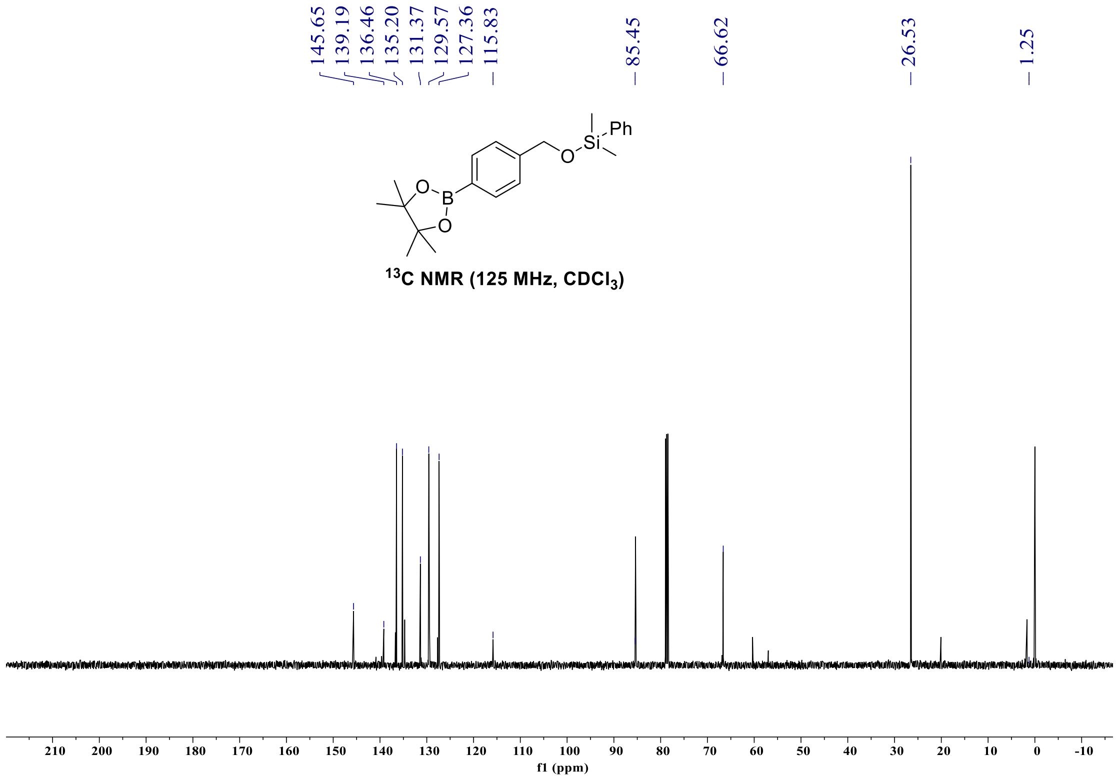

Benzo[*d*][1,3]dioxol-5-ylmethanol (21)

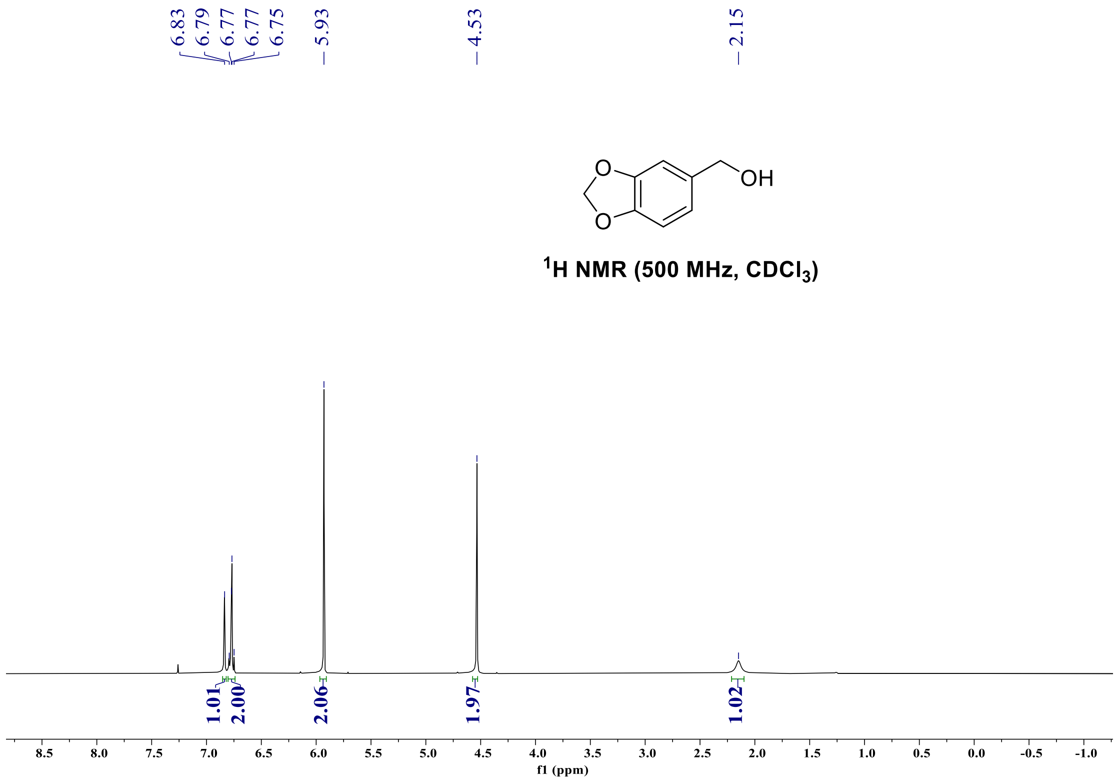

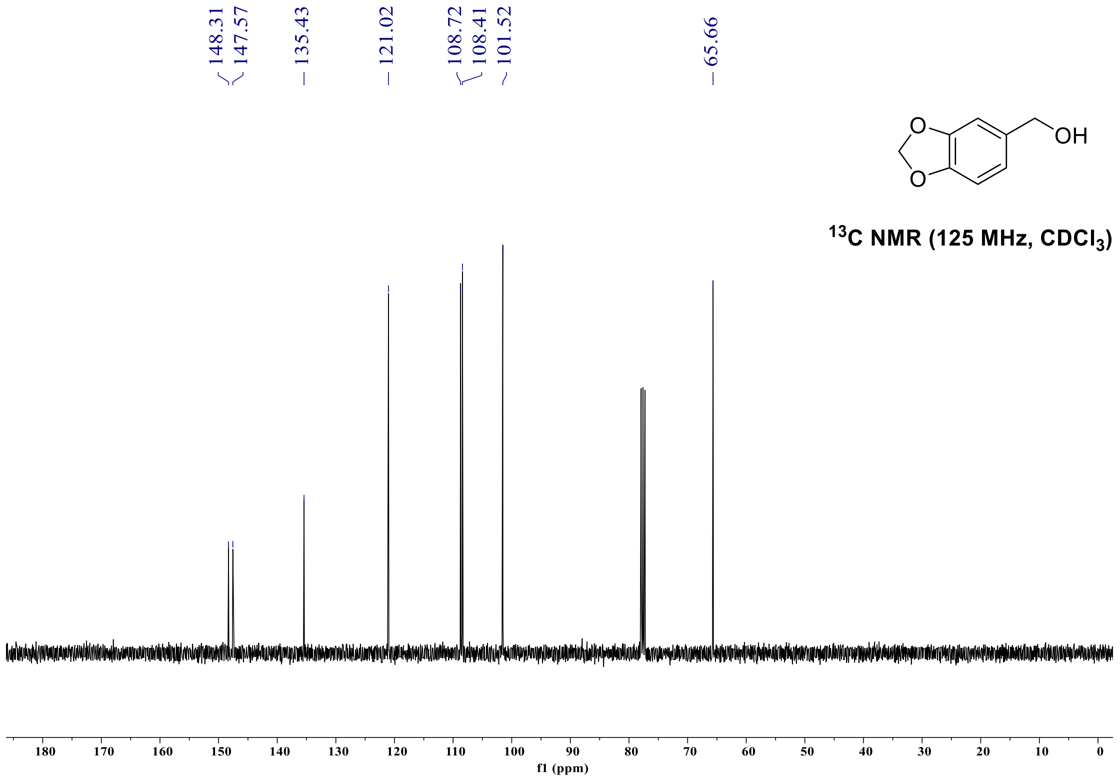

4-(Hydroxymethyl)phenol (22)

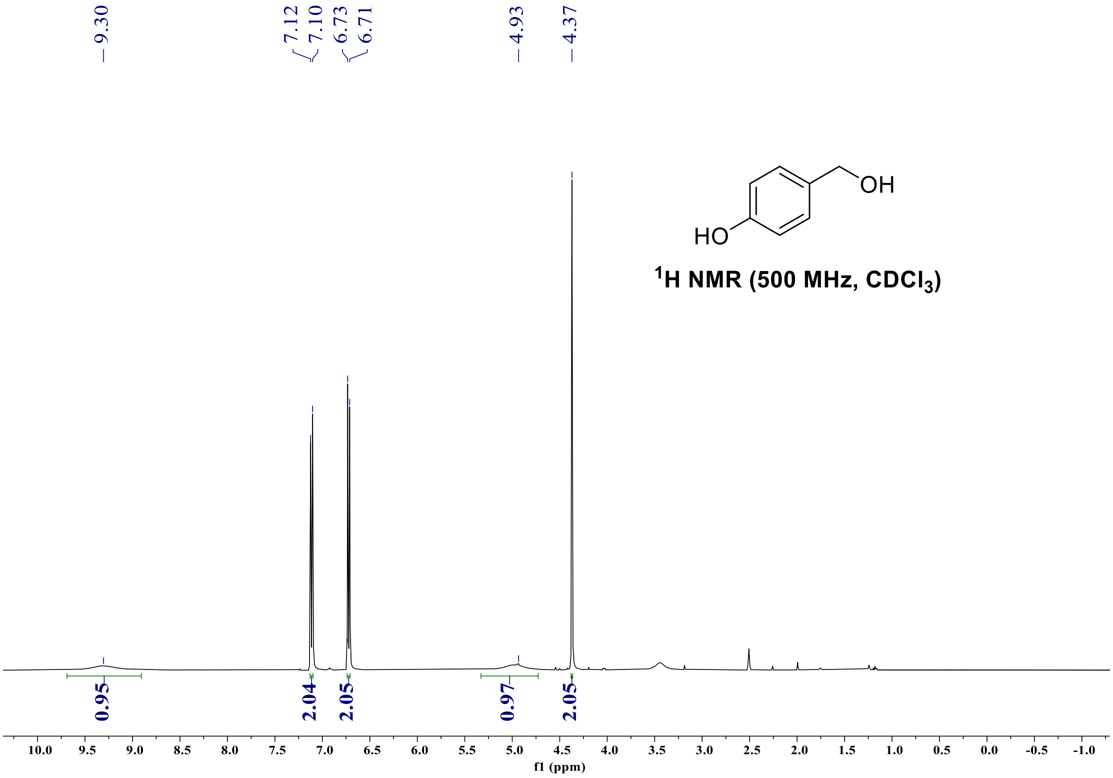

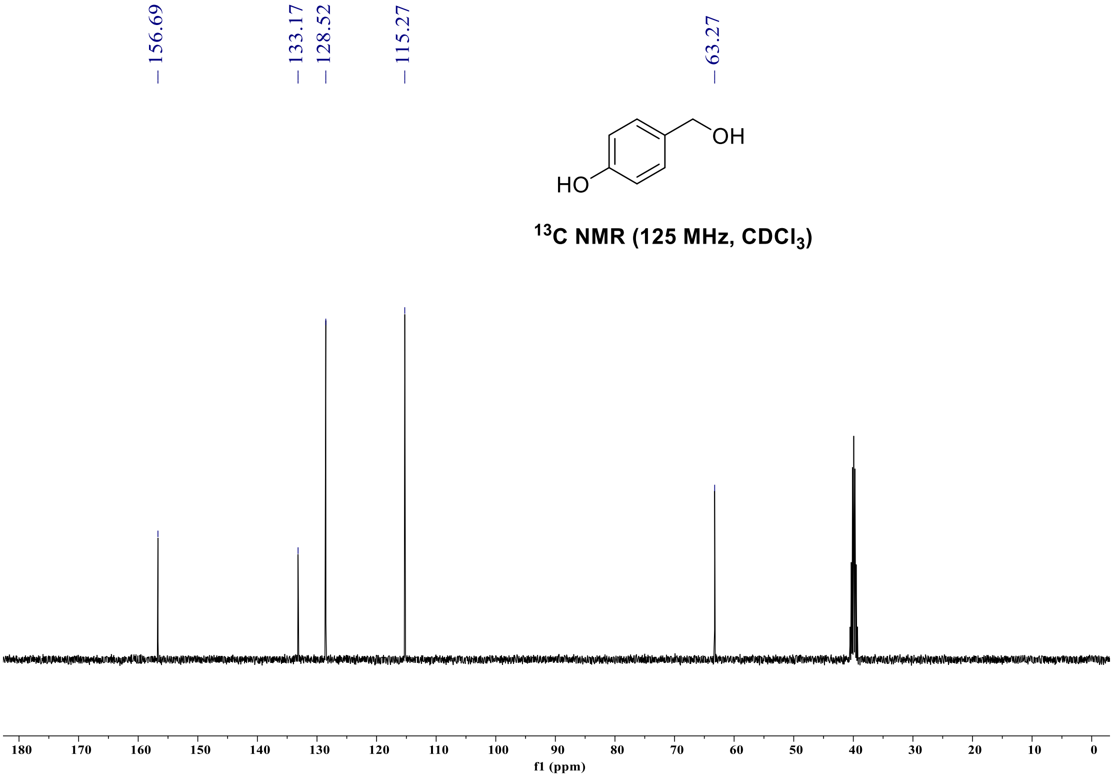

(4-Aminophenyl)methanol (23)

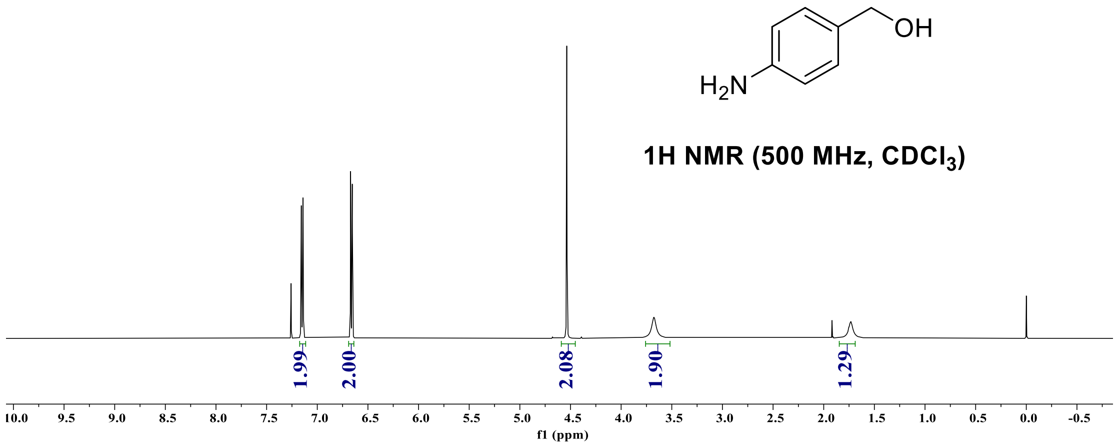

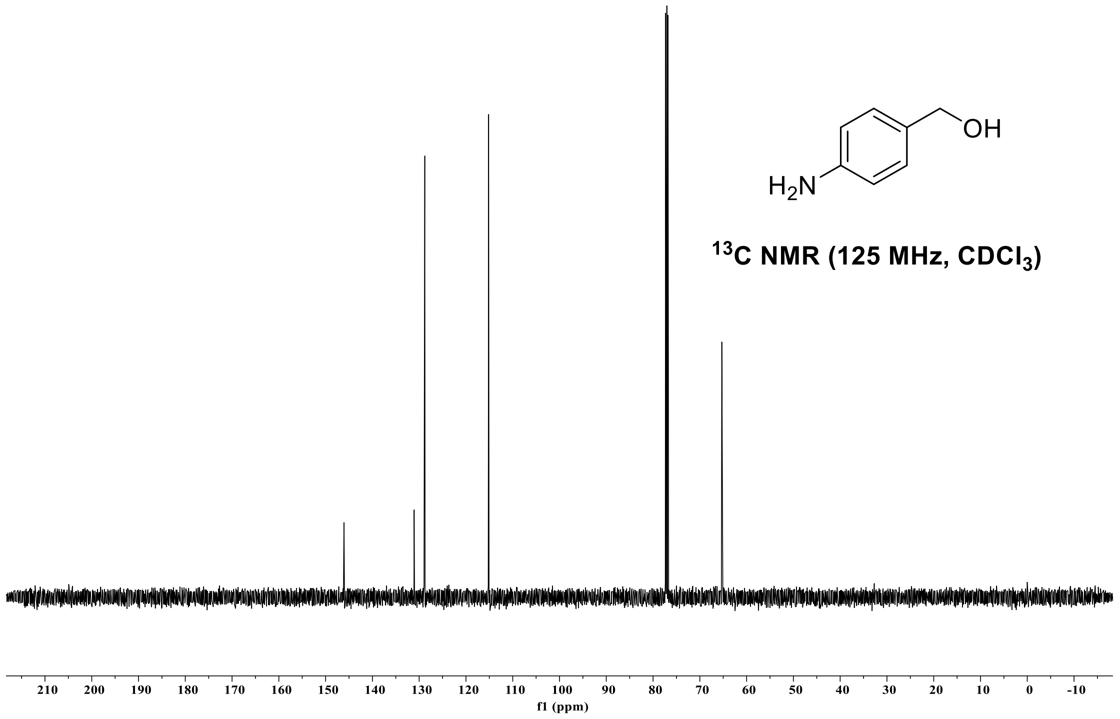

1,4-Phenylenedimethanol (24)

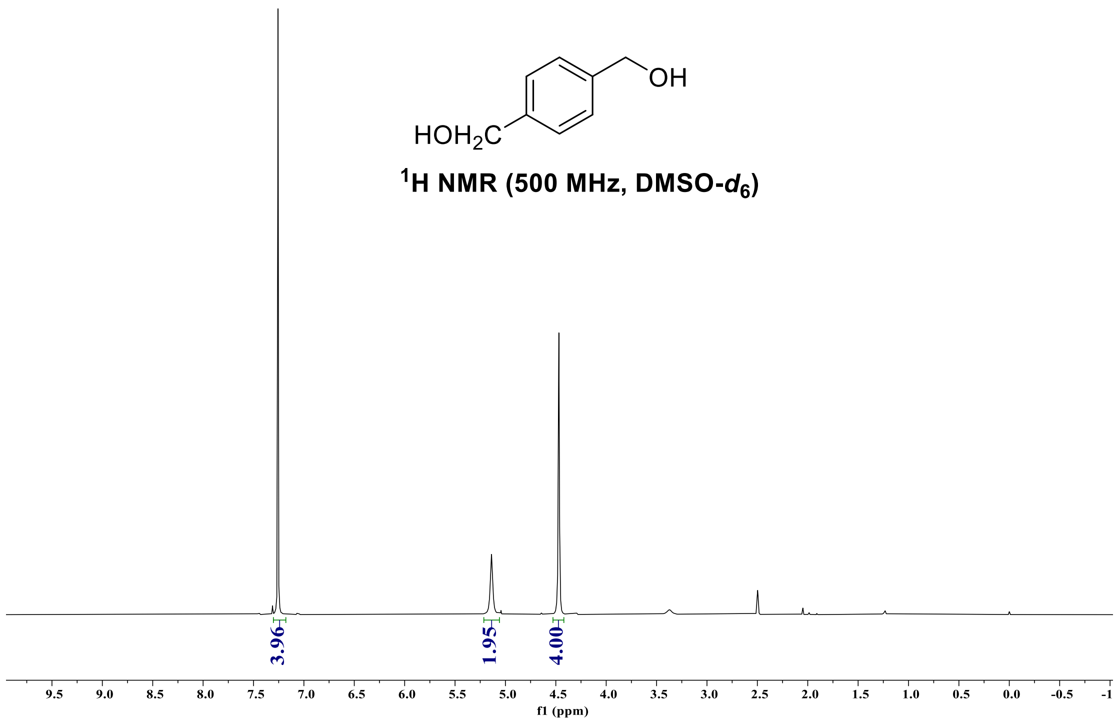

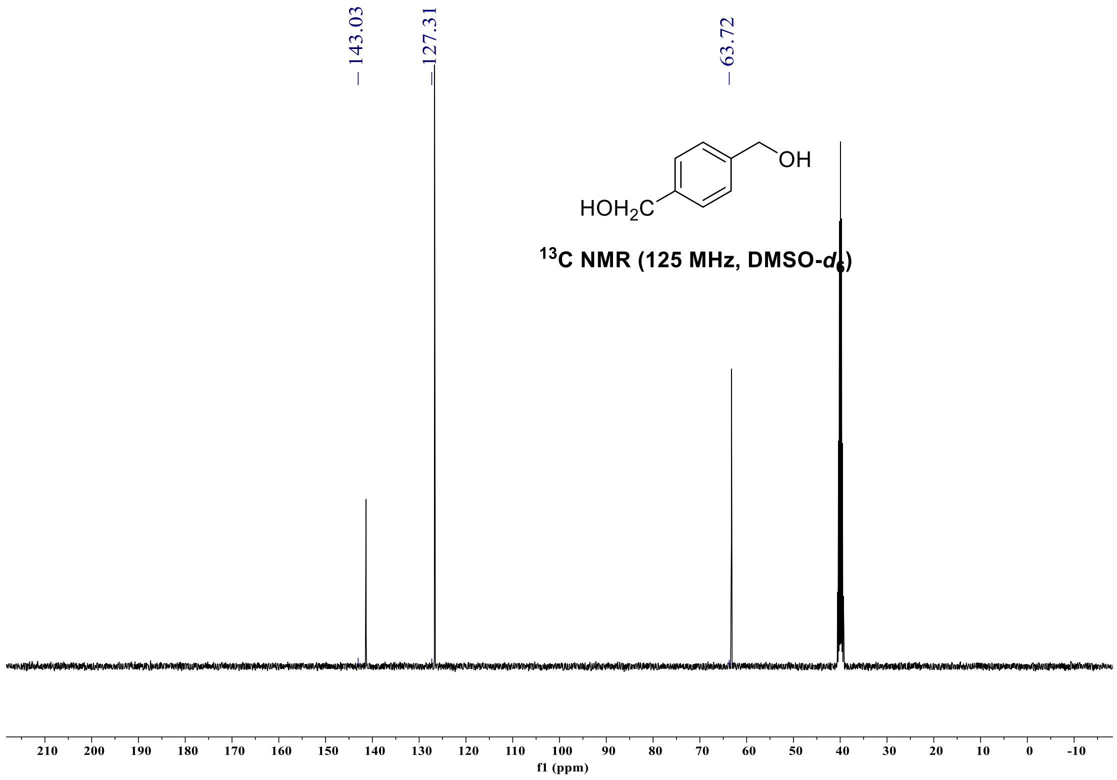

(2-Aminophenyl)methanol (25)

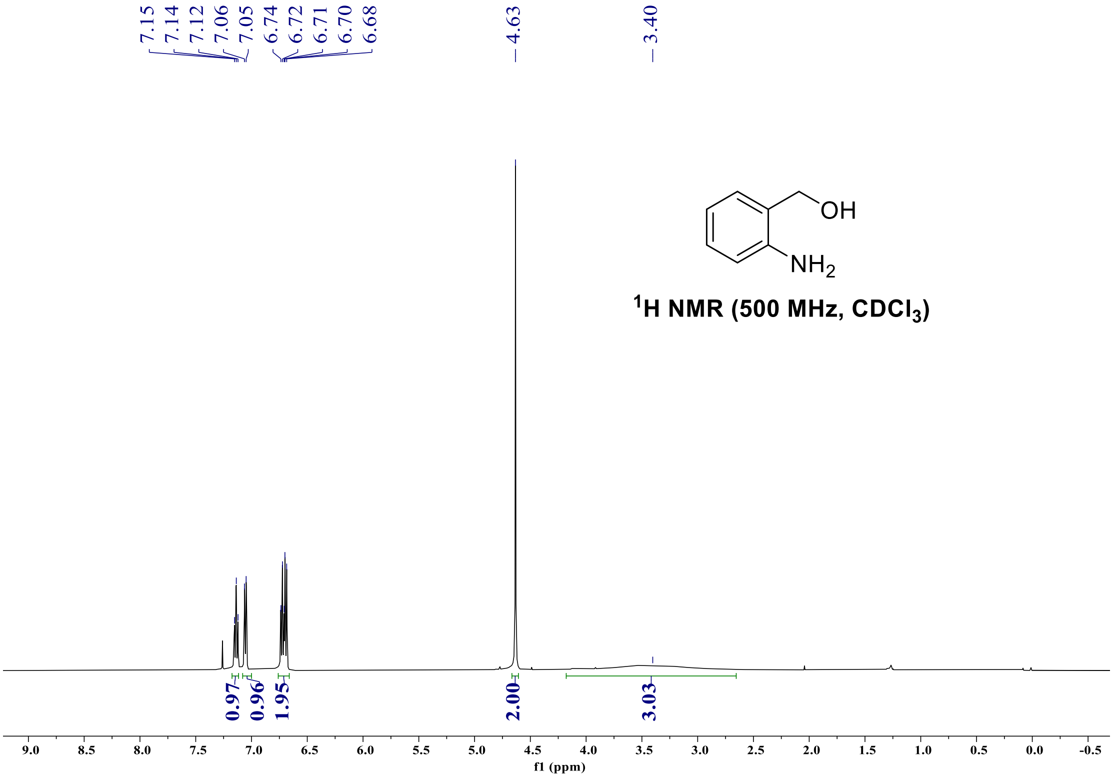

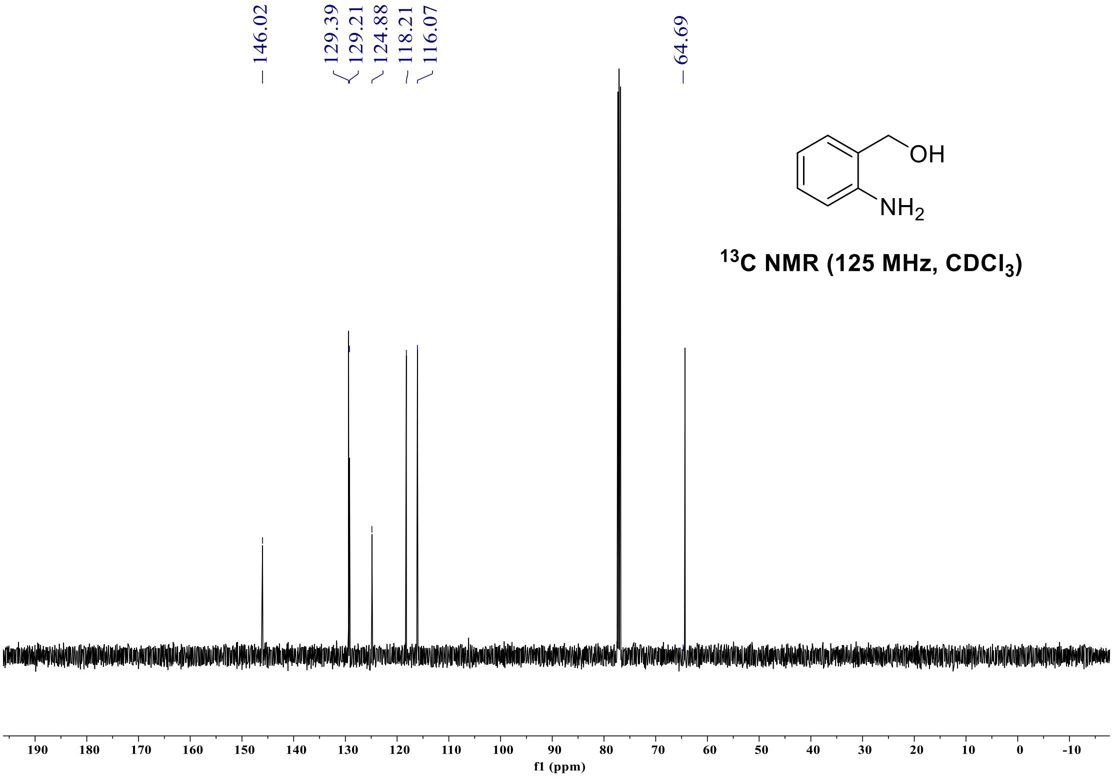

o-tolylmethanol (26)

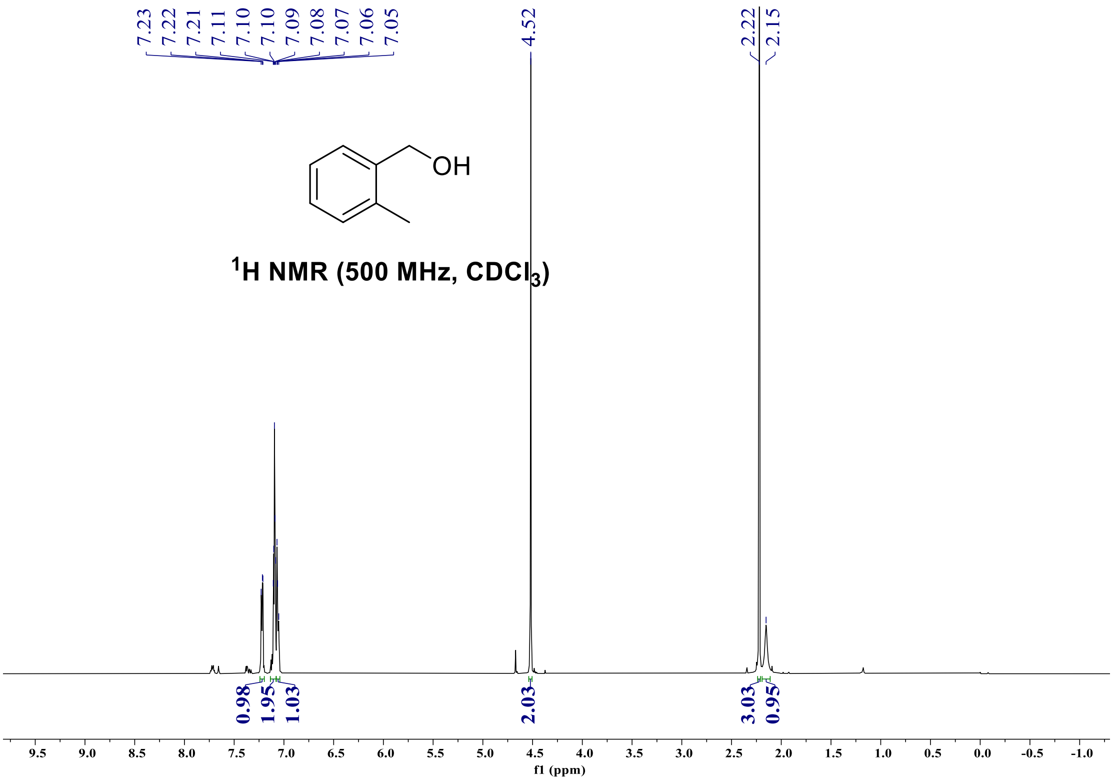

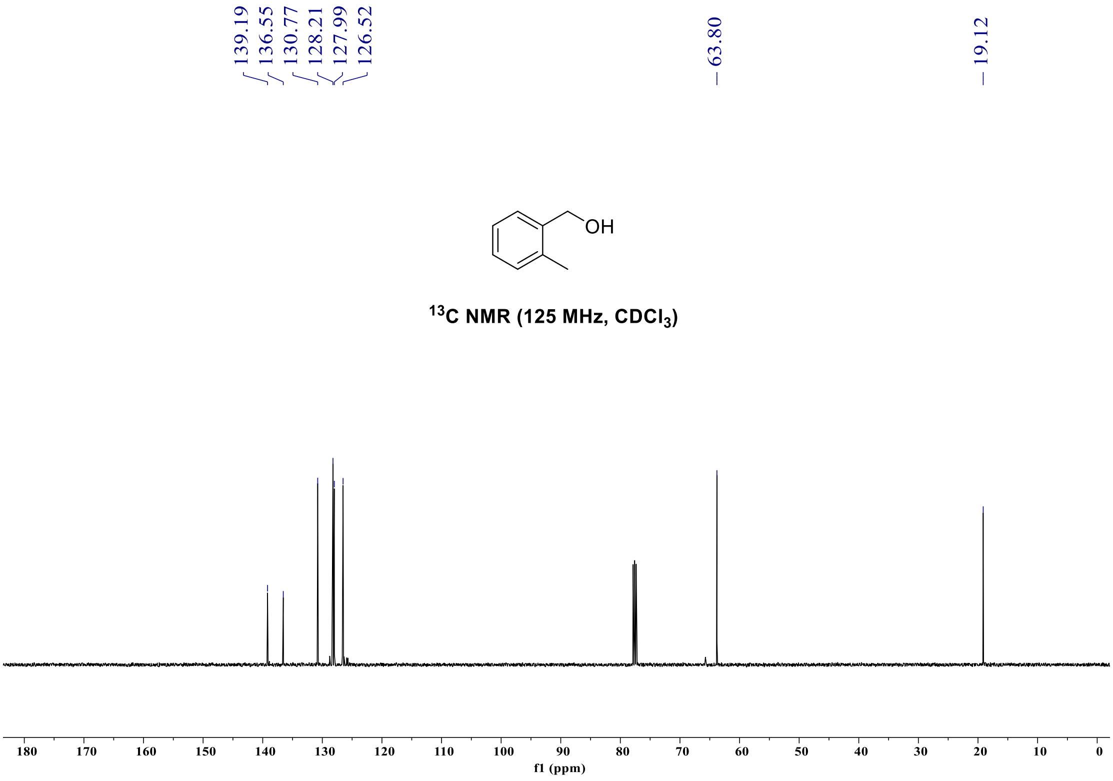

(2-Methoxyphenyl)methanol (27)

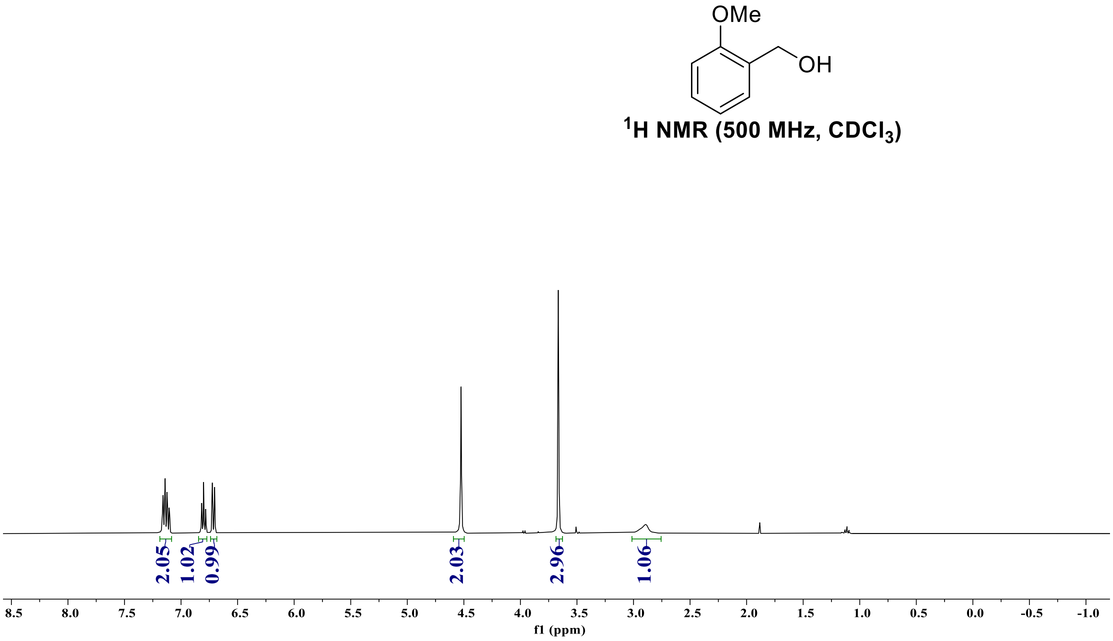

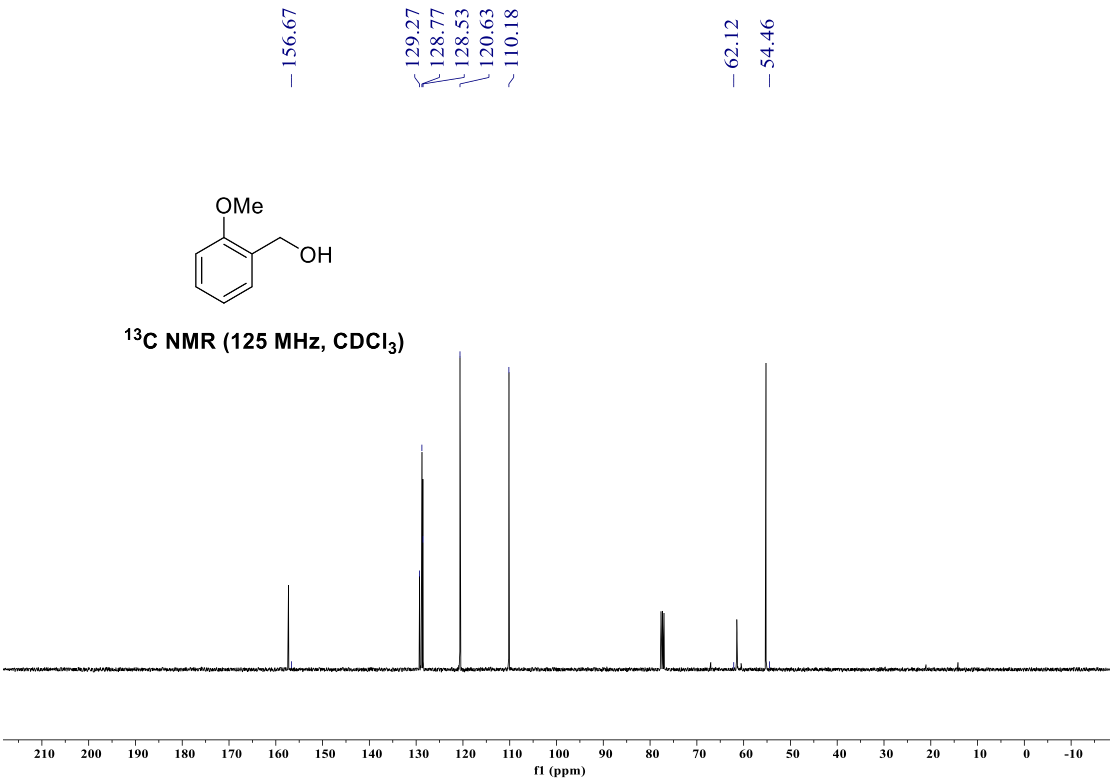

2-(hydroxymethyl)benzonitrile (28)

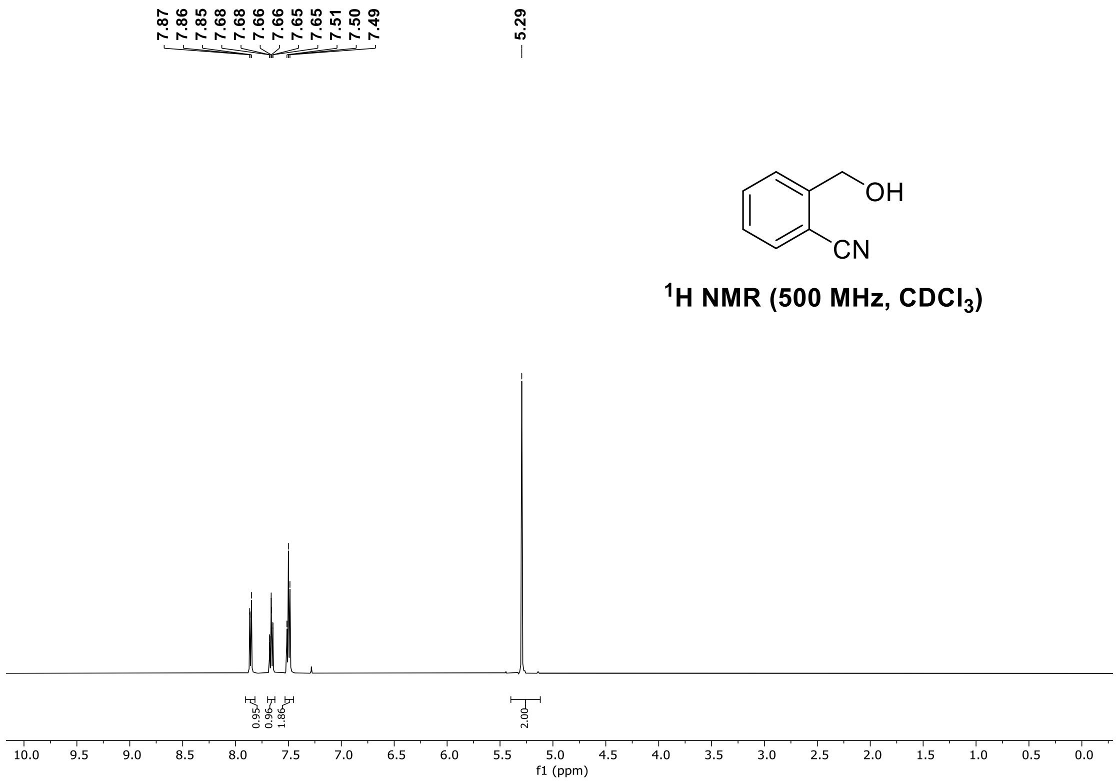

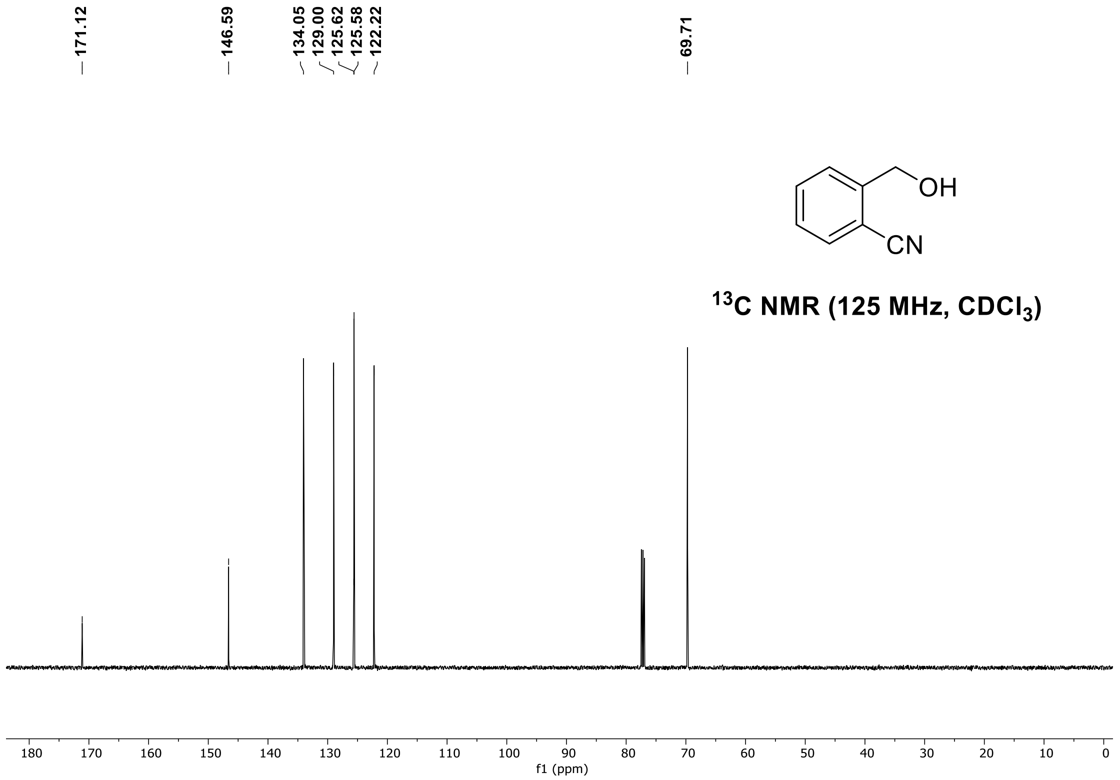

(2-bromophenyl)methanol (29)

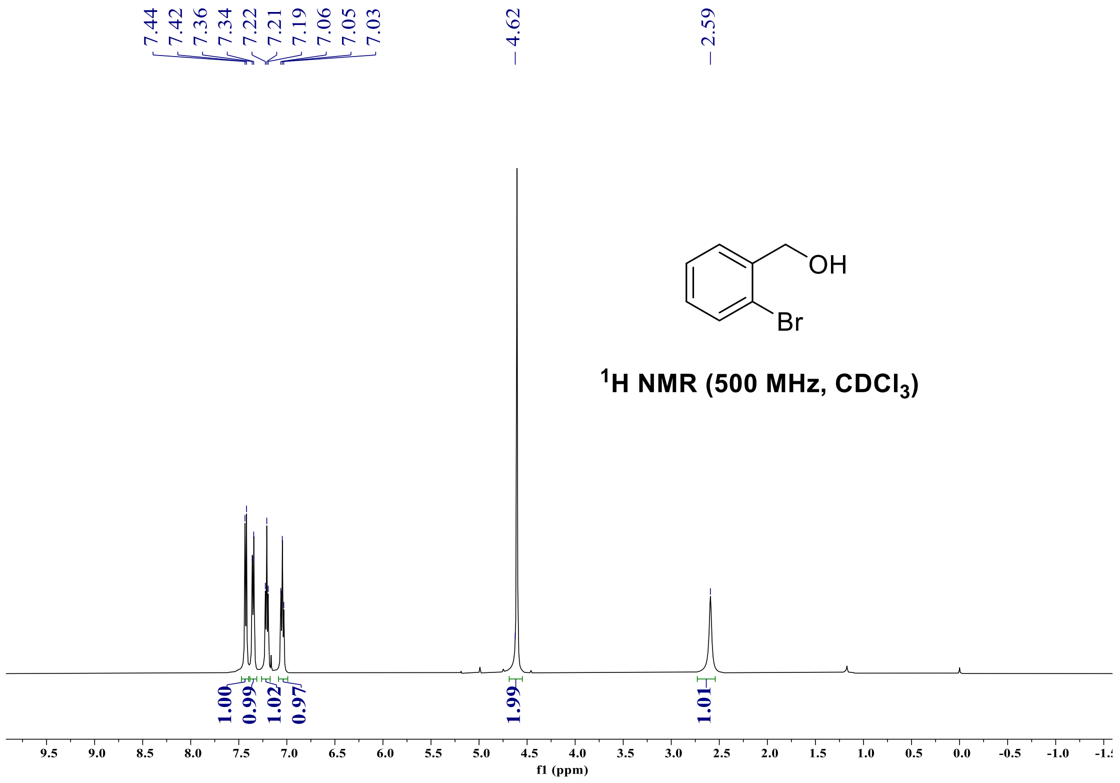

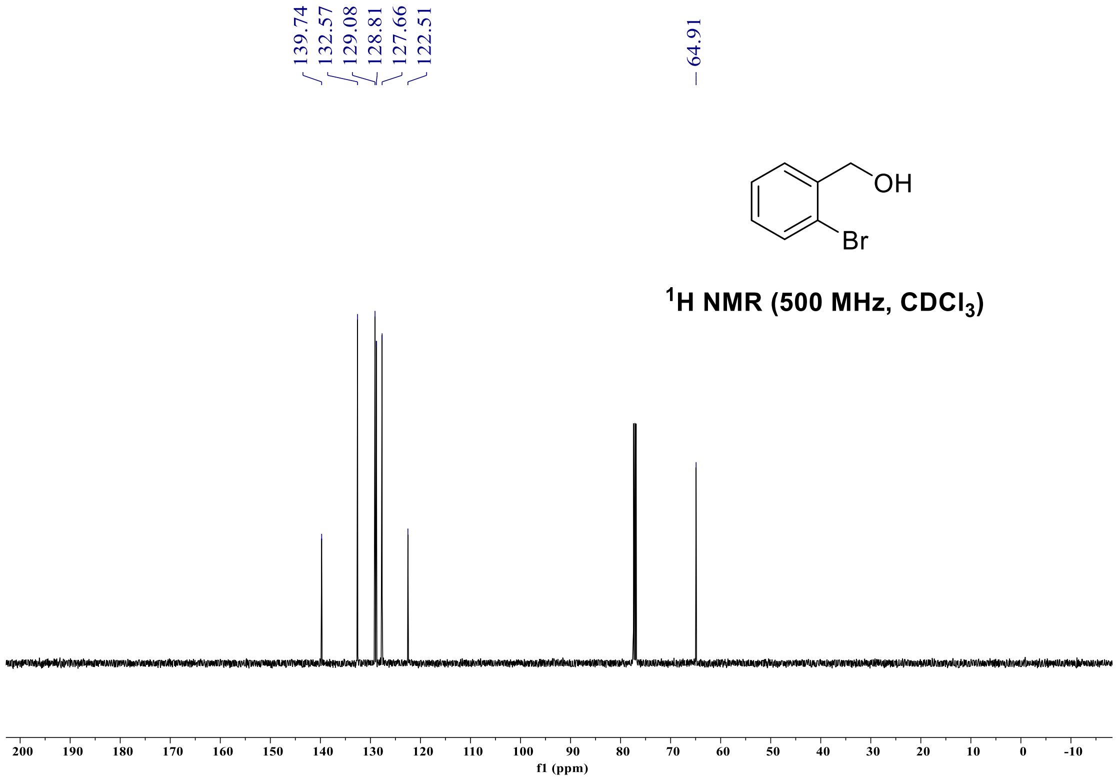

(3-Methoxyphenyl)methanol (30)

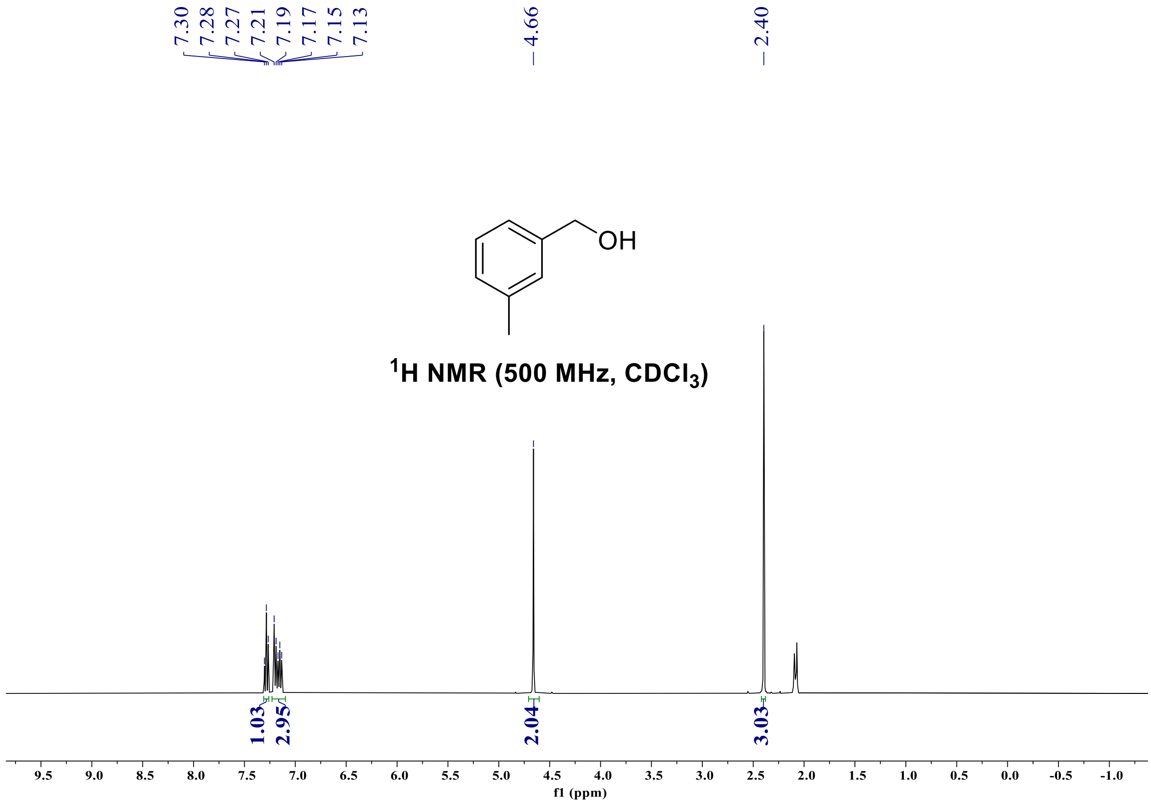

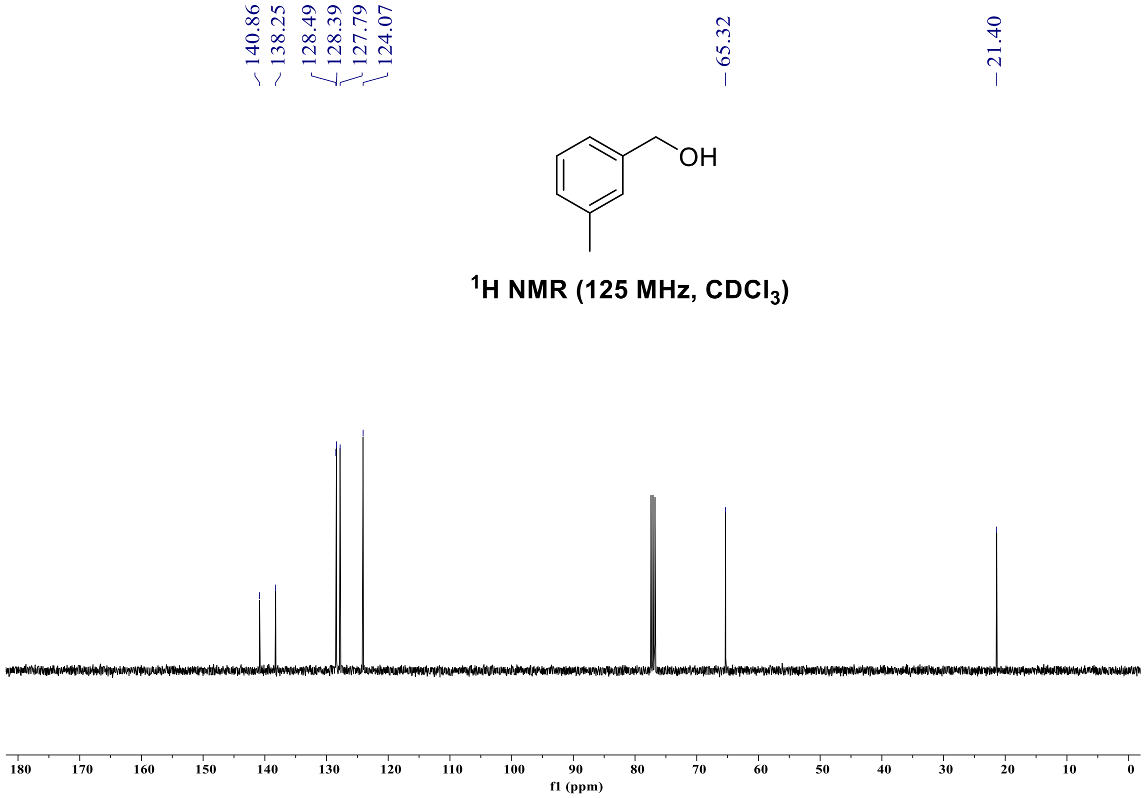

(3-Methoxyphenyl)methanol (31)

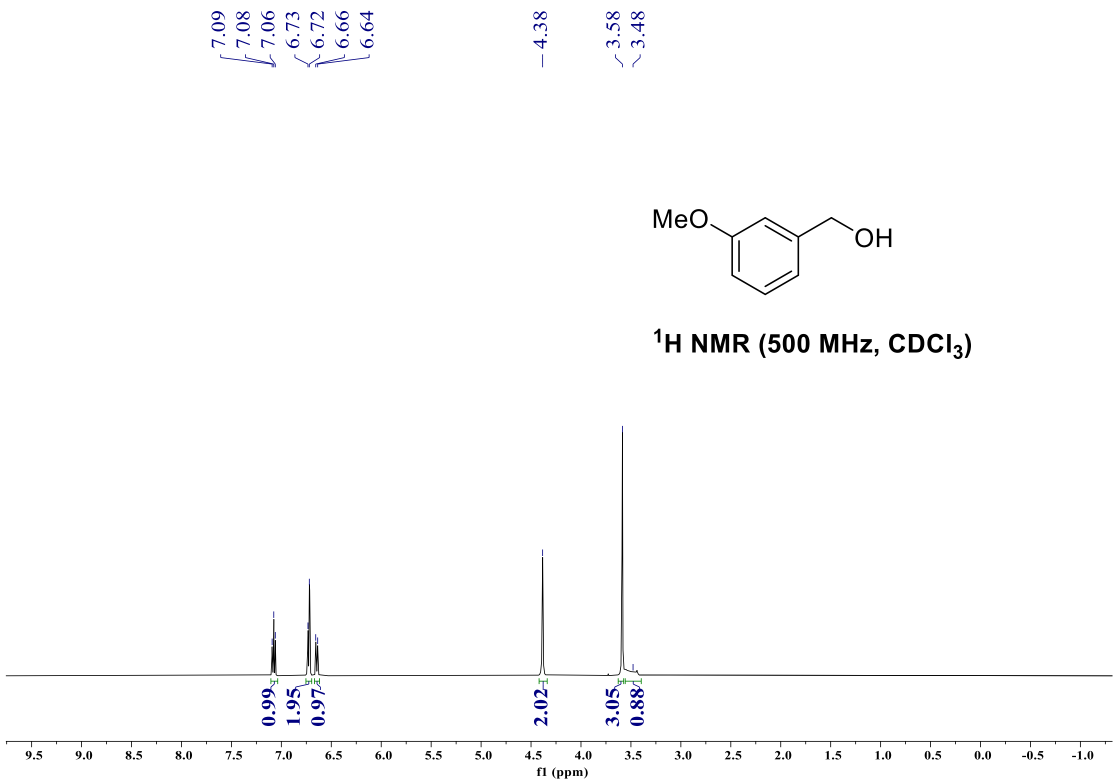

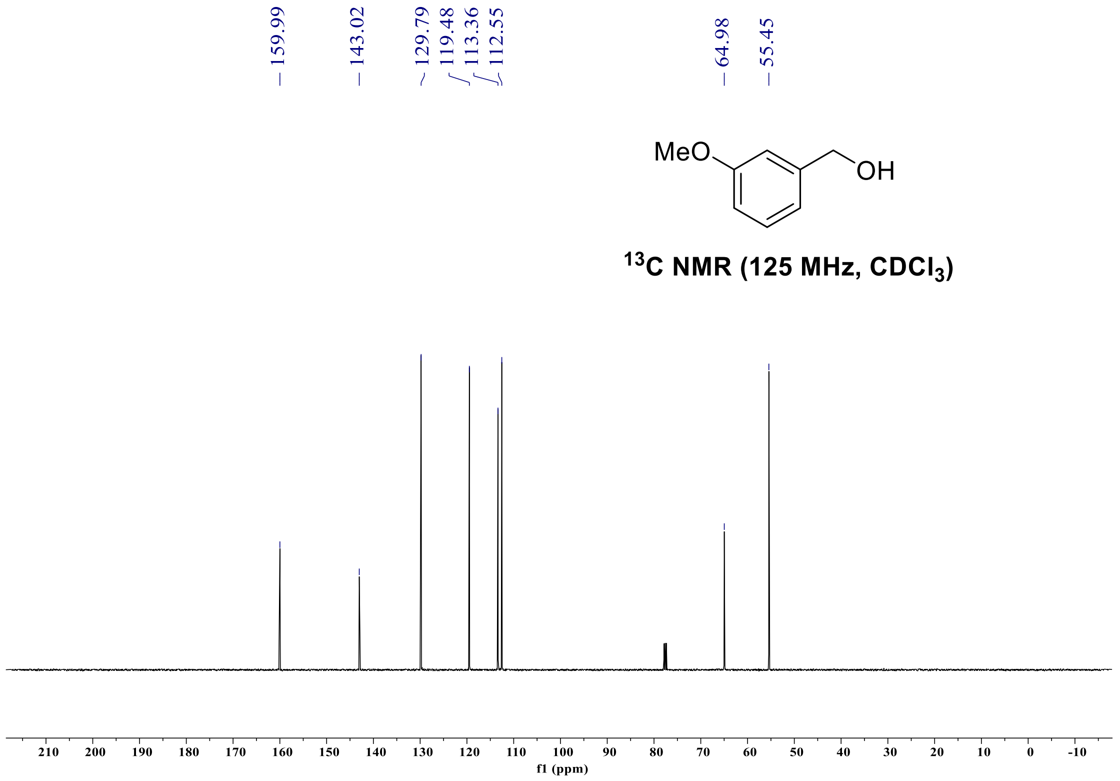

(3-(Trifluoromethyl)phenyl)methanol (32)

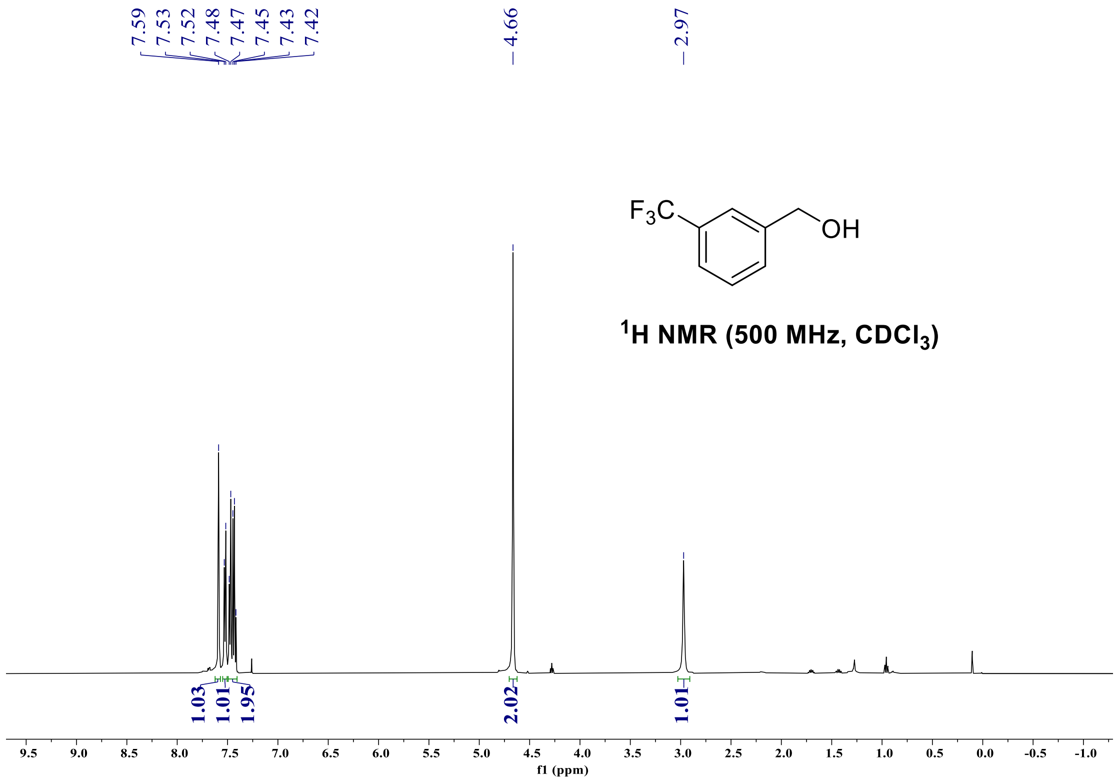

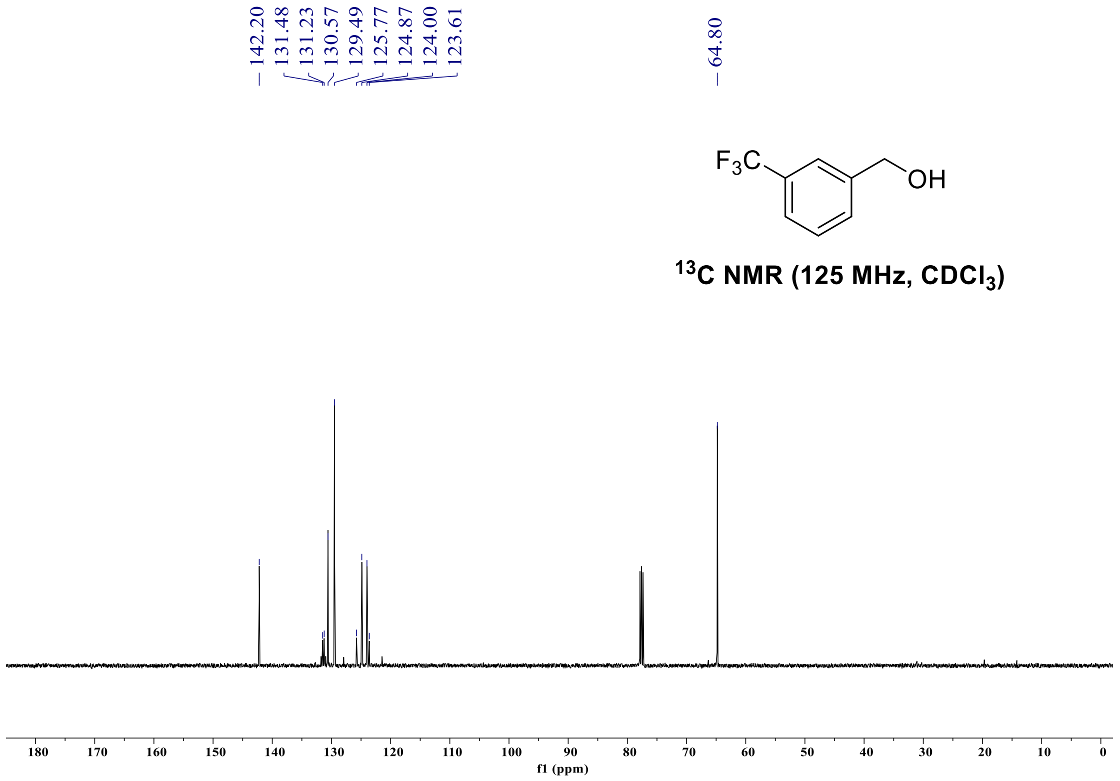

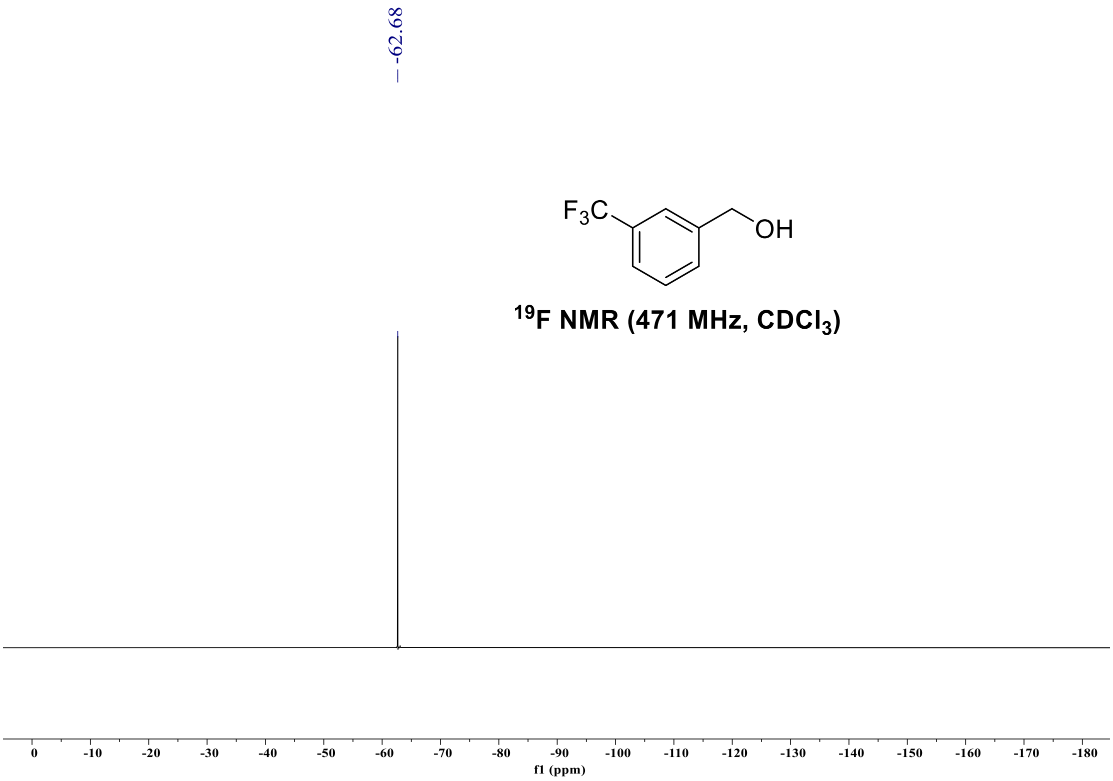

(3-Bromophenyl)methanol (33)

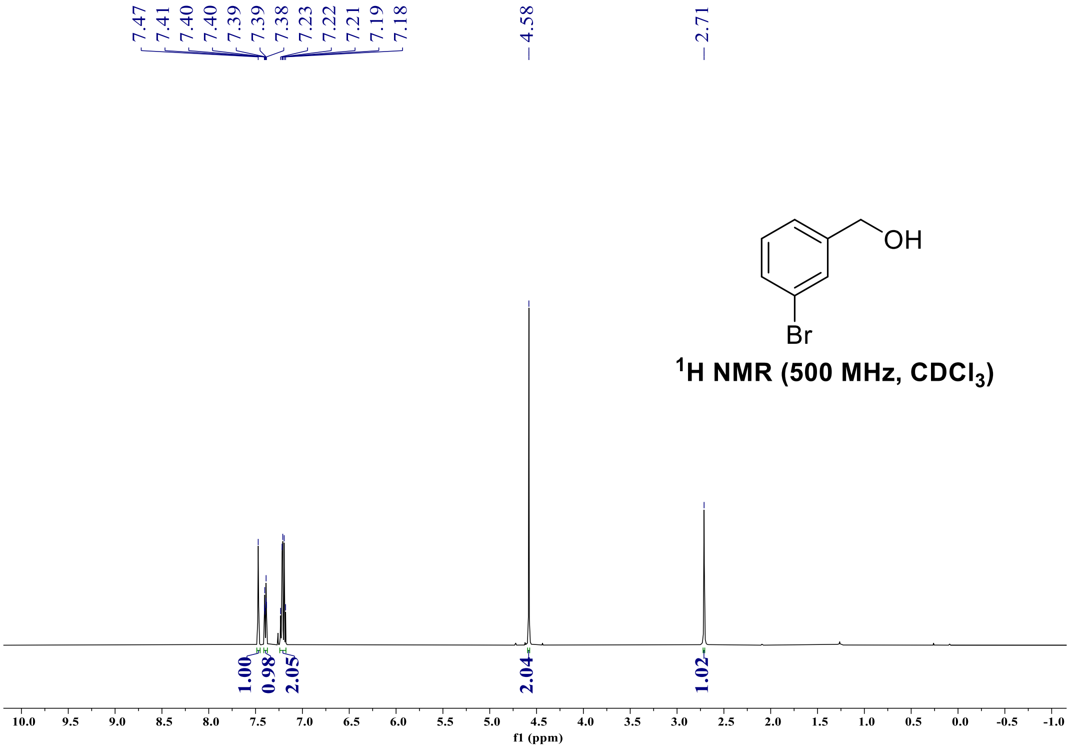

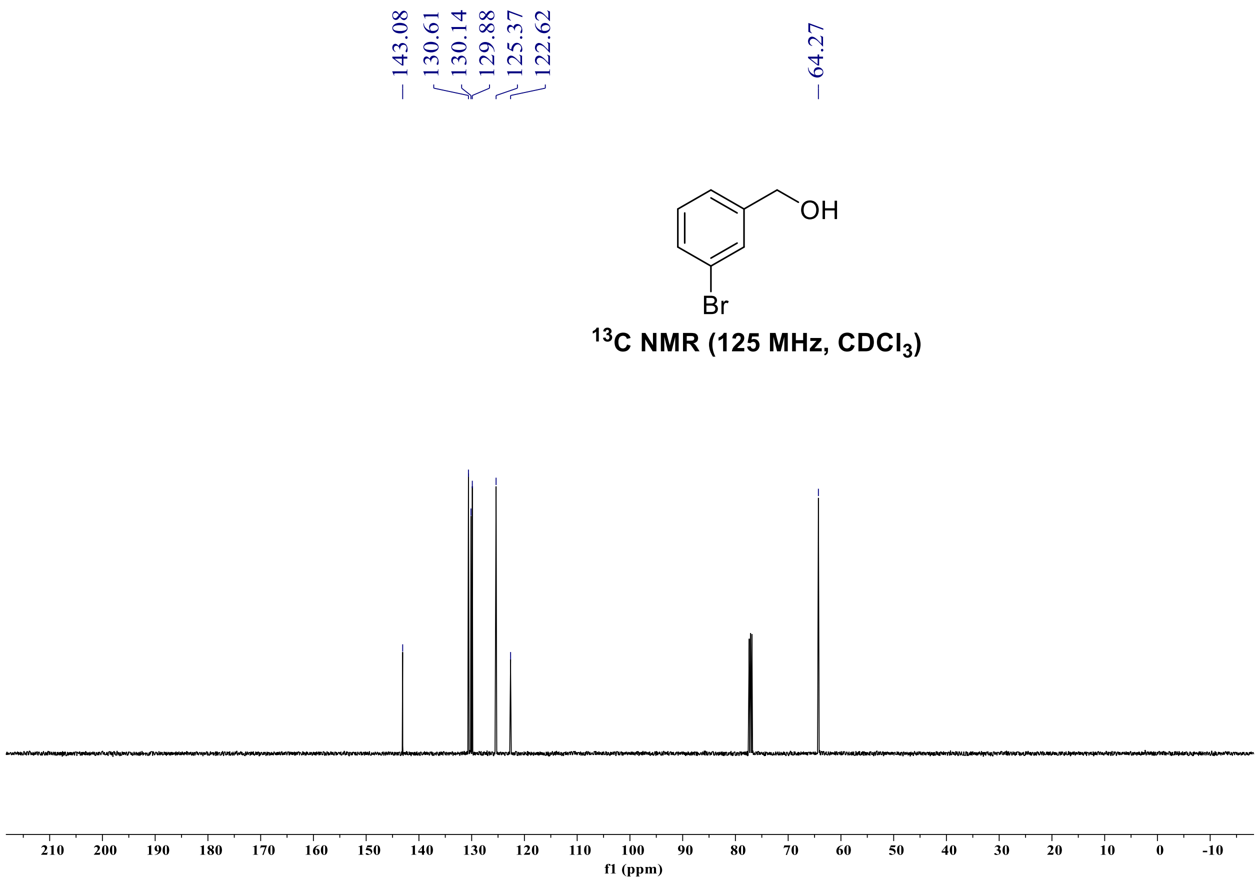

2-(Hydroxymethyl)-6-methylphenol (34)

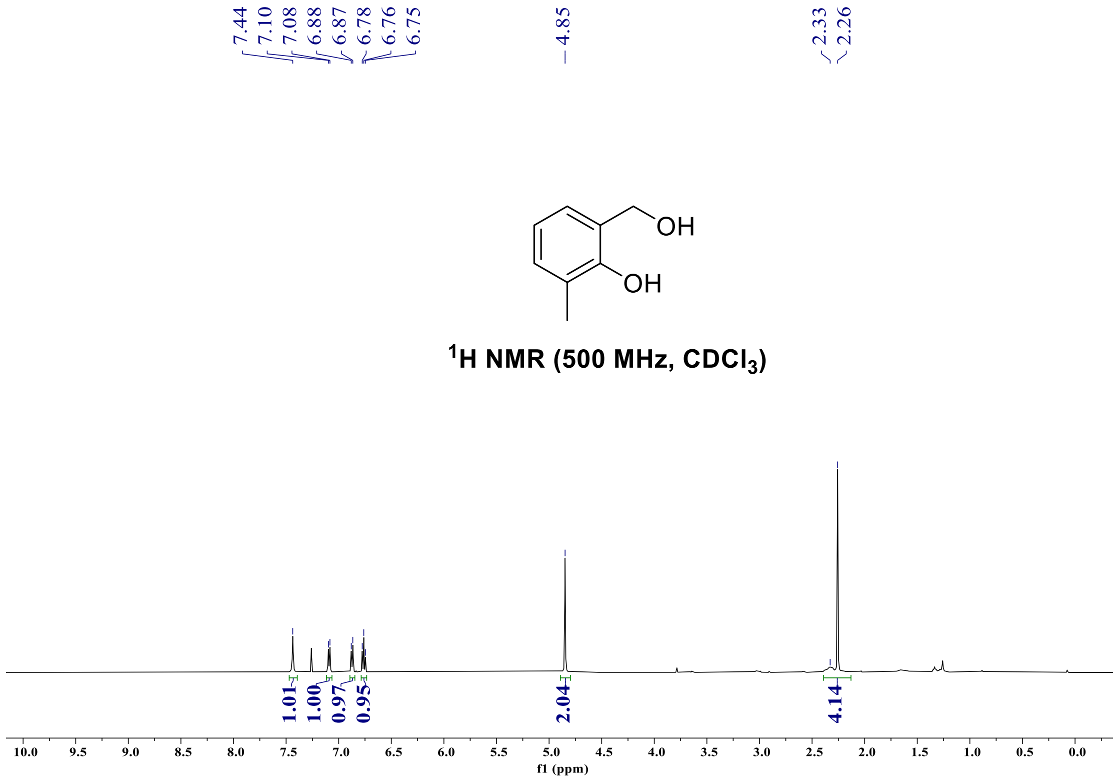

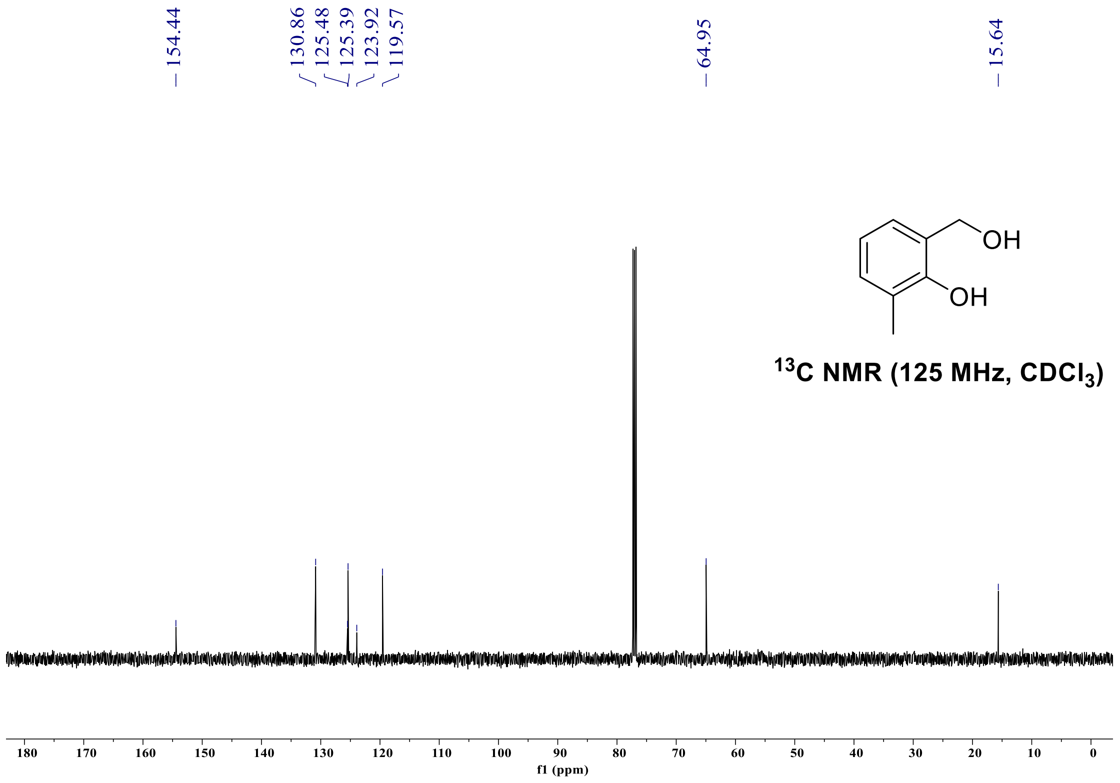

Benzofuran-2-ylmethanol (35)

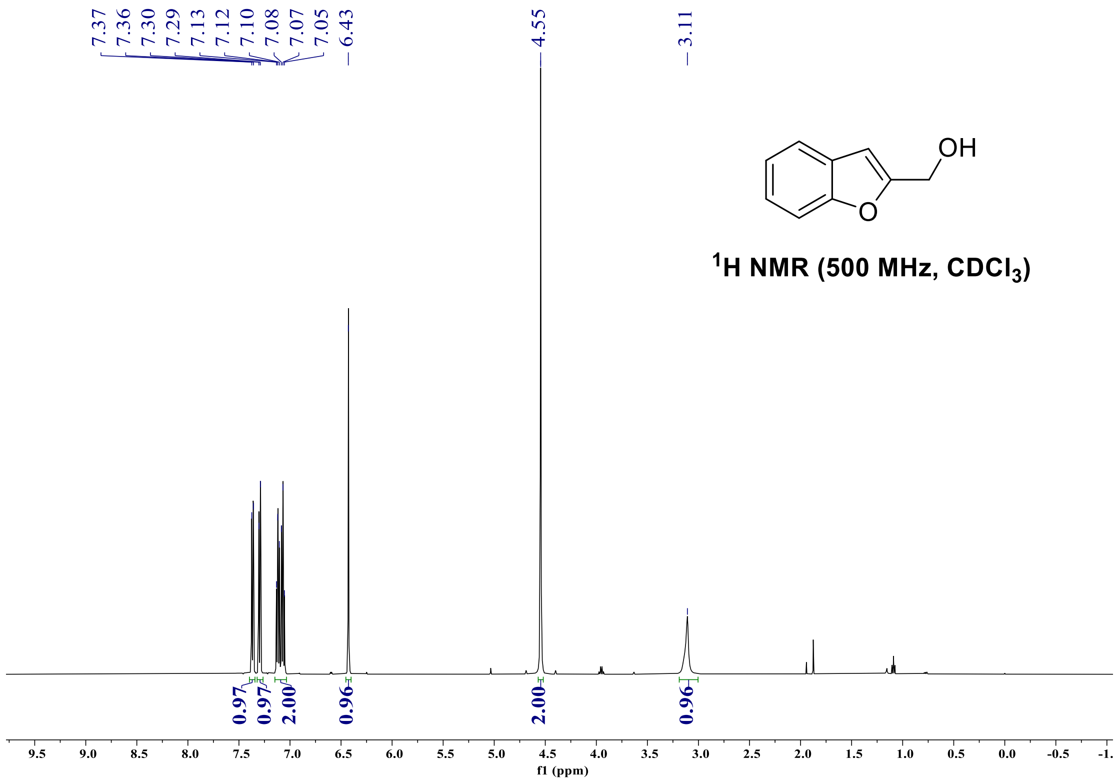

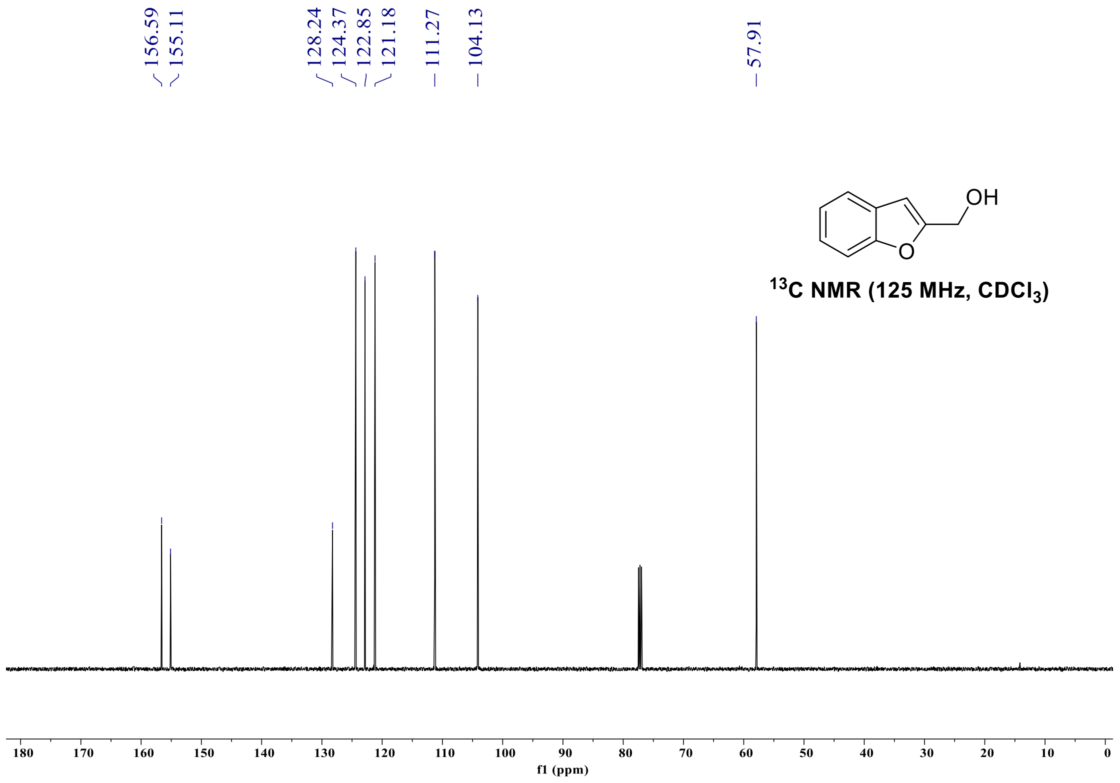

Naphthalen-2-ylmethanol (36)

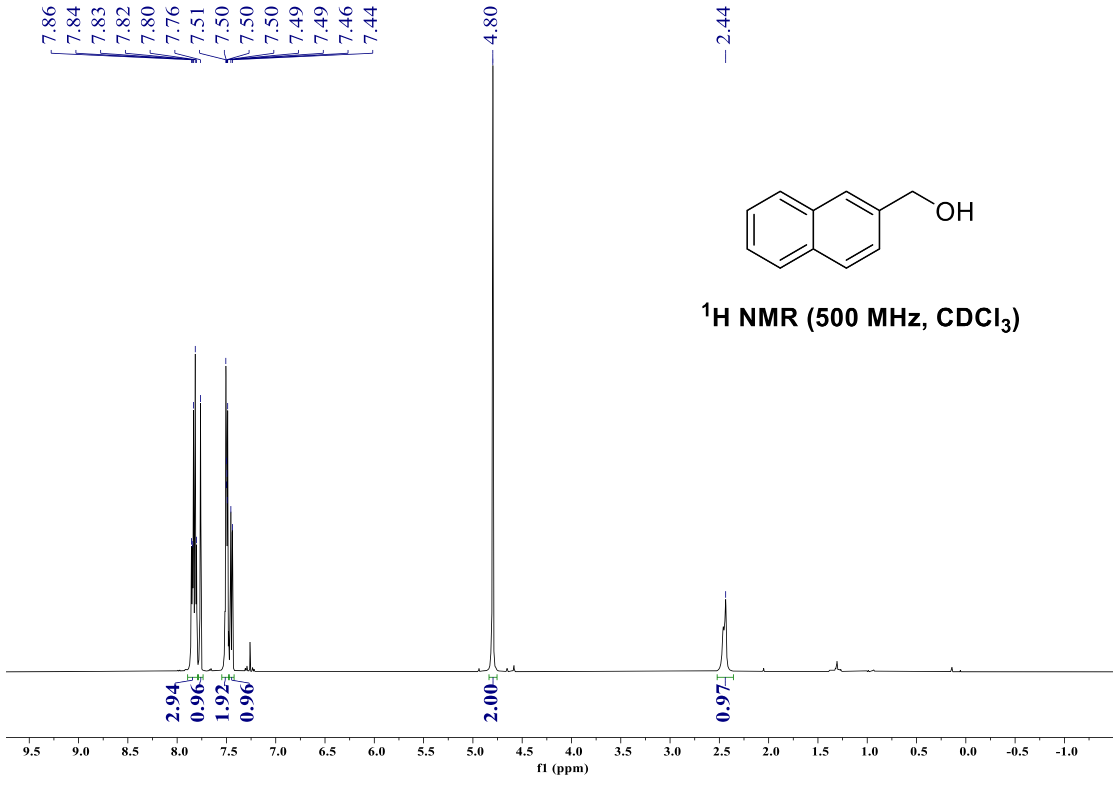

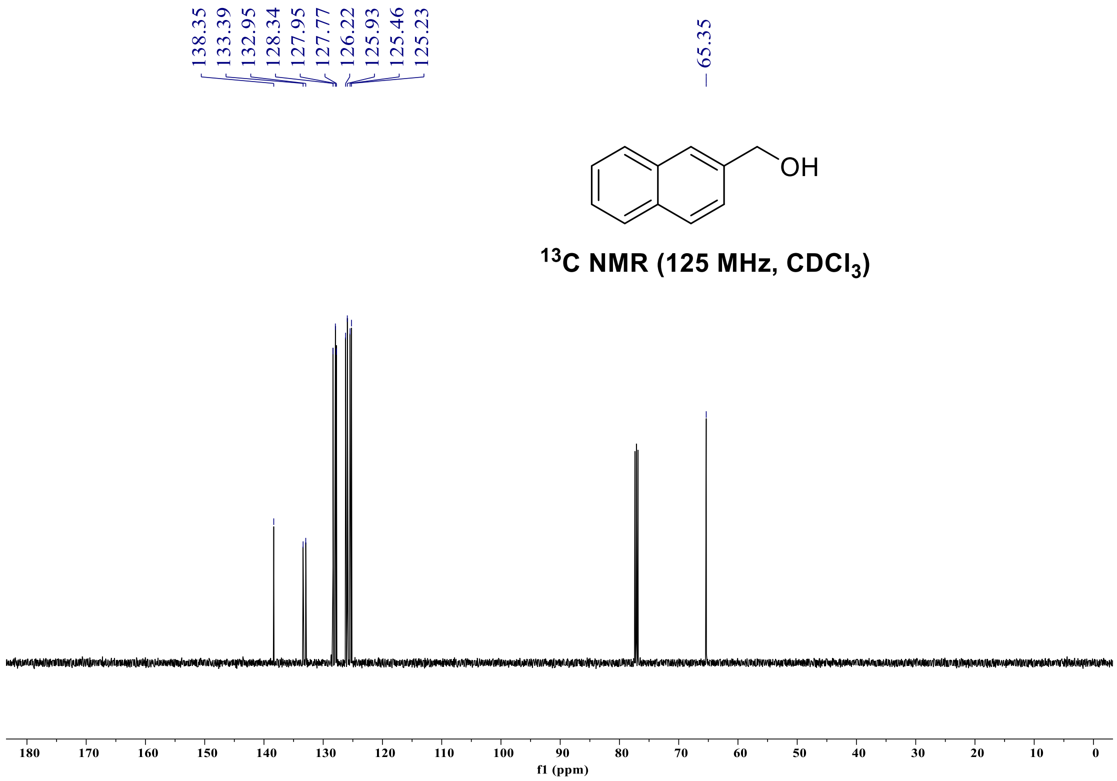

(9-Phenyl-9*H*-carbazol-3-yl)methanol (37)

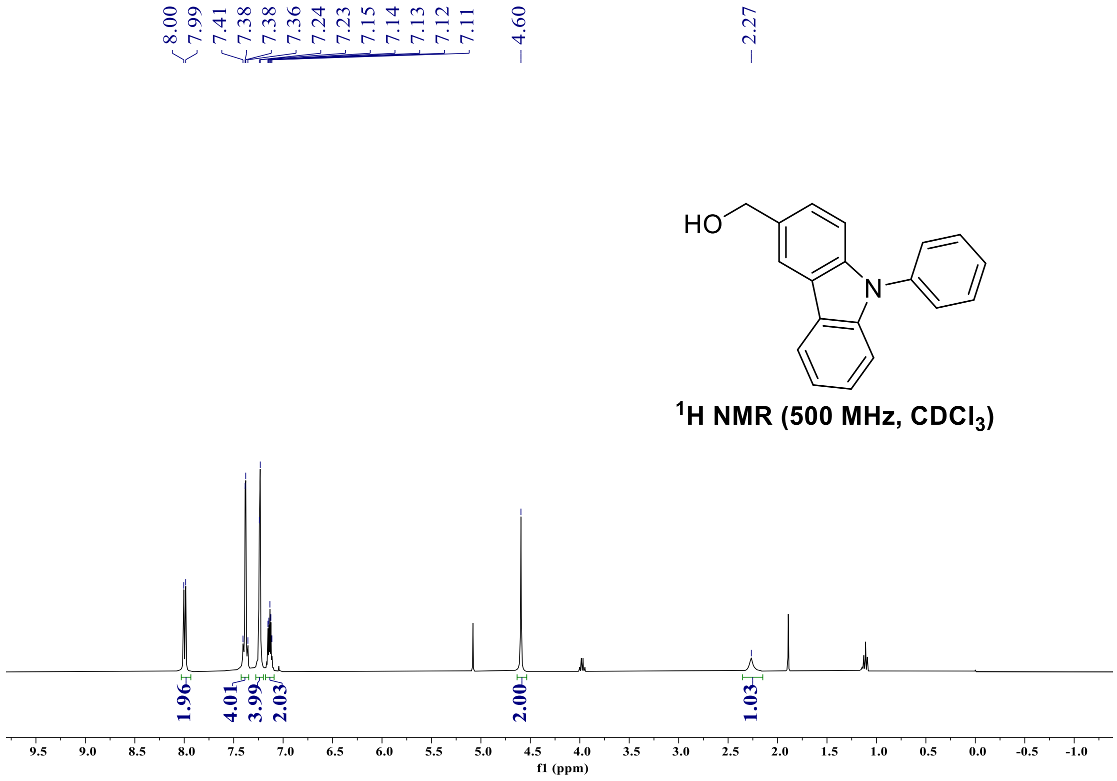

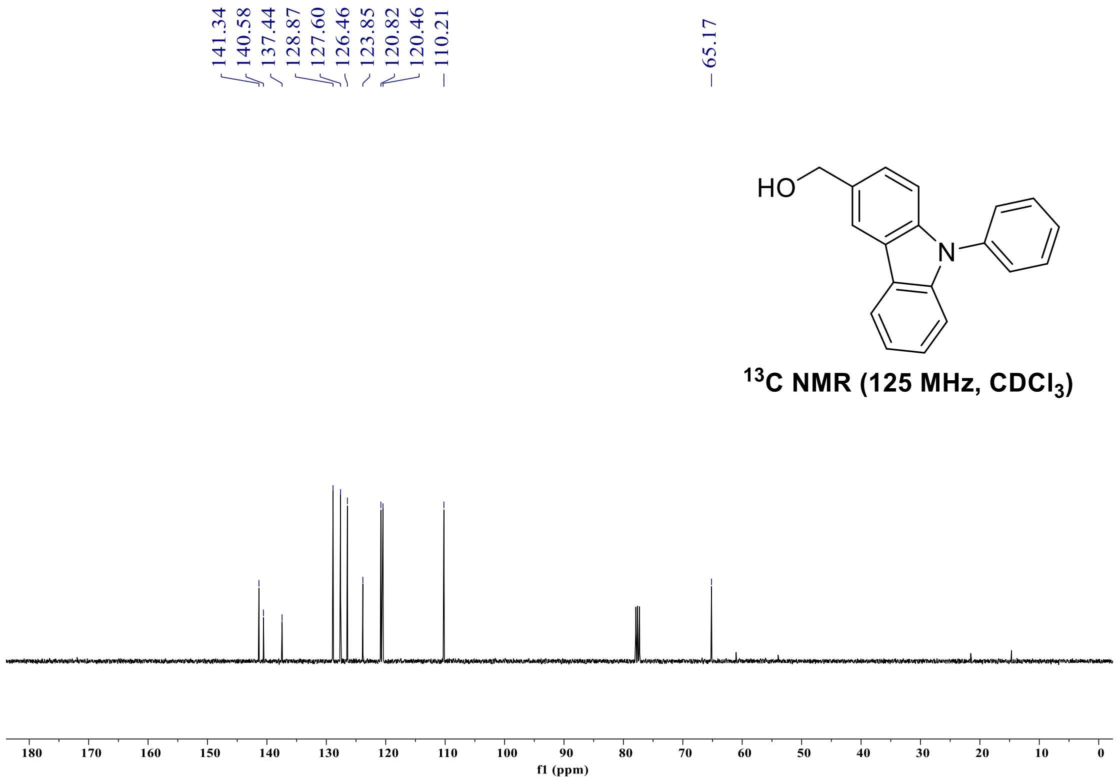

Pyridin-2-ylmethanol (38)

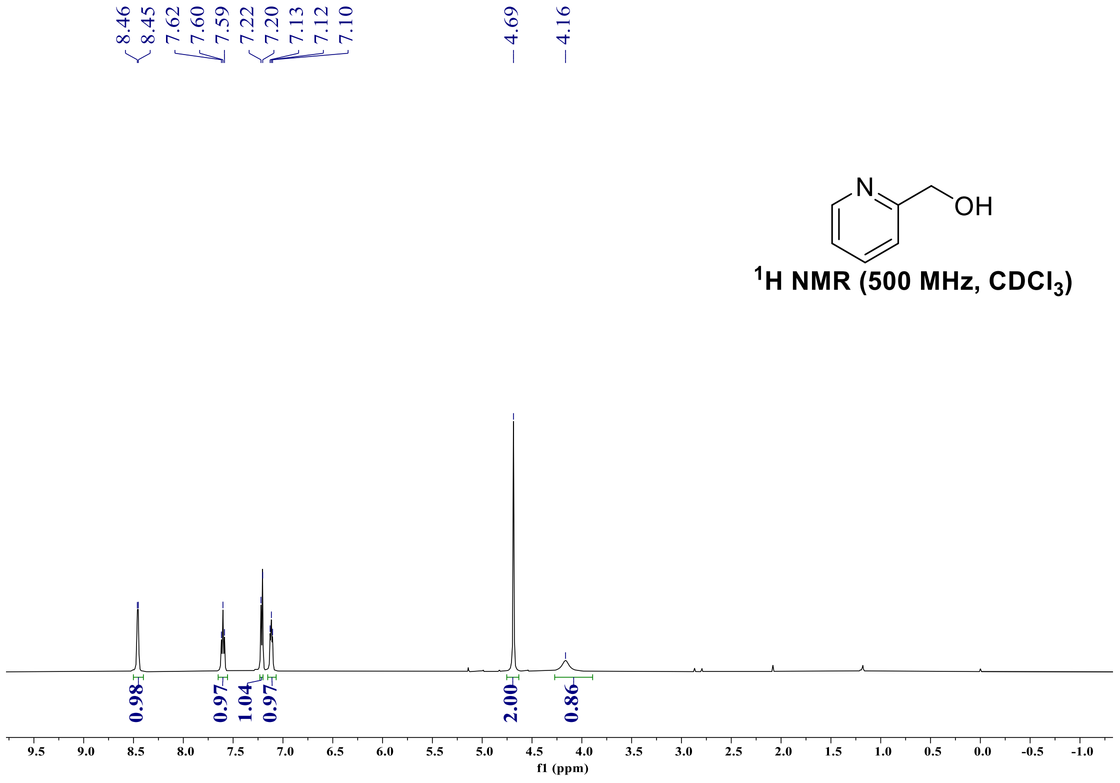

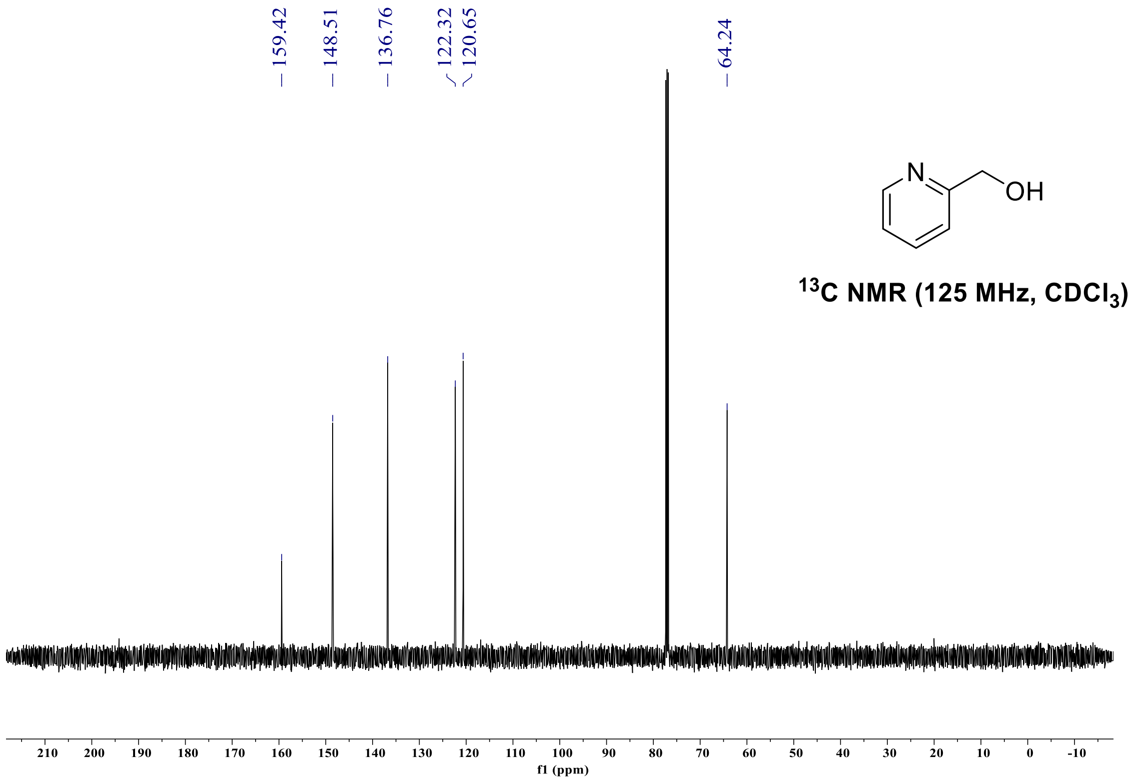

(*E*)-3-phenylprop-2-en-1-ol (39)

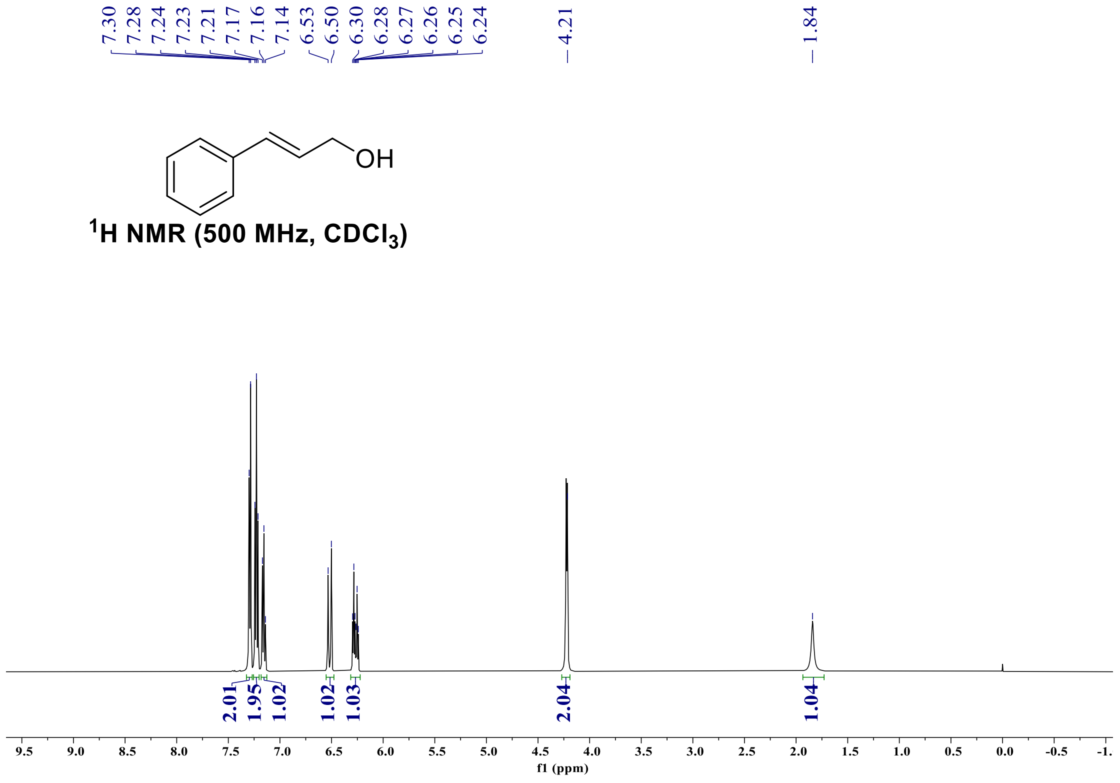

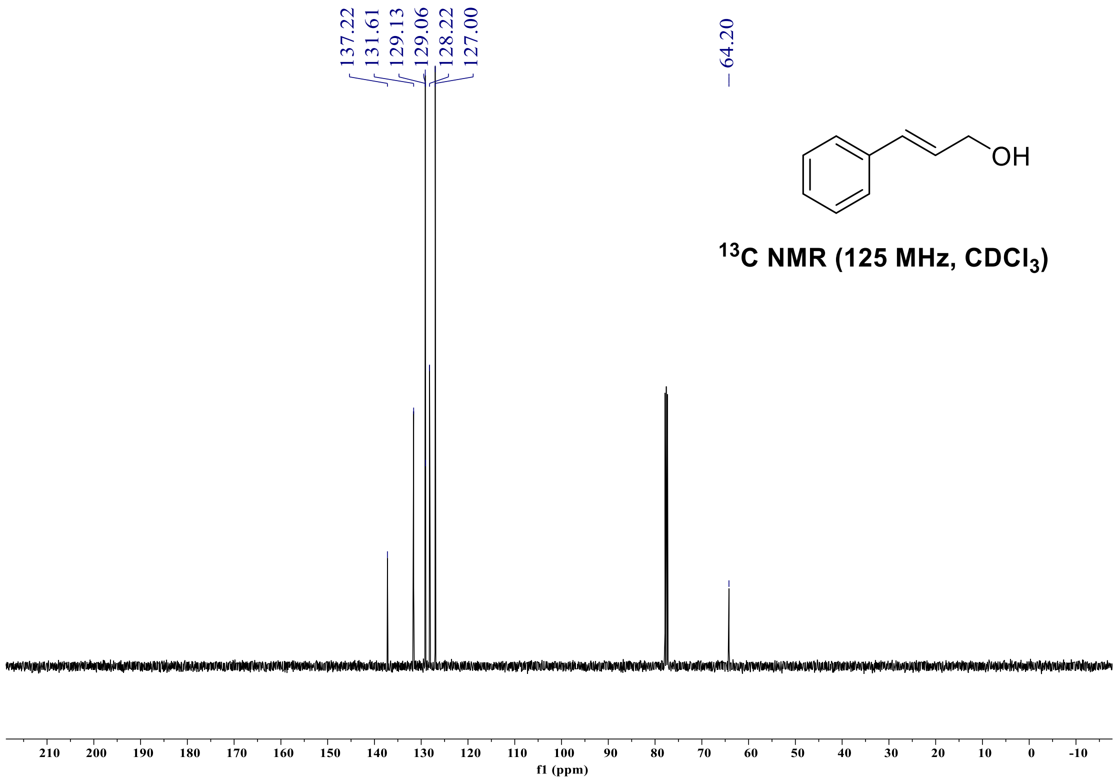

(4-((((3a*S*,5a*R*,8a*R*,8b*S*)-2,2,7,7-tetramethyltetrahydro-3a*H*-bis([1,3]dioxolo)[4,5-b:4',5'-d]pyran-3a-yl)methoxy)methyl)phenyl)methanol (40)

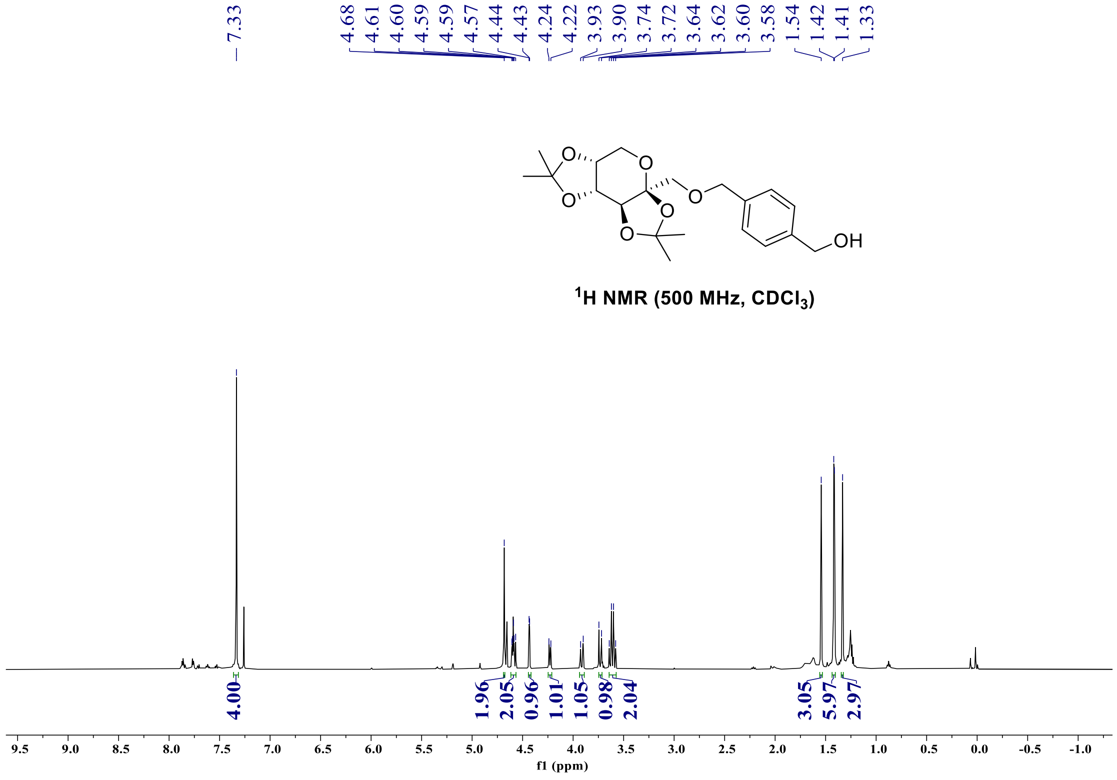

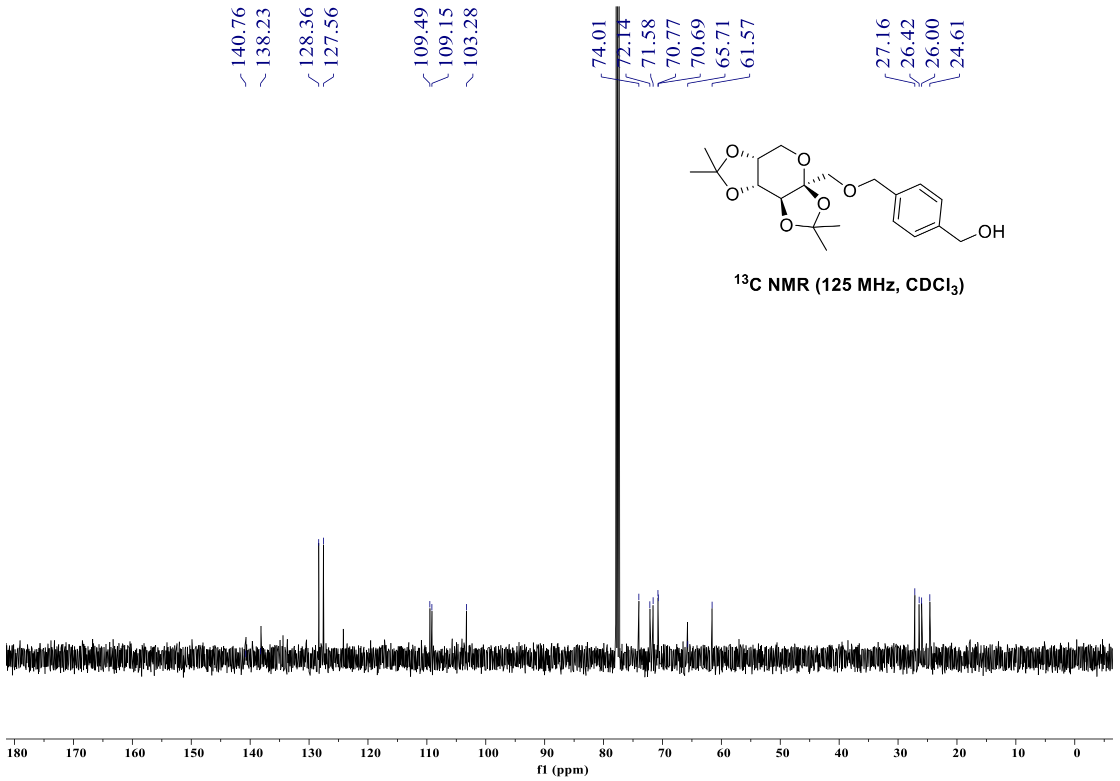

(*S*)-4-(2-((tert-butoxycarbonyl)amino)-3-methoxy-3-oxopropyl)phenyl 4-(((dimethyl(phenyl)silyl)oxy)methyl)benzoate (41)

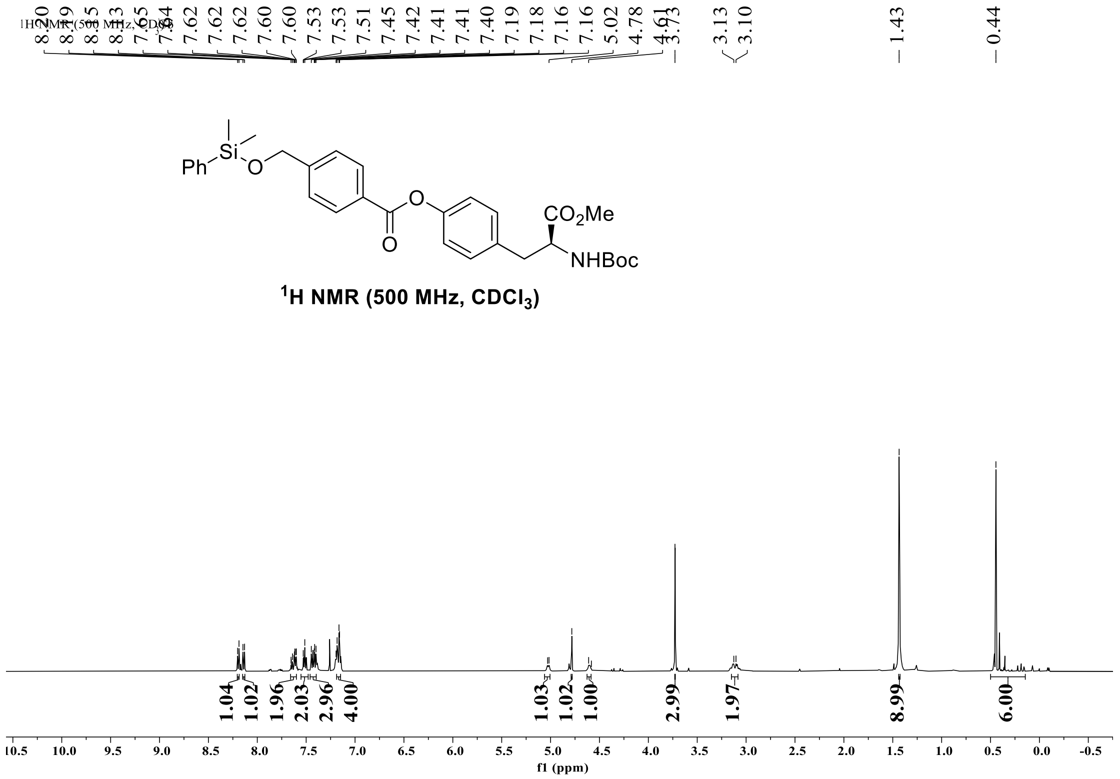

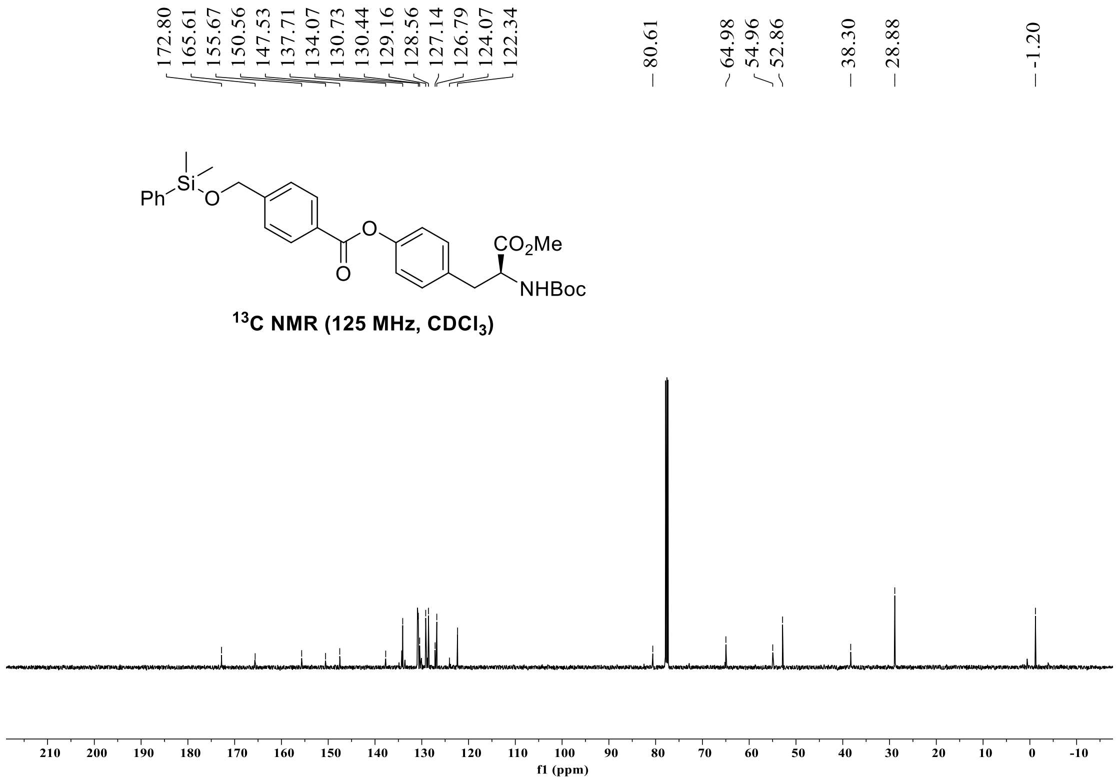

(1*S*,2*S*,5*S*)-2-Isopropyl-5-methylcyclohexyl 4-(hydroxymethyl)benzoate (42)

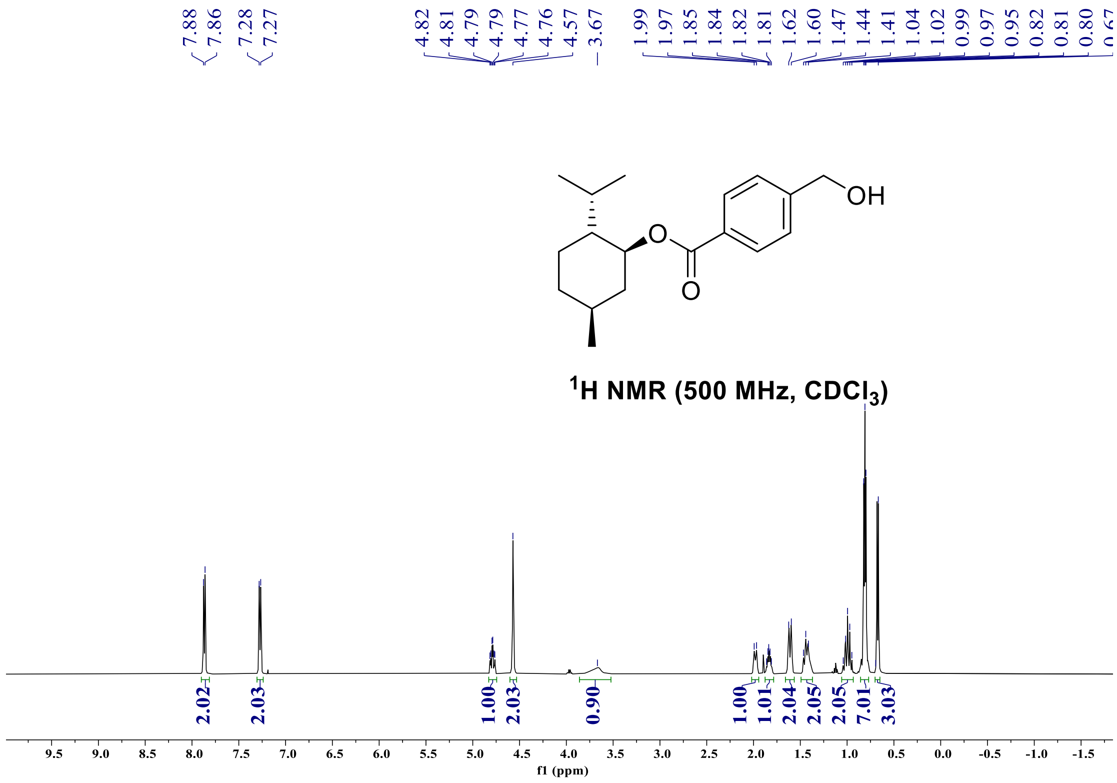

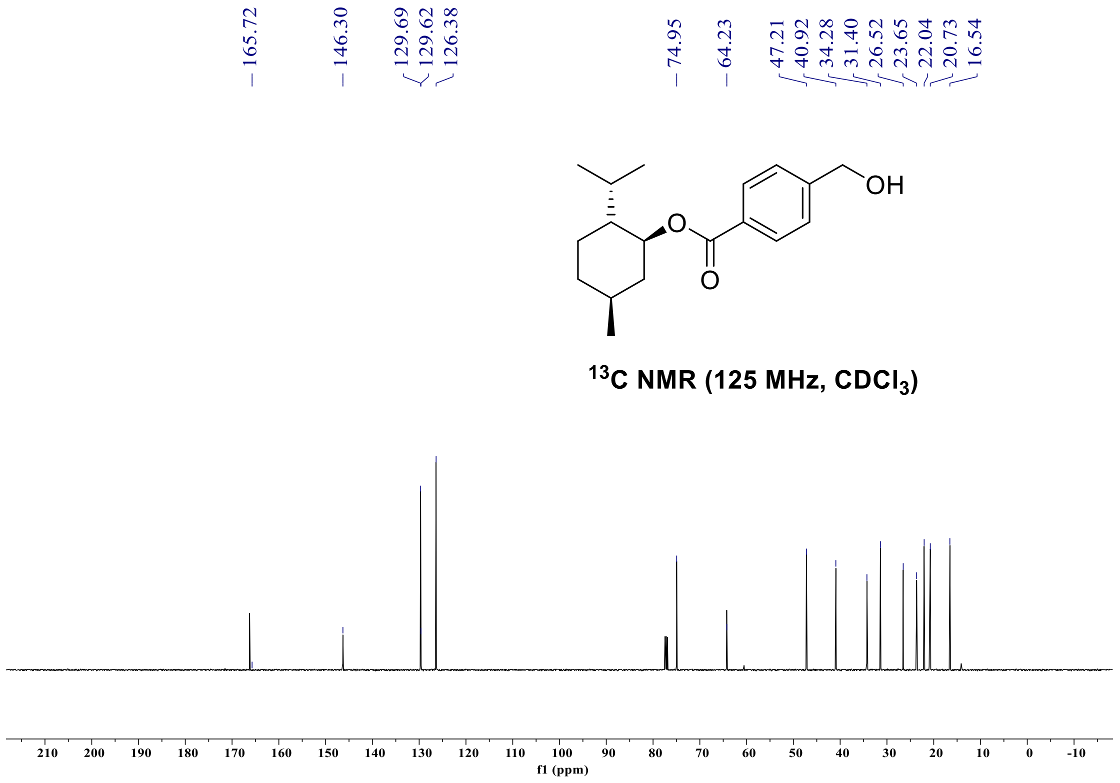

4-Allyl-2-methoxyphenyl 4-(((dimethyl(phenyl)silyl)oxy)methyl)benzoate (43)

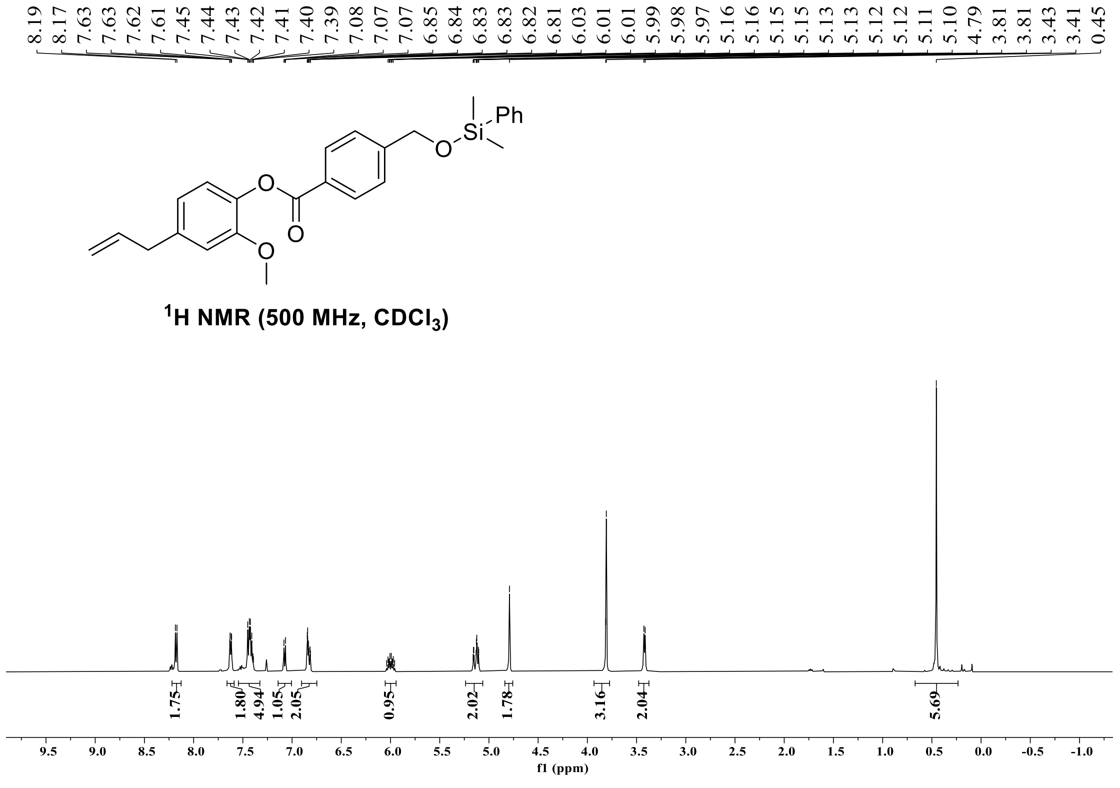

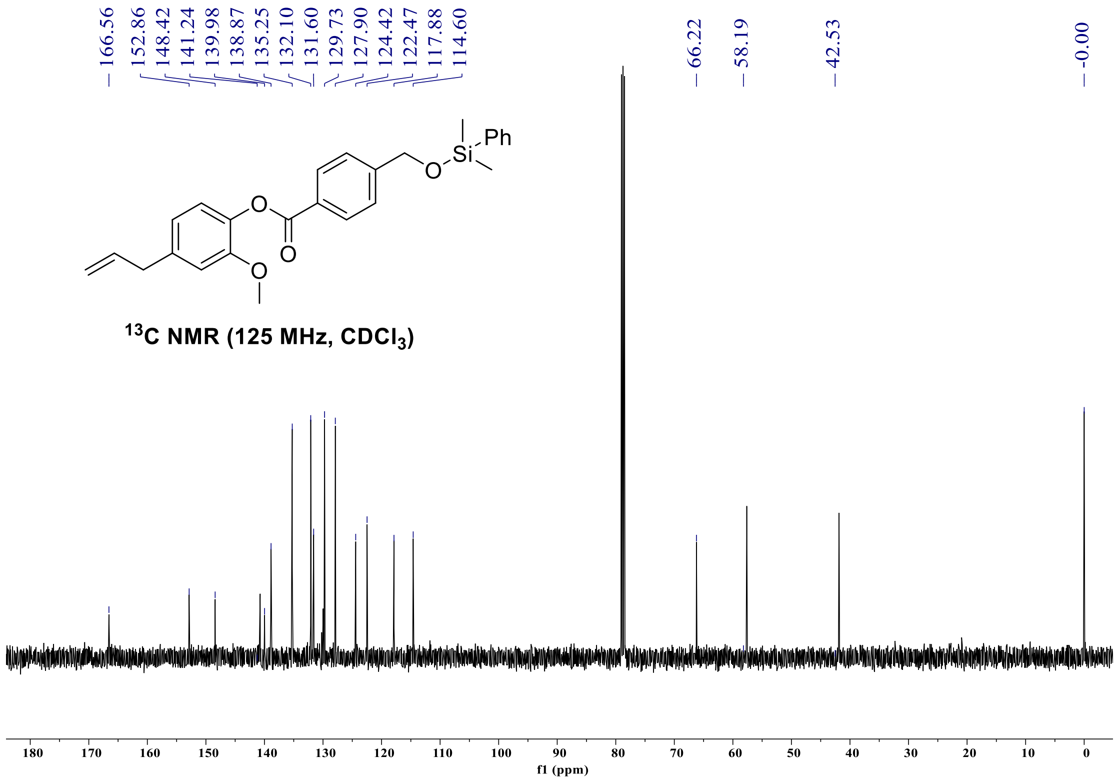

N-(4-(hydroxymethyl)phenyl)-2-(6-methoxynaphthalen-2-yl)propanamide (44)

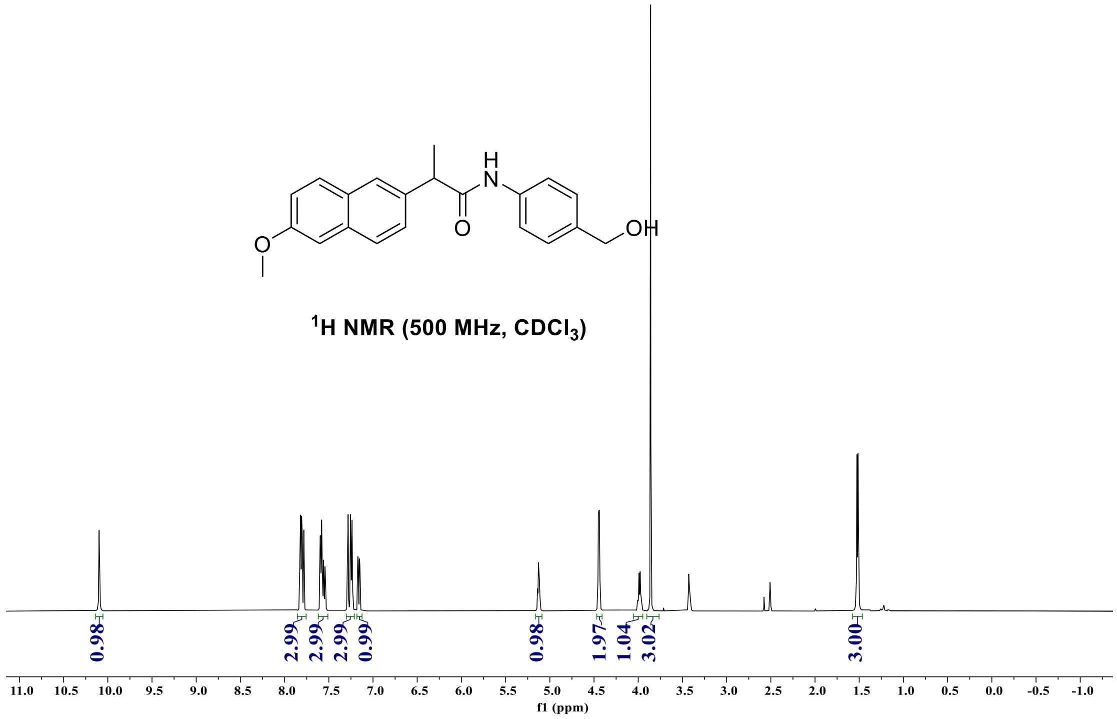

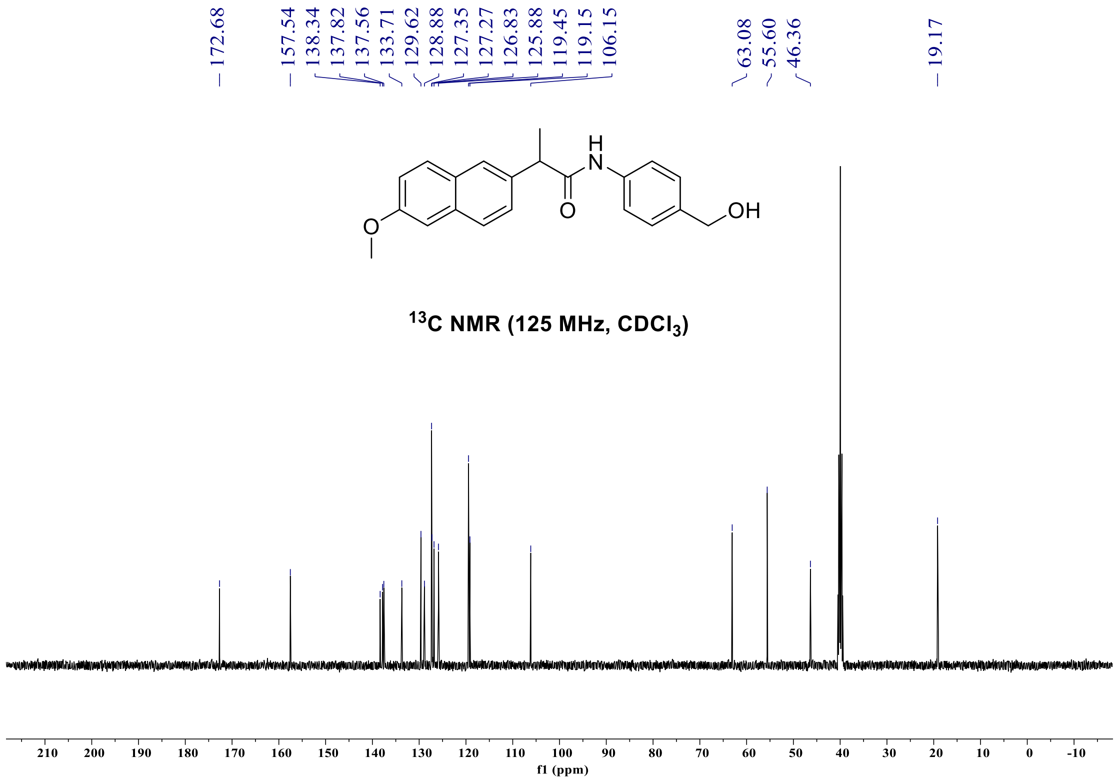

(3*S*,8*S*,9*S*,10*R*,13*R*,14*S*,17*R*)-10,13-Dimethyl-17-((*R*)-6-methylheptan-2-yl)-2,3,4,7,8,9,10,11,12,13,14,15,16,17-tetradecahydro-1*H*-cyclopenta[a]phenanthren-3-yl 4-(hydroxymethyl)benzoate (45)

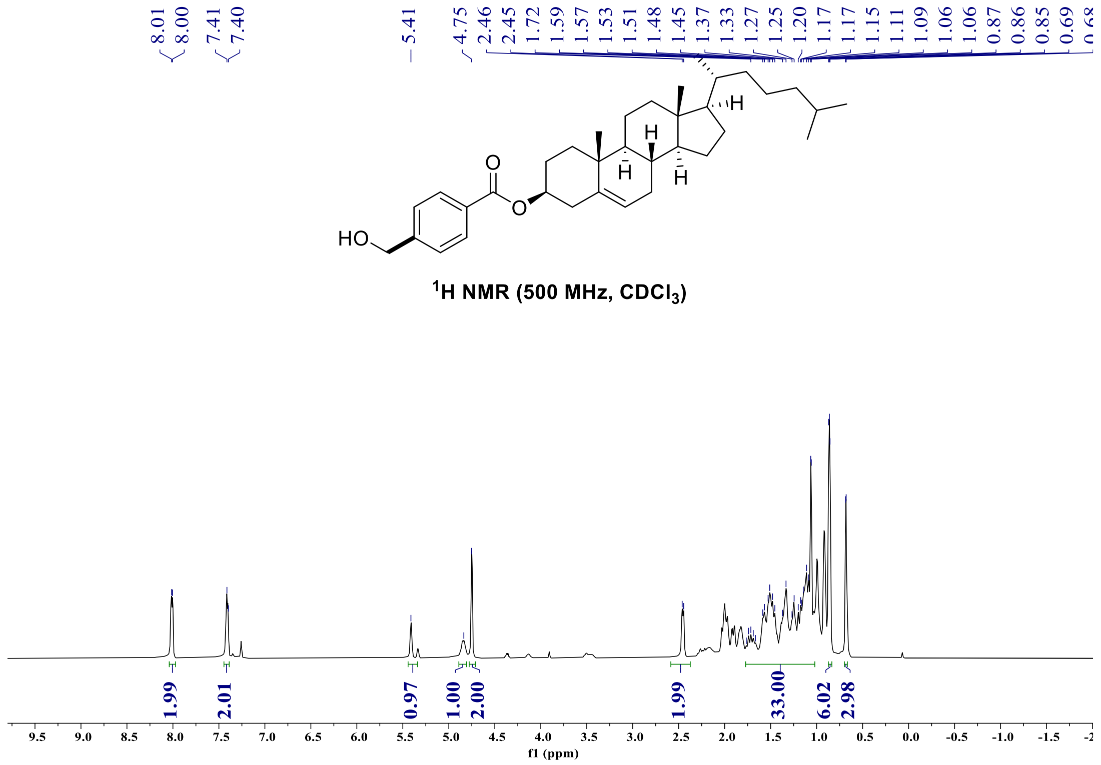

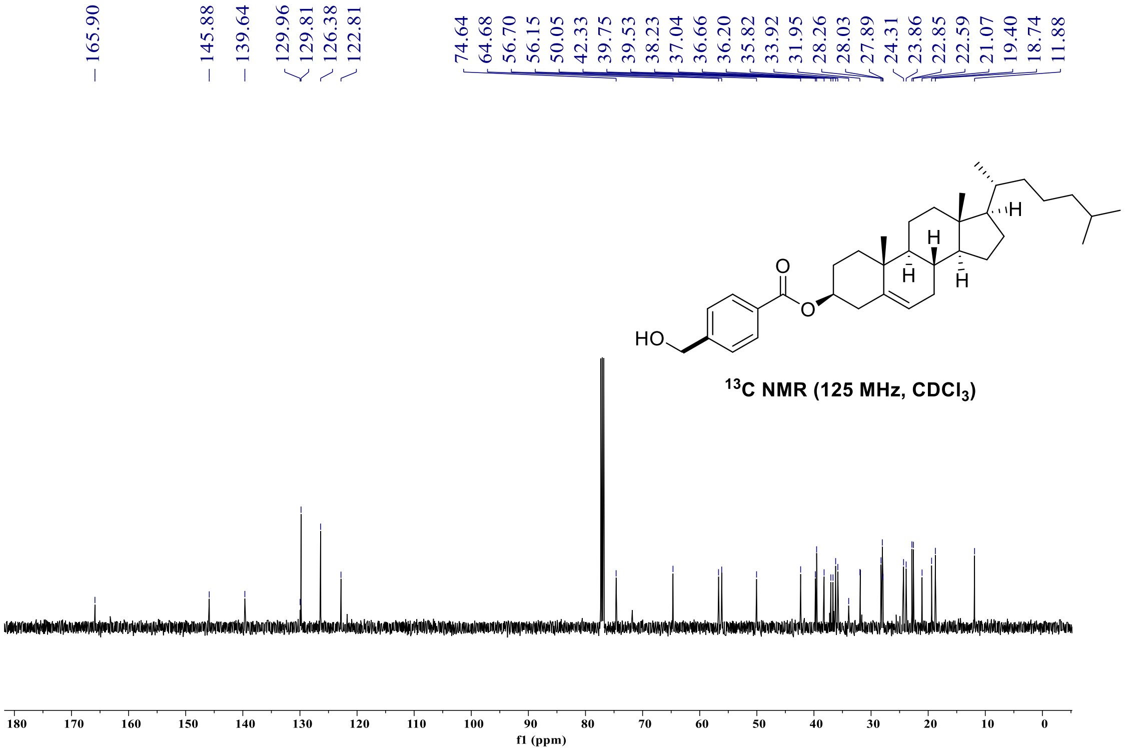

N-(4-(hydroxymethyl)phenyl)-2-(4-isobutylphenyl)propanamide (46)

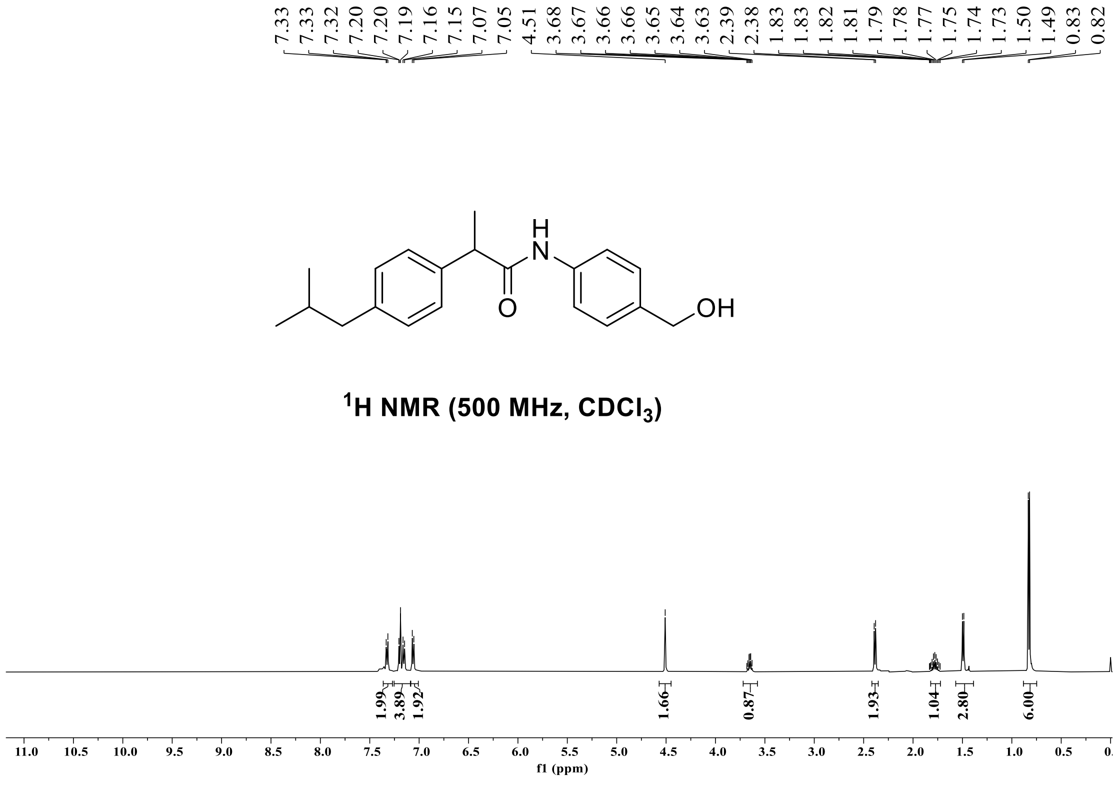

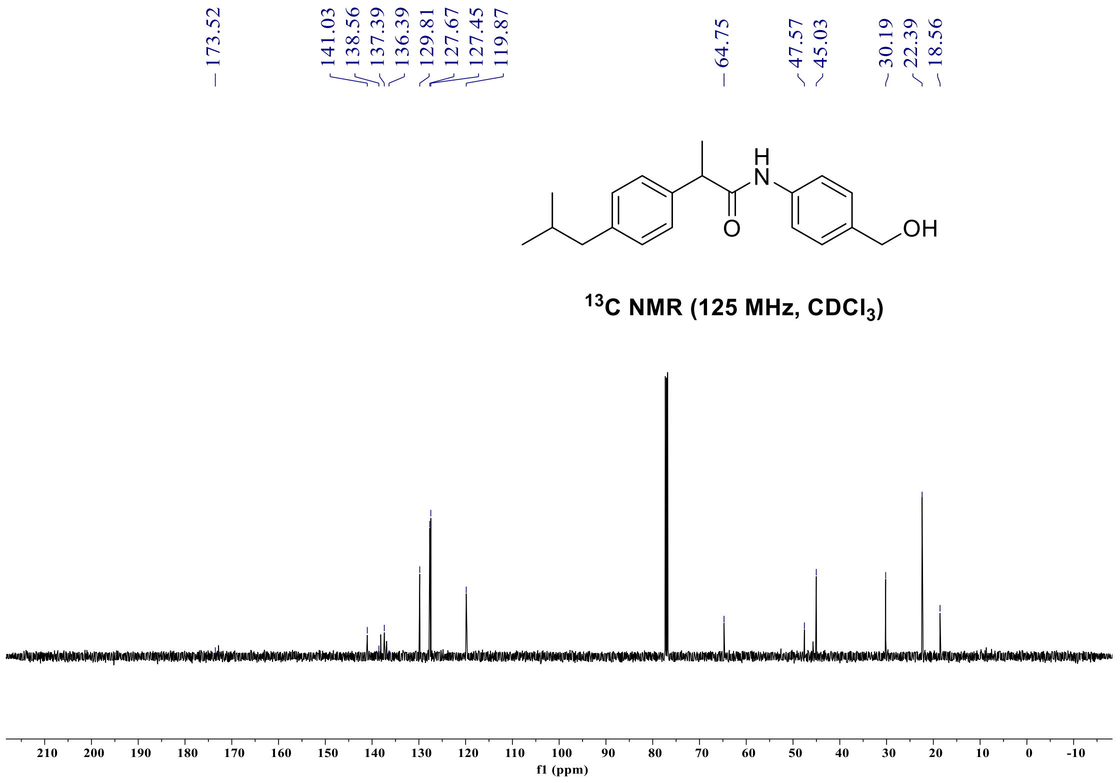

4-(*N*,*N*-dipropylsulfamoyl)-*N*-(4-(hydroxymethyl)phenyl)benzamide (47)

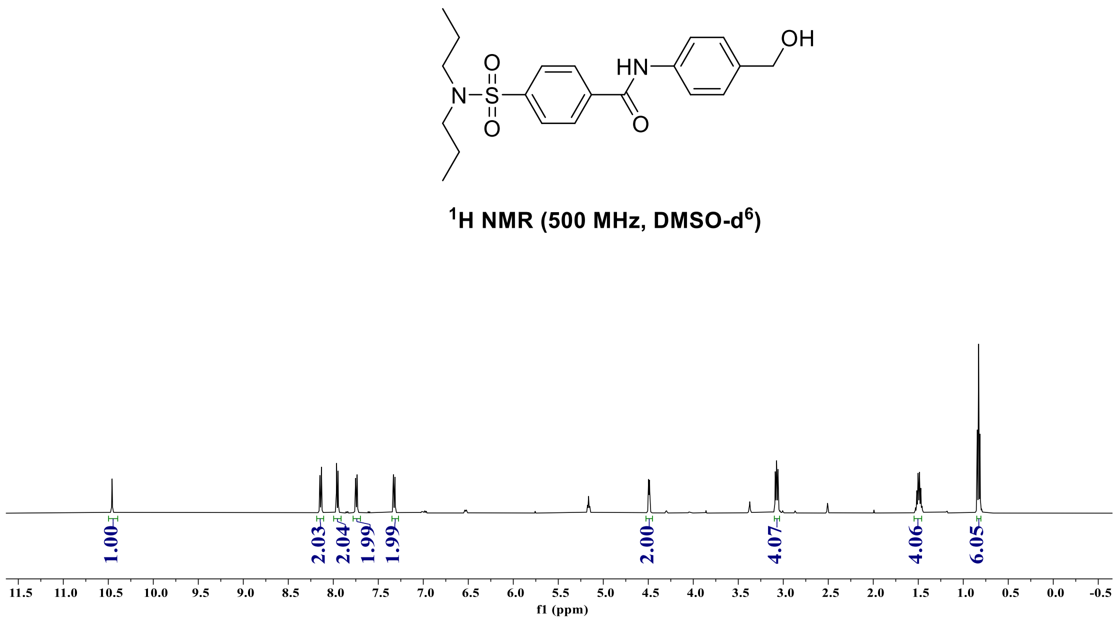

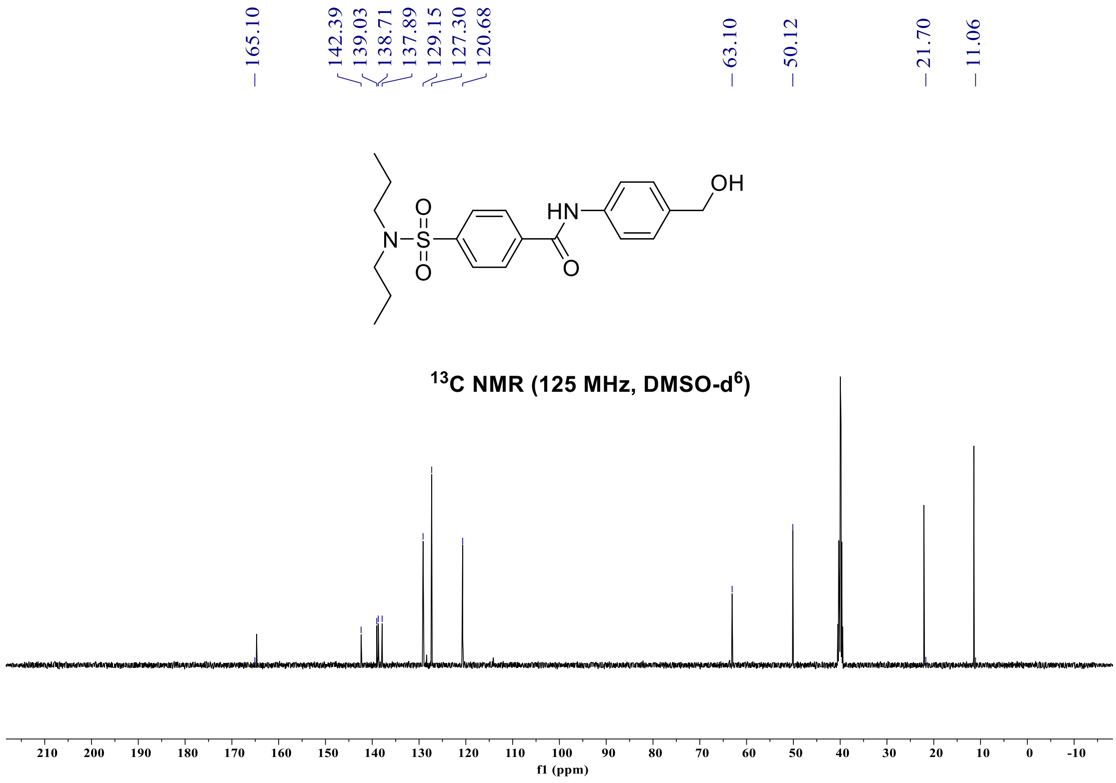

2-(hydroxymethyl)phenol

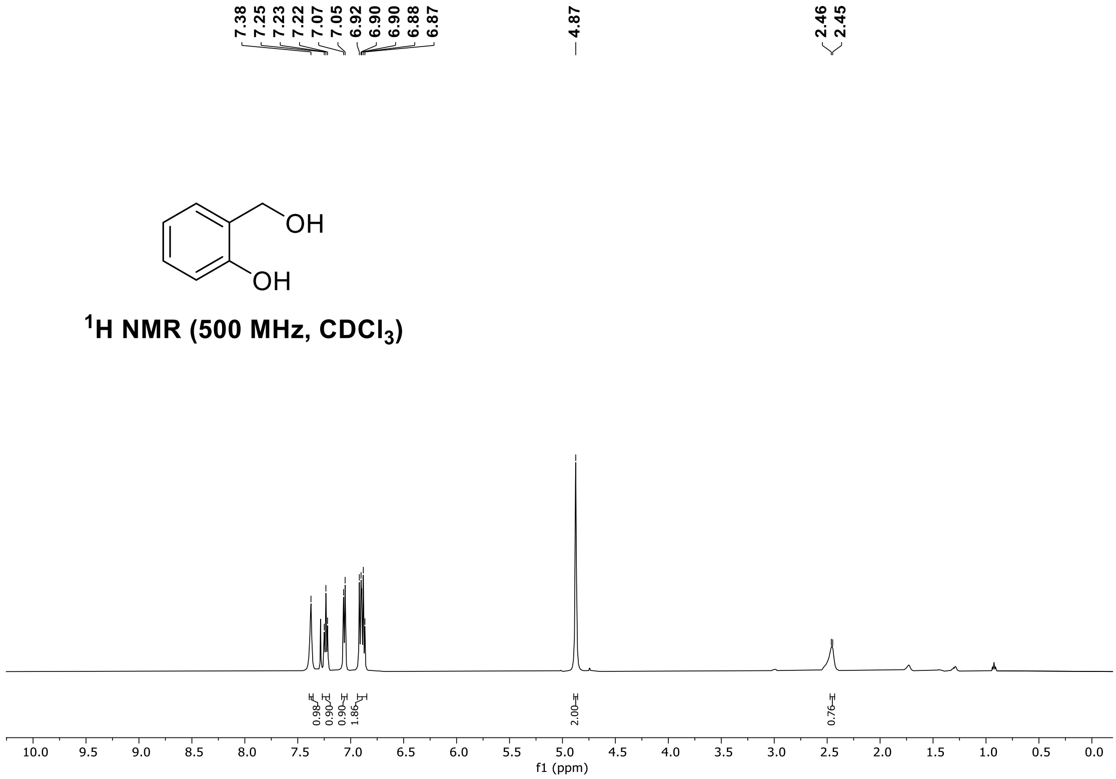

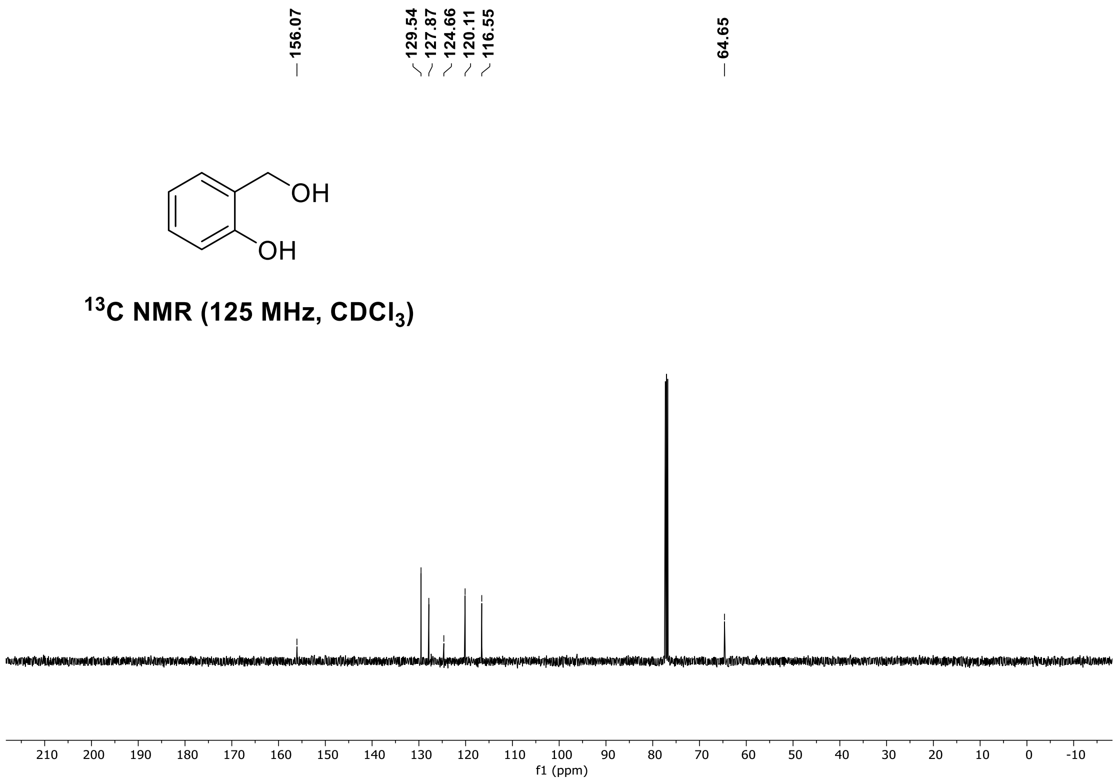

2-hydroxybenzoic acid

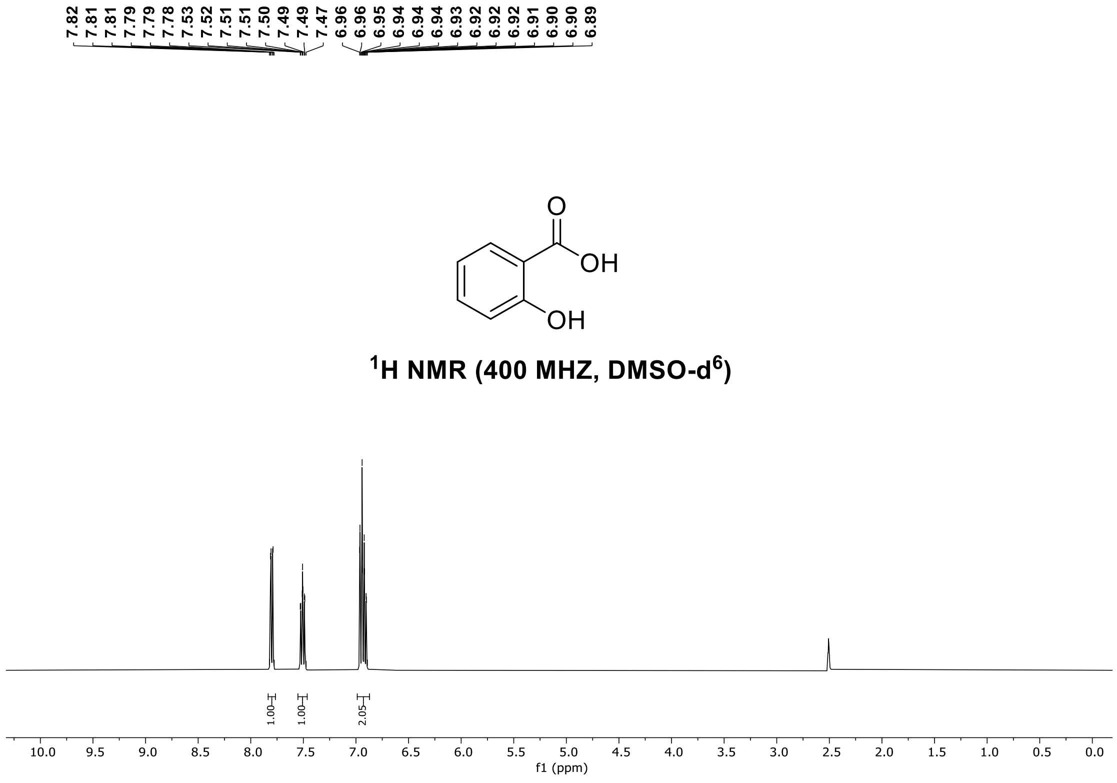

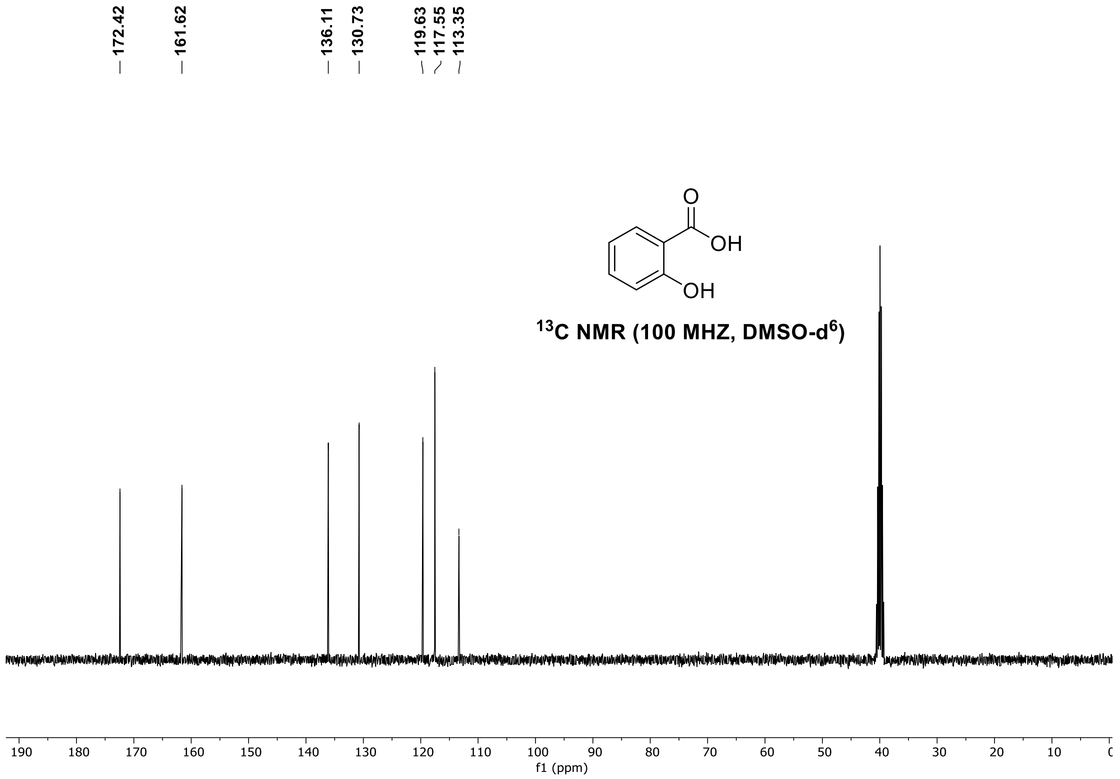

2-hydroxybenzaldehyde

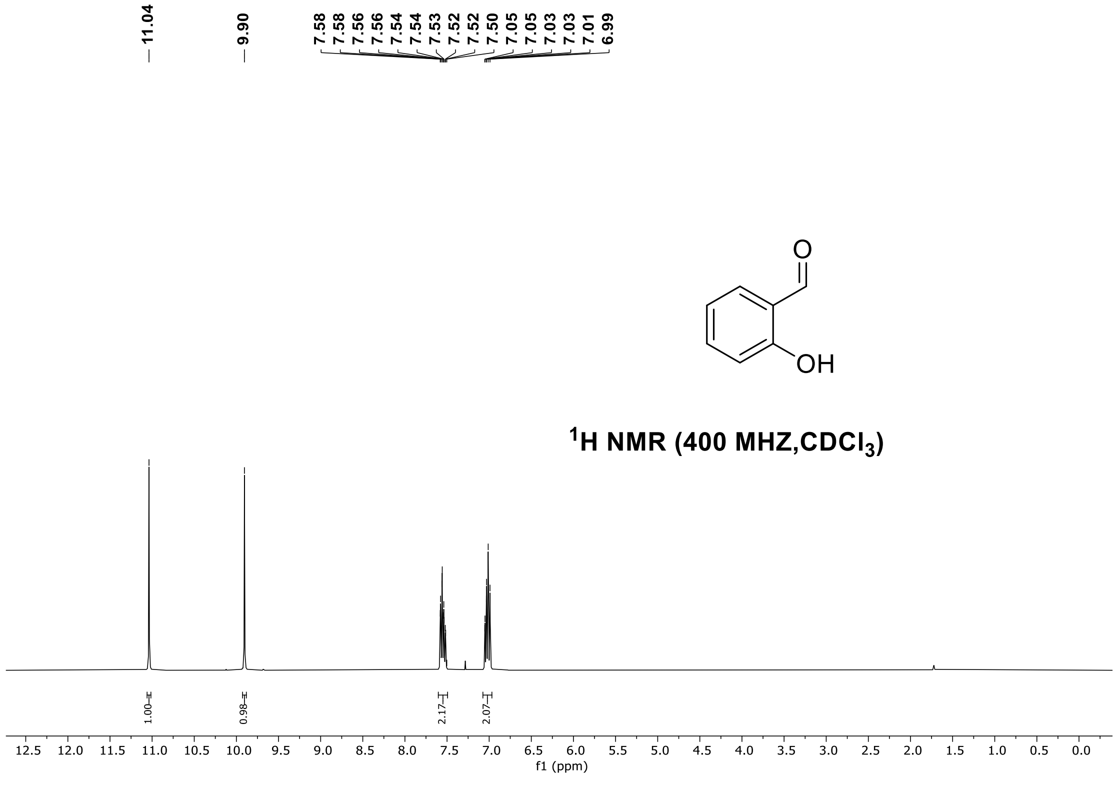

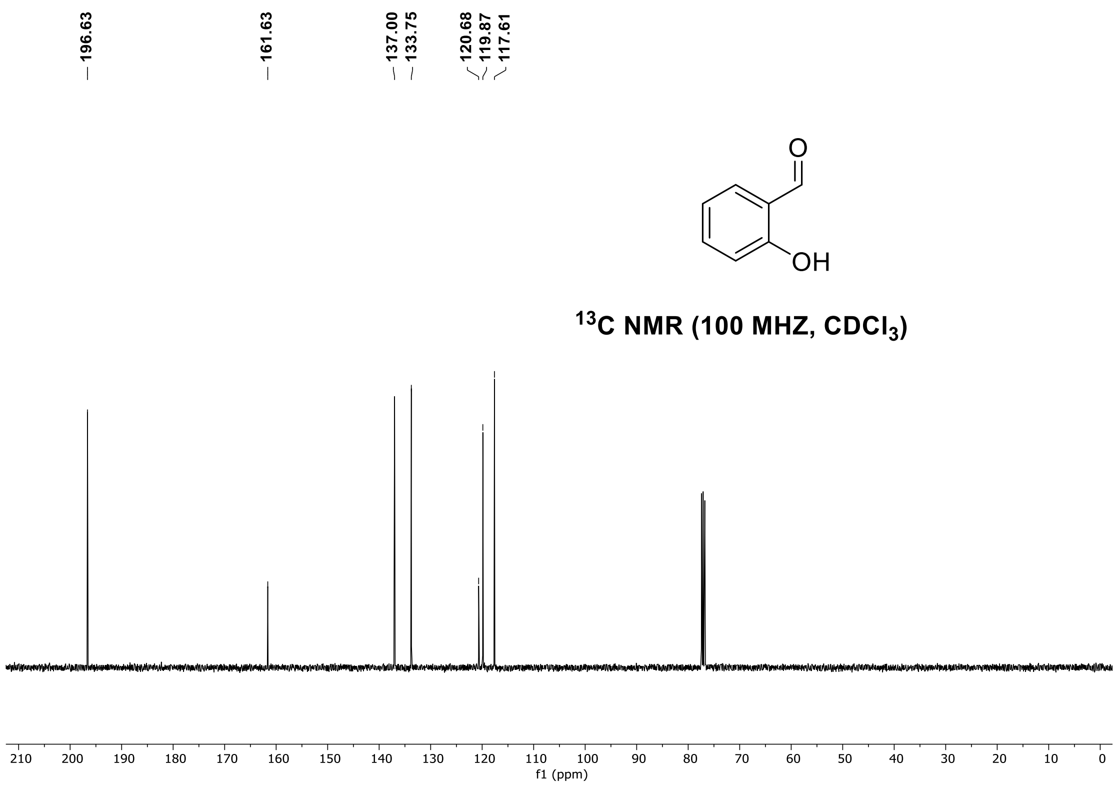

2-hydroxybenzonitrile

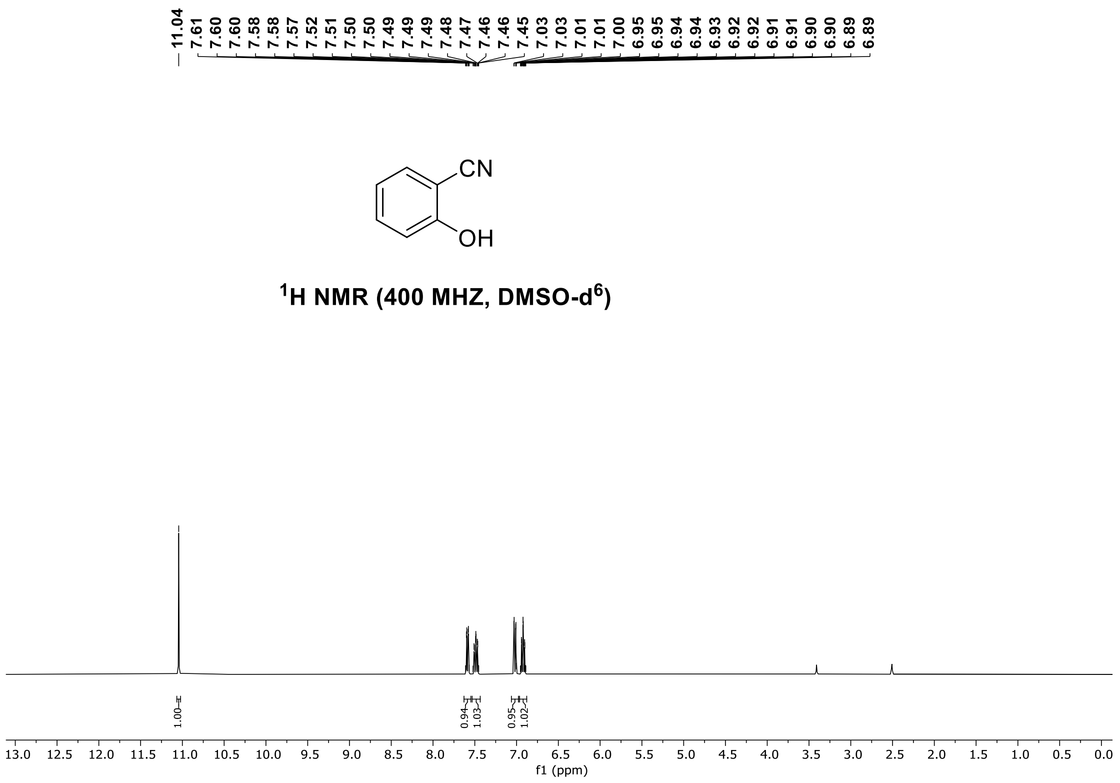

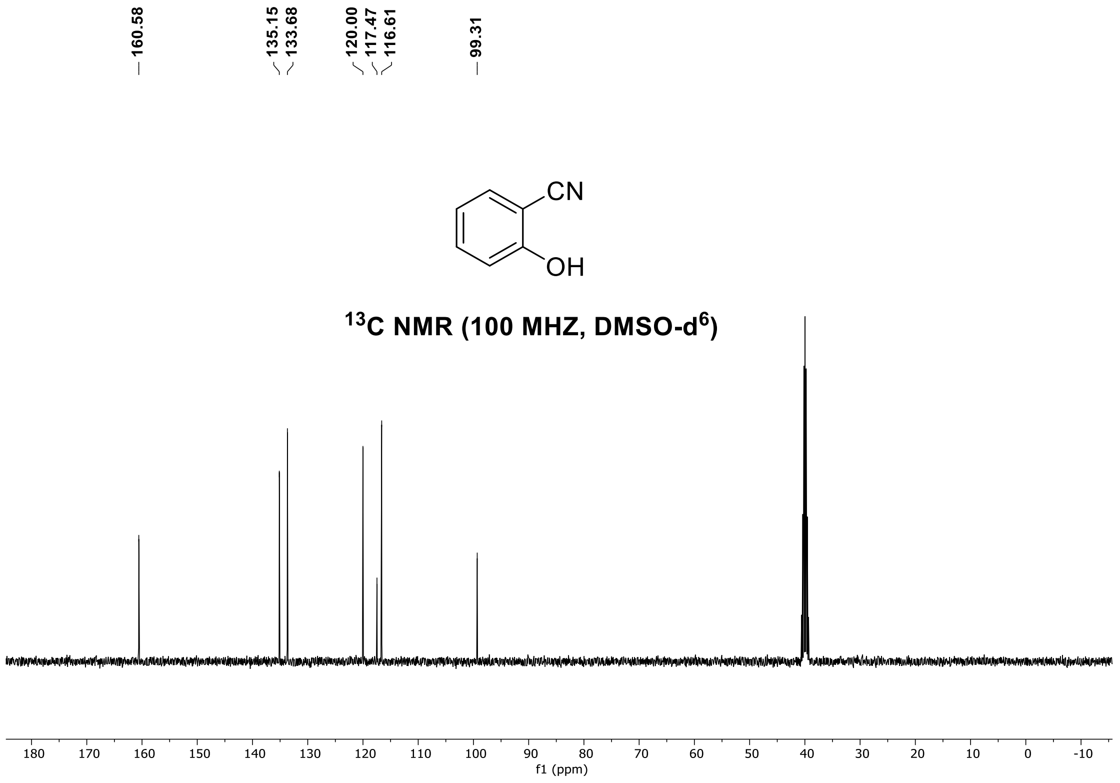

^{13}C NMR (100 MHZ, DMSO-d^{6})

Benzofuran-2(3H)-one

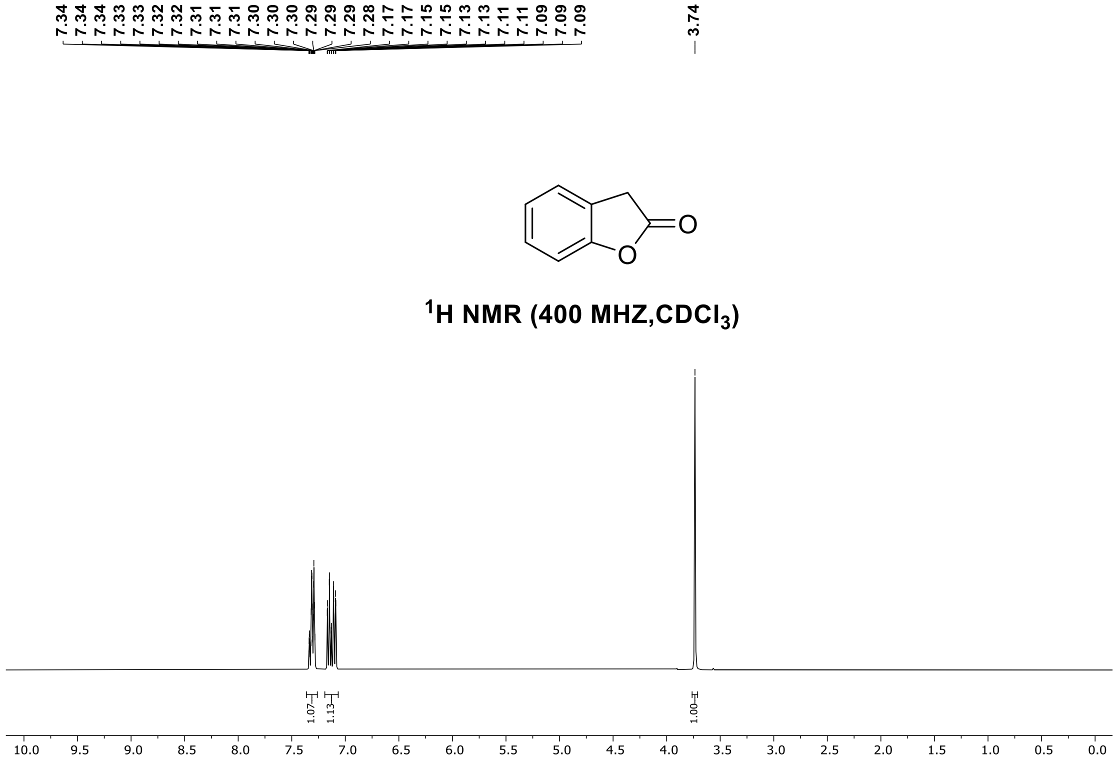

^{1}H NMR (400 MHZ,CDCl$_3$)

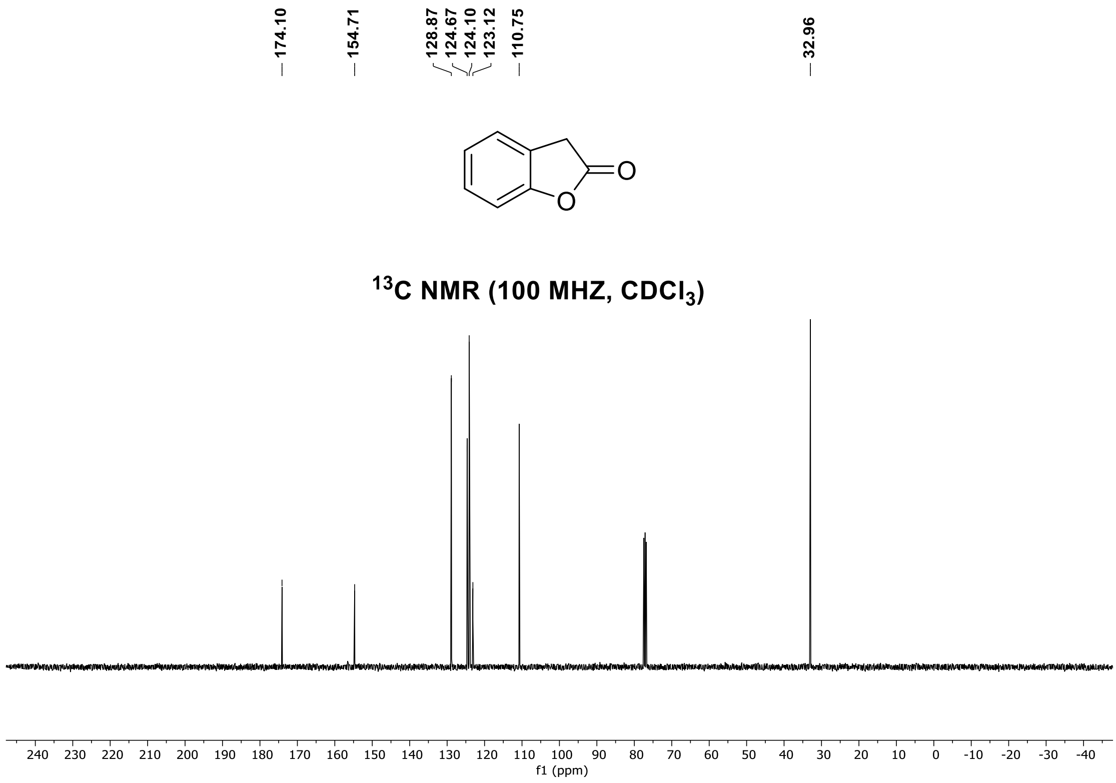

benzo[d]isoxazole

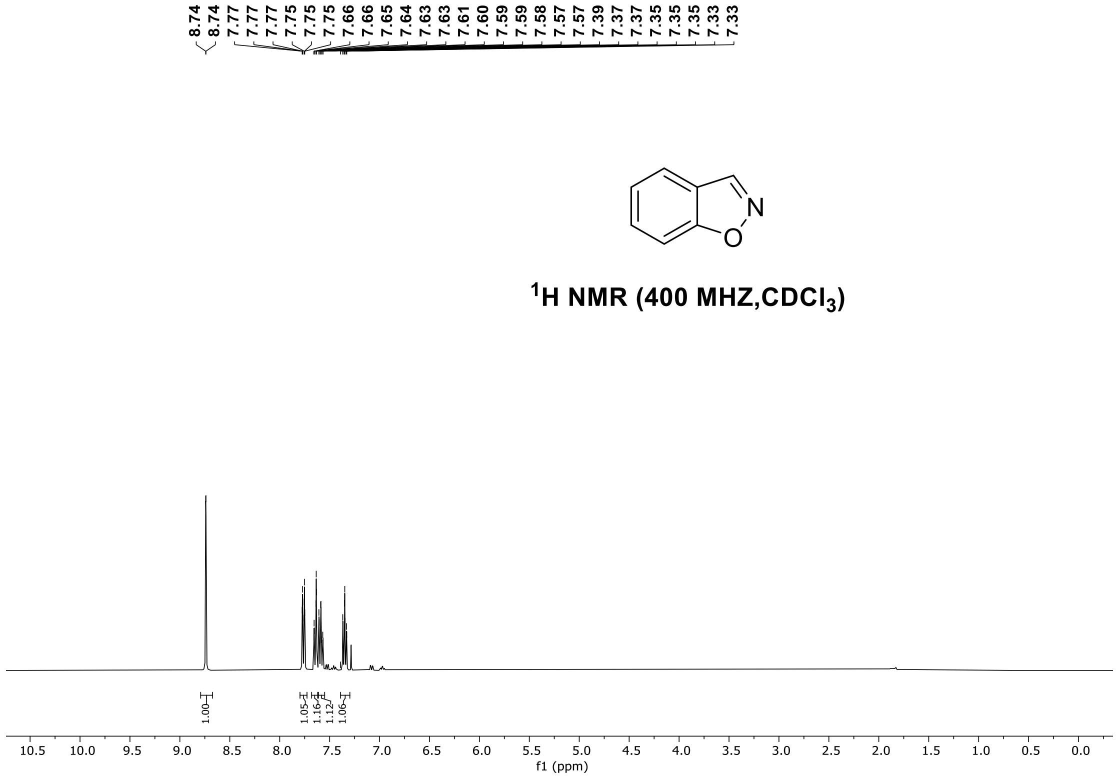

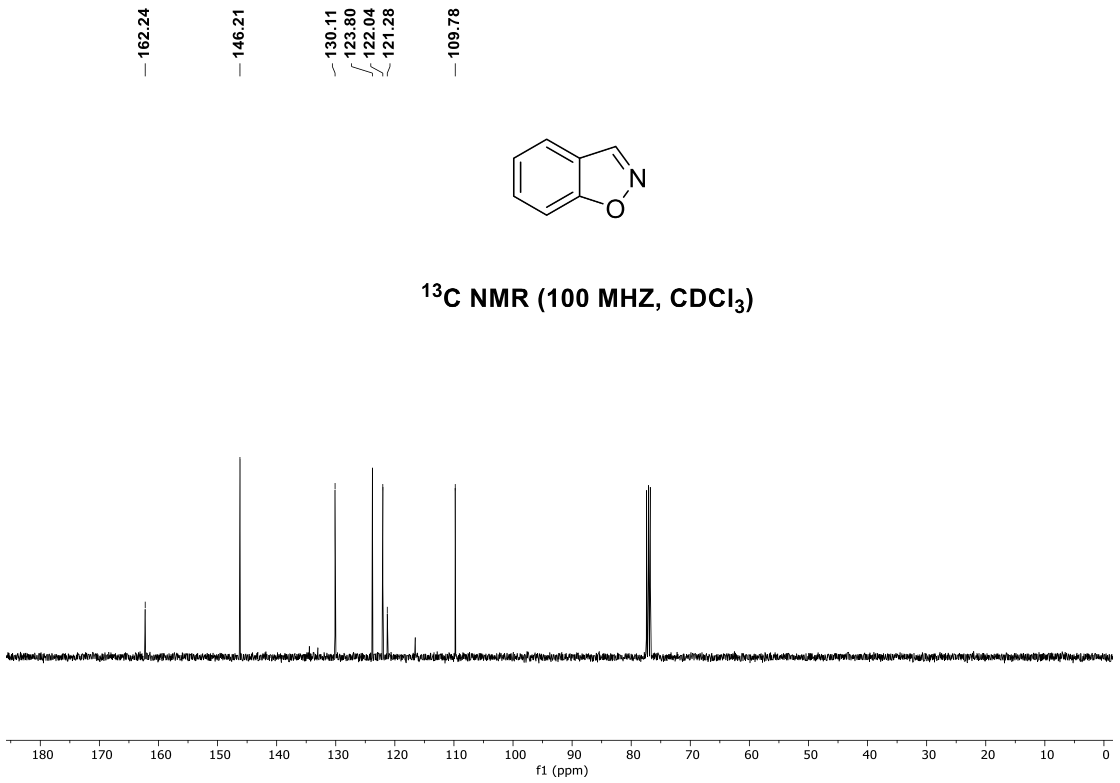

Adapalene (49)

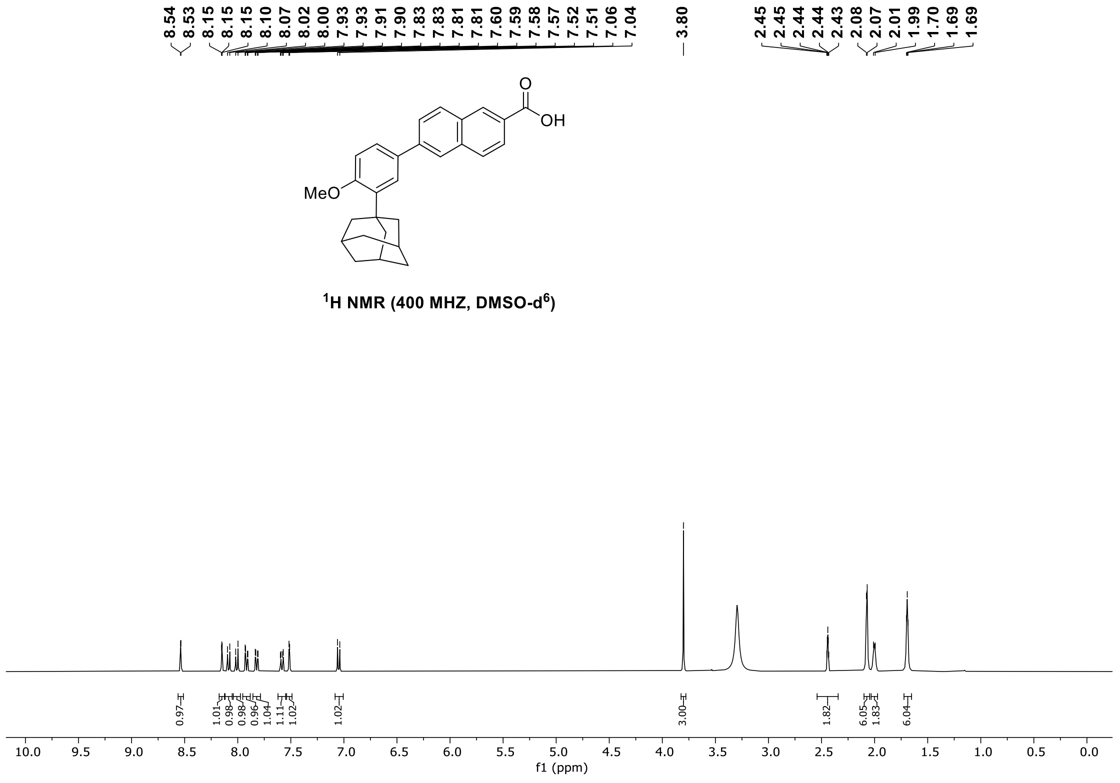

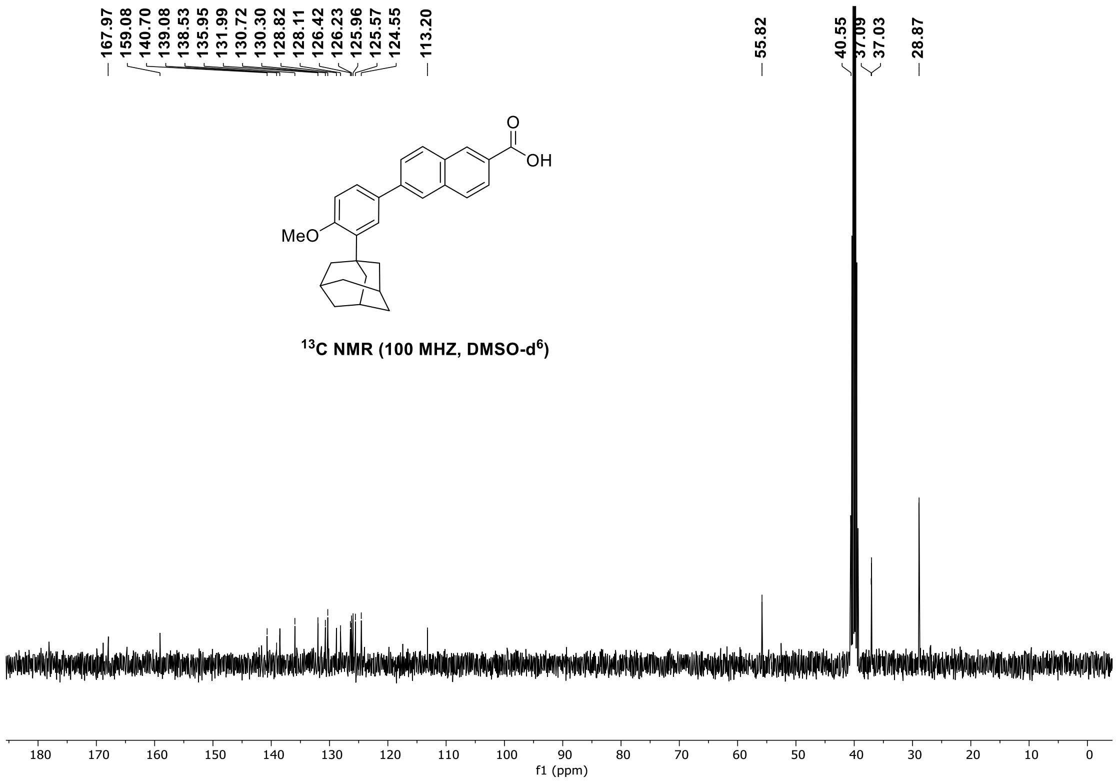

N-benzyl-4-((dimethyl(phenyl)silyl)oxy)butanamide (52)

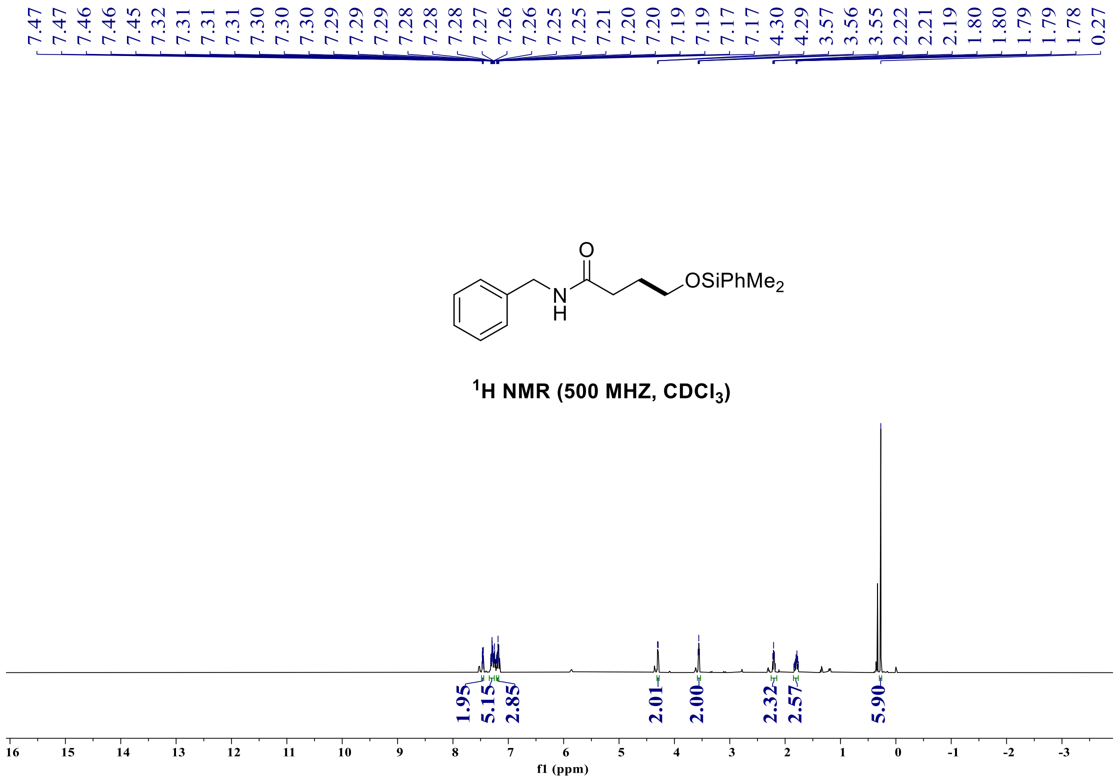

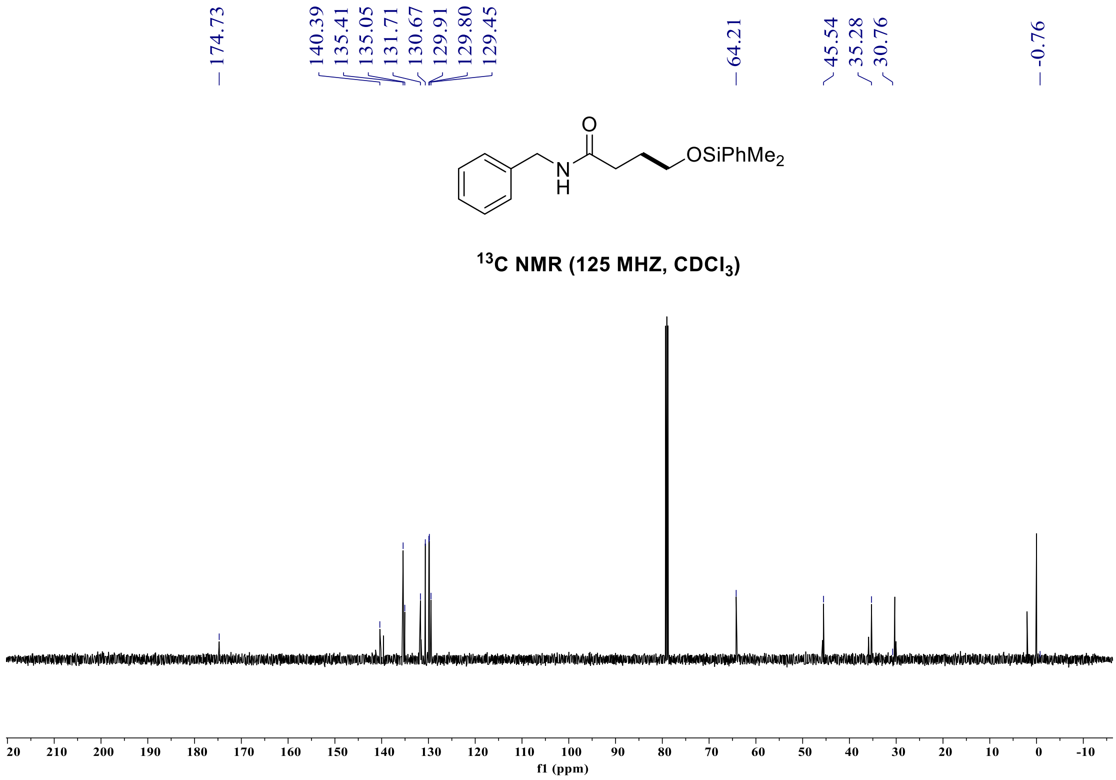

Phthalimide

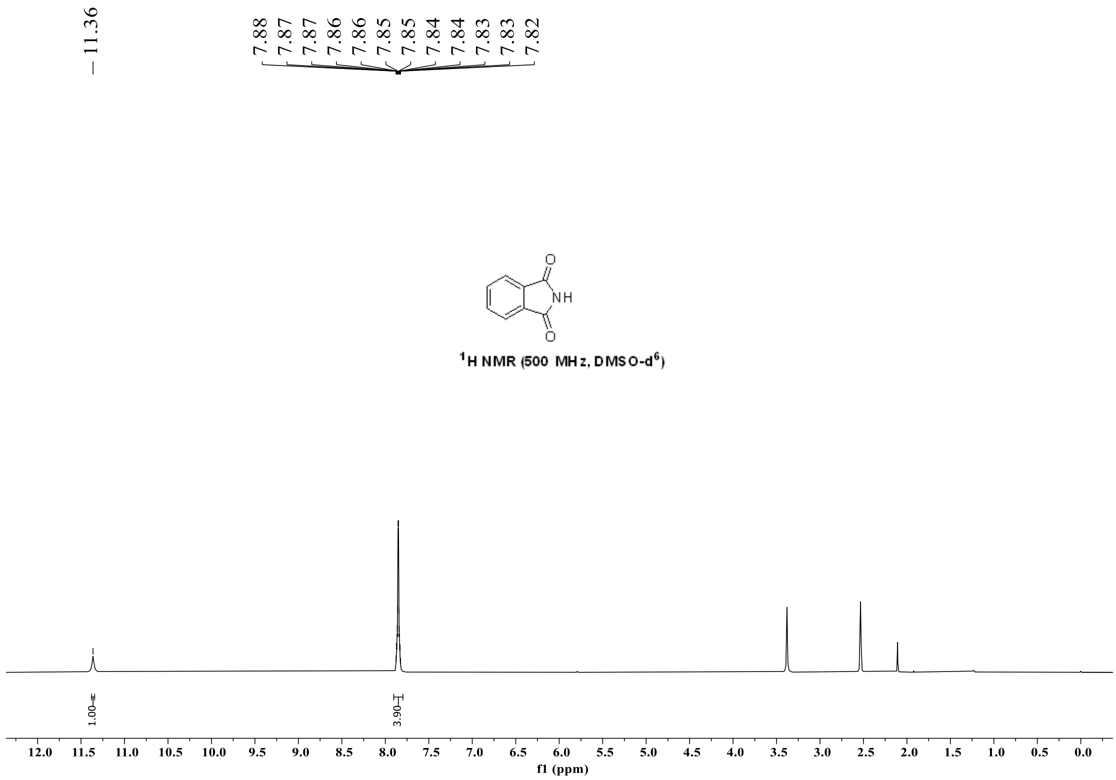

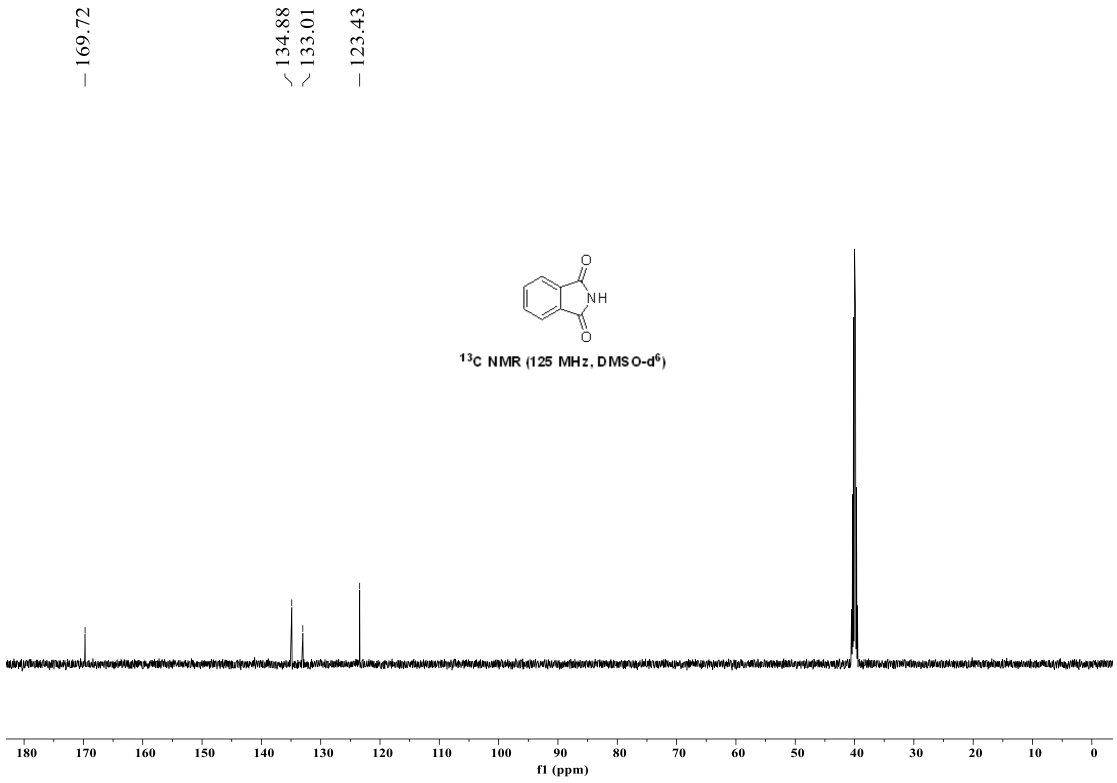
169.72
134.88
133.01
123.43
NH
O
O
13C NMR (125 MHz, DMSO-d6)
180 170 160 150 140 130 120 110 100 90 80 70 60 50 40 30 20 10 0
f1 (ppm)
